# Supplementary material for: Modelling maternal and perinatal risk factors to predict poorly controlled childhood asthma
Source: PLoS One. 2021 May 27;16(5):e0252215. doi: 10.1371/journal.pone.0252215 (PMC8158992; doi:10.1371/journal.pone.0252215)
Supplement: S1 Fig — (PDF) [file pone.0252215.s001.pdf]

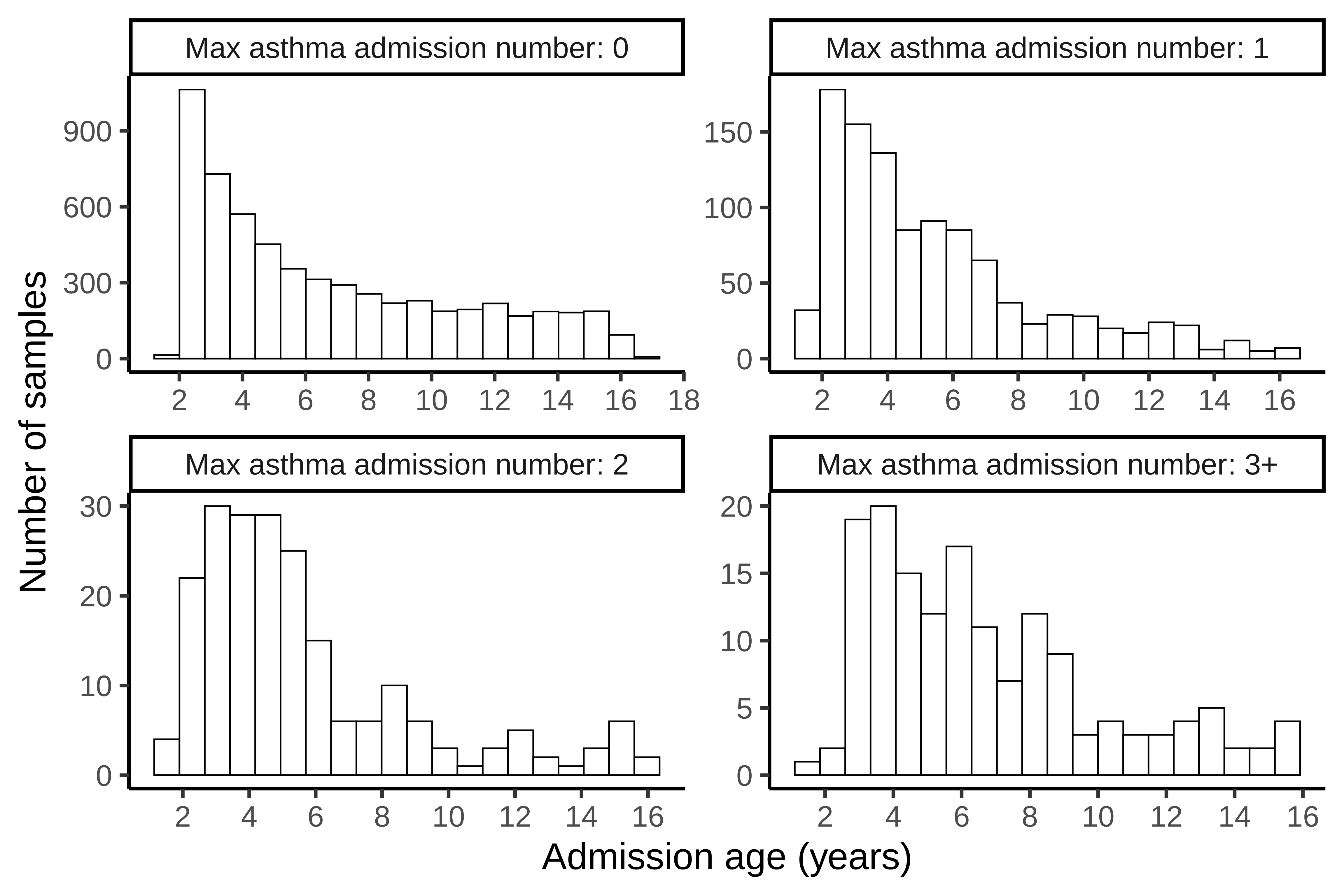
Max asthma admission number: 0
Max asthma admission number: 1
Max asthma admission number: 2
Max asthma admission number: 3+
Number of samples
Admission age (years)
0
300
600
900
0
50
100
150
0
10
20
30
0
5
10
15
20
2
4
6
8
10
12
14
16
18
