# Supplementary figures and images for: Modelling maternal and perinatal risk factors to predict poorly controlled childhood asthma
Source: PLoS One. 2021 May 27;16(5):e0252215. doi: 10.1371/journal.pone.0252215 (PMC8158992; doi:10.1371/journal.pone.0252215)

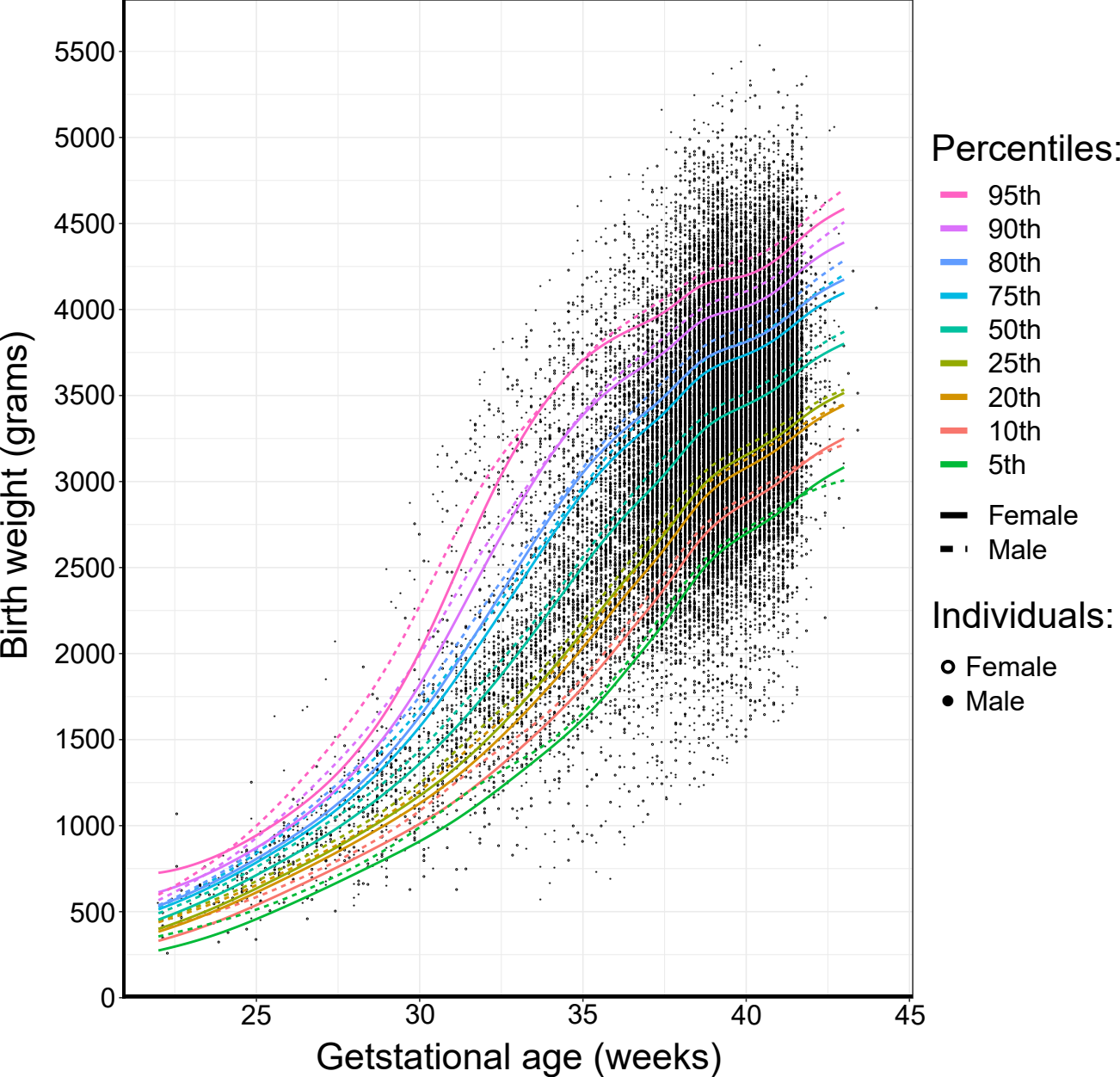

Supplement: S2 Fig — Children below 32 weeks gestation were excluded from our study population, but as growth references for small gestational ages are scarce growth curves for future reference were created starting at 22 weeks gestation. One dot represents one child. (PDF) [file pone.0252215.s002.pdf]

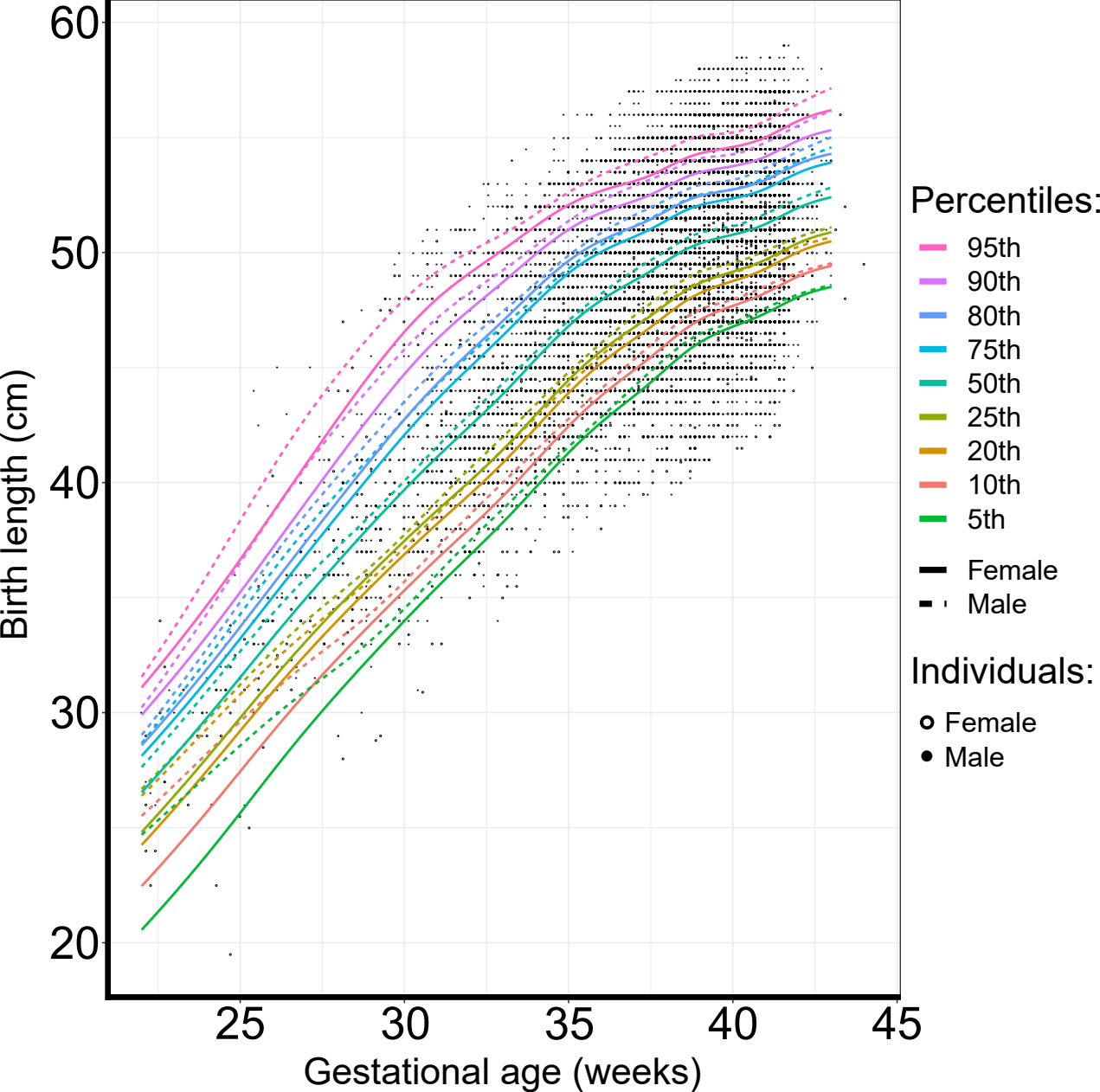

Supplement: S3 Fig — Children below 32 weeks gestation were excluded from our study population, but as growth references for small gestational ages are scarce growth curves for future reference were created starting at 22 weeks gestation. One dot represents one child. (PDF) [file pone.0252215.s003.pdf]

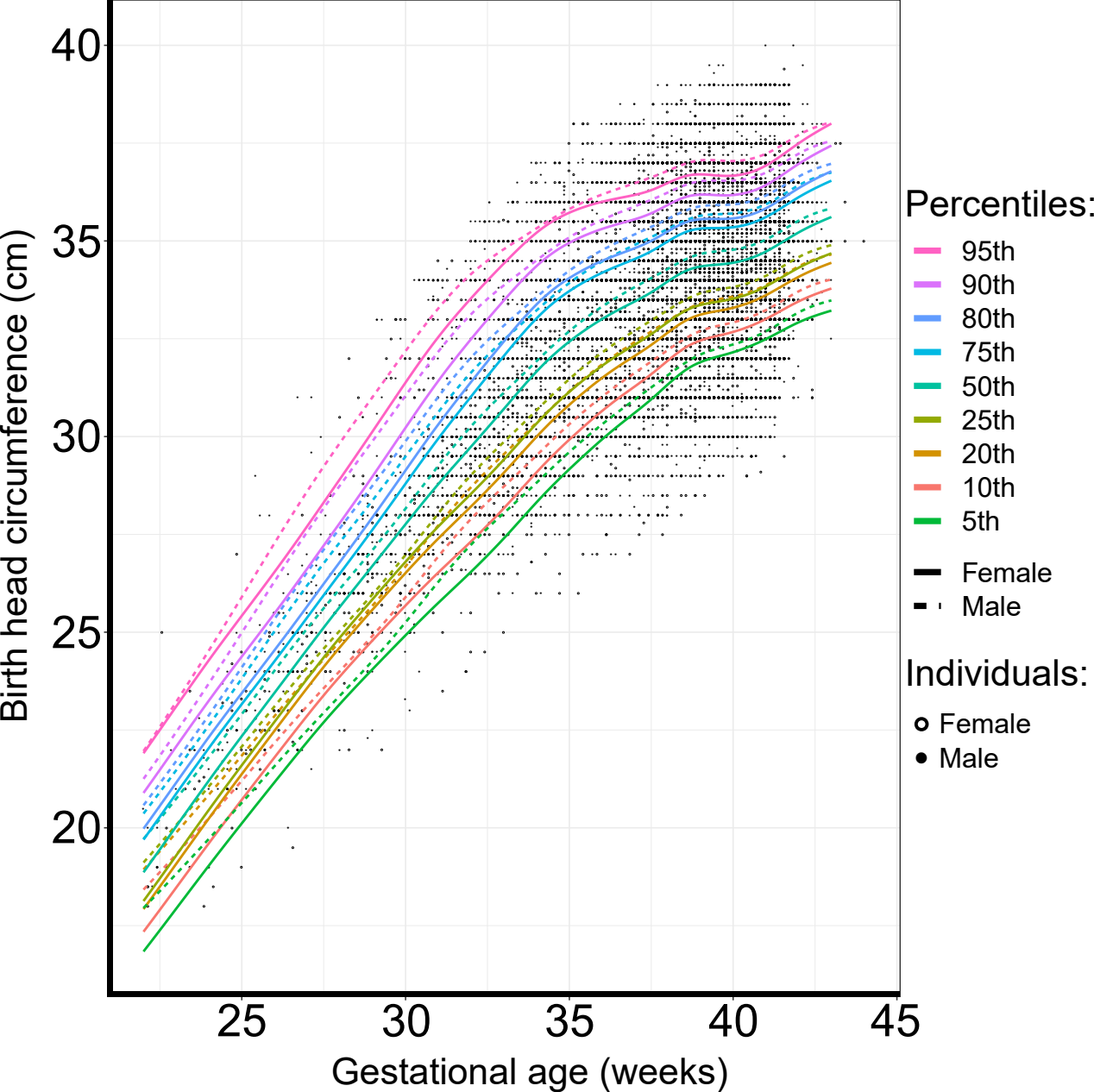

Supplement: S4 Fig — Children below 32 weeks gestation were excluded from our study population, but as growth references for small gestational ages are scarce growth curves for future reference were created starting at 22 weeks gestation. One dot represents one child. (PDF) [file pone.0252215.s004.pdf]

Birth weight, post 32 weeks gestation

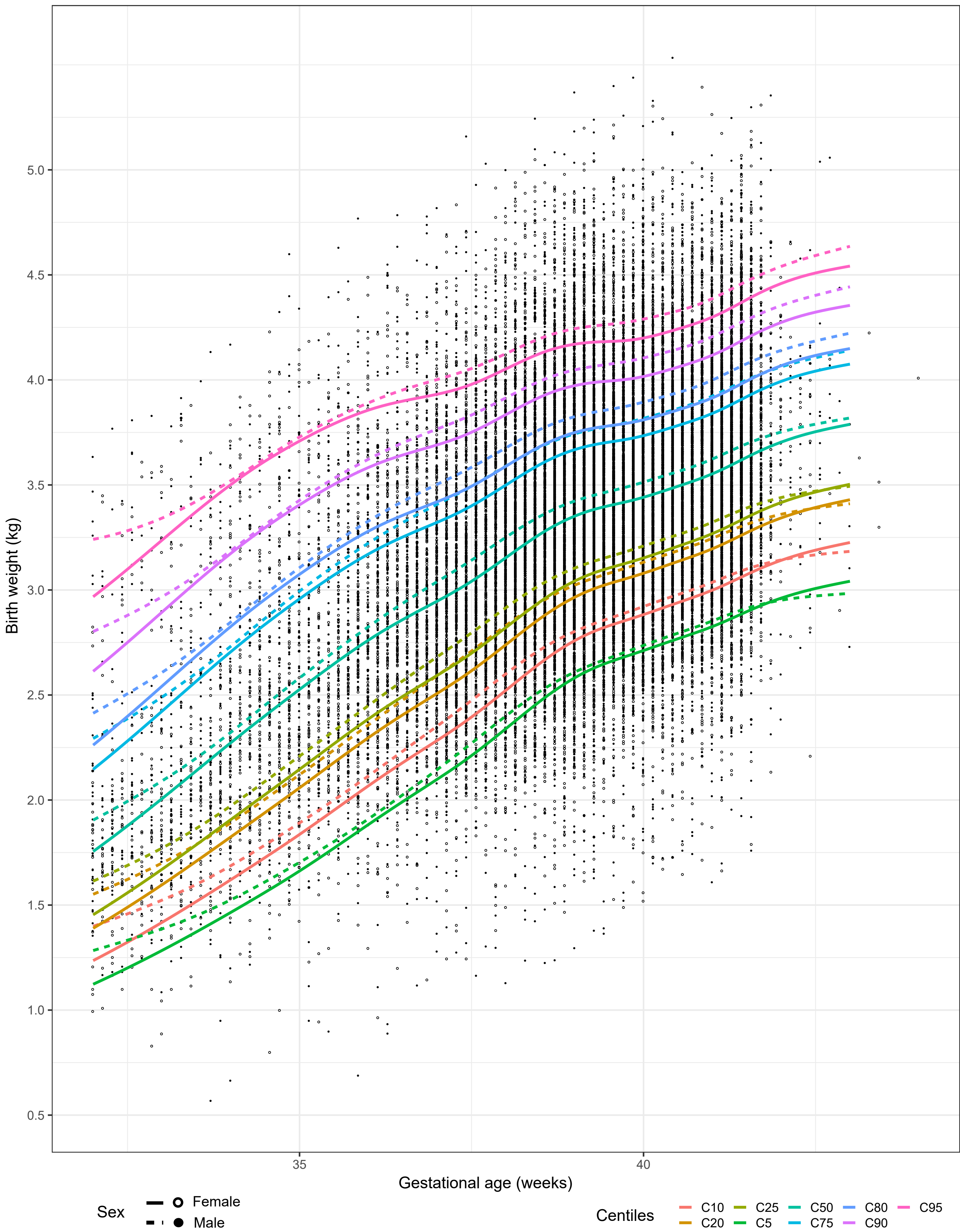

Supplement: S5 Fig — (PDF) [file pone.0252215.s005.pdf]

Birth length, post 32 weeks

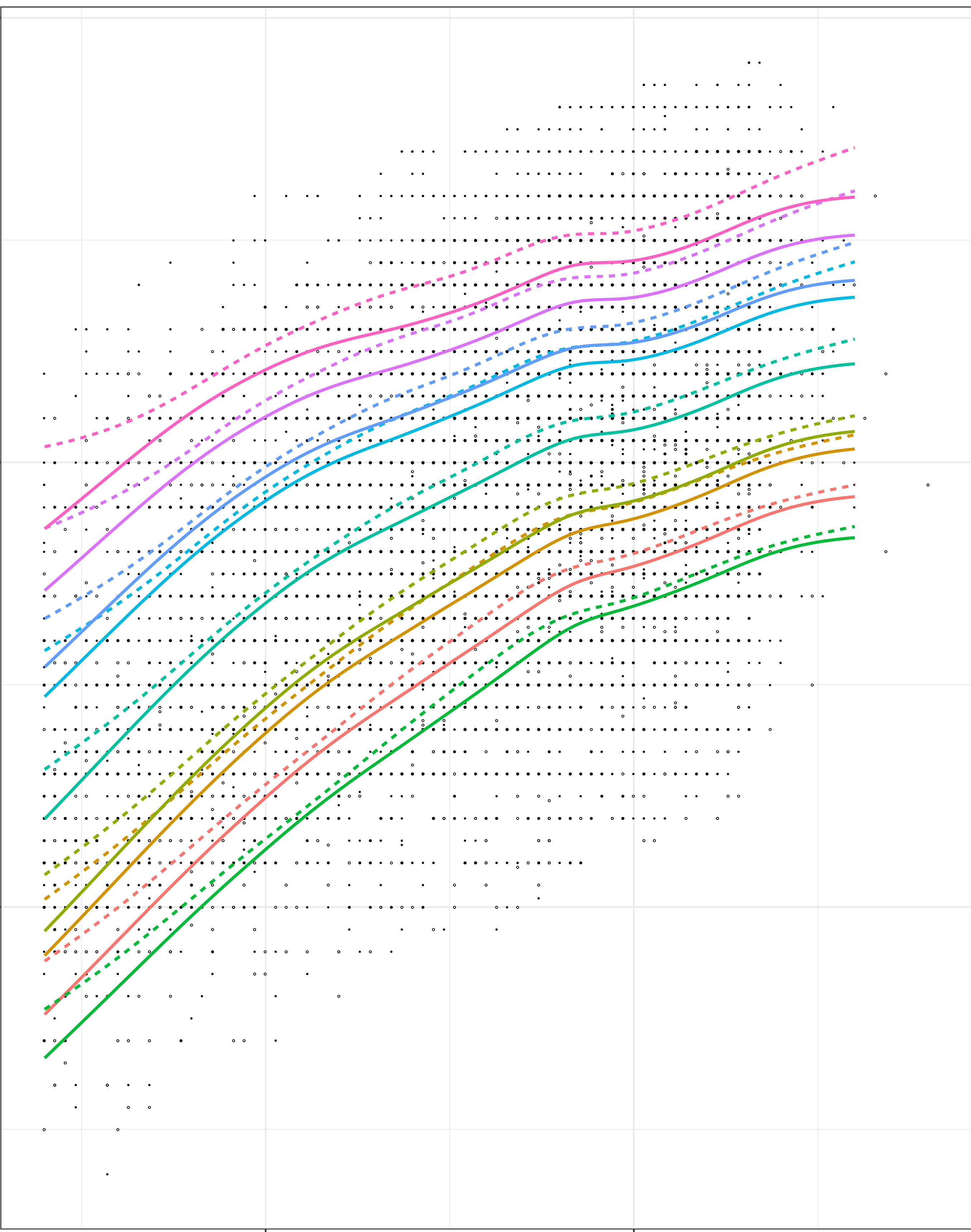

Sex    — ○ Female  
      - - ● Male

Centile    — C10    — C25    — C50    — C80    — C95  
            - - C20    - - C5    - - C75    - - C90

Supplement: S6 Fig — (PDF) [file pone.0252215.s006.pdf]

Birth head circumference, post 32 weeks

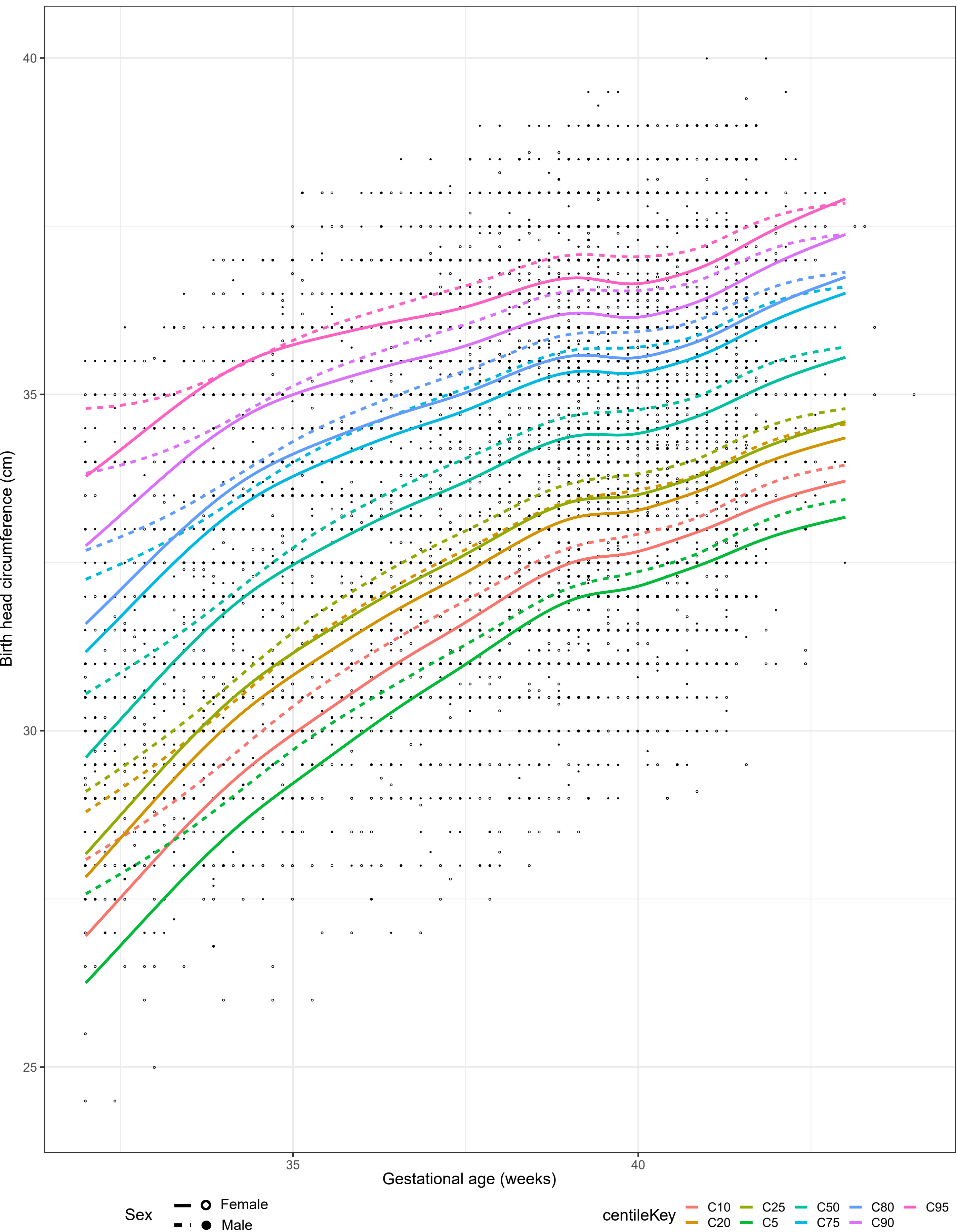

Supplement: S7 Fig — (PDF) [file pone.0252215.s007.pdf]

Variable inclusion tile plot

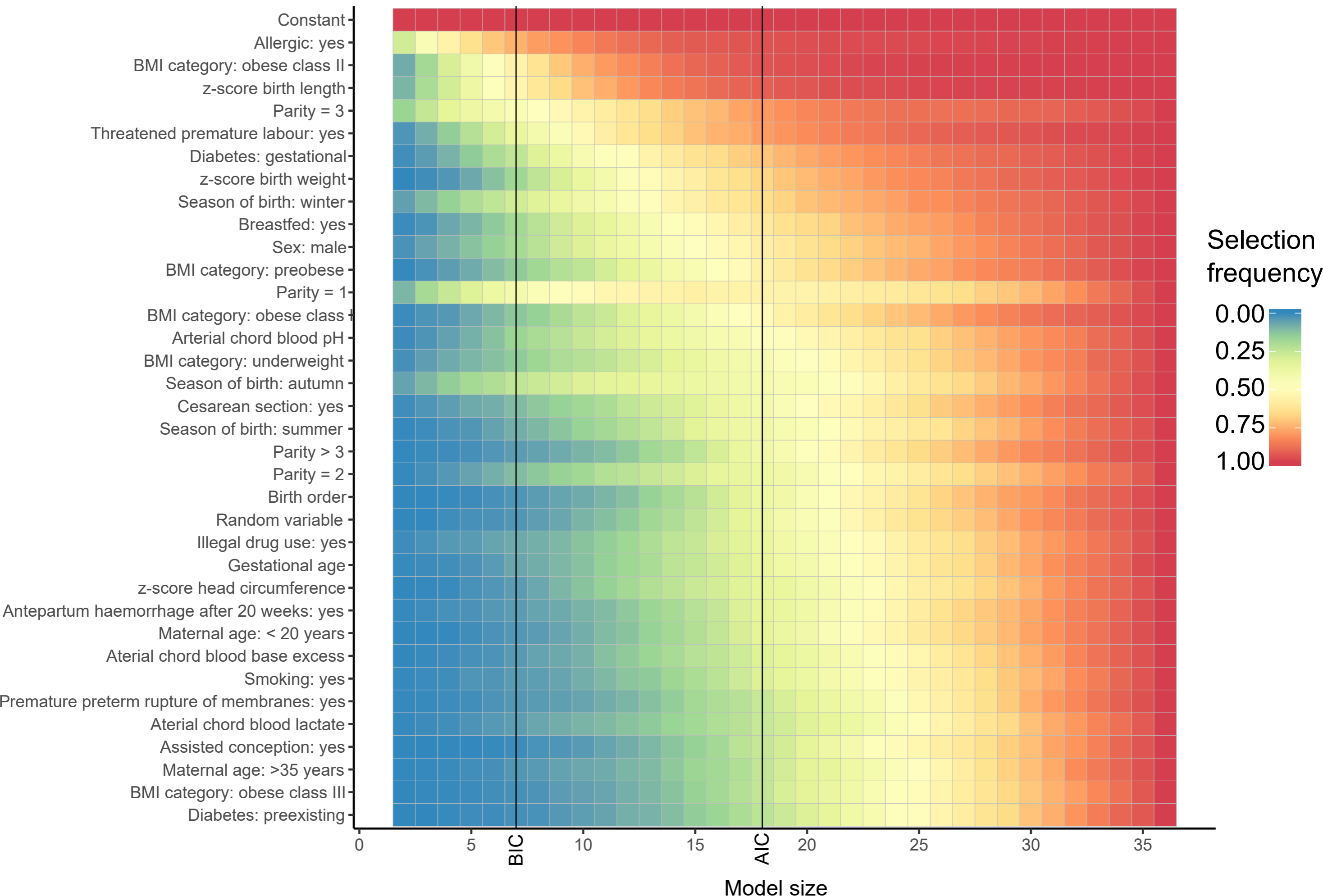

Supplement: S8 Fig — Included in the plot is the AIC and BIC threshold, which both present penalized-likelihood criteria which can be used to choose the best predictor subset. AIC, Akaike information criterion; BIC, Bayesian information criterion. (PDF) [file pone.0252215.s008.pdf]

# Variable inclusion tile plot

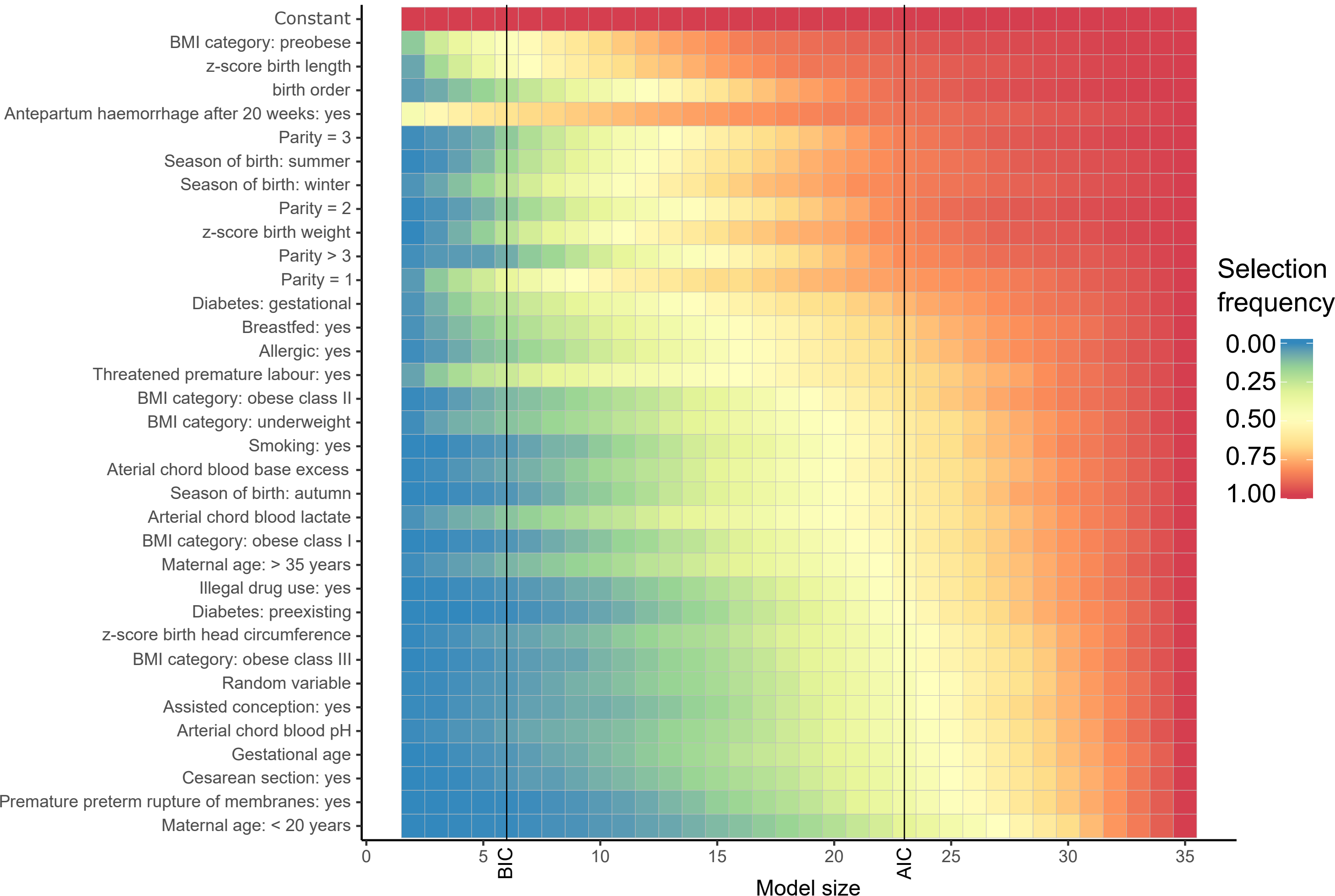

Supplement: S9 Fig — Included in the plot is the AIC and BIC threshold, which both present penalized-likelihood criteria which can be used to choose the best predictor subset. AIC, Akaike information criterion; BIC, Bayesian information criterion. (PDF) [file pone.0252215.s009.pdf]

Variable inclusion tile plot

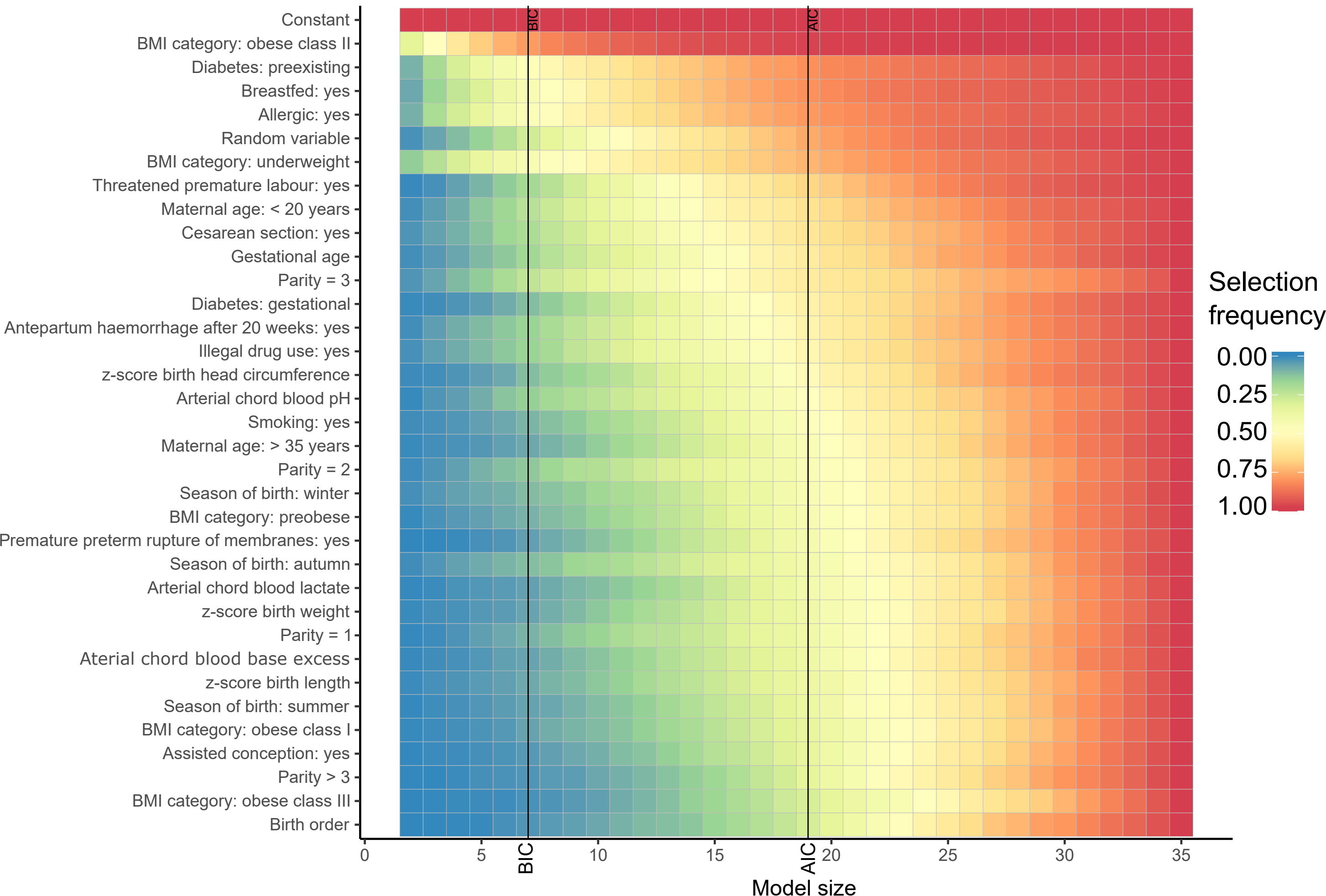

Supplement: S10 Fig — Included in the plot is the AIC and BIC threshold, which both present penalized-likelihood criteria which can be used to choose the best predictor subset. AIC, Akaike information criterion; BIC, Bayesian information criterion. (PDF) [file pone.0252215.s010.pdf]
