# Supplementary material for: Modelling maternal and perinatal risk factors to predict poorly controlled childhood asthma
Source: PLoS One. 2021 May 27;16(5):e0252215. doi: 10.1371/journal.pone.0252215 (PMC8158992; doi:10.1371/journal.pone.0252215)
Supplement: S1 Data — (PDF) [file pone.0252215.s011.pdf]

allergiesCate	maxAsthmaA	aphAfter20W	aterialChordE	aterialChordE	aterialChordE	bmiCategory
Yes	0	No	-0.1	3.9	7.33	NormalWeight
No	0	No	1.6	4.1	7.26	NormalWeight
Yes	0	No	-0.8	4.2	7.287	ObeseClass2
Yes	0	No	2.1	2.7	7.379	NormalWeight
No	0	No	-6.5	8.1	7.29	ObeseClass1
No	0	No	-4.2	4	7.283	NormalWeight
Yes	0	No	9.8	6.5	7.329	UnderWeight
Yes	0	No	1.1	3	7.24	UnderWeight
No	0	No	-0.5	0	7.251	ObeseClass1
Yes	0	No	-2.8	3.2	7.381	NormalWeight
No	0	No	-3.3	2.3	7.309	PreObese
No	0	No	-3.3	4.5	7.34	UnderWeight
No	0	No	-0.9	3	7.36	NormalWeight
No	0	No	-5.7	5.2	7.13	NormalWeight
No	0	No	-2.1	0	7.255	PreObese
No	0	No	-2.2	2.8	7.259	PreObese
No	0	No	-1.1	2	7.304	ObeseClass1
No	0	No	-2.1	4.6	7.3	NormalWeight
No	0	No	1.2	2.8	7.252	NormalWeight
No	0	No	-2.2	3.1	7.279	UnderWeight
No	0	No	-2.7	4	7.18	ObeseClass1
No	0	No	-1.7	2.8	7.294	PreObese
No	0	No	-1.7	3.3	7.316	NormalWeight
Yes	0	No	-4	3.3	7.24	ObeseClass3
No	0	No	-2	4.4	7.25	PreObese
Yes	0	No	2.3	2.4	7.427	ObeseClass1
Yes	0	No	-1.6	4.2	7.286	ObeseClass1
No	0	No	1.5	5.7	7.163	ObeseClass3
Yes	0	No	-5.1	5.2	7.234	ObeseClass2
No	0	No	-5.4	3.9	7.32	ObeseClass2
No	0	No	5	3	7.192	NormalWeight
No	0	No	1.9	5.8	7.17	ObeseClass3
No	0	No	1.6	3	7.29	UnderWeight
No	0	No	-3	2.9	7.35	ObeseClass3
Yes	0	No	-1.5	3.5	7.263	ObeseClass1
Yes	0	No	-4.3	5.5	7.226	NormalWeight
No	0	No	0	4.8	7.288	UnderWeight
No	0	No	-13	11.2	7.06	PreObese
No	0	No	-0.3	2.5	7.321	NormalWeight
No	0	No	-6.3	3.9	7.294	NormalWeight
Yes	0	No	-2.6	0	7.3	PreObese
No	0	No	-5.4	3	7.4	PreObese
No	0	No	-1.6	2.1	7.29	PreObese
No	0	No	2	3.4	7.273	ObeseClass1
No	0	Other	-2.4	3.3	7.316	PreObese
No	0	No	-1.3	5.1	7.298	NormalWeight
No	0	No	-8.8	8.2	7.047	PreObese

No	0	Other	-2	3.5	7.151	PreObese
No	0	No	-4.7	4.9	7.399	UnderWeight
No	0	No	0.1	3.2	7.25	PreObese
No	0	No	2	5.2	7.24	NormalWeight
No	0	No	-3.3	5.1	7.248	PreObese
Yes	0	No	0.3	3.3	7.3	UnderWeight
No	0	No	-5.4	5.5	7.35	NormalWeight
No	0	No	-3.7	4.6	7.242	PreObese
Yes	0	No	-5.4	5.1	7.28	NormalWeight
No	0	No	-3.6	3.7	7.37	ObeseClass2
No	0	No	-3.2	3.1	7.328	NormalWeight
Yes	0	No	-3.6	3.3	7.31	NormalWeight
Yes	0	No	2.8	3.4	7.247	NormalWeight
No	0	No	-3.9	4.3	7.292	UnderWeight
Yes	0	No	-2.8	3.5	7.3	ObeseClass1
No	0	Other	-7.4	5.4	7.22	NormalWeight
No	0	No	-4.3	4.9	7.26	ObeseClass1
No	0	No	-2.4	3.5	7.251	ObeseClass3
Yes	0	No	0.9	0	7.323	NormalWeight
No	0	No	-2.2	3.6	7.26	NormalWeight
No	0	No	-2	2.4	7.289	NormalWeight
Yes	0	No	-2.5	4.2	7.236	PreObese
No	0	No	-1	2.7	7.283	ObeseClass3
No	0	No	1	2.7	7.3	NormalWeight
No	0	No	-2	3.1	7.34	ObeseClass2
No	0	No	-3.1	3.8	7.246	PreObese
No	0	No	-5.4	4.9	7.286	ObeseClass2
Yes	0	No	3.8	3.4	7.4	UnderWeight
No	0	No	2.5	3.1	7.325	PreObese
No	0	No	1.4	3.2	7.346	ObeseClass3
No	0	No	2.4	0.9	7.322	NormalWeight
No	0	No	1.3	3.3	7.28	ObeseClass1
No	0	No	-2.7	3.1	7.361	NormalWeight
No	0	No	-1.7	3.5	7.317	PreObese
No	0	No	-2.8	3.9	7.27	NormalWeight
No	0	No	-6	6.8	7.178	NormalWeight
No	0	Other	-1.9	2.8	7.294	PreObese
No	0	No	-0.8	1.9	7.321	ObeseClass2
No	0	No	-4	5.5	7.18	NormalWeight
Yes	0	No	-1.2	3.9	7.205	NormalWeight
No	0	No	-2.5	2.5	7.308	PreObese
No	0	No	0.8	3	7.277	NormalWeight
No	0	No	0.3	4	7.286	NormalWeight
No	0	No	-0.9	3.7	7.21	PreObese
No	0	No	-16.1	9.7	7.05	ObeseClass1
No	0	No	-2.2	4.5	7.24	NormalWeight
Yes	0	No	-4.1	5	7.278	PreObese
No	0	No	1.7	4.3	7.34	ObeseClass2

No	0	No	2.8	2.3	7.28 PreObese
No	0	No	-2.6	4.5	7.251 NormalWeight
No	0	No	-5	5.9	7.16 UnderWeight
Yes	0	No	-2.1	2.4	7.3 PreObese
Yes	0	No	-4.8	0	7.278 PreObese
No	0	No	-5.8	7.3	7.156 ObeseClass1
Yes	0	No	0.1	3.4	7.295 PreObese
No	0	No	-4.7	4.5	7.43 ObeseClass1
No	0	No	-3.3	3.4	7.309 PreObese
No	0	No	-3.8	4.7	7.308 NormalWeight
No	0	No	-6	6.4	7.205 ObeseClass1
No	0	No	6	6	7.211 UnderWeight
No	0	No	0	3.7	7.232 ObeseClass1
Yes	0	No	6	4.4	7.42 PreObese
No	0	No	-0.8	4.5	7.18 NormalWeight
Yes	0	No	-2.8	3.2	7.232 ObeseClass2
No	0	No	-2.6	3.7	7.28 PreObese
No	0	No	1.2	2.2	7.29 ObeseClass1
No	0	No	2.9	4.6	7.19 ObeseClass1
Yes	0	No	0.7	2.6	7.309 NormalWeight
Yes	0	No	2.4	0.1	7.295 ObeseClass2
No	0	No	-3.7	2.5	7.394 PreObese
No	0	No	-3.1	5.7	7.21 NormalWeight
No	0	No	-0.7	5.3	7.208 NormalWeight
No	0	No	-3.4	3.1	7.33 UnderWeight
Yes	0	No	0.1	2.7	7.308 NormalWeight
Yes	0	No	7.2	7.5	7.135 UnderWeight
No	0	No	1.1	2.5	7.379 NormalWeight
Yes	0	No	-0.5	2	7.37 NormalWeight
Yes	0	No	-6	3.2	7.35 UnderWeight
No	0	No	5.1	5.1	7.23 PreObese
No	0	No	-3.2	3.3	7.352 NormalWeight
No	0	No	2.3	4	7.302 NormalWeight
No	0	No	-0.8	3.8	7.272 ObeseClass2
No	0	No	-0.6	3.6	7.248 NormalWeight
No	0	No	-5.4	4.9	7.215 PreObese
No	0	No	-5.2	2.9	7.26 UnderWeight
No	0	No	-6.2	4.1	7.259 PreObese
No	0	No	2.8	2.4	7.333 NormalWeight
No	0	No	-3.6	4.9	7.304 UnderWeight
No	0	No	3.5	2.7	7.46 PreObese
No	0	No	2.8	4.5	7.275 NormalWeight
No	0	No	-2.3	3	7.269 NormalWeight
No	0	No	3.7	3.7	7.214 UnderWeight
No	0	No	-0.4	3	7.38 ObeseClass1
Yes	0	No	-3.2	5.4	7.245 ObeseClass1
No	0	No	-1	3	7.268 PreObese
No	0	No	-2.6	3.7	7.32 ObeseClass1

No	0	No	4.1	3.6	7.4	NormalWeight
No	0	No	-1.7	3.9	7.26	NormalWeight
No	0	No	-3.1	1.8	7.298	PreObese
Yes	0	No	3.6	4.8	7.22	UnderWeight
No	0	No	0.1	2	7.38	NormalWeight
No	0	No	3.6	4.1	7.2	UnderWeight
No	0	No	-1.5	2.3	7.418	ObeseClass3
Yes	0	No	5.9	7.9	7.16	NormalWeight
No	0	No	-2.6	3.1	7.307	UnderWeight
No	0	No	-2.9	3.2	7.38	ObeseClass1
No	0	No	0.5	3	7.26	NormalWeight
No	0	No	0.8	3.2	7.32	PreObese
No	0	No	0.2	2.5	7.29	ObeseClass2
No	0	No	-7.5	7	7.287	NormalWeight
Yes	0	No	-1.1	4.4	7.27	ObeseClass1
No	0	No	-3	4.1	7.32	ObeseClass1
Yes	0	No	-1.6	3.1	7.4	PreObese
No	0	No	-1.1	3	7.222	ObeseClass2
No	0	No	5	4.3	7.31	NormalWeight
No	0	No	-0.4	2.9	7.303	ObeseClass2
No	0	No	0.2	3.2	7.289	ObeseClass2
Yes	0	No	-0.7	2.1	7.408	NormalWeight
Yes	0	No	-7.8	5.8	7.205	PreObese
Yes	0	No	3.2	3.9	7.34	NormalWeight
Yes	0	No	4.1	3.3	7.35	NormalWeight
No	0	No	-1	3	7.271	ObeseClass2
Yes	0	No	4.3	4.6	7.25	UnderWeight
No	0	No	-6.1	8.5	7.16	ObeseClass1
No	0	No	-3.2	1.7	7.38	PreObese
No	0	No	-1.8	2.6	7.312	NormalWeight
No	0	No	0.2	2.7	7.305	NormalWeight
No	0	No	-1.6	2.1	7.308	NormalWeight
No	0	No	-1	3.9	7.216	NormalWeight
No	0	No	-3.7	2.7	7.348	UnderWeight
No	0	No	-4.1	5.2	7.233	PreObese
No	0	No	-6	6.7	7.269	UnderWeight
No	0	No	1.3	4.8	7.3	ObeseClass1
No	0	No	-5.8	5.9	7.232	ObeseClass1
No	0	No	-4.3	2.2	7.288	PreObese
No	0	No	2.1	4.5	7.33	NormalWeight
No	0	Other	1.1	2.5	7.335	NormalWeight
No	0	No	-3.1	3.5	7.27	NormalWeight
No	0	No	-5.1	5.2	7.212	UnderWeight
No	0	No	-0.3	2.9	7.273	UnderWeight
No	0	No	4.3	3.1	7.263	NormalWeight
No	0	No	0.1	2.4	7.34	UnderWeight
Yes	0	No	-7.6	5.5	7.184	NormalWeight
No	0	Other	-2.4	5	7.29	ObeseClass1

No	0	No	-7	6.3	7.227	NormalWeight
No	0	No	-2	6.1	7.176	NormalWeight
No	0	No	1	5.3	7.22	NormalWeight
No	0	No	-1	2.7	7.33	ObeseClass1
No	0	No	-5.4	6.4	7.195	ObeseClass1
No	0	No	-6.2	6	7.175	ObeseClass1
No	0	No	-0.6	3.6	7.31	PreObese
No	0	No	3.6	4	7.248	NormalWeight
No	0	No	-1.6	3.5	7.232	ObeseClass1
No	0	No	0	2.7	7.26	NormalWeight
No	0	No	3.5	3.2	7.3	ObeseClass1
Yes	0	No	2.2	4	7.21	NormalWeight
No	0	No	1.9	4.1	7.22	NormalWeight
Yes	0	No	-5.1	4.4	7.33	NormalWeight
No	0	No	-5.8	5.8	7.258	NormalWeight
Yes	0	No	1.7	2.8	7.274	PreObese
Yes	0	No	-3.4	3.2	7.344	NormalWeight
No	0	No	-0.6	2.4	7.244	ObeseClass2
No	0	No	-4.5	4.1	7.241	NormalWeight
No	0	No	-2.9	2.5	7.333	ObeseClass1
Yes	0	No	2.9	1.6	7.32	NormalWeight
No	0	No	1.1	4	7.239	ObeseClass3
No	0	No	0.2	3.1	7.247	ObeseClass3
Yes	0	No	-2.6	4.4	7.376	PreObese
Yes	0	No	-0.3	1.6	7.325	ObeseClass1
No	0	No	7.3	4.3	7.24	ObeseClass2
No	0	No	0.3	2.9	7.39	NormalWeight
No	0	No	0	2.2	7.32	ObeseClass1
No	0	No	0.2	4.4	7.24	ObeseClass1
No	0	No	-3.4	3.9	7.27	NormalWeight
Yes	0	No	2.3	3.9	7.252	NormalWeight
No	0	No	-2.7	3.1	7.333	NormalWeight
Yes	0	No	0.7	3.1	7.3	UnderWeight
No	0	No	-2.5	2.9	7.251	ObeseClass2
No	0	No	0.2	3.4	7.324	NormalWeight
No	0	No	-9.3	1.7	7.26	NormalWeight
No	0	No	-4	3.8	7.1	NormalWeight
No	0	No	-1.6	2.4	7.36	NormalWeight
No	0	No	-2.2	2.4	7.251	UnderWeight
No	0	No	-1.4	2.4	7.337	ObeseClass2
No	0	No	-3.2	4.3	7.26	UnderWeight
No	0	No	-2.6	3.2	7.207	NormalWeight
No	0	No	-7.6	6.6	7.25	PreObese
No	0	No	5.2	3.8	7.245	ObeseClass2
No	0	No	-7.1	8	7.195	NormalWeight
Yes	0	No	0.1	1.9	7.32	ObeseClass1
No	0	No	-4.5	2.8	7.406	UnderWeight
No	0	No	1	1.9	7.36	NormalWeight

No	0	No	4.1	5.5	7.202	NormalWeight
No	0	No	0.3	3.5	7.32	PreObese
No	0	No	-4.1	7.2	7.183	ObeseClass1
No	0	No	-1.1	3	7.25	PreObese
No	0	No	7.3	6.7	7.168	ObeseClass1
No	0	No	-8.4	0.8	7.43	ObeseClass1
No	0	No	-1	3.4	7.257	NormalWeight
Yes	0	No	-3.8	3.9	7.355	NormalWeight
No	0	No	-1.6	3.5	7.35	UnderWeight
No	0	No	2.1	3	7.3	PreObese
No	0	No	-3.2	4.6	7.194	NormalWeight
No	0	No	-7.4	6.7	7.135	ObeseClass1
No	0	No	1.1	4.4	7.219	PreObese
No	0	No	-6.7	9.4	7.099	UnderWeight
No	0	No	-0.6	3.2	7.231	NormalWeight
No	0	No	-4.8	5.2	7.16	ObeseClass3
No	0	No	0.6	2.2	7.303	ObeseClass1
No	0	No	-0.7	4.2	7.203	NormalWeight
No	0	No	6.6	5.4	7.165	NormalWeight
No	0	No	2.8	3.1	7.2	UnderWeight
No	0	No	-2.2	4.4	7.32	NormalWeight
No	0	No	-0.8	2.8	7.37	NormalWeight
No	0	No	3.1	4.3	7.5	PreObese
No	0	No	0.1	3.6	7.25	PreObese
No	0	No	1.8	3.9	7.316	NormalWeight
No	0	No	-9.5	4.1	7.246	UnderWeight
Yes	0	No	1.1	2.7	7.37	ObeseClass2
No	0	No	-5.3	5.7	7.293	NormalWeight
Yes	0	No	0.9	2.2	7.295	ObeseClass1
Yes	0	No	1.2	3.2	7.34	NormalWeight
No	0	No	7.1	2.8	7.32	PreObese
No	0	No	-0.4	1.7	7.317	UnderWeight
No	0	No	-0.8	4.2	7.26	ObeseClass1
Yes	0	No	-8.7	7.8	7.115	PreObese
No	0	No	5.9	6.1	7.33	NormalWeight
No	0	No	-1.6	4.4	7.174	ObeseClass2
No	0	No	-1.2	0	7.32	ObeseClass1
Yes	0	No	3.8	0.8	7.48	UnderWeight
No	0	No	-0.7	2.7	7.289	NormalWeight
No	0	No	-0.4	2.1	7.282	PreObese
No	0	No	0.5	2.7	7.353	PreObese
No	0	No	-2.6	4.3	7.22	ObeseClass2
Yes	0	No	-8.9	7.9	7.137	UnderWeight
No	0	No	-8.7	7.3	7.178	ObeseClass3
No	0	No	-4.5	5	7.255	PreObese
No	0	No	-2.5	2.2	7.327	NormalWeight
Yes	0	No	-0.9	2.9	7.283	PreObese
No	0	No	-3.1	5.4	7.31	NormalWeight

Yes	0	No	-6.2	5.1	7.195	PreObese
No	0	No	-1.8	3.6	7.24	PreObese
No	0	No	1.7	4.5	7.24	NormalWeight
No	0	No	-5.8	5.1	7.279	UnderWeight
No	0	No	0.1	2.7	7.284	PreObese
Yes	0	No	-1	2.4	7.284	UnderWeight
Yes	0	No	-1.6	3	7.343	NormalWeight
Yes	0	No	-0.5	2.4	7.342	PreObese
No	0	No	3.5	3.5	7.26	NormalWeight
No	0	No	-4.9	3.3	7.333	ObeseClass1
No	0	No	-2.3	0.8	7.307	ObeseClass2
Yes	0	No	-1.2	2.5	7.323	NormalWeight
Yes	0	No	4.4	4.5	7.291	PreObese
No	0	No	-5.2	6.8	7.226	NormalWeight
No	0	Other	-6.3	1.6	7.272	ObeseClass1
Yes	0	No	1.9	3.2	7.29	NormalWeight
No	0	No	-1.4	4	7.338	UnderWeight
No	0	No	-0.5	3.5	7.24	NormalWeight
Yes	0	No	-1.3	3.1	7.249	ObeseClass1
No	0	No	-7	7.6	7.12	PreObese
Yes	0	No	-3	3.4	7.182	NormalWeight
Yes	0	No	1.7	0.3	7.45	ObeseClass3
Yes	0	No	8.4	9.5	6.98	NormalWeight
No	0	No	-3.8	5	7.28	NormalWeight
Yes	0	No	-5.5	5	7.19	ObeseClass1
No	0	No	-1	3.6	7.34	PreObese
No	0	No	0.2	2.3	7.273	NormalWeight
Yes	0	No	-1.1	3.7	7.299	ObeseClass1
No	0	No	-5.7	5.3	7.3	ObeseClass3
No	0	No	-4	2	7.22	PreObese
No	0	No	-2.3	2.7	7.369	UnderWeight
Yes	0	No	0.2	2	7.358	NormalWeight
No	0	Other	-6.3	4.6	7.26	NormalWeight
No	0	No	1.5	5.1	7.235	PreObese
No	0	No	0.7	2.4	7.244	NormalWeight
No	0	No	0.6	1.9	7.42	NormalWeight
No	0	No	-2.1	2.6	7.301	ObeseClass1
No	0	No	-6.4	4.4	7.467	NormalWeight
No	0	No	1.6	1.9	7.32	NormalWeight
No	0	No	-0.9	3.5	7.27	PreObese
No	0	No	-1.6	1.9	7.279	ObeseClass2
No	0	No	-0.9	3.4	7.298	NormalWeight
No	0	No	1.5	4	7.29	NormalWeight
No	0	No	0.9	2.6	7.288	PreObese
No	0	No	-3.9	5.6	7.179	NormalWeight
No	0	No	11.7	1.7	7.47	ObeseClass1
Yes	0	Other	0.5	2.1	7.327	NormalWeight
No	0	No	-0.9	4.5	7.25	PreObese

No	0	No	0.7	2.1	7.315	NormalWeight
No	0	No	3.9	4	7.229	PreObese
No	0	No	-5	4.6	7.175	NormalWeight
No	0	No	-2.6	3.3	7.248	NormalWeight
No	0	No	2.6	1.7	7.327	PreObese
No	0	No	-8.5	6.5	7.16	NormalWeight
Yes	0	No	-1.5	2.7	7.328	NormalWeight
No	0	No	1.6	3	7.315	NormalWeight
No	0	No	-6.5	4.6	7.278	ObeseClass1
No	0	No	0.2	3.2	7.271	PreObese
No	0	No	-2.3	2	7.359	PreObese
Yes	0	No	-7.5	6.5	7.235	PreObese
Yes	0	No	2	2.7	7.26	ObeseClass1
No	0	No	-8	7.5	7.204	NormalWeight
No	0	No	3.3	6	7.218	UnderWeight
No	0	No	0.6	2.6	7.276	ObeseClass3
No	0	No	-1.5	2.9	7.217	PreObese
No	0	No	-1.7	5.2	7.23	PreObese
No	0	No	-7	4.4	7.19	NormalWeight
No	0	No	0.3	2.1	7.33	NormalWeight
No	0	No	-3.7	3	7.302	PreObese
Yes	0	No	-5.8	5.4	7.22	PreObese
No	0	No	-4.3	3.2	7.277	UnderWeight
Yes	0	No	1.2	2.4	7.264	PreObese
No	0	No	-2.6	1.8	7.297	NormalWeight
No	0	No	-1.6	4.1	7.26	PreObese
No	0	No	-3.4	0.9	7.263	ObeseClass1
No	0	No	0.4	3.4	7.26	ObeseClass1
No	0	No	-0.8	2.8	7.26	ObeseClass2
No	0	No	-1.9	4.8	7.244	NormalWeight
Yes	0	No	-5.4	5.1	7.276	NormalWeight
No	0	No	1.4	3.8	7.27	ObeseClass1
No	0	No	-2	3.8	7.24	NormalWeight
No	0	No	-1.3	5.1	7.28	NormalWeight
No	0	No	-1.2	2.4	7.35	NormalWeight
No	0	No	2.4	3.4	7.362	ObeseClass2
No	0	No	5.5	7.6	7.14	NormalWeight
No	0	No	-7	9	7.153	PreObese
No	0	No	-4.2	5.3	7.309	ObeseClass1
No	0	No	1.7	2.5	7.36	ObeseClass1
Yes	0	No	-1.2	4	7.289	NormalWeight
No	0	No	-3.1	4.6	7.256	ObeseClass1
Yes	0	No	2.4	3.9	7.23	ObeseClass3
No	0	Other	-0.7	3.4	7.295	ObeseClass1
No	0	No	-1.8	2.4	7.33	ObeseClass1
No	0	No	7.7	5.9	7.353	UnderWeight
No	0	No	-0.6	3.9	7.285	PreObese
No	0	No	-0.5	2.1	7.41	UnderWeight

No	0	No	-3.2	3	7.44	NormalWeight
No	0	No	1.2	6.5	7.23	ObeseClass1
No	0	No	1.6	1.3	7.3	NormalWeight
No	0	No	-0.7	3.3	7.286	UnderWeight
No	0	No	-6.3	7.7	7.15	NormalWeight
No	0	No	-9.9	8.6	7.05	ObeseClass1
Yes	0	No	-6.5	7.1	7.277	NormalWeight
Yes	0	No	5.4	5.2	7.47	ObeseClass2
Yes	0	No	-5.9	8.6	7.016	PreObese
No	0	No	-4.4	4.2	7.15	ObeseClass1
Yes	0	No	-4.8	5.9	7.22	PreObese
No	0	No	6.3	5.7	7.19	PreObese
Yes	0	No	-3.8	2.9	7.346	NormalWeight
No	0	Other	-0.5	3.4	7.283	PreObese
Yes	0	No	3	2.2	7.31	UnderWeight
Yes	0	No	-2.1	4.5	7.249	UnderWeight
No	0	No	-10.7	6.3	7.242	NormalWeight
No	0	No	-8	4.3	7.228	NormalWeight
No	0	No	7.4	3.6	7.356	PreObese
No	0	No	-0.3	2.1	7.324	ObeseClass2
Yes	0	No	1.7	4.3	7.37	NormalWeight
No	0	No	3	2.8	7.29	NormalWeight
No	0	No	0.6	4.4	7.39	NormalWeight
No	0	No	0.2	2.6	7.327	PreObese
No	0	No	-6.2	3.1	7.3	ObeseClass3
No	0	No	-2.9	2.3	7.37	PreObese
No	0	No	-8	10.3	7.08	NormalWeight
No	0	No	1	2	7.277	ObeseClass1
No	0	No	0.2	4.9	7.26	PreObese
No	0	No	2.1	5.3	7.19	NormalWeight
Yes	0	No	0.4	2.4	7.372	PreObese
No	0	No	-1.1	4.1	7.261	NormalWeight
No	0	Other	-1.8	4.8	7.239	PreObese
No	0	No	-4.6	3	7.362	NormalWeight
No	0	No	-1.4	3.7	7.32	UnderWeight
No	0	No	-1.6	4.2	7.29	PreObese
No	0	No	6	4.2	7.24	NormalWeight
No	0	No	5.3	4	7.44	ObeseClass2
No	0	No	-1.1	4.3	7.274	NormalWeight
Yes	0	No	-3.5	3.2	7.239	UnderWeight
No	0	No	-6.6	6.3	7.25	PreObese
Yes	0	No	5.8	5.1	7.302	PreObese
No	0	No	-3.2	4.6	7.16	NormalWeight
No	0	No	-1.5	2.8	7.291	NormalWeight
No	0	No	-2.1	2.3	7.386	NormalWeight
No	0	No	-4.5	6.4	7.166	ObeseClass1
No	0	No	-1.1	2.9	7.298	NormalWeight
No	0	No	4.8	4.6	7.221	ObeseClass1

No	0	No	-0.8	3.2	7.348	ObeseClass1
No	0	No	0.1	2.3	7.236	NormalWeight
No	0	No	-1.9	3.8	7.232	ObeseClass1
No	0	No	5.3	3.8	7.37	NormalWeight
Yes	0	No	-0.3	2.1	7.308	PreObese
No	0	No	3.6	1.4	7.379	ObeseClass2
Yes	0	No	-4	5	7.186	NormalWeight
No	0	No	-1.4	3.2	7.33	UnderWeight
Yes	0	No	1.3	3.4	7.357	PreObese
Yes	0	No	4.1	6	7.22	PreObese
No	0	No	3.4	5.1	7.241	PreObese
No	0	No	-2	7.2	7.2	NormalWeight
No	0	No	-5.6	5.5	7.236	NormalWeight
No	0	No	-2.2	4.7	7.274	PreObese
No	0	No	-3.5	2.5	7.316	NormalWeight
Yes	0	No	0.6	2.9	7.318	PreObese
No	0	No	-3	5.2	7.191	ObeseClass1
Yes	0	No	-0.8	4.4	7.189	ObeseClass1
No	0	No	-8.8	7.6	7.158	PreObese
No	0	No	-1.2	4	7.235	PreObese
No	0	No	1.8	3.8	7.38	NormalWeight
Yes	0	Other	-3.2	4.2	7.287	ObeseClass2
No	0	No	0.7	2.4	7.258	ObeseClass3
Yes	0	No	1.6	1.9	7.4	ObeseClass2
No	0	No	-2.9	2.7	7.255	UnderWeight
No	0	No	-4.4	5.6	7.23	NormalWeight
No	0	No	-5.5	7.6	7.121	UnderWeight
No	0	No	-1.4	2.7	7.321	NormalWeight
Yes	0	No	-7.5	4.8	7.237	PreObese
No	0	No	3.7	8.6	7.07	NormalWeight
No	0	No	-6	5.5	7.317	NormalWeight
Yes	0	No	1.3	4	7.3	UnderWeight
No	0	No	-1	2.5	7.228	ObeseClass2
No	0	No	-0.2	2.8	7.416	NormalWeight
No	0	No	-1.5	4.4	7.21	UnderWeight
Yes	0	No	3.1	4.7	7.27	PreObese
Yes	0	No	-4.8	8.6	7.112	PreObese
No	0	No	-6.1	0.8	7.461	NormalWeight
No	0	No	1.7	4.7	7.197	ObeseClass3
Yes	0	No	-5.7	3.4	7.284	UnderWeight
No	0	No	-7.8	4.7	7.183	UnderWeight
No	0	No	-5.5	6.1	7.224	NormalWeight
No	0	No	8.6	6.8	7.137	PreObese
No	0	No	0	3.1	7.258	NormalWeight
No	0	No	0.4	3.5	7.237	NormalWeight
Yes	0	No	0.3	2.5	7.28	ObeseClass1
No	0	No	-5.3	6.3	7.211	NormalWeight
No	0	No	-4.4	2.2	7.475	NormalWeight

No	0	No	-7.8	6.8	7.3	NormalWeight
No	0	No	5.6	6.2	7.28	NormalWeight
Yes	0	No	0.1	3	7.296	NormalWeight
No	0	No	-3.2	4.7	7.274	ObeseClass1
No	0	No	0.5	2.4	7.269	ObeseClass2
Yes	0	No	2.2	1.5	7.316	NormalWeight
No	0	No	-1.7	4.8	7.29	PreObese
No	0	No	0.2	2.4	7.316	PreObese
No	0	No	-2	3.5	7.293	ObeseClass1
No	0	No	-2.1	2.3	7.314	NormalWeight
No	0	No	0.1	2.9	7.301	PreObese
No	0	No	-7.2	5.5	7.215	NormalWeight
Yes	0	No	1.5	3.4	7.32	UnderWeight
No	0	No	2.3	4.1	7.357	ObeseClass1
No	0	No	-0.1	2.9	7.27	PreObese
Yes	0	No	-1.2	3.3	7.49	PreObese
No	0	No	-6.3	5.3	7.272	NormalWeight
Yes	0	No	3.3	3.1	7.293	NormalWeight
No	0	No	-5	4.5	7.25	NormalWeight
No	0	No	-3.9	3.3	7.285	UnderWeight
No	0	No	-2.9	2.9	7.299	PreObese
No	0	No	-4.1	4	7.32	NormalWeight
Yes	0	No	-4.7	4.5	7.31	NormalWeight
No	0	No	0.2	3.3	7.288	NormalWeight
No	0	No	4.5	5.1	7.22	NormalWeight
No	0	No	-0.9	2	7.287	UnderWeight
No	0	No	-2.7	4.5	7.25	PreObese
No	0	No	-1.2	4.3	7.276	NormalWeight
No	0	No	-2.3	2.4	7.29	PreObese
Yes	0	No	-1.6	3.5	7.259	ObeseClass3
No	0	No	-2.6	2.4	7.375	PreObese
No	0	No	-2.6	3.1	7.262	NormalWeight
Yes	0	No	0.9	2.7	7.285	ObeseClass2
No	0	No	1.1	2.9	7.346	PreObese
No	0	No	0.6	3.5	7.27	UnderWeight
No	0	No	0.5	2.1	7.34	ObeseClass3
Yes	0	No	-0.8	4.7	7.27	NormalWeight
No	0	No	0.6	2.5	7.366	NormalWeight
No	0	No	-4.2	8.9	7.24	NormalWeight
No	0	No	4.9	7.1	7.13	ObeseClass3
No	0	No	3	3.4	7.25	ObeseClass1
No	0	No	-1	5.2	7.219	ObeseClass1
No	0	No	-4.9	6.2	7.295	NormalWeight
No	0	No	0.2	3.5	7.22	ObeseClass3
No	0	No	-0.9	2.5	7.334	NormalWeight
No	0	No	-2.7	3.2	7.202	NormalWeight
No	0	No	7.2	6.1	7.37	PreObese
Yes	0	No	1.4	2.9	7.323	NormalWeight

No	0	No	-5.4	4.2	7.31	NormalWeight
No	0	No	-2.2	2.9	7.279	UnderWeight
Yes	0	No	-4.9	4.8	7.254	ObeseClass3
No	0	No	2.3	3.4	7.268	NormalWeight
No	0	No	5.9	3.4	7.39	NormalWeight
Yes	0	Other	-6	8.7	7.111	ObeseClass1
No	0	No	-2	2.3	7.323	ObeseClass1
No	0	No	-3.4	3.1	7.238	UnderWeight
No	0	No	1.7	3.2	7.253	PreObese
Yes	0	No	-1.2	2.3	7.343	NormalWeight
Yes	0	No	1.3	4.8	7.32	NormalWeight
Yes	0	No	-2.8	3.5	7.225	PreObese
No	0	No	-3	5.7	7.244	NormalWeight
No	0	No	1.8	5.3	7.229	ObeseClass3
No	0	No	-1.8	4.2	7.244	ObeseClass1
No	0	No	1.3	4.5	7.29	PreObese
No	0	No	-7.9	5.3	7.264	PreObese
Yes	0	No	-0.6	2.1	7.351	PreObese
No	0	No	-3	4.6	7.247	NormalWeight
Yes	0	No	1.3	3	7.275	ObeseClass1
Yes	0	No	-2.5	4.5	7.275	UnderWeight
No	0	No	-3.6	4	7.234	PreObese
No	0	No	1.7	3.8	7.273	NormalWeight
No	0	No	-2.9	4.5	7.218	ObeseClass1
Yes	0	No	-4	7.4	7.22	NormalWeight
No	0	No	-2.6	2.6	7.3	NormalWeight
Yes	0	No	1.2	3.7	7.3	ObeseClass2
No	0	No	-7.3	7.5	7.11	PreObese
No	0	No	-3.7	5.7	7.21	NormalWeight
No	0	No	-4.2	4.2	7.315	NormalWeight
No	0	No	-1.1	3.5	7.283	NormalWeight
Yes	0	No	1.4	2.2	7.2	NormalWeight
No	0	No	0.2	2.3	7.34	UnderWeight
No	0	No	-1.6	1.9	7.47	PreObese
No	0	No	-1.3	1.8	7.441	NormalWeight
No	0	No	2.1	5.3	7.14	NormalWeight
No	0	No	-3.4	0.7	7.288	PreObese
No	0	No	-3.8	5.3	7.255	NormalWeight
Yes	0	No	1.8	4.1	7.412	ObeseClass1
No	0	Other	-1.7	1.8	7.295	PreObese
No	0	No	-5.7	4.4	7.234	NormalWeight
No	0	No	-0.7	4.2	7.282	NormalWeight
Yes	0	No	-2.6	6	7.205	NormalWeight
Yes	0	No	-4	0.7	7.307	NormalWeight
No	0	No	-0.4	2.7	7.284	NormalWeight
No	0	No	2.4	3.5	7.336	NormalWeight
No	0	No	-9.8	7.7	7.076	NormalWeight
No	0	No	5.3	3.5	7.29	NormalWeight

Yes	0	No	4.6	5.7	7.16	ObeseClass1
No	0	No	-9.1	8.1	7.273	NormalWeight
Yes	0	No	-1.8	5.7	7.19	NormalWeight
Yes	0	No	0.6	3	7.258	PreObese
No	0	No	-3.2	4.8	7.11	PreObese
No	0	No	-4.3	2.5	7.394	NormalWeight
Yes	0	No	-1.5	3.2	7.3	NormalWeight
No	0	No	-5.4	3.4	7.278	NormalWeight
No	0	No	5.1	5.9	7.346	NormalWeight
No	0	No	6.5	7.9	7.22	ObeseClass3
No	0	No	-3.8	4.4	7.237	NormalWeight
No	0	No	0	2.8	7.296	PreObese
No	0	No	6.5	5.5	7.28	NormalWeight
No	0	No	0	5	7.28	NormalWeight
No	0	No	-0.8	1.4	7.5	UnderWeight
No	0	No	2.3	2.8	7.279	ObeseClass1
No	0	Other	-7	8.1	7.082	PreObese
No	0	No	3.5	4.2	7.36	ObeseClass1
No	0	No	-1.5	3.2	7.26	NormalWeight
No	0	No	9.9	4.2	7.341	PreObese
No	0	No	-11.7	8.5	7.09	PreObese
No	0	No	-10.7	6.3	7.2	NormalWeight
No	0	No	-0.4	2.8	7.3	PreObese
Yes	0	No	-0.2	4.7	7.235	PreObese
No	0	No	-2.3	2.7	7.295	PreObese
No	0	No	-3	6.3	7.15	UnderWeight
No	0	No	0.1	4	7.281	NormalWeight
No	0	No	-6.2	4.6	7.363	PreObese
No	0	No	1.7	3	7.357	ObeseClass3
No	0	No	0.2	3.9	7.295	ObeseClass1
Yes	0	No	-3.7	6.2	7.199	NormalWeight
No	0	No	1.3	2.7	7.235	ObeseClass1
No	0	No	-4.8	6.1	7.106	NormalWeight
No	0	No	-4.5	5	7.27	NormalWeight
No	0	No	-1.2	2.1	7.322	ObeseClass1
No	0	No	-4.7	3.9	7.254	ObeseClass1
No	0	No	-8.4	8.4	7.28	UnderWeight
No	0	No	-3.4	3.5	7.334	PreObese
No	0	No	-1.6	4.4	7.333	PreObese
No	0	No	-0.1	2.6	7.29	NormalWeight
No	0	No	1.4	3	7.23	NormalWeight
Yes	0	No	1.2	2.5	7.296	PreObese
No	0	No	-6.9	6.9	7.14	UnderWeight
No	0	No	-6.3	5.3	7.218	PreObese
No	0	No	-4.2	3.6	7.225	NormalWeight
Yes	0	No	0.5	4	7.28	UnderWeight
No	0	No	-0.8	4.5	7.26	ObeseClass2
No	0	No	-3	5.3	7.253	NormalWeight

No	0	No	-0.2	3.7	7.333 UnderWeight
Yes	0	No	-2.4	4.1	7.307 NormalWeight
No	0	No	1.3	2.7	7.313 ObeseClass2
No	0	No	0.3	3.6	7.33 NormalWeight
No	0	No	-5	5.4	7.2 NormalWeight
No	0	No	0.2	3.4	7.269 ObeseClass1
No	0	No	-8.4	8.2	7.172 NormalWeight
Yes	0	No	0.6	3.2	7.304 UnderWeight
No	0	No	0.9	2.4	7.333 ObeseClass2
No	0	No	-0.1	2.8	7.264 ObeseClass2
No	0	No	-3.5	3.8	7.28 PreObese
No	0	No	-5.8	5.6	7.291 PreObese
No	0	No	-3.5	3.8	7.31 UnderWeight
No	0	No	-1.1	2.5	7.32 UnderWeight
No	0	No	-2.7	3.3	7.35 NormalWeight
No	0	No	3.3	4.5	7.38 NormalWeight
Yes	0	No	8	7.8	7.2 PreObese
No	0	No	1.2	4.6	7.25 ObeseClass2
No	0	No	-7.2	4.2	7.22 PreObese
No	0	No	2.5	3.3	7.281 ObeseClass2
No	0	No	-0.9	3.3	7.322 ObeseClass1
Yes	0	No	-2.4	4.7	7.249 UnderWeight
No	0	No	-3.9	2.4	7.339 NormalWeight
No	0	No	-6	6.9	7.267 NormalWeight
Yes	0	No	2.2	5	7.243 ObeseClass1
No	0	No	4.4	5.1	7.21 UnderWeight
No	0	Other	-4.8	3.8	7.2 NormalWeight
No	0	No	0.5	2.6	7.216 ObeseClass3
No	0	No	-2.8	3.5	7.238 ObeseClass3
No	0	No	-4.5	4.5	7.165 ObeseClass1
Yes	0	No	-4.3	5.2	7.226 NormalWeight
Yes	0	No	-2.2	4.2	7.258 ObeseClass2
No	0	No	-5.1	3.5	7.3 ObeseClass1
No	0	No	1.3	4.3	7.26 NormalWeight
Yes	0	No	-3.4	3.4	7.237 PreObese
No	0	No	-1.3	3.1	7.214 ObeseClass1
No	0	No	-3.8	5.8	7.311 NormalWeight
Yes	0	No	2.8	2	7.38 UnderWeight
No	0	No	2.8	2.7	7.386 NormalWeight
Yes	0	No	-0.3	4.8	7.31 UnderWeight
No	0	No	-5.3	5.2	7.307 NormalWeight
No	0	No	-0.8	4.1	7.25 UnderWeight
No	0	No	4.8	4.3	7.25 ObeseClass2
No	0	No	7.9	6.6	7.09 PreObese
Yes	0	No	-3.7	3	7.33 NormalWeight
No	0	No	-2.3	2.6	7.309 NormalWeight
No	0	No	-1.7	4.9	7.23 NormalWeight
No	0	No	2	2.9	7.27 NormalWeight

No	0	No	-3.7	6.4	7.225	PreObese
Yes	0	No	0.7	3.2	7.24	UnderWeight
No	0	No	-2.3	2.4	7.4	UnderWeight
No	0	No	3.4	2.3	7.41	NormalWeight
No	0	No	2.6	4.9	7.24	PreObese
No	0	No	1.4	2.4	7.284	ObeseClass3
No	0	No	-6.7	7	7.172	NormalWeight
No	0	No	-3.7	7.4	7.163	NormalWeight
No	0	No	-5.2	6.7	7.21	NormalWeight
Yes	0	No	-3.8	4.4	7.277	ObeseClass3
No	0	No	-4.1	6.3	7.167	ObeseClass2
No	0	No	1.4	2.4	7.28	UnderWeight
No	0	No	2.9	2.2	7.37	ObeseClass1
No	0	No	1.2	1.9	7.318	NormalWeight
No	0	Other	2.2	2.3	7.31	UnderWeight
No	0	No	-2.6	3.2	7.36	ObeseClass1
Yes	0	No	-3.2	3.1	7.251	NormalWeight
No	0	No	2.6	3.9	7.21	ObeseClass3
No	0	No	2.3	4.7	7.236	NormalWeight
No	0	No	-3.4	6.3	7.166	ObeseClass1
No	0	No	-3.1	4.4	7.33	NormalWeight
No	0	No	1.5	3	7.36	NormalWeight
No	0	No	1.5	2.8	7.303	ObeseClass1
Yes	0	No	2.4	3.7	7.3	NormalWeight
Yes	0	No	2.3	2.8	7.31	NormalWeight
Yes	0	No	4.1	5.3	7.287	NormalWeight
No	0	No	1.3	3.6	7.3	NormalWeight
No	0	No	-4.5	2.9	7.32	PreObese
No	0	Other	-4.8	3.3	7.253	NormalWeight
No	0	No	1.8	4.9	7.3	UnderWeight
Yes	0	No	1.8	2.1	7.261	ObeseClass1
No	0	No	-2.7	4.4	7.228	NormalWeight
No	0	No	-1.5	2.7	7.29	ObeseClass2
No	0	No	-1.9	2.4	7.342	NormalWeight
Yes	0	No	-2.3	3.7	7.315	NormalWeight
No	0	No	0.4	2.8	7.287	NormalWeight
No	0	No	-2.3	5.6	7.26	ObeseClass3
No	0	No	0	1.7	7.32	NormalWeight
No	0	No	0.3	3.8	7.26	ObeseClass1
Yes	0	No	-8.1	5.9	7.182	NormalWeight
No	0	No	-3.7	4.1	7.274	NormalWeight
No	0	No	-6.5	6.5	7.27	UnderWeight
Yes	0	No	-2.2	4.8	7.241	NormalWeight
No	0	No	-2	3.8	7.31	PreObese
No	0	No	0.2	1.7	7.336	NormalWeight
No	0	No	-1.2	1.9	7.3	PreObese
No	0	No	-3.8	4	7.22	NormalWeight
No	0	No	-2.8	3.7	7.282	ObeseClass1

No	0	No	-1	4.3	7.223	ObeseClass2
No	0	No	6.6	0.8	7.41	NormalWeight
No	0	No	-1.1	3.5	7.27	UnderWeight
Yes	0	No	0.6	2.7	7.334	PreObese
Yes	0	No	-0.9	2.5	7.3	ObeseClass1
No	0	No	-4.2	5.3	7.2	ObeseClass1
No	0	No	0.2	3.1	7.28	UnderWeight
No	0	No	3.4	2.3	7.32	PreObese
Yes	0	No	-2.1	3.1	7.27	UnderWeight
No	0	No	2.3	2.9	7.31	NormalWeight
No	0	No	-6.8	4.8	7.217	PreObese
No	0	No	-1.6	3.9	7.301	UnderWeight
Yes	0	No	2.1	3.6	7.27	NormalWeight
No	0	No	-2.1	2.5	7.26	PreObese
Yes	0	No	0.8	3.2	7.27	ObeseClass1
No	0	No	-4.3	7.3	7.158	UnderWeight
No	0	No	0.5	3.8	7.3	ObeseClass1
No	0	No	-3.7	5.8	7.177	PreObese
No	0	No	-2.1	3	7.283	ObeseClass1
No	0	No	-5.2	8.6	7.14	NormalWeight
No	0	No	-3.8	5.6	7.211	NormalWeight
No	0	No	-6.5	5.7	7.33	PreObese
No	0	No	0	1.2	7.357	NormalWeight
No	0	No	-7	0	7.314	NormalWeight
No	0	No	-2.5	3	7.33	PreObese
No	0	No	1.5	2.1	7.28	ObeseClass1
No	0	No	2.6	3.6	7.35	PreObese
Yes	0	No	0.4	3.7	7.26	NormalWeight
Yes	0	Other	-2.4	3.1	7.315	ObeseClass1
No	0	No	-2.5	3.8	7.255	ObeseClass3
No	0	No	-3.6	2.8	7.297	PreObese
Yes	0	No	-3.8	5.3	7.282	ObeseClass1
No	0	No	1	3	7.27	NormalWeight
No	0	No	-0.9	3.5	7.336	PreObese
No	0	No	-1.8	2.9	7.255	NormalWeight
Yes	0	No	-0.1	2.5	7.306	ObeseClass3
No	0	No	2.2	4	7.34	PreObese
No	0	No	5.3	6.1	7.22	NormalWeight
No	0	No	1	5.3	7.25	UnderWeight
No	0	No	-9.6	6.1	7.15	ObeseClass1
No	0	No	0.2	4	7.312	PreObese
No	0	No	1.5	3.8	7.21	NormalWeight
No	0	No	-2.8	3	7.34	NormalWeight
No	0	No	0.3	0.8	7.398	NormalWeight
Yes	0	No	7.4	6.9	7.142	ObeseClass3
Yes	0	No	7.2	8.7	7.22	UnderWeight
No	0	No	-7.6	5.1	7.36	PreObese
No	0	No	-2	5.2	7.19	NormalWeight

No	0	No	-2.8	2.4	7.327 PreObese
Yes	0	No	4.6	6.7	7.172 NormalWeight
No	0	No	0.3	2.5	7.35 ObeseClass3
No	0	No	-5.6	4.9	7.276 PreObese
No	0	No	6.7	5.5	7.34 NormalWeight
No	0	No	-1.2	3.1	7.27 PreObese
No	0	No	0.3	2.5	7.28 ObeseClass3
No	0	No	2.8	2.4	7.29 NormalWeight
No	0	No	-4.7	5.1	7.334 ObeseClass2
Yes	0	No	3.1	4.5	7.24 NormalWeight
Yes	0	No	1.1	1.8	7.28 UnderWeight
Yes	0	No	-7.3	7.8	7.16 PreObese
Yes	0	No	-5.4	5.7	7.233 NormalWeight
No	0	No	-3.3	7	7.23 PreObese
No	0	No	1.7	3.4	7.314 PreObese
No	0	No	-4.5	3.4	7.23 NormalWeight
No	0	No	-7.3	5.7	7.206 PreObese
No	0	No	2.9	1.7	7.41 NormalWeight
No	0	No	5.1	5.7	7.2 PreObese
Yes	0	No	-5.7	3.9	7.34 NormalWeight
No	0	No	3	4.1	7.31 NormalWeight
No	0	No	-0.9	4.3	7.367 UnderWeight
No	0	No	-4.1	6	7.262 NormalWeight
No	0	Other	-7.3	3.4	7.335 NormalWeight
No	0	No	0.3	3.7	7.33 NormalWeight
No	0	No	1.4	0.7	7.455 ObeseClass1
Yes	0	No	-2.1	3.4	7.307 ObeseClass2
No	0	No	-6.4	6	7.176 UnderWeight
No	0	No	1.3	6.2	7.228 NormalWeight
No	0	No	-0.8	3.6	7.251 PreObese
No	0	No	-4.5	2.8	7.39 NormalWeight
No	0	No	-1.9	3.5	7.289 NormalWeight
No	0	No	-7.3	7.8	7.03 ObeseClass2
No	0	No	-5.9	5.3	7.329 NormalWeight
No	0	No	-4.7	6.3	7.223 NormalWeight
No	0	No	2.9	2.8	7.381 NormalWeight
No	0	No	-3.8	3.1	7.399 NormalWeight
Yes	0	No	-3.4	6.4	7.196 UnderWeight
No	0	No	-3.2	3.6	7.29 NormalWeight
No	0	No	0.5	4	7.306 UnderWeight
No	0	No	-0.9	2.7	7.319 PreObese
No	0	No	1.9	5.3	7.28 PreObese
Yes	0	No	0.6	2.1	7.395 PreObese
No	0	No	-2.5	4.8	7.242 PreObese
Yes	0	No	2.4	5.7	7.22 NormalWeight
No	0	No	-1.1	3.6	7.286 PreObese
No	0	No	-5	5.1	7.325 UnderWeight
Yes	0	No	6.3	6.7	7.21 PreObese

No	0	No	-2.8	2.8	7.48	NormalWeight
No	0	No	-2.9	3.6	7.28	ObeseClass2
No	0	No	-0.1	2.5	7.231	ObeseClass3
Yes	0	No	0.3	1.9	7.26	ObeseClass1
No	0	No	-3.4	5.5	7.18	NormalWeight
No	0	No	-2.5	3.1	7.3	NormalWeight
Yes	0	No	5	4.1	7.22	PreObese
No	0	No	-10	6.3	7.18	NormalWeight
No	0	No	-1	2.2	7.29	UnderWeight
No	0	No	-3.4	5.4	7.19	ObeseClass1
Yes	0	No	-3.5	4.7	7.256	ObeseClass1
No	0	No	-3.9	2.9	7.28	NormalWeight
No	0	No	1.7	3.3	7.26	NormalWeight
No	0	No	-2.1	4.3	7.28	PreObese
No	0	No	2.3	5.6	7.232	NormalWeight
No	0	No	0.6	2.6	7.334	ObeseClass2
Yes	0	No	2.9	4.6	7.22	PreObese
Yes	0	No	-2.7	1.8	7.31	ObeseClass3
No	0	No	-0.1	3.3	7.244	UnderWeight
No	0	No	-2.1	6.1	7.205	PreObese
Yes	0	No	5.3	7.4	7.14	NormalWeight
No	0	No	-0.6	5.1	7.302	PreObese
No	0	No	-2.7	5.8	7.198	NormalWeight
No	0	No	2.4	4	7.28	NormalWeight
No	0	No	-2.7	3.8	7.3	NormalWeight
No	0	No	-5	6.2	7.181	ObeseClass1
Yes	0	No	0.1	3.1	7.318	ObeseClass1
No	0	No	2	4.1	7.25	NormalWeight
Yes	0	No	5.8	5.1	7.304	PreObese
Yes	0	No	2.8	3.1	7.29	UnderWeight
No	0	No	-3.4	2.8	7.318	NormalWeight
No	0	No	-2.7	5.3	7.16	ObeseClass3
No	0	No	1.9	3.9	7.3	ObeseClass2
Yes	0	No	0	2.9	7.32	NormalWeight
No	0	No	-8.2	8.7	7.344	NormalWeight
No	0	No	-3.9	3.6	7.307	NormalWeight
No	0	No	-6.2	6.2	7.23	ObeseClass1
No	0	No	-0.3	2.6	7.343	PreObese
No	0	No	0.6	3.6	7.31	PreObese
Yes	0	No	1	2.9	7.266	UnderWeight
No	0	No	5.1	8.4	7.16	NormalWeight
Yes	0	No	1.5	5	7.261	NormalWeight
Yes	0	No	0.3	3.6	7.26	ObeseClass2
No	0	No	0.7	2.5	7.299	NormalWeight
Yes	0	No	-4.6	5.8	7.206	NormalWeight
Yes	0	No	-3.8	5.5	7.272	ObeseClass1
No	0	No	-4.4	2.4	7.39	NormalWeight
No	0	No	-2.8	3.3	7.332	NormalWeight

No	0	No	2.3	5.1	7.21	NormalWeight
No	0	No	4	5.2	7.39	NormalWeight
No	0	No	0.9	3.9	7.315	PreObese
No	0	No	-4.1	3.2	7.26	NormalWeight
No	0	No	-0.5	5.6	7.161	ObeseClass2
Yes	0	No	-7.9	5.8	7.301	UnderWeight
No	0	No	-2.6	2.6	7.48	NormalWeight
No	0	No	2.7	3.7	7.41	PreObese
No	0	No	8.4	5.7	7.205	PreObese
Yes	0	No	-3.5	4.8	7.28	PreObese
No	0	No	-2.7	5.2	7.24	NormalWeight
No	0	No	1.5	3.5	7.27	NormalWeight
No	0	No	2.1	1.4	7.36	PreObese
Yes	0	No	-4.4	4.2	7.339	ObeseClass1
Yes	0	No	-0.5	3	7.264	NormalWeight
No	0	No	1.5	2.9	7.255	UnderWeight
Yes	0	No	-2.7	5.5	7.203	ObeseClass1
No	0	No	0	5	7.24	PreObese
Yes	0	No	8	9.2	7.11	PreObese
No	0	No	-6	6.7	7.13	PreObese
No	0	No	-9.8	6	7.146	ObeseClass3
No	0	No	4.1	5.3	7.228	PreObese
Yes	0	No	0.3	3.2	7.407	NormalWeight
No	0	No	-3	4.7	7.25	ObeseClass1
Yes	0	No	-8.2	4.8	7.371	NormalWeight
No	0	No	-1.8	4.1	7.3	PreObese
No	0	No	-4.8	5.8	7.15	ObeseClass1
No	0	No	-2.4	3.1	7.287	PreObese
No	0	No	1.4	2.8	7.28	NormalWeight
Yes	0	No	-1.2	3.2	7.277	PreObese
Yes	0	No	1.4	3.5	7.293	UnderWeight
Yes	0	No	7.5	2.1	7.382	NormalWeight
No	0	Other	-4.9	2.4	7.37	NormalWeight
No	0	No	-2.9	1.5	7.32	NormalWeight
No	0	No	-2.9	6.3	7.21	PreObese
No	0	No	-1.8	3.6	7.266	ObeseClass1
No	0	No	6.5	5.5	7.34	UnderWeight
Yes	0	No	-4.1	3.7	7.17	NormalWeight
No	0	No	-2.8	5.3	7.261	NormalWeight
Yes	0	No	2.3	3.6	7.28	NormalWeight
No	0	No	-1.3	3.8	7.247	PreObese
No	0	No	-0.5	3	7.33	NormalWeight
No	0	No	3.7	4.4	7.231	PreObese
Yes	0	No	2.2	3.9	7.25	NormalWeight
No	0	Other	5.2	3.1	7.28	NormalWeight
No	0	No	-0.4	3.5	7.255	PreObese
No	0	No	6	6.9	7.094	NormalWeight
No	0	No	-4.7	4.8	7.2	UnderWeight

Yes	0	No	-1.9	1.9	7.287	PreObese
No	0	No	0.8	1.9	7.3	UnderWeight
No	0	No	-2.3	3.7	7.294	ObeseClass3
No	0	No	-4.2	4.1	7.38	NormalWeight
No	0	No	2.6	3	7.3	NormalWeight
Yes	0	No	-1.6	3.9	7.288	UnderWeight
No	0	No	-0.5	5.4	7.28	UnderWeight
No	0	No	1.8	2.3	7.3	PreObese
No	0	No	-5.9	4.4	7.338	ObeseClass2
No	0	No	-4.6	8.8	7.191	UnderWeight
Yes	0	No	1.2	3.4	7.327	ObeseClass1
Yes	0	No	-0.9	2.4	7.327	ObeseClass1
No	0	No	-3	6.3	7.196	ObeseClass1
No	0	No	-4.7	3.9	7.311	NormalWeight
No	0	No	-1.6	2.8	7.399	PreObese
No	0	No	-2.7	3.9	7.303	PreObese
No	0	No	-5.7	6.6	7.235	NormalWeight
No	0	No	-1.5	2.8	7.244	NormalWeight
No	0	No	-0.9	3.7	7.361	NormalWeight
Yes	0	No	-2.5	3.1	7.294	ObeseClass1
No	0	No	-1.9	5.1	7.257	ObeseClass1
Yes	0	No	-10.7	7.8	7.169	NormalWeight
Yes	0	No	2.5	3	7.32	NormalWeight
Yes	0	No	-2	3	7.306	PreObese
No	0	No	1.3	2.5	7.306	UnderWeight
No	0	No	0.9	1.5	7.312	UnderWeight
Yes	0	No	-0.3	2.3	7.314	NormalWeight
No	0	No	-1.7	3.9	7.291	NormalWeight
No	0	No	1.5	4	7.17	PreObese
No	0	No	-6.4	3.7	7.4	NormalWeight
No	0	No	-3.5	6	7.233	UnderWeight
No	0	No	0.4	2.1	7.36	ObeseClass1
No	0	No	-0.4	5.5	7.204	UnderWeight
Yes	0	No	0.3	1.7	7.32	NormalWeight
No	0	No	-6.6	5.8	7.2	ObeseClass1
No	0	No	-4.5	4.6	7.38	NormalWeight
No	0	No	4	6.1	7.21	NormalWeight
Yes	0	No	-2.9	2.1	7.31	ObeseClass3
No	0	Other	-2.4	4	7.213	PreObese
No	0	No	7.1	8	7.2	NormalWeight
Yes	0	No	-1.9	3.6	7.45	NormalWeight
No	0	No	-1.5	6.1	7.3	ObeseClass1
Yes	0	No	5.1	3.9	7.38	NormalWeight
No	0	No	-5.5	5.6	7.197	NormalWeight
Yes	0	No	5.8	6.7	7.16	ObeseClass1
No	0	No	1.1	2.4	7.337	ObeseClass2
No	0	No	-3.2	2.6	7.24	NormalWeight
No	0	No	1.6	3.4	7.282	PreObese

Yes	0	No	-1.9	3.3	7.272	PreObese
No	0	No	-1.9	6.2	7.178	UnderWeight
No	0	No	-4.7	8.1	7.23	NormalWeight
Yes	0	No	-6.4	6.8	7.25	NormalWeight
Yes	0	No	-0.8	2.2	7.344	NormalWeight
Yes	0	No	1.6	4.1	7.298	NormalWeight
Yes	0	No	-10.7	3.6	7.262	NormalWeight
No	0	No	-7.1	6.8	7.16	ObeseClass3
No	0	No	-6.2	5.1	7.196	NormalWeight
No	0	No	-0.8	3.5	7.302	NormalWeight
No	0	No	-1.2	3.1	7.388	ObeseClass2
Yes	0	No	-2.7	4.6	7.189	ObeseClass1
Yes	0	No	-3.9	5.1	7.332	UnderWeight
No	0	No	-3.6	5.5	7.189	ObeseClass1
No	0	No	-1.8	3.2	7.28	ObeseClass1
No	0	No	-2.6	4.3	7.255	NormalWeight
Yes	0	No	-1.8	1.8	7.27	NormalWeight
No	0	No	0.8	2.2	7.29	NormalWeight
No	0	No	2.9	4.3	7.227	ObeseClass1
No	0	No	1.3	4.2	7.24	PreObese
No	0	No	5.1	7.7	7.14	ObeseClass1
No	0	No	2.4	2.2	7.245	ObeseClass3
Yes	0	No	-2.6	4.9	7.252	NormalWeight
No	0	No	-1.6	4.2	7.33	ObeseClass1
Yes	0	No	-0.6	2.6	7.36	ObeseClass1
Yes	0	No	4.8	3.3	7.256	ObeseClass1
No	0	No	-1.7	2.8	7.298	ObeseClass2
No	0	No	-6.4	4.5	7.36	NormalWeight
No	0	No	-6.3	2.3	7.365	NormalWeight
No	0	No	-2.6	4.5	7.216	NormalWeight
No	0	No	-7.8	5.8	7.264	NormalWeight
Yes	0	No	-5.4	4.4	7.429	PreObese
No	0	No	2.9	2.1	7.32	UnderWeight
No	0	No	0.1	2.6	7.287	ObeseClass1
Yes	0	No	-0.9	2.6	7.313	NormalWeight
No	0	No	-0.6	3.9	7.23	PreObese
No	0	No	2.5	4.7	7.188	NormalWeight
No	0	No	1.6	2.7	7.3	NormalWeight
No	0	No	-1.6	2.9	7.28	UnderWeight
Yes	0	No	-8.8	5.9	7.264	PreObese
No	0	No	0.4	2.1	7.289	ObeseClass3
No	0	No	-5.4	2.6	7.354	PreObese
No	0	No	3.7	4.4	7.18	PreObese
No	0	No	2.2	4.2	7.24	PreObese
No	0	No	0.5	3.1	7.29	ObeseClass2
No	0	No	0.8	2.9	7.22	NormalWeight
No	0	No	5.5	4.9	7.242	NormalWeight
No	0	No	-2.3	2.4	7.28	NormalWeight

Yes	0	No	3.6	2.5	7.438	NormalWeight
Yes	0	No	-3.2	4.1	7.33	NormalWeight
No	0	No	-6.6	4.4	7.286	UnderWeight
No	0	No	-9.1	7.5	7.25	NormalWeight
No	0	No	-0.1	4.5	7.32	UnderWeight
No	0	No	-6.9	9.7	7.11	PreObese
Yes	0	No	-0.6	3.4	7.302	NormalWeight
No	0	No	4	5.6	7.14	NormalWeight
Yes	0	No	-4.7	6.3	7.205	NormalWeight
No	0	No	2.9	2.1	7.424	ObeseClass3
Yes	0	No	1	2.1	7.327	PreObese
No	0	Other	6.8	4.8	7.334	NormalWeight
No	0	No	-3.4	5.6	7.22	ObeseClass1
Yes	0	No	-4.1	5.9	7.225	NormalWeight
No	0	No	0.2	3.7	7.3	NormalWeight
No	0	No	2	5.6	7.269	NormalWeight
No	0	No	-3.6	3.5	7.347	NormalWeight
No	0	No	-3.8	3.5	7.298	NormalWeight
Yes	0	No	-2.5	4.1	7.292	NormalWeight
No	0	No	4.6	6.2	7.21	ObeseClass1
No	0	No	0	3.3	7.26	ObeseClass2
No	0	No	-3.7	1.1	7.27	ObeseClass1
Yes	0	No	-4.5	4.5	7.2	ObeseClass1
Yes	0	No	-3	3.2	7.32	PreObese
Yes	0	No	-3.1	5.3	7.234	NormalWeight
No	0	No	1.1	1.9	7.34	PreObese
No	0	No	0.6	2.5	7.317	PreObese
No	0	No	0.5	2.1	7.3	PreObese
Yes	0	No	0.2	2	7.34	PreObese
Yes	0	No	-3.6	3.7	7.4	UnderWeight
No	0	No	0.4	2.8	7.266	ObeseClass2
Yes	0	No	8.5	0.3	7.5	NormalWeight
No	0	No	0.9	3	7.265	NormalWeight
No	0	No	5.5	5.5	7.29	ObeseClass1
No	0	No	0.6	3.2	7.274	PreObese
Yes	0	No	-3.4	3	7.4	UnderWeight
No	0	No	2.6	2.3	7.413	NormalWeight
No	0	No	2.4	4.2	7.301	PreObese
No	0	No	-7.5	3.7	7.27	PreObese
Yes	0	No	1.3	3	7.37	PreObese
No	0	No	-5.4	6.4	7.24	NormalWeight
No	0	No	-3.5	4.5	7.222	NormalWeight
No	0	No	-4.5	5.2	7.213	NormalWeight
Yes	0	No	0.8	2.3	7.2	PreObese
Yes	0	No	-0.7	2.8	7.3	UnderWeight
Yes	0	No	-0.9	2.2	7.293	NormalWeight
No	0	No	-1.5	4.5	7.23	NormalWeight
No	0	No	-1.8	4.1	7.22	NormalWeight

No	0	Other	3.9	2.2	7.289	PreObese
No	0	No	-1.1	3.4	7.244	ObeseClass1
No	0	No	-2.3	3.2	7.3	NormalWeight
Yes	0	No	-5.3	4.3	7.26	NormalWeight
Yes	0	No	5.6	6.7	7.26	PreObese
No	0	No	4.9	3.8	7.31	NormalWeight
No	0	No	-2.9	3.2	7.295	ObeseClass1
No	0	No	-4.6	5.3	7.24	NormalWeight
Yes	0	No	0.9	2.5	7.4	NormalWeight
No	0	No	-2.5	2.3	7.4	ObeseClass1
No	0	No	-1.6	3.6	7.29	PreObese
No	0	No	2.1	2.1	7.238	PreObese
Yes	0	No	3.1	3.2	7.276	PreObese
No	0	No	0.9	3.8	7.24	NormalWeight
No	0	No	2.2	2.4	7.304	NormalWeight
No	0	No	-3.6	4.4	7.269	NormalWeight
No	0	No	-3.2	3.4	7.396	NormalWeight
No	0	No	-4.4	3.8	7.29	NormalWeight
No	0	No	0.6	2.8	7.29	NormalWeight
Yes	0	No	-4.8	4.4	7.25	ObeseClass1
No	0	No	-1.2	2.7	7.33	ObeseClass3
Yes	0	No	3.8	3.4	7.35	NormalWeight
No	0	No	-0.7	3.4	7.322	NormalWeight
No	0	No	-3.2	0	7.17	PreObese
No	0	No	4.6	2.7	7.315	NormalWeight
No	0	No	1.3	2.8	7.2	PreObese
No	0	No	-3.1	4.2	7.169	NormalWeight
No	0	No	-1	4.1	7.215	PreObese
No	0	No	1.5	2.5	7.33	PreObese
Yes	0	No	-6.7	6.3	7.335	NormalWeight
No	0	Other	-2.3	3.2	7.297	NormalWeight
No	0	No	-0.1	2.1	7.2	ObeseClass1
No	0	No	-7.3	6.8	7.209	PreObese
No	0	No	-0.2	3.4	7.288	ObeseClass1
Yes	0	No	2.2	3.1	7.313	PreObese
No	0	No	0.4	2.5	7.305	ObeseClass2
Yes	0	No	0.2	2.3	7.321	PreObese
Yes	0	No	-3.3	5.4	7.198	PreObese
No	0	Other	1.2	2.1	7.31	NormalWeight
Yes	0	No	0.8	2.3	7.37	NormalWeight
No	0	No	2.1	3.5	7.25	ObeseClass1
No	0	No	-1	2.8	7.321	NormalWeight
No	0	No	-1.8	0.8	7.43	NormalWeight
No	0	No	0.4	2	7.346	ObeseClass1
No	0	No	-4	5.4	7.261	PreObese
Yes	0	No	0.2	3.2	7.355	ObeseClass1
No	0	No	1.4	3.2	7.312	NormalWeight
No	0	No	1.1	3.6	7.264	PreObese

No	0	No	-1.6	1.9	7.312	ObeseClass2
No	0	No	-1.6	5.4	7.26	PreObese
No	0	No	5.8	4	7.23	NormalWeight
No	0	No	-1.5	4.4	7.39	NormalWeight
No	0	No	-11.1	8	7.098	NormalWeight
No	0	No	-3.4	4.6	7.215	PreObese
No	0	No	14.5	1	7.5	PreObese
Yes	0	No	2.8	2.9	7.29	UnderWeight
No	0	No	-5.1	4.1	7.177	NormalWeight
No	0	No	-0.9	5.7	7.19	UnderWeight
Yes	0	No	-0.1	4.4	7.24	PreObese
No	0	No	-0.2	2.8	7.263	PreObese
No	0	No	1.8	4.1	7.33	UnderWeight
No	0	No	4	4.1	7.46	NormalWeight
No	0	No	-1.5	2	7.33	ObeseClass2
No	0	No	3.9	5.3	7.233	UnderWeight
No	0	Other	-4.5	1.9	7.36	NormalWeight
No	0	No	0.4	2.3	7.31	UnderWeight
No	0	No	-0.7	4.5	7.28	NormalWeight
No	0	No	-0.1	1.9	7.314	NormalWeight
No	0	No	-2.5	2.3	7.341	PreObese
No	0	No	-1.9	2	7.341	PreObese
No	0	No	-2	5.1	7.25	NormalWeight
Yes	0	No	-0.6	2.5	7.259	UnderWeight
No	0	No	-4.1	5.4	7.243	PreObese
No	0	No	-4	5.5	7.205	NormalWeight
Yes	0	No	0.3	2.3	7.29	NormalWeight
No	0	No	0.8	2.5	7.271	NormalWeight
No	0	No	-0.4	5.8	7.16	UnderWeight
No	0	No	0.2	3.8	7.29	NormalWeight
No	0	No	-1	2.5	7.308	ObeseClass1
No	0	No	0.7	3.1	7.27	NormalWeight
No	0	No	-1.2	1.6	7.44	PreObese
No	0	No	8.5	8	7.202	NormalWeight
No	0	No	-3.7	4.7	7.203	NormalWeight
No	0	No	0.8	2.8	7.302	NormalWeight
No	0	No	-4.2	5.1	7.207	UnderWeight
No	0	No	2.3	0.8	7.5	NormalWeight
No	0	No	0.8	3.9	7.282	NormalWeight
No	0	No	-1.8	4.4	7.248	ObeseClass3
No	0	No	1.5	3.8	7.264	PreObese
Yes	0	No	-5.1	4.2	7.283	PreObese
No	0	No	0.7	4.2	7.269	NormalWeight
No	0	No	2.7	2.3	7.31	ObeseClass2
Yes	0	No	-1	4.1	7.255	ObeseClass2
No	0	No	1.6	2.8	7.311	PreObese
No	0	No	-4.7	5.7	7.228	NormalWeight
No	0	No	1.1	3	7.28	NormalWeight

No	0	No	-2.9	4	7.23	PreObese
Yes	0	No	2.4	3.2	7.288	UnderWeight
No	0	No	-3.3	7.1	7.115	NormalWeight
No	0	No	-6.7	5.8	7.388	NormalWeight
No	0	No	-3.6	4.5	7.28	ObeseClass3
No	0	No	0.7	2.8	7.263	ObeseClass2
No	0	No	-0.4	2.3	7.36	NormalWeight
No	0	No	0.4	3	7.321	PreObese
No	0	No	8.3	5.8	7.38	PreObese
No	0	Other	-4.6	3.2	7.5	ObeseClass1
Yes	0	No	3.6	3.8	7.31	UnderWeight
Yes	0	No	0	4.2	7.256	ObeseClass1
Yes	0	No	-2.3	5	7.365	ObeseClass2
Yes	0	No	3.2	1.7	7.218	NormalWeight
Yes	0	No	-4.9	4.4	7.292	ObeseClass1
Yes	0	No	0.6	3.2	7.3	NormalWeight
No	0	No	0.8	3.8	7.311	NormalWeight
Yes	0	No	2.2	2.1	7.34	PreObese
No	0	No	-1.9	4.1	7.21	NormalWeight
No	0	No	-5.5	3.6	7.458	NormalWeight
No	0	No	-3.3	3.4	7.37	NormalWeight
No	0	No	4.3	6.1	7.21	UnderWeight
No	0	No	7.2	8.2	7.187	PreObese
Yes	0	No	-8.2	8.3	7.07	NormalWeight
No	0	No	-5.1	7.8	7.318	NormalWeight
No	0	No	-4.4	5.4	7.254	NormalWeight
No	0	No	-1.5	4.3	7.29	PreObese
No	0	No	-0.3	2.6	7.28	ObeseClass1
No	0	No	-2.9	2.4	7.357	ObeseClass1
No	0	No	7.9	5	7.148	ObeseClass1
Yes	0	No	-6.2	9.5	7.12	UnderWeight
No	0	No	-1.8	4.1	7.24	NormalWeight
Yes	0	No	-1.7	5.6	7.221	NormalWeight
No	0	No	1.5	3.6	7.23	NormalWeight
No	0	No	-1.6	2.5	7.271	UnderWeight
No	0	No	2.7	5.6	7.118	UnderWeight
Yes	0	No	1.1	2.3	7.32	NormalWeight
No	0	No	4.5	3.4	7.407	NormalWeight
Yes	0	No	0	3.2	7.293	ObeseClass3
No	0	No	-5.6	4.2	7.23	NormalWeight
No	0	No	-1.3	3.7	7.29	NormalWeight
No	0	No	-0.6	2.9	7.29	PreObese
Yes	0	No	4.8	7	7.25	NormalWeight
Yes	0	No	-0.1	2.6	7.321	NormalWeight
No	0	No	-1.6	3	7.299	NormalWeight
No	0	No	-3.3	4.4	7.26	NormalWeight
No	0	No	6.3	6	7.26	UnderWeight
No	0	Other	-2.3	2.7	7.27	ObeseClass2

Yes	0	No	-3.4	2.9	7.315	PreObese
No	0	No	0.5	1.7	7.38	PreObese
No	0	No	-5.3	7.5	7.299	UnderWeight
No	0	No	0.6	2.9	7.295	NormalWeight
Yes	0	No	0.8	4.4	7.28	UnderWeight
No	0	No	-1.4	3.2	7.3	ObeseClass3
No	0	No	4.7	4.5	7.34	NormalWeight
No	0	No	-4.2	2.8	7.38	PreObese
Yes	0	No	1.1	4	7.26	PreObese
Yes	0	No	-6.3	4.8	7.261	ObeseClass1
No	0	No	-0.6	4.4	7.279	PreObese
No	0	No	2.9	2.5	7.292	NormalWeight
Yes	0	No	-3.4	3.4	7.3	ObeseClass2
No	0	No	2.3	2.6	7.24	ObeseClass2
No	0	No	-2.7	5.1	7.22	NormalWeight
No	0	No	-7.8	6.2	7.288	PreObese
Yes	0	No	1.1	3.1	7.31	ObeseClass3
No	0	No	0.2	4.2	7.264	NormalWeight
No	0	No	5.5	4.9	7.17	PreObese
Yes	0	No	7	8.3	7.237	ObeseClass1
No	0	No	6	6.3	7.17	PreObese
No	0	No	-1.8	3.7	7.331	NormalWeight
Yes	0	No	-1.9	3.2	7.275	UnderWeight
Yes	0	No	-15.3	9.9	7.13	UnderWeight
No	0	No	-4.1	3.3	7.287	NormalWeight
Yes	0	No	1.2	4.1	7.279	NormalWeight
Yes	0	No	-2.6	5.1	7.26	PreObese
Yes	0	No	0.2	2.9	7.257	UnderWeight
No	0	No	-1.6	3.8	7.416	NormalWeight
No	0	No	2.4	3.3	7.43	NormalWeight
No	0	No	-5.1	5.1	7.296	PreObese
No	0	No	2.1	4.2	7.22	ObeseClass1
Yes	0	No	0.7	2.9	7.29	NormalWeight
Yes	0	No	3.5	4.2	7.38	ObeseClass1
Yes	0	No	-2	6.2	7.2	UnderWeight
No	0	No	2.4	2.8	7.3	UnderWeight
No	0	No	-1.2	1.9	7.266	ObeseClass2
No	0	No	-4.5	3.9	7.249	NormalWeight
No	0	No	-8.7	8	7.164	PreObese
No	0	No	2.2	3	7.418	ObeseClass1
Yes	0	No	-3.5	6.7	7.16	NormalWeight
No	0	No	-3.4	6.3	7.098	ObeseClass2
Yes	0	No	0.9	1.7	7.31	ObeseClass2
No	0	No	2.7	5.6	7.27	NormalWeight
Yes	0	No	1.9	2.7	7.24	ObeseClass1
Yes	0	No	-6.2	6.6	7.206	PreObese
Yes	0	No	0.6	2.8	7.273	ObeseClass2
No	0	No	-4.9	4.9	7.185	UnderWeight

Yes	0	Other	0.8	1.8	7.4	ObeseClass1
No	0	No	0.8	3.1	7.25	NormalWeight
Yes	0	No	-5.3	3.7	7.218	NormalWeight
No	0	No	2.9	1.8	7.301	PreObese
No	0	Other	-3	5.1	7.29	UnderWeight
Yes	0	No	5.1	3.7	7.359	PreObese
No	0	No	-7.9	6.4	7.29	PreObese
No	0	No	1.8	6	7.19	ObeseClass2
Yes	0	No	0.1	2	7.362	NormalWeight
Yes	0	No	-6.9	6	7.226	ObeseClass1
No	0	No	2.3	4	7.38	NormalWeight
No	0	No	1.2	5.6	7.268	PreObese
Yes	0	No	6.2	5.8	7.233	ObeseClass1
No	0	No	0.7	3.1	7.28	NormalWeight
Yes	0	No	6.9	5.9	7.3	NormalWeight
Yes	0	No	-4.1	6.1	7.202	UnderWeight
No	0	No	4.3	4.3	7.34	PreObese
No	0	No	5.6	7.1	7.18	NormalWeight
No	0	No	-3.4	4.4	7.283	NormalWeight
No	0	No	-2	4.2	7.38	ObeseClass1
Yes	0	No	-0.2	2.9	7.3	PreObese
Yes	0	No	3.5	3.9	7.33	NormalWeight
No	0	No	0.9	2.7	7.287	NormalWeight
No	0	No	-5.2	3.8	7.31	NormalWeight
No	0	No	-6.2	4	7.25	UnderWeight
No	0	No	1.9	0.6	7.49	UnderWeight
No	0	No	-0.7	3.4	7.26	UnderWeight
Yes	0	No	0.4	3.1	7.27	ObeseClass3
No	0	No	3.6	3.8	7.37	UnderWeight
Yes	0	No	0.9	3.5	7.299	UnderWeight
Yes	0	No	-2.5	3.7	7.38	NormalWeight
Yes	0	No	-2.7	1.7	7.23	PreObese
No	0	Other	-3	4.5	7.203	NormalWeight
Yes	0	No	-4.1	5.3	7.21	PreObese
Yes	0	No	0.9	3.1	7.3	PreObese
Yes	0	No	1.1	2.2	7.305	PreObese
Yes	0	No	2.7	5.4	7.236	NormalWeight
No	0	No	-2.8	3.4	7.361	PreObese
Yes	0	No	-4.9	3.4	7.29	UnderWeight
No	0	No	-2	3.3	7.309	PreObese
Yes	0	No	-2.8	4.5	7.343	NormalWeight
No	0	No	0.6	4.9	7.33	ObeseClass1
No	0	No	0.1	2.3	7.25	PreObese
No	0	No	-3.6	4.6	7.311	NormalWeight
Yes	0	No	3.8	4.6	7.25	PreObese
No	0	No	-2.1	3.6	7.4	PreObese
No	0	No	3.9	3.6	7.36	PreObese
No	0	No	-0.1	2.1	7.29	NormalWeight

No	0	No	0.4	2.1	7.31	NormalWeight
No	0	No	-1.6	3.9	7.312	NormalWeight
Yes	0	No	1	2.1	7.29	NormalWeight
No	0	No	-5.2	3.7	7.399	NormalWeight
No	0	No	-2.3	4.2	7.361	NormalWeight
Yes	0	No	4.3	3.2	7.369	NormalWeight
No	0	No	4.1	4.9	7.236	NormalWeight
Yes	0	No	-3.7	3	7.28	NormalWeight
No	0	No	0.9	3.5	7.25	UnderWeight
No	1	No	-10.6	9	7.14	ObeseClass1
No	1	No	5.2	4.7	7.245	NormalWeight
Yes	1	No	-1.3	3.7	7.282	ObeseClass2
No	1	No	1	4.4	7.22	ObeseClass2
No	1	No	-4.1	4.6	7.412	ObeseClass3
No	1	No	-0.8	3.1	7.203	ObeseClass2
No	1	No	-3.8	5	7.311	UnderWeight
Yes	1	No	-3.4	4	7.24	ObeseClass2
No	1	No	2.8	4.8	7.32	NormalWeight
No	1	No	2.7	2	7.364	NormalWeight
No	1	Other	-3	4.9	7.162	UnderWeight
No	1	No	-10.6	7	7.2	ObeseClass1
No	1	No	2.6	4.3	7.205	UnderWeight
Yes	1	No	1.7	2.3	7.321	NormalWeight
No	1	No	-0.8	4.1	7.219	ObeseClass2
No	1	No	-2	3.8	7.232	ObeseClass3
No	1	No	2.3	3.2	7.305	ObeseClass3
No	1	No	0.5	2.7	7.23	ObeseClass1
No	1	No	-2	5.1	7.17	NormalWeight
No	1	No	-1.6	3.6	7.257	NormalWeight
No	1	No	-3.9	5.9	7.254	NormalWeight
No	1	No	2.5	4.7	7.204	ObeseClass1
No	1	No	0.7	2.4	7.316	ObeseClass1
No	1	No	-3.4	3.7	7.351	UnderWeight
No	1	No	-4.6	7.1	7.131	ObeseClass2
Yes	1	No	1.9	5.3	7.24	PreObese
No	1	No	5.9	5.9	7.304	UnderWeight
No	1	No	6.1	4.2	7.2	NormalWeight
No	1	No	5	8.1	7.086	PreObese
No	1	No	-2.5	2.3	7.306	PreObese
No	1	No	-0.4	4.5	7.278	PreObese
Yes	1	No	-7.9	4.5	7.349	PreObese
No	1	No	-6.8	0	7.165	NormalWeight
No	1	No	0.6	2.4	7.268	PreObese
Yes	1	No	4.2	6	7.223	NormalWeight
Yes	1	No	2.1	2.9	7.237	UnderWeight
No	1	No	-7.3	8.8	7.177	PreObese
Yes	1	No	-5.1	1.3	7.322	NormalWeight
No	1	No	-2.9	3.3	7.256	PreObese

Yes	1	No	-1.8	5.2	7.31	PreObese
No	1	No	-8.1	5	7.164	PreObese
Yes	1	No	-0.1	3.6	7.281	NormalWeight
No	1	No	-2	3.2	7.227	NormalWeight
No	1	No	-2.4	3.7	7.2	PreObese
No	1	No	2	2.7	7.321	PreObese
No	1	No	-5.6	4.3	7.263	PreObese
No	1	No	-1.8	2.5	7.313	ObeseClass1
No	1	No	-2.7	3.9	7.234	ObeseClass2
No	1	No	5.9	5.1	7.24	ObeseClass1
No	1	No	-5.3	2	7.437	PreObese
No	1	No	-3.7	4	7.331	ObeseClass1
No	1	Other	-6.9	5.9	7.194	NormalWeight
No	1	No	-0.8	2.4	7.321	ObeseClass2
No	1	No	5.4	5.5	7.2	PreObese
No	1	No	2.2	3.1	7.27	PreObese
No	1	No	0	2.2	7.284	ObeseClass2
Yes	1	No	-3.6	5.8	7.22	NormalWeight
No	1	No	1.5	3.1	7.28	NormalWeight
No	1	No	-3.5	3.8	7.343	ObeseClass3
No	1	No	5.5	5.4	7.18	NormalWeight
No	1	No	-5.1	3.4	7.283	PreObese
Yes	1	No	-3.6	2.9	7.346	NormalWeight
No	1	No	-0.6	1.9	7.3	PreObese
No	1	No	1.2	3.2	7.241	ObeseClass2
Yes	1	No	-3.2	5	7.198	NormalWeight
No	1	No	-5	7.1	7.277	ObeseClass1
No	1	No	-7.4	6.8	7.179	NormalWeight
No	1	No	-11.3	10.2	7.115	PreObese
No	1	No	-5.7	6	7.163	NormalWeight
No	1	No	4.1	4.8	7.19	ObeseClass3
No	1	No	-4.3	0	7.21	PreObese
No	1	No	0.4	3.8	7.28	PreObese
No	1	No	2.9	4.3	7.181	ObeseClass1
No	1	No	2.4	4	7.2	PreObese
No	1	No	-1.3	2.3	7.32	ObeseClass1
No	1	No	-3.2	3.3	7.3	NormalWeight
Yes	1	No	-4.7	5.2	7.37	ObeseClass1
No	1	No	3.5	4.5	7.284	PreObese
No	1	No	0.4	1.6	7.307	PreObese
No	1	No	-4.2	4.3	7.398	NormalWeight
No	1	No	0.1	3.5	7.355	NormalWeight
No	1	No	-3.3	2.8	7.29	PreObese
Yes	1	No	-1.5	3	7.27	PreObese
No	1	No	5.4	7.2	7.183	ObeseClass1
No	1	No	6.8	8.7	7.098	ObeseClass2
No	1	Other	3.5	7.3	7.13	PreObese
No	1	No	-6.5	2.7	7.294	PreObese

No	1	No	3.9	5.2	7.2	ObeseClass2
No	1	No	-1.9	3.3	7.435	NormalWeight
No	1	No	-1.2	2.4	7.308	ObeseClass2
No	1	No	-3	6.5	7.25	NormalWeight
No	1	No	0.1	3.8	7.241	NormalWeight
No	1	No	2.4	1.4	7.254	ObeseClass3
No	1	No	0.7	3.8	7.29	NormalWeight
No	1	No	-4.4	5	7.259	PreObese
Yes	1	No	1.2	2.4	7.279	UnderWeight
Yes	1	No	-5.1	5.6	7.359	PreObese
Yes	1	No	-3.5	2.2	7.319	NormalWeight
Yes	1	No	0	2.2	7.27	PreObese
No	1	No	-6.7	6.7	7.22	PreObese
No	1	No	0.3	3.3	7.28	PreObese
No	1	No	-1.9	2.8	7.31	PreObese
No	1	No	0.4	3	7.27	NormalWeight
Yes	1	No	-5.4	5.5	7.19	NormalWeight
No	1	No	-4.1	1.8	7.35	NormalWeight
No	1	No	-3.5	5.5	7.119	NormalWeight
Yes	1	No	-5.9	6.4	7.183	PreObese
No	1	No	-4.3	4.4	7.18	PreObese
No	1	No	-1.6	3.2	7.29	UnderWeight
No	1	No	-3.4	4.5	7.177	ObeseClass2
No	1	No	4	5.3	7.22	NormalWeight
No	1	No	-3.8	5.4	7.25	ObeseClass1
No	1	No	-3.8	4.3	7.39	NormalWeight
No	1	No	-4.5	6.5	7.22	UnderWeight
No	1	No	2.5	3.7	7.32	ObeseClass2
Yes	1	No	-4.7	6.5	7.148	NormalWeight
No	1	No	-2.9	4.9	7.306	NormalWeight
Yes	1	No	6.6	6.9	7.192	NormalWeight
No	1	No	-11.3	5.4	7.16	NormalWeight
No	1	No	-5.4	4.2	7.331	UnderWeight
Yes	1	No	0	2.8	7.43	NormalWeight
No	1	No	-6.8	9.6	7.169	PreObese
No	1	No	0.2	2.5	7.3	UnderWeight
No	1	No	-1.9	3.3	7.327	ObeseClass1
No	1	No	-3.8	4.8	7.206	NormalWeight
No	1	No	-6.1	4.8	7.284	NormalWeight
No	1	No	-6.7	7.2	7.085	UnderWeight
No	1	No	4.8	5	7.193	UnderWeight
No	1	No	-2.4	4.2	7.254	NormalWeight
Yes	1	No	-1.6	2	7.35	PreObese
No	1	No	-1.9	3.3	7.363	NormalWeight
No	1	No	-1.1	3.4	7.331	NormalWeight
Yes	1	No	-1.8	3.6	7.264	PreObese
No	1	No	-0.5	2	7.372	UnderWeight
No	1	No	-0.8	2.6	7.3	NormalWeight

No	1	No	-5.1	4.5	7.334	UnderWeight
No	1	No	-4.2	3.6	7.344	PreObese
Yes	1	No	-4.2	4.3	7.344	PreObese
No	1	No	1.3	2.6	7.302	PreObese
No	1	No	1.8	1.8	7.314	NormalWeight
Yes	1	No	4.6	6.7	7.19	NormalWeight
No	1	No	0.2	4	7.232	NormalWeight
No	1	No	-0.2	3.9	7.278	PreObese
No	1	No	-3.4	3.9	7.346	ObeseClass1
No	1	No	-2.8	7.5	7.215	PreObese
No	1	No	-10.6	6.6	7.27	PreObese
No	1	No	0.8	2.4	7.279	ObeseClass3
No	1	No	-3.8	8.3	7.273	ObeseClass2
No	1	No	11.6	11	7.12	PreObese
No	1	No	-4	4.9	7.357	NormalWeight
No	1	No	0.8	4	7.244	PreObese
No	1	No	3.7	7.1	7.21	UnderWeight
No	1	No	9.8	7.7	7.093	ObeseClass1
No	1	No	-1.6	6.5	7.31	NormalWeight
No	1	No	-0.3	2.7	7.268	ObeseClass1
No	1	No	3	3.5	7.27	NormalWeight
No	1	No	0	3.5	7.287	PreObese
Yes	1	No	0.3	3.3	7.29	NormalWeight
No	1	No	1.5	1.9	7.28	NormalWeight
No	1	No	-1.5	5.7	7.158	NormalWeight
No	1	No	-7.2	8	7.071	ObeseClass3
No	1	No	-5.7	5	7.38	PreObese
No	1	No	-6.2	6.1	7.143	PreObese
No	1	No	1.6	3	7.26	ObeseClass1
No	1	No	1	2.3	7.308	PreObese
No	1	No	3.2	2	7.34	ObeseClass2
No	1	No	0.6	3.5	7.297	NormalWeight
No	1	No	-0.1	5.1	7.3	ObeseClass2
No	1	No	-0.3	3	7.287	NormalWeight
No	1	No	-0.6	2.5	7.29	NormalWeight
Yes	1	No	-0.4	2.7	7.448	NormalWeight
No	1	No	-0.8	2.4	7.305	NormalWeight
No	1	No	-2.9	3.6	7.363	NormalWeight
No	1	No	-4.5	6.3	7.159	ObeseClass1
No	1	No	-2.2	5.3	7.24	ObeseClass1
No	1	No	-4.7	3.2	7.322	PreObese
No	1	No	-0.8	4	7.187	UnderWeight
No	1	No	7.9	8.1	7.18	ObeseClass3
No	1	No	-1.4	4.1	7.172	ObeseClass2
No	1	No	0.3	2.4	7.3	UnderWeight
No	1	No	-2.6	2.5	7.349	ObeseClass1
No	1	No	-0.6	2.9	7.287	NormalWeight
No	1	No	-1.5	2.8	7.309	NormalWeight

No	1	No	-5.4	4.8	7.31	PreObese
No	1	No	-2.9	3.4	7.27	PreObese
No	1	No	0.9	3.4	7.284	PreObese
No	1	No	1.9	4.1	7.24	PreObese
No	1	No	0.5	2.7	7.302	UnderWeight
No	1	No	-1.1	4.8	7.286	NormalWeight
No	1	No	1.5	4	7.272	PreObese
No	1	No	-2.5	4.5	7.258	UnderWeight
No	1	No	-1.8	2.6	7.33	UnderWeight
Yes	1	No	-0.2	4.1	7.28	ObeseClass2
No	1	No	-5.6	3.7	7.276	ObeseClass1
No	1	No	-3.6	2.6	7.261	NormalWeight
No	1	No	-3.3	6.1	7.161	PreObese
No	1	No	2.4	3.3	7.297	ObeseClass1
No	1	No	-3.2	5.4	7.272	NormalWeight
No	1	No	-3.1	2.8	7.33	NormalWeight
No	1	No	-0.8	1.6	7.27	PreObese
Yes	1	No	2.6	3.3	7.284	NormalWeight
No	1	No	-0.1	3.3	7.24	ObeseClass2
No	1	No	2.4	3.1	7.23	ObeseClass1
No	1	No	-0.8	3.2	7.358	NormalWeight
No	1	No	7.3	4.4	7.25	NormalWeight
Yes	1	No	-5.7	4.6	7.269	ObeseClass2
No	1	No	-1.3	2.1	7.388	NormalWeight
No	1	No	2.9	3.6	7.29	NormalWeight
No	1	Other	2.1	2.8	7.24	ObeseClass3
No	1	No	-1.2	2	7.3	NormalWeight
Yes	1	No	1.2	2.3	7.314	ObeseClass2
No	1	No	6.7	7.6	7.15	PreObese
No	1	No	5	3.8	7.358	ObeseClass1
No	1	No	-5.8	6.5	7.28	PreObese
No	1	No	1.9	5.1	7.21	ObeseClass1
No	1	No	-4.4	3.9	7.293	UnderWeight
No	1	No	-1.5	2	7.3	PreObese
No	1	No	-2.8	3.6	7.19	ObeseClass1
No	1	No	8.7	8.5	7.31	ObeseClass1
No	1	No	-3.2	2.6	7.287	NormalWeight
No	1	No	-1.2	3.5	7.36	ObeseClass2
No	1	No	2.1	2.1	7.355	NormalWeight
No	1	No	-4	4.9	7.17	PreObese
No	1	No	4.8	6.1	7.193	NormalWeight
No	1	No	-4.1	3.2	7.28	NormalWeight
No	1	No	-2.4	3	7.284	NormalWeight
No	1	No	-2.7	8.1	7.17	NormalWeight
No	1	No	-0.9	2.5	7.387	PreObese
No	1	No	-3.5	2.7	7.345	PreObese
No	1	No	-3.6	2.1	7.296	NormalWeight
No	1	No	-6.5	5.1	7.25	NormalWeight

No	1	No	-2.9	4.2	7.21 NormalWeight
No	1	No	0	2.2	7.31 NormalWeight
No	1	No	-1.6	4.3	7.323 ObeseClass1
No	1	No	2.5	3.3	7.39 PreObese
Yes	1	No	-3.7	3.8	7.38 NormalWeight
No	1	No	-2.1	5.9	7.22 NormalWeight
No	1	No	6	6.9	7.15 UnderWeight
No	1	No	5.5	3.6	7.46 PreObese
No	1	No	2.8	2	7.27 PreObese
No	1	No	-4.2	0.8	7.365 ObeseClass1
No	1	No	-5.4	6.6	7.21 PreObese
No	1	No	-5.6	6.4	7.245 PreObese
Yes	1	No	-7.2	4.2	7.27 NormalWeight
No	1	No	-1.3	2.8	7.25 NormalWeight
No	1	No	-0.3	3.6	7.216 ObeseClass1
No	1	No	-4.6	6.6	7.265 PreObese
No	1	No	5.7	8.3	7.1 PreObese
No	1	No	-6.7	6.4	7.26 NormalWeight
Yes	1	No	4.8	6	7.25 PreObese
No	1	No	0.8	3.1	7.35 PreObese
No	1	No	-4.3	5	7.228 UnderWeight
No	1	No	1.6	1.1	7.39 ObeseClass1
No	1	No	-3.4	4.7	7.22 NormalWeight
No	1	No	-0.8	2.4	7.39 PreObese
No	1	No	-3	4.2	7.35 NormalWeight
No	1	No	8.9	5.5	7.3 NormalWeight
Yes	1	No	-2.2	2.3	7.355 ObeseClass2
Yes	1	No	-0.5	3.2	7.22 NormalWeight
No	1	No	-1.5	4.7	7.241 PreObese
No	1	No	4.9	3	7.356 UnderWeight
Yes	1	No	1.4	2.8	7.27 NormalWeight
Yes	1	No	0.3	2.4	7.31 NormalWeight
No	2	No	2.5	3.3	7.28 NormalWeight
Yes	2	Other	-1.2	5.7	7.344 ObeseClass1
No	2	No	-3.5	2.9	7.3 ObeseClass2
Yes	2	No	2.4	3.7	7.209 ObeseClass1
No	2	No	-0.8	3.9	7.24 NormalWeight
No	2	No	-3.4	3.9	7.34 PreObese
No	2	No	-1.1	3.2	7.332 ObeseClass2
No	2	No	0.9	2.9	7.305 PreObese
No	2	No	1.8	3	7.29 NormalWeight
No	2	No	-1.9	4.8	7.268 ObeseClass3
No	2	No	5.8	5.5	7.418 NormalWeight
No	2	No	0.3	2.8	7.312 PreObese
Yes	2	No	5.3	5	7.348 PreObese
No	2	No	1.9	3.2	7.33 UnderWeight
No	2	No	0.2	2.6	7.266 PreObese
Yes	2	No	-1.7	2.9	7.313 NormalWeight

No	2	No	-3.2	3.8	7.387 ObeseClass1
Yes	2	No	-0.2	2.5	7.279 PreObese
Yes	2	No	0.7	3.2	7.311 NormalWeight
No	2	No	-1.1	3	7.302 ObeseClass1
Yes	2	No	-1.7	2.4	7.342 PreObese
Yes	2	No	-3.6	3.4	7.25 NormalWeight
No	2	No	10.7	0.9	7.5 PreObese
No	2	No	-8	7.5	7.24 NormalWeight
Yes	2	No	-1.1	3.3	7.245 NormalWeight
Yes	2	No	-5.1	7	7.12 PreObese
No	2	No	-0.5	2.5	7.39 ObeseClass2
Yes	2	No	-2	5.2	7.18 NormalWeight
No	2	No	-2.7	4.6	7.23 ObeseClass2
No	2	No	0.4	2.5	7.2 NormalWeight
No	2	No	-1.3	4.3	7.2 ObeseClass1
No	2	No	-5.1	2.8	7.336 NormalWeight
No	2	No	-2.7	3.9	7.29 PreObese
No	2	No	-1.1	2.4	7.38 ObeseClass1
Yes	2	No	-0.3	3.1	7.34 NormalWeight
No	2	Other	-1.3	3.7	7.308 NormalWeight
Yes	2	No	-1.1	2.5	7.313 NormalWeight
No	2	No	-0.6	4.5	7.262 ObeseClass1
No	2	No	2.4	3.1	7.252 NormalWeight
Yes	2	No	4.3	4.6	7.247 NormalWeight
Yes	2	No	-1.7	6.9	7.167 NormalWeight
Yes	2	No	-4.7	6.7	7.21 NormalWeight
No	2	No	1.2	2.3	7.322 ObeseClass3
Yes	2	No	1.1	5.2	7.221 PreObese
No	2	No	1	3.5	7.224 ObeseClass1
No	2	No	0	3.3	7.317 ObeseClass2
Yes	2	No	-7.2	6	7.268 ObeseClass1
No	2	No	2.6	4.4	7.22 NormalWeight
No	2	No	-4.4	5.3	7.195 NormalWeight
No	2	Other	-4.7	5.2	7.245 NormalWeight
No	2	No	-4.2	4.8	7.241 PreObese
No	2	No	3.2	6.9	7.17 PreObese
Yes	2	No	-4.5	5	7.33 NormalWeight
No	2	No	0.3	2.3	7.4 ObeseClass2
Yes	2	No	0.5	4.2	7.3 NormalWeight
No	2	No	-3.2	3.3	7.34 NormalWeight
No	2	No	1.9	3	7.3 UnderWeight
No	2	No	2.5	4.5	7.396 UnderWeight
No	2	No	-8.7	7.2	7.12 UnderWeight
No	2	No	-4.8	3.7	7.366 NormalWeight
No	2	No	1	3.1	7.25 ObeseClass1
No	2	No	-4.7	3.3	7.411 ObeseClass1
Yes	2	No	-4.4	2.9	7.35 NormalWeight
Yes	2	No	3.8	0.6	7.29 NormalWeight

No	2	No	7.1	6.1	7.17	PreObese
No	2	No	-5.9	4.7	7.337	UnderWeight
Yes	2	No	-1.5	2.7	7.303	NormalWeight
No	2	No	-1.3	1.2	7.207	ObeseClass1
No	3	No	-1	2.4	7.284	NormalWeight
No	3	No	-3.2	2.2	7.3	ObeseClass2
No	3	No	-1.2	2.7	7.33	ObeseClass2
No	3	No	-3.1	2.1	7.35	NormalWeight
Yes	3	No	1.2	3	7.323	NormalWeight
No	3	No	1.3	4.7	7.28	NormalWeight
Yes	3	No	-2.5	4.1	7.255	PreObese
Yes	3	No	2.2	2.4	7.334	NormalWeight
No	3	No	-3.1	6.4	7.168	ObeseClass3
No	3	No	2.9	6.5	7.11	NormalWeight
No	3	No	-2.5	1.8	7.376	ObeseClass2
No	3	No	6	8.4	7.2	PreObese
No	3	No	-4.6	5.1	7.186	NormalWeight
No	3	No	-2.4	2	7.28	ObeseClass2
No	3	Other	1.1	2.9	7.26	NormalWeight
Yes	3	No	-2.5	4.4	7.209	ObeseClass1
Yes	3	No	1.2	3.8	7.292	UnderWeight
Yes	3	No	2.1	4	7.208	ObeseClass3
No	3	No	1.6	2.8	7.311	NormalWeight
Yes	3	No	-2	3.1	7.28	UnderWeight
Yes	3	No	-6.8	5.5	7.22	NormalWeight
Yes	3	Other	2.2	3.6	7.22	NormalWeight
Yes	3	No	0.5	2.1	7.286	PreObese
No	3	No	-5	4.9	7.25	ObeseClass1
No	3	No	-1.4	2.7	7.38	NormalWeight
No	3	No	-5.7	4.8	7.203	NormalWeight
No	3	No	1	3.4	7.26	PreObese
No	3	No	1.3	2	7.37	ObeseClass1
Yes	3	No	2.2	3.4	7.36	ObeseClass1
Yes	3	No	1.4	2.7	7.316	PreObese
Yes	3	No	-3.8	4.2	7.401	NormalWeight
Yes	3	No	-1.9	2.1	7.41	ObeseClass2
No	3	No	2.9	5.5	7.12	NormalWeight
Yes	3	No	1	3.4	7.29	PreObese
Yes	3	No	5.2	5.1	7.39	UnderWeight
No	4	No	2.4	2.3	7.35	PreObese
No	4	No	-5.9	5.3	7.16	ObeseClass3
Yes	4	No	-3.8	3.6	7.284	ObeseClass2
No	4	No	-2.4	4.9	7.188	ObeseClass2
No	4	No	-6.5	6.2	7.37	NormalWeight
No	4	No	-7.1	6.9	7.196	PreObese
No	4	No	0.1	3.5	7.333	ObeseClass1
No	4	No	-7.1	8.4	7.156	UnderWeight
Yes	4	No	-3	4.5	7.27	UnderWeight

No	4	No	6.2	2.1	7.35	PreObese
Yes	4	No	-0.2	4.1	7.272	ObeseClass1
Yes	4	No	2.2	1.9	7.294	ObeseClass1
No	4	No	1.6	2.4	7.279	NormalWeight
Yes	4	No	-5.4	7.4	7.151	NormalWeight
No	5	No	-5.8	3.6	7.41	ObeseClass2
Yes	5	No	-1.9	2.7	7.32	ObeseClass2
No	5	No	-3.2	5.8	7.278	PreObese
No	5	No	1.6	5	7.23	PreObese
Yes	5	No	-6.6	6.1	7.135	NormalWeight
Yes	5	No	2.9	0.4	7.39	UnderWeight
Yes	6	No	-2.3	3.3	7.3	NormalWeight
Yes	9	No	-1.8	3.2	7.33	NormalWeight
Yes	11	No	-6.7	3.5	7.25	ObeseClass1
Yes	12	No	-4.5	3.9	7.316	NormalWeight

diabetesCate	feedingDisch	gestationalA	illegalDrugU	maternalAge	modeBirthCa	parity
No	breastMilk	40.1428571	No	LessThan35	VaginalBirth	3
No	breastMilk	40.8571429	No	LessThan35	VaginalBirth	0
No	breastMilk	41.4285714	No	LessThan35	VaginalBirth	MoreThan3
No	breastMilk	40	No	LessThan35	VaginalBirth	2
No	breastMilk	39.1428571	No	LessThan35	VaginalBirth	0
No	breastMilk	38.4285714	No	MoreThan35	VaginalBirth	2
No	breastMilk	40	No	LessThan35	VaginalBirth	3
No	nonBreastMi	39.4285714	No	LessThan35	CSection	1
No	nonBreastMi	39	No	LessThan35	CSection	1
No	breastMilk	40.4285714	No	LessThan35	VaginalBirth	1
No	breastMilk	37.8571429	No	LessThan35	VaginalBirth	2
No	breastMilk	40	No	LessThan35	VaginalBirth	1
No	breastMilk	39.1428571	No	MoreThan35	VaginalBirth	MoreThan3
No	breastMilk	39.5714286	No	LessThan35	VaginalBirth	2
No	nonBreastMi	39.4285714	No	MoreThan35	VaginalBirth	MoreThan3
No	nonBreastMi	39.5714286	No	MoreThan35	CSection	0
No	nonBreastMi	37.5714286	No	LessThan35	CSection	1
No	breastMilk	38.7142857	No	LessThan35	CSection	1
No	nonBreastMi	41.1428571	No	LessThan35	VaginalBirth	2
No	nonBreastMi	41.4285714	No	LessThan35	CSection	0
No	breastMilk	37.4285714	No	LessThan35	CSection	3
No	breastMilk	40.8571429	No	LessThan35	CSection	0
No	breastMilk	41	No	LessThan35	VaginalBirth	1
GestationalD	breastMilk	38.7142857	No	LessThan35	CSection	2
No	nonBreastMi	41.5714286	No	LessThan35	VaginalBirth	1
No	breastMilk	36.4285714	No	LessThan35	VaginalBirth	1
No	breastMilk	39.1428571	No	LessThan20	CSection	0
GestationalD	breastMilk	39.1428571	No	LessThan35	CSection	1
No	breastMilk	37	No	MoreThan35	CSection	2
No	breastMilk	37	No	MoreThan35	CSection	2
No	breastMilk	40.1428571	No	LessThan20	VaginalBirth	0
No	breastMilk	35.2857143	No	LessThan35	CSection	0
No	breastMilk	37.1428571	No	LessThan35	CSection	0
No	breastMilk	41.1428571	No	LessThan35	CSection	1
GestationalD	breastMilk	35.1428571	No	LessThan35	CSection	0
No	breastMilk	41.2857143	No	LessThan35	VaginalBirth	2
GestationalD	nonBreastMi	39	Yes	LessThan35	VaginalBirth	0
No	breastMilk	41.4285714	No	LessThan35	VaginalBirth	2
GestationalD	breastMilk	38	No	LessThan35	CSection	1
No	breastMilk	38.7142857	No	MoreThan35	VaginalBirth	0
No	breastMilk	37.8571429	No	LessThan35	VaginalBirth	1
No	breastMilk	39.7142857	No	LessThan35	VaginalBirth	2
No	nonBreastMi	39.2857143	No	LessThan35	CSection	0
No	breastMilk	39.7142857	No	LessThan35	CSection	0
No	nonBreastMi	39.5714286	No	LessThan35	CSection	0
No	breastMilk	40	No	MoreThan35	CSection	1
No	breastMilk	37.8571429	No	MoreThan35	VaginalBirth	1

No	breastMilk	36	No		MoreThan35	CSection	MoreThan3
No	breastMilk	39.7142857	No		LessThan35	VaginalBirths	1
No	nonBreastMi	41.1428571	No		LessThan35	VaginalBirths	2
No	nonBreastMi	39.5714286	No		LessThan20	CSection	0
No	nonBreastMi	39.4285714	Yes		LessThan35	VaginalBirths	0
No	nonBreastMi	39	No		LessThan35	CSection	0
No	breastMilk	38.7142857	No		LessThan35	VaginalBirths	2
No	breastMilk	38.4285714	No		LessThan35	VaginalBirths	0
No	breastMilk	38	No		LessThan35	VaginalBirths	0
No	nonBreastMi	41.5714286	No		LessThan35	VaginalBirths	0
No	breastMilk	37.8571429	No		LessThan35	VaginalBirths	0
No	breastMilk	39.5714286	No		LessThan35	VaginalBirths	1
GestationalD	breastMilk	39.4285714	No		LessThan35	VaginalBirths	0
No	breastMilk	37.7142857	No		LessThan35	VaginalBirths	1
No	breastMilk	39	No		LessThan35	CSection	1
No	breastMilk	39.4285714	No		LessThan35	VaginalBirths	0
No	nonBreastMi	39.7142857	No		LessThan35	VaginalBirths	1
No	breastMilk	38.7142857	No		MoreThan35	CSection	1
No	breastMilk	36.2857143	No		LessThan35	CSection	1
No	breastMilk	38	No		LessThan35	VaginalBirths	0
No	breastMilk	40.7142857	Yes		LessThan20	VaginalBirths	1
No	breastMilk	39.1428571	No		MoreThan35	CSection	1
No	breastMilk	39	No		LessThan35	VaginalBirths	1
No	breastMilk	40.2857143	No		LessThan35	VaginalBirths	1
No	breastMilk	40	No		LessThan35	VaginalBirths	3
No	breastMilk	40.2857143	No		LessThan35	VaginalBirths	0
GestationalD	nonBreastMi	37.7142857	No		LessThan35	VaginalBirths	0
No	breastMilk	38.2857143	No		LessThan35	VaginalBirths	0
No	nonBreastMi	39.1428571	No		MoreThan35	CSection	3
No	breastMilk	38.4285714	No		LessThan35	CSection	3
No	breastMilk	40	No		LessThan35	VaginalBirths	0
PreExisting	breastMilk	36.4285714	No		MoreThan35	CSection	MoreThan3
No	breastMilk	40.8571429	No		LessThan35	VaginalBirths	1
No	breastMilk	37.1428571	No		LessThan35	VaginalBirths	1
No	nonBreastMi	41.4285714	No		LessThan35	VaginalBirths	0
No	breastMilk	41.5714286	No		MoreThan35	VaginalBirths	0
No	breastMilk	40.4285714	No		MoreThan35	CSection	1
No	breastMilk	40.1428571	No		LessThan35	CSection	1
No	breastMilk	39.5714286	No		LessThan35	VaginalBirths	0
No	breastMilk	40.4285714	No		LessThan35	VaginalBirths	2
No	nonBreastMi	37.8571429	No		MoreThan35	VaginalBirths	0
No	breastMilk	38.8571429	No		LessThan35	CSection	1
No	breastMilk	39.8571429	No		LessThan35	VaginalBirths	3
PreExisting	breastMilk	37	No		LessThan35	CSection	0
No	breastMilk	41.2857143	No		LessThan35	VaginalBirths	0
No	breastMilk	41.4285714	No		MoreThan35	CSection	0
No	breastMilk	40.1428571	No		LessThan35	CSection	0
GestationalD	breastMilk	40.5714286	No		LessThan35	VaginalBirths	1

No	breastMilk	38.1428571	No	LessThan35	CSection	0
No	breastMilk	40.4285714	No	LessThan35	CSection	0
No	breastMilk	39.8571429	No	LessThan20	VaginalBirths	0
No	breastMilk	38.1428571	No	LessThan20	VaginalBirths	0
No	breastMilk	40.8571429	No	LessThan35	CSection	0
No	breastMilk	40.4285714	No	LessThan35	VaginalBirths	0
No	breastMilk	41	No	LessThan35	CSection	0
No	breastMilk	39.5714286	No	LessThan35	VaginalBirths	1
No	breastMilk	39	No	MoreThan35	VaginalBirths	2
No	breastMilk	38.4285714	No	LessThan35	VaginalBirths	0
No	breastMilk	41.8571429	No	LessThan35	VaginalBirths	1
No	breastMilk	39.1428571	No	LessThan35	VaginalBirths	0
No	breastMilk	39.2857143	No	LessThan35	CSection	1
No	breastMilk	38.8571429	No	LessThan35	VaginalBirths	1
No	breastMilk	40	No	LessThan35	CSection	0
No	breastMilk	38.4285714	No	LessThan35	CSection	MoreThan3
No	breastMilk	40.5714286	No	MoreThan35	VaginalBirths	2
No	breastMilk	39.4285714	No	LessThan35	CSection	1
No	nonBreastMi	38.4285714	No	LessThan35	VaginalBirths	MoreThan3
No	nonBreastMi	38.5714286	No	MoreThan35	CSection	1
No	breastMilk	41	No	LessThan35	CSection	0
No	breastMilk	40.8571429	No	LessThan35	VaginalBirths	2
No	breastMilk	41	No	LessThan20	VaginalBirths	0
No	breastMilk	41.4285714	No	LessThan35	CSection	0
No	breastMilk	41.4285714	No	LessThan35	CSection	1
No	breastMilk	38.5714286	No	LessThan35	CSection	1
No	nonBreastMi	41.1428571	No	LessThan20	VaginalBirths	0
No	breastMilk	38.4285714	No	LessThan35	VaginalBirths	0
No	breastMilk	36	No	LessThan35	CSection	1
No	nonBreastMi	38.5714286	No	LessThan35	VaginalBirths	0
No	breastMilk	41.2857143	No	LessThan35	VaginalBirths	0
No	breastMilk	40	No	MoreThan35	VaginalBirths	2
No	breastMilk	32.2857143	No	MoreThan35	CSection	1
No	breastMilk	39.2857143	No	LessThan35	CSection	1
No	breastMilk	39.1428571	No	LessThan35	CSection	1
No	breastMilk	39	No	LessThan35	VaginalBirths	0
No	nonBreastMi	39.1428571	No	LessThan35	CSection	1
No	breastMilk	41.4285714	No	LessThan35	VaginalBirths	1
No	breastMilk	39.8571429	No	LessThan35	CSection	0
No	nonBreastMi	38.1428571	No	LessThan20	VaginalBirths	1
No	breastMilk	40.5714286	No	LessThan35	VaginalBirths	1
No	nonBreastMi	39	No	LessThan35	VaginalBirths	0
No	breastMilk	39	No	LessThan35	VaginalBirths	0
No	nonBreastMi	40.1428571	No	LessThan35	CSection	0
No	nonBreastMi	38.1428571	No	LessThan35	VaginalBirths	2
No	breastMilk	40	No	LessThan35	VaginalBirths	0
No	breastMilk	39	No	MoreThan35	CSection	2
GestationalD	nonBreastMi	38.2857143	No	MoreThan35	VaginalBirths	2

No	breastMilk	39	No	LessThan20	VaginalBirths	0
No	breastMilk	39.4285714	Yes	MoreThan35	CSection	2
No	breastMilk	41.4285714	No	LessThan35	CSection	0
No	nonBreastMi	39.8571429	No	LessThan20	VaginalBirths	1
No	breastMilk	39.1428571	No	LessThan35	VaginalBirths	2
No	breastMilk	39.8571429	No	LessThan35	VaginalBirths	0
No	nonBreastMi	39.8571429	No	LessThan35	VaginalBirths	1
No	breastMilk	40.1428571	No	LessThan35	CSection	0
No	breastMilk	36.1428571	No	LessThan35	VaginalBirths	0
No	breastMilk	39.2857143	No	LessThan35	VaginalBirths	3
No	breastMilk	41.4285714	Yes	LessThan35	CSection	0
No	breastMilk	39.1428571	No	LessThan35	CSection	1
No	breastMilk	40.5714286	No	LessThan35	CSection	1
No	breastMilk	40.8571429	No	LessThan35	VaginalBirths	0
No	breastMilk	40.1428571	No	LessThan35	CSection	0
No	breastMilk	38.2857143	No	LessThan35	CSection	2
No	nonBreastMi	33.1428571	No	LessThan35	VaginalBirths	0
No	breastMilk	37.1428571	No	LessThan35	VaginalBirths	1
No	breastMilk	38.8571429	No	LessThan35	VaginalBirths	1
No	breastMilk	40.4285714	No	LessThan20	CSection	0
No	nonBreastMi	37.8571429	No	LessThan35	CSection	2
No	breastMilk	40.1428571	No	MoreThan35	VaginalBirths	2
GestationalD	breastMilk	38.5714286	No	MoreThan35	VaginalBirths	0
No	breastMilk	38.7142857	No	LessThan35	VaginalBirths	0
No	breastMilk	39	No	LessThan20	VaginalBirths	0
No	breastMilk	40.2857143	No	LessThan35	CSection	1
No	breastMilk	39.2857143	No	LessThan35	VaginalBirths	0
No	breastMilk	40.7142857	No	LessThan35	VaginalBirths	2
No	breastMilk	39.4285714	No	LessThan35	VaginalBirths	0
No	breastMilk	41.7142857	No	LessThan35	CSection	0
No	breastMilk	38.8571429	No	LessThan35	CSection	3
No	breastMilk	39.2857143	No	LessThan35	CSection	1
GestationalD	breastMilk	38.2857143	No	LessThan35	CSection	0
No	nonBreastMi	40.7142857	No	LessThan35	VaginalBirths	1
No	breastMilk	40.7142857	No	LessThan35	VaginalBirths	0
No	nonBreastMi	38.8571429	No	LessThan35	VaginalBirths	2
No	breastMilk	40.7142857	No	LessThan35	VaginalBirths	1
No	breastMilk	40.5714286	No	LessThan35	VaginalBirths	0
No	nonBreastMi	38.1428571	No	MoreThan35	CSection	2
No	breastMilk	40	No	LessThan35	VaginalBirths	3
No	breastMilk	38.4285714	No	LessThan35	CSection	0
No	breastMilk	41.1428571	No	LessThan35	CSection	0
No	breastMilk	34.4285714	No	LessThan35	CSection	0
No	breastMilk	37.5714286	No	LessThan35	CSection	1
No	nonBreastMi	37	No	LessThan35	CSection	1
No	nonBreastMi	39.1428571	No	MoreThan35	CSection	2
No	breastMilk	38.1428571	Yes	MoreThan35	VaginalBirths	1
No	breastMilk	39.1428571	No	MoreThan35	VaginalBirths	2

No	nonBreastMi	40.2857143	No	LessThan20	VaginalBirths	0
No	breastMilk	40.8571429	No	LessThan35	VaginalBirths	0
No	breastMilk	41.2857143	No	LessThan20	VaginalBirths	0
No	nonBreastMi	41.1428571	No	LessThan20	CSection	0
No	nonBreastMi	39.4285714	No	LessThan35	VaginalBirths	0
No	breastMilk	38.7142857	No	LessThan35	VaginalBirths	2
No	nonBreastMi	39	No	LessThan35	CSection	1
No	breastMilk	39	No	LessThan35	VaginalBirths	0
No	breastMilk	40	No	LessThan35	CSection	1
No	nonBreastMi	39.4285714	Yes	LessThan35	VaginalBirths	0
No	nonBreastMi	41.5714286	No	LessThan35	VaginalBirths	3
No	nonBreastMi	40.1428571	No	LessThan20	VaginalBirths	1
No	breastMilk	40.5714286	No	LessThan35	VaginalBirths	1
No	breastMilk	38.1428571	No	LessThan35	VaginalBirths	0
No	breastMilk	34.8571429	Yes	LessThan35	VaginalBirths	0
GestationalD	breastMilk	39.1428571	No	LessThan35	CSection	0
No	breastMilk	40.1428571	No	LessThan35	VaginalBirths	0
No	breastMilk	41.4285714	No	LessThan35	CSection	0
No	breastMilk	38.4285714	No	LessThan35	CSection	0
GestationalD	breastMilk	38.7142857	No	LessThan35	VaginalBirths	1
No	breastMilk	37.5714286	No	MoreThan35	CSection	MoreThan3
No	breastMilk	39.2857143	No	LessThan35	CSection	0
No	nonBreastMi	39.4285714	No	LessThan35	CSection	3
No	breastMilk	41.5714286	No	LessThan35	VaginalBirths	1
No	breastMilk	38.2857143	No	MoreThan35	CSection	3
No	breastMilk	41.2857143	No	LessThan35	VaginalBirths	1
GestationalD	breastMilk	35	Yes	LessThan35	VaginalBirths	2
No	breastMilk	41.2857143	No	LessThan20	CSection	0
No	breastMilk	40.1428571	No	LessThan35	CSection	1
No	nonBreastMi	37	No	LessThan20	CSection	0
No	nonBreastMi	40.2857143	No	LessThan20	VaginalBirths	0
No	breastMilk	40.1428571	No	LessThan35	VaginalBirths	1
No	breastMilk	38	No	LessThan35	CSection	1
No	nonBreastMi	38	No	LessThan35	VaginalBirths	2
GestationalD	breastMilk	38.2857143	No	LessThan35	CSection	1
No	breastMilk	41.5714286	No	LessThan35	VaginalBirths	MoreThan3
No	breastMilk	36.4285714	No	MoreThan35	VaginalBirths	0
No	breastMilk	40.2857143	No	LessThan35	CSection	0
No	breastMilk	39.5714286	No	LessThan35	CSection	2
No	breastMilk	39.2857143	No	LessThan35	VaginalBirths	1
No	breastMilk	38.7142857	No	LessThan35	VaginalBirths	0
GestationalD	nonBreastMi	36.7142857	No	LessThan35	VaginalBirths	0
PreExisting	breastMilk	39	No	LessThan35	VaginalBirths	0
No	nonBreastMi	41.7142857	No	LessThan35	VaginalBirths	0
No	breastMilk	40.5714286	No	LessThan35	VaginalBirths	0
No	breastMilk	38.1428571	No	LessThan35	CSection	1
No	breastMilk	40.4285714	No	LessThan35	VaginalBirths	2
No	breastMilk	35.7142857	No	MoreThan35	VaginalBirths	MoreThan3

No	breastMilk	38.5714286	No	LessThan35	VaginalBirths	1
No	breastMilk	41.4285714	No	LessThan35	CSection	0
GestationalD	breastMilk	38.1428571	No	LessThan35	VaginalBirths	1
No	breastMilk	39	No	LessThan35	CSection	0
No	nonBreastMi	41.4285714	No	LessThan35	VaginalBirths	1
No	breastMilk	38.4285714	No	LessThan35	VaginalBirths	1
No	breastMilk	40.2857143	No	LessThan35	CSection	MoreThan3
No	nonBreastMi	35.8571429	No	LessThan35	VaginalBirths	0
No	breastMilk	39.4285714	No	LessThan35	VaginalBirths	1
No	breastMilk	38.4285714	No	MoreThan35	VaginalBirths	3
No	nonBreastMi	40	No	MoreThan35	VaginalBirths	MoreThan3
GestationalD	breastMilk	40.1428571	No	LessThan35	VaginalBirths	0
No	breastMilk	39.5714286	No	LessThan35	VaginalBirths	2
No	breastMilk	41	No	LessThan35	VaginalBirths	0
No	breastMilk	41.8571429	No	LessThan35	VaginalBirths	0
No	breastMilk	40.2857143	No	LessThan35	VaginalBirths	MoreThan3
No	breastMilk	39	No	LessThan35	CSection	2
No	breastMilk	39.8571429	No	LessThan35	VaginalBirths	2
No	nonBreastMi	39.4285714	No	LessThan35	VaginalBirths	2
No	breastMilk	38.8571429	No	LessThan35	CSection	1
No	breastMilk	38.4285714	No	LessThan35	VaginalBirths	1
No	breastMilk	39.1428571	No	MoreThan35	VaginalBirths	2
No	nonBreastMi	40.8571429	No	LessThan35	CSection	0
GestationalD	breastMilk	39.1428571	No	LessThan35	VaginalBirths	0
No	breastMilk	40.2857143	No	LessThan35	VaginalBirths	2
No	breastMilk	41	No	LessThan35	VaginalBirths	0
No	breastMilk	40.2857143	No	LessThan35	VaginalBirths	3
No	breastMilk	39.2857143	No	LessThan35	VaginalBirths	2
No	breastMilk	38.1428571	No	LessThan35	CSection	3
No	nonBreastMi	36.7142857	No	LessThan35	VaginalBirths	2
No	breastMilk	39.1428571	No	LessThan35	VaginalBirths	0
No	breastMilk	38	No	LessThan35	VaginalBirths	3
No	breastMilk	33.1428571	No	LessThan35	CSection	0
No	breastMilk	39.1428571	No	LessThan35	CSection	0
No	breastMilk	41.8571429	No	LessThan35	VaginalBirths	0
No	nonBreastMi	39.4285714	No	LessThan35	CSection	0
No	breastMilk	40.1428571	No	LessThan35	VaginalBirths	0
No	breastMilk	39.8571429	No	LessThan20	VaginalBirths	0
No	nonBreastMi	34	No	LessThan35	CSection	2
No	breastMilk	38.5714286	Yes	LessThan20	CSection	0
No	breastMilk	40.5714286	No	LessThan35	CSection	2
No	breastMilk	39.2857143	No	LessThan35	VaginalBirths	3
No	breastMilk	38.5714286	No	LessThan35	VaginalBirths	0
No	breastMilk	39.8571429	No	LessThan35	VaginalBirths	0
No	nonBreastMi	39.2857143	No	LessThan35	VaginalBirths	2
No	breastMilk	39	No	LessThan35	CSection	1
No	breastMilk	37.2857143	Yes	LessThan35	CSection	1
No	breastMilk	41.2857143	No	LessThan35	VaginalBirths	0

GestationalD	breastMilk	37.4285714	No	LessThan35	CSection	0
No	nonBreastMi	39.2857143	No	LessThan35	VaginalBirths	1
No	breastMilk	38.7142857	No	MoreThan35	VaginalBirths	0
No	breastMilk	40.4285714	No	LessThan35	VaginalBirths	0
No	breastMilk	39.1428571	No	LessThan35	CSection	2
No	nonBreastMi	38.4285714	No	LessThan35	VaginalBirths	1
No	breastMilk	37.5714286	No	LessThan35	VaginalBirths	MoreThan3
No	breastMilk	34.5714286	No	LessThan35	CSection	MoreThan3
No	breastMilk	40.4285714	No	LessThan35	VaginalBirths	1
No	breastMilk	38.7142857	No	MoreThan35	VaginalBirths	2
No	breastMilk	40.7142857	No	LessThan35	VaginalBirths	1
No	nonBreastMi	39.4285714	No	LessThan35	CSection	2
GestationalD	breastMilk	36.8571429	No	MoreThan35	VaginalBirths	1
No	breastMilk	39.7142857	No	LessThan35	VaginalBirths	0
GestationalD	nonBreastMi	39.4285714	Yes	LessThan35	VaginalBirths	1
No	breastMilk	39.7142857	No	LessThan35	CSection	0
No	breastMilk	40.5714286	No	LessThan35	VaginalBirths	1
No	breastMilk	39.2857143	No	MoreThan35	CSection	1
No	breastMilk	41.5714286	No	LessThan35	CSection	0
No	breastMilk	41.1428571	No	LessThan35	VaginalBirths	3
No	nonBreastMi	40.4285714	No	LessThan35	CSection	0
No	breastMilk	41.5714286	No	LessThan35	VaginalBirths	1
No	breastMilk	38	No	LessThan35	CSection	1
No	breastMilk	40.4285714	No	LessThan35	VaginalBirths	0
No	nonBreastMi	39.5714286	No	LessThan35	VaginalBirths	1
No	breastMilk	41.5714286	No	LessThan35	VaginalBirths	0
No	nonBreastMi	41.7142857	No	LessThan35	CSection	0
No	breastMilk	38.2857143	No	LessThan35	CSection	0
No	breastMilk	41.2857143	No	MoreThan35	VaginalBirths	2
No	breastMilk	34	No	LessThan35	VaginalBirths	0
No	breastMilk	41	No	LessThan35	VaginalBirths	0
No	breastMilk	39.7142857	No	LessThan35	CSection	1
No	breastMilk	41.2857143	No	LessThan35	VaginalBirths	0
No	breastMilk	39	No	LessThan35	VaginalBirths	1
GestationalD	breastMilk	39.4285714	No	LessThan35	CSection	0
No	breastMilk	39.2857143	No	LessThan35	VaginalBirths	1
No	breastMilk	39.1428571	No	LessThan35	VaginalBirths	3
No	breastMilk	39.5714286	No	LessThan35	VaginalBirths	2
No	breastMilk	39.1428571	No	LessThan35	CSection	0
No	breastMilk	38.5714286	No	LessThan35	CSection	2
No	breastMilk	35.8571429	No	LessThan35	CSection	1
No	breastMilk	41	No	MoreThan35	CSection	0
No	breastMilk	41	No	LessThan20	VaginalBirths	0
No	breastMilk	38.1428571	No	LessThan35	CSection	2
No	breastMilk	40.7142857	No	LessThan35	VaginalBirths	0
No	breastMilk	40.2857143	No	LessThan35	VaginalBirths	0
No	breastMilk	39.2857143	No	LessThan35	VaginalBirths	1
No	breastMilk	38.4285714	No	LessThan35	CSection	0

No	breastMilk	40.8571429	No	LessThan35	VaginalBirths	1
No	breastMilk	41.5714286	No	LessThan35	VaginalBirths	1
No	breastMilk	39	No	LessThan35	VaginalBirths	0
No	nonBreastMi	38.7142857	No	LessThan35	CSection	1
No	nonBreastMi	40.7142857	No	LessThan35	VaginalBirths	1
No	breastMilk	40.5714286	No	LessThan35	VaginalBirths	0
No	breastMilk	39.8571429	No	LessThan35	VaginalBirths	1
No	nonBreastMi	39.7142857	No	LessThan35	VaginalBirths	1
No	nonBreastMi	36.1428571	No	LessThan35	VaginalBirths	0
No	breastMilk	41	No	LessThan35	CSection	1
No	nonBreastMi	39.5714286	No	LessThan35	CSection	2
No	breastMilk	41.2857143	No	LessThan20	VaginalBirths	0
No	breastMilk	38.2857143	No	MoreThan35	CSection	2
No	breastMilk	39	No	LessThan35	VaginalBirths	0
No	breastMilk	37.4285714	Yes	LessThan35	VaginalBirths	0
GestationalD	breastMilk	38.8571429	No	MoreThan35	CSection	1
No	breastMilk	39.2857143	No	LessThan35	CSection	2
No	breastMilk	41.4285714	No	LessThan35	VaginalBirths	1
No	breastMilk	39.4285714	No	LessThan35	VaginalBirths	0
No	breastMilk	40	No	LessThan35	CSection	1
No	breastMilk	38.5714286	No	LessThan35	CSection	1
No	breastMilk	39.7142857	No	LessThan35	VaginalBirths	0
No	breastMilk	36.8571429	Yes	LessThan35	VaginalBirths	1
No	nonBreastMi	38.5714286	No	LessThan35	CSection	2
No	nonBreastMi	37.5714286	No	LessThan35	VaginalBirths	1
No	nonBreastMi	39.8571429	No	LessThan35	VaginalBirths	1
No	breastMilk	40.2857143	No	MoreThan35	VaginalBirths	1
No	breastMilk	36.7142857	No	LessThan35	CSection	1
No	breastMilk	40.4285714	No	LessThan35	CSection	0
No	nonBreastMi	39.1428571	No	LessThan20	VaginalBirths	0
No	nonBreastMi	41.5714286	No	LessThan35	VaginalBirths	MoreThan3
No	breastMilk	36.7142857	No	LessThan35	CSection	1
No	nonBreastMi	39.5714286	No	LessThan35	VaginalBirths	0
GestationalD	breastMilk	39.7142857	No	LessThan35	VaginalBirths	1
No	breastMilk	39	No	LessThan35	CSection	0
No	breastMilk	38.5714286	No	LessThan35	VaginalBirths	0
No	breastMilk	41.4285714	No	LessThan35	VaginalBirths	0
No	breastMilk	39.4285714	No	LessThan35	VaginalBirths	0
No	breastMilk	36.1428571	No	MoreThan35	VaginalBirths	1
No	breastMilk	39.4285714	No	LessThan35	VaginalBirths	1
No	breastMilk	41	No	LessThan35	CSection	0
No	breastMilk	40.5714286	No	LessThan35	VaginalBirths	0
No	nonBreastMi	38.4285714	No	MoreThan35	CSection	3
No	breastMilk	37	No	LessThan35	CSection	0
GestationalD	nonBreastMi	38.8571429	No	MoreThan35	CSection	3
No	nonBreastMi	40.5714286	No	LessThan35	VaginalBirths	0
No	breastMilk	36.1428571	No	LessThan35	CSection	1
No	nonBreastMi	40	No	LessThan20	VaginalBirths	0

No	nonBreastMi	40	No	LessThan35	VaginalBirths	2
No	nonBreastMi	41.4285714	No	LessThan35	VaginalBirths	0
No	breastMilk	36.7142857	No	MoreThan35	CSection	2
No	breastMilk	40.1428571	No	LessThan20	VaginalBirths	1
No	breastMilk	41	No	LessThan20	VaginalBirths	0
No	breastMilk	38.8571429	No	LessThan20	VaginalBirths	0
No	nonBreastMi	37.4285714	No	LessThan35	VaginalBirths	0
No	nonBreastMi	38	No	LessThan35	VaginalBirths	1
No	breastMilk	39	No	LessThan35	VaginalBirths	1
No	nonBreastMi	40.5714286	No	LessThan35	CSection	0
No	breastMilk	38.7142857	No	LessThan35	VaginalBirths	0
No	breastMilk	39.5714286	No	LessThan35	VaginalBirths	0
No	nonBreastMi	34.7142857	No	LessThan35	VaginalBirths	0
No	breastMilk	39.5714286	No	LessThan35	VaginalBirths	0
No	breastMilk	36	No	MoreThan35	CSection	0
No	nonBreastMi	39.4285714	No	LessThan20	VaginalBirths	0
No	nonBreastMi	33.8571429	No	LessThan35	VaginalBirths	MoreThan3
No	breastMilk	39.5714286	No	LessThan35	VaginalBirths	2
No	breastMilk	38.2857143	No	LessThan35	VaginalBirths	2
No	nonBreastMi	40	No	LessThan35	CSection	2
No	breastMilk	40.4285714	No	LessThan20	CSection	0
No	breastMilk	40.7142857	No	LessThan35	CSection	0
No	breastMilk	36.8571429	No	LessThan35	VaginalBirths	2
No	breastMilk	34.7142857	No	LessThan35	CSection	2
No	breastMilk	39.1428571	No	LessThan35	VaginalBirths	2
No	breastMilk	40.7142857	No	LessThan35	VaginalBirths	1
No	breastMilk	37.8571429	No	LessThan35	VaginalBirths	2
No	nonBreastMi	38.2857143	No	LessThan35	CSection	2
No	breastMilk	37.1428571	No	LessThan35	VaginalBirths	1
No	breastMilk	40.5714286	No	LessThan35	VaginalBirths	1
No	breastMilk	38.8571429	No	MoreThan35	CSection	3
No	nonBreastMi	41.1428571	No	LessThan35	VaginalBirths	1
No	nonBreastMi	37.2857143	No	LessThan35	CSection	0
No	nonBreastMi	41.4285714	No	LessThan35	CSection	0
No	nonBreastMi	38.5714286	No	LessThan35	VaginalBirths	3
No	breastMilk	41.4285714	No	LessThan35	VaginalBirths	0
No	breastMilk	38.5714286	No	LessThan35	VaginalBirths	1
No	breastMilk	39.8571429	No	MoreThan35	VaginalBirths	2
No	nonBreastMi	39.4285714	No	LessThan35	VaginalBirths	1
PreExisting	breastMilk	38.2857143	No	LessThan35	VaginalBirths	1
No	breastMilk	41.4285714	No	LessThan35	VaginalBirths	1
No	nonBreastMi	34.7142857	No	LessThan35	VaginalBirths	0
No	breastMilk	39.5714286	No	LessThan35	VaginalBirths	0
GestationalD	breastMilk	39.1428571	No	MoreThan35	CSection	2
No	breastMilk	38.4285714	Yes	LessThan35	VaginalBirths	0
No	nonBreastMi	41	No	LessThan35	VaginalBirths	0
No	nonBreastMi	39	No	LessThan35	CSection	2
No	breastMilk	38.5714286	No	LessThan35	VaginalBirths	2

No	breastMilk	39.5714286	No	LessThan35	CSection	MoreThan3
No	breastMilk	39.8571429	No	LessThan35	CSection	1
No	breastMilk	39.5714286	No	LessThan35	VaginalBirths	1
No	nonBreastMi	39.5714286	No	LessThan35	VaginalBirths	0
No	breastMilk	40	No	LessThan35	VaginalBirths	3
No	nonBreastMi	40.8571429	No	LessThan35	VaginalBirths	1
No	breastMilk	39.2857143	No	LessThan35	VaginalBirths	0
No	breastMilk	40	No	LessThan35	CSection	0
No	breastMilk	39	No	LessThan35	VaginalBirths	2
No	breastMilk	38.2857143	No	LessThan35	VaginalBirths	1
No	breastMilk	39.5714286	No	LessThan35	VaginalBirths	0
No	nonBreastMi	38.5714286	No	LessThan35	VaginalBirths	0
GestationalD	breastMilk	38.4285714	No	MoreThan35	CSection	0
No	breastMilk	38.4285714	No	MoreThan35	VaginalBirths	2
No	breastMilk	38.7142857	No	LessThan35	CSection	0
No	breastMilk	40.5714286	No	LessThan35	CSection	0
No	breastMilk	41.1428571	No	LessThan35	VaginalBirths	2
No	breastMilk	40.1428571	No	LessThan35	CSection	0
No	breastMilk	38.8571429	No	LessThan35	VaginalBirths	0
GestationalD	breastMilk	35.4285714	No	MoreThan35	CSection	2
No	breastMilk	38.7142857	No	LessThan35	VaginalBirths	1
No	breastMilk	39.4285714	No	LessThan35	CSection	1
No	breastMilk	38.2857143	No	LessThan35	CSection	2
No	nonBreastMi	39	No	LessThan35	VaginalBirths	3
No	nonBreastMi	38.4285714	No	LessThan35	VaginalBirths	3
No	breastMilk	40	No	LessThan35	VaginalBirths	0
No	nonBreastMi	38.8571429	No	LessThan35	VaginalBirths	1
No	nonBreastMi	35.8571429	No	LessThan35	CSection	0
No	breastMilk	40.8571429	No	LessThan35	CSection	0
No	breastMilk	40.1428571	No	LessThan35	VaginalBirths	1
No	breastMilk	41.1428571	No	LessThan35	VaginalBirths	0
No	breastMilk	40	No	LessThan35	VaginalBirths	0
No	breastMilk	39.5714286	No	LessThan35	CSection	2
No	breastMilk	38.8571429	No	LessThan35	VaginalBirths	2
No	breastMilk	39.2857143	No	LessThan35	VaginalBirths	0
No	breastMilk	32.5714286	No	LessThan35	VaginalBirths	1
No	breastMilk	40.2857143	No	LessThan35	VaginalBirths	1
No	breastMilk	39.7142857	No	LessThan20	VaginalBirths	0
GestationalD	nonBreastMi	39	No	LessThan35	VaginalBirths	1
No	nonBreastMi	37	Yes	LessThan35	CSection	3
No	nonBreastMi	37	Yes	LessThan35	CSection	3
No	breastMilk	40.1428571	No	LessThan35	VaginalBirths	0
No	nonBreastMi	41	No	LessThan35	VaginalBirths	0
No	breastMilk	39.2857143	No	LessThan35	VaginalBirths	0
No	breastMilk	41	No	LessThan35	VaginalBirths	1
GestationalD	breastMilk	38.4285714	No	LessThan35	VaginalBirths	0
No	nonBreastMi	38.8571429	No	LessThan35	VaginalBirths	0
No	breastMilk	40.2857143	No	LessThan20	VaginalBirths	1

No	breastMilk	40.7142857	No	LessThan35	VaginalBirths	1
No	breastMilk	38.8571429	No	LessThan35	VaginalBirths	0
No	breastMilk	41.2857143	No	LessThan35	VaginalBirths	2
No	breastMilk	38.8571429	No	LessThan35	CSection	0
No	breastMilk	39.2857143	No	LessThan35	CSection	1
No	breastMilk	39.4285714	No	LessThan35	CSection	2
No	breastMilk	40.2857143	No	LessThan35	CSection	0
No	breastMilk	39.7142857	No	MoreThan35	CSection	1
No	breastMilk	37.4285714	No	LessThan35	CSection	2
No	breastMilk	41.4285714	No	LessThan35	CSection	2
No	breastMilk	41.2857143	No	LessThan35	CSection	0
No	breastMilk	39	No	LessThan35	VaginalBirths	0
No	breastMilk	40.7142857	No	MoreThan35	VaginalBirths	3
No	nonBreastMi	40	No	LessThan35	VaginalBirths	0
No	breastMilk	38.7142857	No	MoreThan35	VaginalBirths	1
No	breastMilk	40.5714286	No	LessThan35	VaginalBirths	2
No	nonBreastMi	39.8571429	No	LessThan35	VaginalBirths	1
No	breastMilk	41	No	LessThan35	VaginalBirths	1
No	breastMilk	39.7142857	No	LessThan35	VaginalBirths	MoreThan3
No	breastMilk	38.1428571	No	LessThan35	VaginalBirths	0
No	nonBreastMi	40.2857143	No	LessThan35	VaginalBirths	0
No	breastMilk	41.1428571	No	LessThan35	VaginalBirths	0
No	breastMilk	39.7142857	Yes	MoreThan35	VaginalBirths	MoreThan3
No	nonBreastMi	41.1428571	No	LessThan35	VaginalBirths	1
No	nonBreastMi	39.7142857	No	LessThan20	VaginalBirths	0
No	breastMilk	39.2857143	No	LessThan35	CSection	2
No	nonBreastMi	38.7142857	No	LessThan35	VaginalBirths	1
No	breastMilk	39.5714286	No	LessThan35	VaginalBirths	0
No	breastMilk	39.5714286	No	LessThan35	CSection	2
No	nonBreastMi	33.1428571	No	LessThan35	CSection	0
No	breastMilk	40.1428571	No	LessThan35	VaginalBirths	1
No	breastMilk	41.2857143	No	LessThan35	CSection	0
No	breastMilk	37.8571429	No	LessThan35	CSection	0
No	nonBreastMi	38.2857143	No	MoreThan35	VaginalBirths	2
No	breastMilk	41.1428571	No	LessThan35	CSection	0
No	breastMilk	41.4285714	No	LessThan35	VaginalBirths	1
No	nonBreastMi	37.5714286	No	MoreThan35	CSection	1
No	breastMilk	37.8571429	No	LessThan35	VaginalBirths	2
No	breastMilk	41.2857143	No	LessThan35	VaginalBirths	0
No	nonBreastMi	32	No	LessThan35	CSection	0
No	breastMilk	39.5714286	No	LessThan35	VaginalBirths	1
No	nonBreastMi	41.2857143	No	LessThan35	VaginalBirths	2
No	breastMilk	40.5714286	No	MoreThan35	VaginalBirths	0
No	breastMilk	39.4285714	No	MoreThan35	CSection	3
No	breastMilk	39.1428571	No	LessThan35	VaginalBirths	2
No	breastMilk	39.2857143	No	LessThan35	CSection	0
No	breastMilk	39.8571429	No	LessThan35	VaginalBirths	0
No	breastMilk	40.2857143	No	LessThan35	VaginalBirths	3

No	breastMilk	39.1428571	No	LessThan35	VaginalBirths	0
No	nonBreastMi	39.2857143	No	LessThan35	CSection	0
No	breastMilk	40.8571429	No	LessThan35	VaginalBirths	0
No	nonBreastMi	39.7142857	No	LessThan35	VaginalBirths	0
No	breastMilk	40.5714286	No	LessThan35	VaginalBirths	0
No	breastMilk	40	No	LessThan35	VaginalBirths	0
No	breastMilk	40.8571429	No	LessThan35	CSection	0
GestationalD	nonBreastMi	38.7142857	No	MoreThan35	CSection	0
No	breastMilk	39.4285714	No	LessThan35	CSection	2
No	breastMilk	39.4285714	No	MoreThan35	CSection	1
No	breastMilk	35	No	LessThan35	VaginalBirths	1
No	breastMilk	38.7142857	No	LessThan35	VaginalBirths	0
No	breastMilk	39.7142857	No	LessThan20	VaginalBirths	0
PreExisting	breastMilk	38	No	LessThan35	VaginalBirths	MoreThan3
No	breastMilk	39.7142857	No	LessThan35	CSection	0
No	breastMilk	38.8571429	No	LessThan35	VaginalBirths	1
No	breastMilk	39.4285714	No	LessThan35	VaginalBirths	1
No	breastMilk	39.7142857	No	LessThan35	VaginalBirths	1
No	breastMilk	40	No	LessThan35	VaginalBirths	0
No	breastMilk	40.7142857	No	LessThan35	CSection	1
No	breastMilk	39.2857143	No	LessThan35	VaginalBirths	0
No	breastMilk	41.2857143	No	LessThan35	CSection	1
PreExisting	nonBreastMi	38	No	LessThan35	VaginalBirths	0
GestationalD	breastMilk	38.2857143	No	LessThan35	CSection	0
No	nonBreastMi	41.1428571	No	LessThan35	CSection	0
No	breastMilk	39.8571429	No	LessThan35	VaginalBirths	1
No	breastMilk	40	No	LessThan35	CSection	0
GestationalD	breastMilk	39.5714286	No	MoreThan35	VaginalBirths	1
No	breastMilk	39.5714286	No	LessThan35	VaginalBirths	0
No	breastMilk	41.4285714	No	LessThan35	VaginalBirths	1
No	breastMilk	39.1428571	No	LessThan35	CSection	0
GestationalD	nonBreastMi	39	No	LessThan35	CSection	1
No	breastMilk	39.8571429	No	LessThan35	VaginalBirths	2
No	breastMilk	38	No	LessThan35	VaginalBirths	2
No	breastMilk	38.1428571	No	LessThan35	VaginalBirths	1
No	breastMilk	41.1428571	No	LessThan35	CSection	0
No	breastMilk	39.8571429	No	LessThan35	VaginalBirths	3
No	breastMilk	38.5714286	No	LessThan35	VaginalBirths	0
GestationalD	nonBreastMi	37	No	LessThan35	VaginalBirths	3
No	breastMilk	36.5714286	No	LessThan35	CSection	1
No	nonBreastMi	40.5714286	No	LessThan20	VaginalBirths	0
No	breastMilk	41	No	LessThan35	CSection	0
No	breastMilk	40.2857143	No	LessThan35	VaginalBirths	0
No	breastMilk	40.7142857	No	LessThan35	CSection	1
No	nonBreastMi	40	No	LessThan35	CSection	3
No	breastMilk	38	No	LessThan35	VaginalBirths	1
No	breastMilk	40.7142857	No	LessThan35	VaginalBirths	2
No	breastMilk	38.7142857	No	LessThan35	VaginalBirths	1

No	breastMilk	41.1428571	No	LessThan35	VaginalBirths	1
No	breastMilk	40.2857143	No	LessThan35	VaginalBirths	0
No	breastMilk	40	No	LessThan35	VaginalBirths	0
No	nonBreastMi	40.5714286	No	LessThan35	VaginalBirths	2
No	nonBreastMi	39.8571429	No	LessThan35	VaginalBirths	1
No	breastMilk	36.4285714	No	LessThan35	VaginalBirths	0
GestationalD	breastMilk	35	No	LessThan35	CSection	1
No	breastMilk	37.8571429	No	MoreThan35	VaginalBirths	2
No	breastMilk	38.8571429	No	LessThan35	VaginalBirths	0
No	breastMilk	39.7142857	No	LessThan35	CSection	0
No	breastMilk	37.1428571	No	LessThan35	VaginalBirths	1
No	breastMilk	37.8571429	No	LessThan35	CSection	2
No	breastMilk	41.2857143	No	LessThan35	VaginalBirths	0
No	nonBreastMi	37.5714286	No	LessThan35	CSection	0
No	breastMilk	39.4285714	No	LessThan35	CSection	0
No	breastMilk	41.4285714	No	LessThan35	CSection	0
No	breastMilk	39.8571429	No	LessThan35	VaginalBirths	2
No	breastMilk	34.7142857	No	MoreThan35	VaginalBirths	1
No	breastMilk	39.1428571	No	LessThan35	CSection	1
GestationalD	breastMilk	38.1428571	No	LessThan35	VaginalBirths	0
No	breastMilk	39.4285714	No	MoreThan35	VaginalBirths	0
No	breastMilk	40.7142857	No	LessThan35	VaginalBirths	0
No	breastMilk	39.7142857	No	LessThan35	VaginalBirths	1
No	breastMilk	38.8571429	No	LessThan35	CSection	1
No	breastMilk	38.7142857	No	MoreThan35	CSection	2
No	breastMilk	41.4285714	No	LessThan35	VaginalBirths	2
No	nonBreastMi	39	No	LessThan35	CSection	0
No	breastMilk	40.8571429	No	MoreThan35	VaginalBirths	0
No	breastMilk	40.8571429	No	LessThan35	VaginalBirths	2
No	breastMilk	41.4285714	No	LessThan35	VaginalBirths	1
No	breastMilk	41	No	LessThan35	VaginalBirths	0
GestationalD	breastMilk	40.5714286	No	LessThan35	CSection	0
GestationalD	breastMilk	37.5714286	No	LessThan35	VaginalBirths	1
No	nonBreastMi	40.7142857	No	LessThan20	VaginalBirths	0
No	nonBreastMi	40.7142857	No	LessThan35	CSection	0
No	breastMilk	35.1428571	No	MoreThan35	VaginalBirths	1
No	nonBreastMi	38.5714286	No	LessThan20	VaginalBirths	0
No	nonBreastMi	40.1428571	No	LessThan35	VaginalBirths	1
No	breastMilk	40.1428571	No	LessThan20	VaginalBirths	0
No	breastMilk	38.5714286	No	LessThan35	VaginalBirths	1
No	breastMilk	38.7142857	No	MoreThan35	CSection	2
GestationalD	breastMilk	39.2857143	No	LessThan35	CSection	0
No	breastMilk	39.2857143	No	LessThan35	VaginalBirths	1
No	breastMilk	41.2857143	No	LessThan35	VaginalBirths	3
No	breastMilk	40	No	LessThan35	CSection	0
No	breastMilk	34	No	LessThan35	CSection	1
No	breastMilk	40.1428571	No	LessThan35	VaginalBirths	0
No	breastMilk	38.5714286	No	LessThan35	VaginalBirths	0

No	breastMilk	39	No		MoreThan35	VaginalBirths	0
No	breastMilk	37.1428571	No		LessThan35	CSection	0
GestationalD	breastMilk	38.4285714	No		LessThan35	CSection	1
No	breastMilk	41.1428571	No		LessThan35	CSection	2
No	breastMilk	40	No		LessThan35	VaginalBirths	0
No	nonBreastMi	41.5714286	No		LessThan35	CSection	0
No	breastMilk	39.5714286	No		LessThan35	VaginalBirths	0
No	breastMilk	33	No		LessThan35	CSection	0
No	breastMilk	39.8571429	No		LessThan35	CSection	1
No	breastMilk	39.1428571	No		LessThan35	CSection	0
No	breastMilk	40.1428571	No		MoreThan35	VaginalBirths	0
No	breastMilk	41.7142857	No		LessThan35	VaginalBirths	0
No	breastMilk	39.5714286	No		LessThan35	VaginalBirths	0
No	breastMilk	39	No		LessThan35	CSection	1
GestationalD	breastMilk	39.1428571	No		LessThan35	VaginalBirths	2
No	breastMilk	38.4285714	No		MoreThan35	VaginalBirths	2
No	breastMilk	39.7142857	No		LessThan35	VaginalBirths	0
No	nonBreastMi	39.7142857	No		LessThan35	CSection	1
No	breastMilk	39.7142857	No		MoreThan35	VaginalBirths	2
No	breastMilk	38.7142857	No		LessThan35	CSection	1
GestationalD	breastMilk	39.8571429	No		LessThan35	CSection	0
No	breastMilk	38.7142857	No		LessThan35	VaginalBirths	1
No	breastMilk	34.5714286	No		MoreThan35	CSection	0
No	nonBreastMi	39.1428571	No		LessThan35	VaginalBirths	0
No	breastMilk	41.4285714	No		LessThan35	VaginalBirths	0
GestationalD	breastMilk	40	No		LessThan35	CSection	0
No	nonBreastMi	37.5714286	Yes		LessThan35	VaginalBirths	1
No	nonBreastMi	38.7142857	No		LessThan35	CSection	0
No	breastMilk	38	No		LessThan35	VaginalBirths	1
GestationalD	nonBreastMi	38.8571429	No		LessThan35	VaginalBirths	0
No	breastMilk	39.1428571	No		LessThan35	VaginalBirths	0
No	nonBreastMi	40.1428571	No		LessThan35	VaginalBirths	1
No	nonBreastMi	39.2857143	No		LessThan35	VaginalBirths	1
No	breastMilk	39.2857143	No		LessThan35	VaginalBirths	2
No	breastMilk	39.8571429	No		LessThan35	CSection	1
No	breastMilk	41.1428571	No		LessThan35	VaginalBirths	0
No	breastMilk	40.7142857	No		LessThan35	CSection	0
No	nonBreastMi	41.4285714	No		LessThan35	VaginalBirths	1
No	breastMilk	41.2857143	No		LessThan35	VaginalBirths	1
No	breastMilk	39.8571429	No		LessThan35	CSection	0
No	breastMilk	40	No		LessThan35	VaginalBirths	1
No	breastMilk	39.5714286	No		LessThan35	CSection	1
No	breastMilk	40.7142857	No		LessThan35	VaginalBirths	1
No	breastMilk	39.5714286	No		LessThan35	VaginalBirths	0
No	breastMilk	39.4285714	No		MoreThan35	CSection	0
No	breastMilk	38.2857143	No		LessThan35	VaginalBirths	MoreThan3
No	breastMilk	40.2857143	No		LessThan35	VaginalBirths	0
No	nonBreastMi	39	No		LessThan35	CSection	0

No	nonBreastMi	41.2857143	No	LessThan35	VaginalBirths	0
PreExisting	breastMilk	37.7142857	No	LessThan35	CSection	2
GestationalD	nonBreastMi	39.2857143	No	MoreThan35	VaginalBirths	2
No	breastMilk	39.5714286	No	LessThan35	VaginalBirths	2
No	breastMilk	41.4285714	No	LessThan35	CSection	0
GestationalD	nonBreastMi	38.5714286	No	LessThan35	CSection	1
No	breastMilk	40.7142857	No	LessThan35	VaginalBirths	2
No	breastMilk	39	No	LessThan35	VaginalBirths	1
No	breastMilk	40.1428571	No	MoreThan35	VaginalBirths	2
No	breastMilk	37.4285714	No	LessThan35	VaginalBirths	0
No	breastMilk	41.2857143	No	LessThan35	VaginalBirths	1
No	nonBreastMi	35.4285714	No	LessThan35	VaginalBirths	3
No	breastMilk	40.8571429	No	LessThan35	VaginalBirths	3
No	breastMilk	39.4285714	No	LessThan35	CSection	1
No	nonBreastMi	34.8571429	No	MoreThan35	CSection	1
No	breastMilk	39.4285714	No	LessThan35	VaginalBirths	2
No	nonBreastMi	39.1428571	No	LessThan35	VaginalBirths	0
No	nonBreastMi	38.8571429	No	LessThan35	CSection	2
No	breastMilk	36.8571429	No	LessThan35	VaginalBirths	1
GestationalD	breastMilk	39.8571429	No	MoreThan35	CSection	0
No	breastMilk	40.8571429	No	LessThan35	VaginalBirths	0
No	breastMilk	41.5714286	No	LessThan35	VaginalBirths	1
No	nonBreastMi	40	No	LessThan20	CSection	0
No	breastMilk	32.7142857	No	LessThan35	CSection	2
No	breastMilk	39.1428571	No	LessThan35	CSection	0
No	breastMilk	41	No	LessThan35	VaginalBirths	1
GestationalD	breastMilk	41.8571429	No	LessThan35	CSection	0
No	nonBreastMi	37.7142857	No	LessThan35	CSection	0
No	breastMilk	36.1428571	No	MoreThan35	VaginalBirths	2
No	breastMilk	39.7142857	No	LessThan35	VaginalBirths	3
No	breastMilk	39	No	LessThan35	CSection	1
No	nonBreastMi	32.7142857	No	MoreThan35	VaginalBirths	MoreThan3
No	breastMilk	37.5714286	No	LessThan35	CSection	3
No	breastMilk	37.5714286	No	LessThan35	CSection	1
No	breastMilk	39.7142857	No	LessThan35	VaginalBirths	0
No	breastMilk	38.8571429	No	LessThan35	CSection	1
GestationalD	breastMilk	40	No	LessThan35	VaginalBirths	0
No	breastMilk	36.4285714	No	LessThan35	CSection	1
No	breastMilk	40	No	LessThan35	VaginalBirths	1
GestationalD	breastMilk	36.7142857	No	MoreThan35	VaginalBirths	MoreThan3
No	breastMilk	39.5714286	No	LessThan35	VaginalBirths	1
No	breastMilk	40.4285714	No	LessThan35	VaginalBirths	0
No	breastMilk	41.1428571	No	LessThan35	VaginalBirths	0
No	nonBreastMi	39	No	LessThan35	VaginalBirths	2
No	nonBreastMi	38.1428571	No	LessThan35	VaginalBirths	1
GestationalD	nonBreastMi	38.5714286	No	MoreThan35	CSection	1
No	breastMilk	40.5714286	No	MoreThan35	CSection	2
No	breastMilk	39.1428571	No	LessThan35	VaginalBirths	3

No	breastMilk	39.5714286	No	MoreThan35	VaginalBirths	2
No	breastMilk	41.1428571	No	MoreThan35	VaginalBirths	0
No	breastMilk	38.5714286	No	MoreThan35	CSection	1
No	nonBreastMi	32.1428571	No	LessThan35	CSection	2
No	breastMilk	39.1428571	No	LessThan35	CSection	2
No	breastMilk	41.1428571	No	LessThan35	VaginalBirths	0
No	breastMilk	39	No	LessThan20	CSection	0
No	breastMilk	34.5714286	No	LessThan35	VaginalBirths	0
No	nonBreastMi	39.5714286	No	LessThan20	VaginalBirths	0
No	nonBreastMi	41	No	LessThan35	VaginalBirths	2
No	breastMilk	40.1428571	No	LessThan35	CSection	0
No	breastMilk	37.7142857	No	MoreThan35	CSection	2
No	breastMilk	38.4285714	No	LessThan35	CSection	0
GestationalD	breastMilk	39.1428571	No	MoreThan35	CSection	1
No	breastMilk	37	No	LessThan35	VaginalBirths	2
No	breastMilk	41.2857143	No	LessThan35	VaginalBirths	0
No	nonBreastMi	41	No	LessThan35	VaginalBirths	0
No	breastMilk	35.5714286	No	MoreThan35	VaginalBirths	1
No	breastMilk	39	No	MoreThan35	CSection	MoreThan3
No	breastMilk	39.8571429	No	LessThan35	VaginalBirths	1
No	breastMilk	41	No	LessThan35	CSection	0
No	breastMilk	41.1428571	No	MoreThan35	VaginalBirths	0
No	breastMilk	40	No	LessThan35	VaginalBirths	1
No	breastMilk	37.7142857	No	LessThan35	VaginalBirths	0
No	breastMilk	40.2857143	No	LessThan35	VaginalBirths	0
No	breastMilk	38.7142857	No	MoreThan35	VaginalBirths	1
No	breastMilk	39.5714286	No	LessThan35	VaginalBirths	0
No	breastMilk	41.1428571	No	MoreThan35	CSection	1
PreExisting	breastMilk	38.2857143	No	LessThan35	VaginalBirths	1
No	nonBreastMi	36.1428571	No	LessThan35	VaginalBirths	2
No	breastMilk	38.7142857	No	LessThan35	CSection	2
No	breastMilk	39	No	LessThan35	VaginalBirths	0
No	breastMilk	41	No	MoreThan35	CSection	1
No	breastMilk	40.4285714	No	LessThan35	VaginalBirths	2
GestationalD	breastMilk	36.7142857	No	LessThan35	CSection	1
No	breastMilk	38	No	LessThan35	CSection	1
No	breastMilk	39.2857143	No	LessThan35	VaginalBirths	MoreThan3
No	breastMilk	41	No	LessThan20	VaginalBirths	0
No	breastMilk	38.1428571	No	LessThan35	VaginalBirths	1
GestationalD	breastMilk	39.8571429	No	LessThan35	VaginalBirths	0
No	nonBreastMi	39.5714286	No	LessThan35	CSection	1
No	breastMilk	39.8571429	No	LessThan35	CSection	1
No	breastMilk	40.5714286	No	LessThan35	VaginalBirths	2
No	breastMilk	40.4285714	No	LessThan35	VaginalBirths	1
No	breastMilk	37.7142857	No	LessThan35	VaginalBirths	0
No	breastMilk	38.8571429	No	LessThan35	VaginalBirths	0
No	breastMilk	41.2857143	No	LessThan35	VaginalBirths	1
No	nonBreastMi	38.8571429	No	LessThan35	VaginalBirths	1

No	nonBreastMi	39.1428571	No	LessThan35	VaginalBirths	MoreThan3
No	breastMilk	37	No	MoreThan35	VaginalBirths	2
No	breastMilk	40.1428571	No	LessThan35	VaginalBirths	MoreThan3
No	nonBreastMi	39.8571429	No	LessThan20	VaginalBirths	0
No	breastMilk	41.4285714	No	MoreThan35	VaginalBirths	3
No	breastMilk	38	No	LessThan35	CSection	2
GestationalD	nonBreastMi	39	No	LessThan35	CSection	1
No	breastMilk	37.4285714	No	LessThan35	VaginalBirths	MoreThan3
No	nonBreastMi	37.7142857	No	LessThan35	VaginalBirths	1
No	breastMilk	39.7142857	No	MoreThan35	VaginalBirths	3
No	nonBreastMi	36.2857143	No	LessThan35	CSection	1
No	nonBreastMi	37.2857143	No	LessThan35	VaginalBirths	1
No	breastMilk	39	No	LessThan35	VaginalBirths	0
No	breastMilk	38.8571429	No	LessThan35	VaginalBirths	1
No	breastMilk	40	No	LessThan35	CSection	0
No	breastMilk	38.1428571	No	LessThan35	CSection	0
No	breastMilk	38.8571429	No	LessThan35	VaginalBirths	0
No	breastMilk	38.4285714	No	MoreThan35	VaginalBirths	MoreThan3
GestationalD	breastMilk	40	No	LessThan35	VaginalBirths	0
No	nonBreastMi	36.5714286	Yes	LessThan20	VaginalBirths	0
No	breastMilk	39.4285714	No	LessThan35	VaginalBirths	2
No	breastMilk	40.2857143	No	LessThan35	VaginalBirths	1
No	breastMilk	40.8571429	No	MoreThan35	CSection	0
No	breastMilk	37.4285714	No	LessThan35	VaginalBirths	0
No	breastMilk	41.5714286	No	MoreThan35	CSection	2
No	nonBreastMi	38.2857143	No	LessThan35	VaginalBirths	2
No	breastMilk	39.1428571	No	LessThan35	CSection	0
No	nonBreastMi	40.5714286	No	LessThan20	VaginalBirths	0
No	breastMilk	38	No	LessThan35	CSection	0
No	breastMilk	39	No	LessThan35	CSection	1
No	breastMilk	41	No	LessThan35	CSection	0
No	breastMilk	37.4285714	No	LessThan35	CSection	2
No	breastMilk	39.8571429	No	MoreThan35	CSection	1
No	breastMilk	41.2857143	No	LessThan35	VaginalBirths	0
No	breastMilk	40	No	LessThan35	VaginalBirths	0
No	nonBreastMi	40.2857143	No	LessThan35	VaginalBirths	0
No	breastMilk	40.4285714	No	LessThan35	VaginalBirths	0
No	breastMilk	39.7142857	No	LessThan35	VaginalBirths	1
No	breastMilk	39	No	LessThan35	CSection	0
No	breastMilk	38.5714286	No	LessThan35	VaginalBirths	2
No	nonBreastMi	39.1428571	No	LessThan35	CSection	1
No	breastMilk	40.4285714	No	LessThan35	VaginalBirths	0
No	breastMilk	38.8571429	No	LessThan35	CSection	2
GestationalD	nonBreastMi	39.7142857	No	LessThan35	CSection	0
GestationalD	nonBreastMi	39	No	LessThan35	VaginalBirths	2
No	breastMilk	38	No	MoreThan35	CSection	2
No	breastMilk	38.4285714	No	LessThan35	VaginalBirths	0
No	breastMilk	41	No	LessThan35	VaginalBirths	0

No	breastMilk	40.7142857	No	LessThan35	VaginalBirths	2
No	breastMilk	37.7142857	No	LessThan35	CSection	3
No	breastMilk	38.4285714	No	LessThan35	CSection	0
No	breastMilk	41.4285714	No	LessThan35	CSection	0
No	breastMilk	41.5714286	No	LessThan35	CSection	0
No	breastMilk	40.1428571	No	MoreThan35	CSection	MoreThan3
No	breastMilk	40.5714286	No	LessThan20	VaginalBirths	0
No	breastMilk	39.2857143	No	LessThan35	VaginalBirths	0
No	breastMilk	38.2857143	No	LessThan35	CSection	3
No	breastMilk	39.4285714	No	LessThan35	CSection	0
No	nonBreastMi	39.5714286	No	LessThan20	VaginalBirths	1
No	breastMilk	39.8571429	No	LessThan35	CSection	0
No	breastMilk	40.5714286	No	LessThan35	VaginalBirths	0
No	breastMilk	39	No	MoreThan35	VaginalBirths	MoreThan3
No	breastMilk	41.5714286	No	LessThan35	CSection	0
No	nonBreastMi	37.4285714	No	LessThan35	CSection	1
No	breastMilk	39.8571429	No	LessThan35	CSection	0
No	breastMilk	39.2857143	No	LessThan35	VaginalBirths	1
GestationalD	breastMilk	39.1428571	No	LessThan35	CSection	0
No	breastMilk	38.1428571	No	LessThan35	VaginalBirths	0
No	breastMilk	40.1428571	No	LessThan35	VaginalBirths	0
No	breastMilk	38.8571429	No	LessThan35	VaginalBirths	1
No	nonBreastMi	35.8571429	No	LessThan35	VaginalBirths	0
No	breastMilk	41.4285714	No	LessThan35	VaginalBirths	0
No	breastMilk	39.2857143	No	LessThan35	VaginalBirths	0
No	breastMilk	38.5714286	No	LessThan35	VaginalBirths	0
No	breastMilk	40.5714286	No	MoreThan35	CSection	2
No	nonBreastMi	38.1428571	No	LessThan35	CSection	0
No	nonBreastMi	36.7142857	No	LessThan35	VaginalBirths	2
No	nonBreastMi	39.8571429	No	LessThan35	VaginalBirths	0
No	breastMilk	40.1428571	No	LessThan35	CSection	0
No	breastMilk	40.8571429	No	LessThan35	VaginalBirths	1
No	breastMilk	41.4285714	No	MoreThan35	VaginalBirths	1
No	breastMilk	40.5714286	No	MoreThan35	CSection	0
No	breastMilk	38.7142857	No	MoreThan35	VaginalBirths	1
No	breastMilk	40.7142857	No	LessThan35	VaginalBirths	0
No	breastMilk	41.5714286	No	LessThan35	VaginalBirths	1
No	breastMilk	38.8571429	No	MoreThan35	VaginalBirths	2
No	breastMilk	34.4285714	No	LessThan35	VaginalBirths	1
No	nonBreastMi	38.2857143	No	MoreThan35	CSection	1
No	breastMilk	41.1428571	No	LessThan20	VaginalBirths	0
No	breastMilk	39.8571429	No	LessThan35	VaginalBirths	0
No	breastMilk	36.1428571	No	LessThan35	VaginalBirths	3
No	nonBreastMi	40.5714286	No	LessThan35	CSection	0
No	breastMilk	39	No	MoreThan35	VaginalBirths	2
No	breastMilk	41.2857143	Yes	LessThan35	VaginalBirths	MoreThan3
No	breastMilk	37.1428571	No	LessThan35	VaginalBirths	2
No	nonBreastMi	40.1428571	No	LessThan35	CSection	0

No	breastMilk	41	No	LessThan35	VaginalBirths		1
No	breastMilk	39.1428571	No	LessThan35	VaginalBirths		1
No	nonBreastMi	40.4285714	No	LessThan35	CSection		1
No	breastMilk	39.1428571	No	LessThan20	VaginalBirths		0
No	breastMilk	41.4285714	No	LessThan35	CSection		0
No	nonBreastMi	39.2857143	No	LessThan20	VaginalBirths		0
No	breastMilk	39.1428571	No	LessThan35	VaginalBirths		0
No	breastMilk	39.7142857	No	LessThan35	VaginalBirths		2
No	breastMilk	39.8571429	No	LessThan35	VaginalBirths		0
GestationalD	breastMilk	40.2857143	No	LessThan35	VaginalBirths		2
No	breastMilk	41.4285714	No	LessThan35	VaginalBirths		0
No	nonBreastMi	39.2857143	No	LessThan35	VaginalBirths		1
No	breastMilk	38	No	MoreThan35	VaginalBirths	MoreThan3	
No	breastMilk	38.4285714	No	LessThan35	VaginalBirths		0
No	breastMilk	39	No	LessThan35	CSection		0
No	breastMilk	39	No	MoreThan35	VaginalBirths		1
PreExisting	breastMilk	39.1428571	No	LessThan35	VaginalBirths		0
No	breastMilk	40.1428571	No	LessThan35	VaginalBirths		0
No	nonBreastMi	40.2857143	No	LessThan35	VaginalBirths		0
No	breastMilk	41	No	LessThan35	VaginalBirths		1
GestationalD	nonBreastMi	39.5714286	No	LessThan35	CSection		1
No	breastMilk	39.2857143	No	LessThan35	VaginalBirths		0
No	breastMilk	37.8571429	No	LessThan35	VaginalBirths		1
No	breastMilk	39.7142857	No	LessThan35	VaginalBirths		1
No	nonBreastMi	39.5714286	No	LessThan35	VaginalBirths		0
No	breastMilk	38.4285714	No	LessThan35	CSection		0
No	nonBreastMi	41.4285714	No	LessThan35	VaginalBirths		1
No	breastMilk	36.2857143	No	LessThan20	CSection		0
PreExisting	breastMilk	36.7142857	No	LessThan35	CSection		0
No	breastMilk	40	No	LessThan35	CSection		0
No	nonBreastMi	40.5714286	No	LessThan20	CSection		0
No	breastMilk	38.1428571	No	LessThan35	CSection		0
No	nonBreastMi	38.7142857	No	LessThan35	VaginalBirths		0
No	breastMilk	40.1428571	No	LessThan35	VaginalBirths		0
No	breastMilk	40.7142857	No	LessThan35	CSection		0
No	breastMilk	38.5714286	No	LessThan35	CSection		0
No	breastMilk	40	No	LessThan35	VaginalBirths		0
PreExisting	breastMilk	39	No	LessThan35	CSection		1
GestationalD	breastMilk	39.4285714	No	LessThan35	VaginalBirths		0
No	breastMilk	39.8571429	No	LessThan35	CSection		0
No	breastMilk	38	No	LessThan35	VaginalBirths		0
No	nonBreastMi	38	No	LessThan35	VaginalBirths		0
No	breastMilk	40.5714286	No	LessThan35	VaginalBirths		1
No	breastMilk	38.5714286	No	MoreThan35	CSection		1
No	breastMilk	41.1428571	No	MoreThan35	CSection		0
No	breastMilk	40.2857143	No	LessThan35	VaginalBirths		0
No	breastMilk	39.2857143	No	LessThan35	VaginalBirths		0
No	breastMilk	39.8571429	No	LessThan35	VaginalBirths		3

No	breastMilk	40.2857143	No		LessThan35	CSection	0
No	nonBreastMi	40.2857143	No		LessThan35	CSection	2
No	breastMilk	39.8571429	No		MoreThan35	VaginalBirths	0
No	nonBreastMi	39.7142857	No		LessThan35	VaginalBirths	1
No	breastMilk	40.4285714	No		LessThan35	VaginalBirths	3
No	nonBreastMi	39.1428571	No		LessThan35	CSection	0
GestationalD	breastMilk	39.5714286	No		LessThan35	VaginalBirths	0
No	breastMilk	40	No		LessThan35	CSection	1
No	breastMilk	40	No		LessThan35	VaginalBirths	0
No	breastMilk	41	No		LessThan35	VaginalBirths	1
No	nonBreastMi	40.7142857	No		LessThan35	CSection	0
No	nonBreastMi	37.7142857	No		MoreThan35	CSection	3
No	breastMilk	41.5714286	No		LessThan35	VaginalBirths	0
No	nonBreastMi	37	No		LessThan35	VaginalBirths	1
No	breastMilk	41.4285714	No		LessThan35	VaginalBirths	1
No	breastMilk	34.8571429	No		LessThan35	VaginalBirths	2
No	breastMilk	41.5714286	No		LessThan35	VaginalBirths	0
No	nonBreastMi	38.1428571	No		LessThan35	VaginalBirths	0
No	breastMilk	40	No		LessThan35	VaginalBirths	0
No	breastMilk	37.4285714	No		LessThan35	VaginalBirths	2
No	nonBreastMi	39.5714286	No		LessThan35	VaginalBirths	1
No	breastMilk	40	No		LessThan35	VaginalBirths	0
No	breastMilk	40.7142857	No		LessThan35	VaginalBirths	0
No	breastMilk	38.5714286	No		MoreThan35	CSection	0
No	nonBreastMi	38.7142857	No		LessThan35	CSection	2
No	nonBreastMi	39	No		LessThan35	CSection	3
No	breastMilk	38.1428571	No		LessThan35	CSection	2
No	breastMilk	38.1428571	No		LessThan35	VaginalBirths	1
No	breastMilk	37.7142857	No		LessThan35	VaginalBirths	2
No	breastMilk	41	No		LessThan35	VaginalBirths	1
No	breastMilk	40.5714286	No		LessThan35	VaginalBirths	0
No	breastMilk	39.7142857	No		LessThan35	CSection	0
No	breastMilk	41	No		LessThan35	VaginalBirths	3
No	nonBreastMi	39.8571429	No		LessThan35	CSection	1
No	nonBreastMi	40.8571429	No		LessThan35	CSection	0
No	breastMilk	39	No		LessThan35	VaginalBirths	3
No	nonBreastMi	39.7142857	No		LessThan35	VaginalBirths	2
No	breastMilk	39.7142857	No		MoreThan35	CSection	1
GestationalD	breastMilk	35.2857143	No		LessThan35	CSection	2
No	breastMilk	40	No		LessThan20	VaginalBirths	0
No	breastMilk	38.4285714	No		LessThan35	VaginalBirths	1
No	breastMilk	41.4285714	No		LessThan35	VaginalBirths	1
No	breastMilk	40.7142857	No		LessThan35	VaginalBirths	1
No	breastMilk	40.5714286	No		MoreThan35	CSection	1
No	breastMilk	40	No		LessThan35	VaginalBirths	0
GestationalD	breastMilk	38.5714286	No		MoreThan35	CSection	0
No	breastMilk	37.1428571	No		LessThan35	VaginalBirths	0
No	breastMilk	40.1428571	No		LessThan35	CSection	0

No	nonBreastMi	41.4285714	No	LessThan35	CSection		0
No	nonBreastMi	39.8571429	No	LessThan35	CSection		1
No	breastMilk	40.5714286	No	LessThan20	VaginalBirths		0
No	nonBreastMi	39.8571429	No	LessThan35	VaginalBirths		1
No	breastMilk	35	No	LessThan35	CSection		2
No	breastMilk	40.1428571	No	LessThan35	VaginalBirths		1
No	breastMilk	41.4285714	No	LessThan35	CSection		0
No	breastMilk	39.8571429	No	LessThan20	VaginalBirths		0
No	breastMilk	38.5714286	No	LessThan35	VaginalBirths		0
No	breastMilk	39.1428571	No	LessThan35	VaginalBirths		2
No	breastMilk	39.7142857	No	MoreThan35	VaginalBirths		2
GestationalD	breastMilk	35.7142857	No	LessThan35	VaginalBirths		0
No	breastMilk	39.5714286	No	MoreThan35	VaginalBirths		0
No	breastMilk	40.5714286	No	LessThan20	CSection		0
No	nonBreastMi	41.4285714	No	LessThan35	CSection		0
No	breastMilk	37.4285714	No	LessThan35	VaginalBirths		0
No	breastMilk	40.2857143	No	LessThan35	VaginalBirths		3
No	breastMilk	41	No	LessThan35	VaginalBirths		1
No	breastMilk	39	No	LessThan35	VaginalBirths		1
No	breastMilk	36.4285714	No	LessThan35	CSection		1
No	breastMilk	41.4285714	No	LessThan35	VaginalBirths		1
GestationalD	breastMilk	39	No	LessThan35	CSection		1
No	breastMilk	36	No	LessThan35	VaginalBirths		1
No	breastMilk	41	No	LessThan35	VaginalBirths		2
PreExisting	breastMilk	38.1428571	No	LessThan35	VaginalBirths		3
No	nonBreastMi	39.4285714	No	LessThan35	VaginalBirths		1
No	breastMilk	37.5714286	No	MoreThan35	CSection		2
No	breastMilk	38.4285714	No	LessThan35	VaginalBirths		0
No	breastMilk	40.4285714	No	LessThan35	VaginalBirths		2
No	nonBreastMi	41.1428571	No	LessThan35	VaginalBirths		1
No	breastMilk	40.5714286	No	LessThan35	VaginalBirths		0
No	breastMilk	38.1428571	No	LessThan35	VaginalBirths		2
No	breastMilk	39.1428571	No	LessThan35	CSection		1
No	nonBreastMi	36.5714286	No	MoreThan35	CSection		0
No	breastMilk	39.5714286	No	LessThan35	CSection		1
No	nonBreastMi	39.1428571	No	MoreThan35	VaginalBirths		2
No	nonBreastMi	39.4285714	No	LessThan35	CSection		1
No	breastMilk	35.5714286	No	LessThan35	CSection		3
No	breastMilk	38.4285714	No	MoreThan35	CSection	MoreThan3	
No	breastMilk	40.5714286	No	LessThan35	VaginalBirths		0
No	breastMilk	38	No	MoreThan35	CSection		2
No	breastMilk	40	No	LessThan35	VaginalBirths		0
No	breastMilk	40.1428571	No	LessThan35	VaginalBirths		0
No	nonBreastMi	41.1428571	No	LessThan35	VaginalBirths		1
No	breastMilk	35.7142857	No	LessThan35	CSection		1
GestationalD	breastMilk	40	No	LessThan35	CSection		0
No	breastMilk	37.4285714	No	LessThan35	VaginalBirths		1
No	breastMilk	40.4285714	No	MoreThan35	CSection		0

No	nonBreastMi	40.4285714	No	LessThan20	VaginalBirths	0
No	nonBreastMi	38.1428571	No	MoreThan35	VaginalBirths	1
No	breastMilk	40.2857143	No	LessThan35	VaginalBirths	0
No	breastMilk	39.8571429	No	MoreThan35	VaginalBirths	0
No	breastMilk	41	No	LessThan35	CSection	1
No	breastMilk	40.8571429	No	LessThan35	CSection	0
No	breastMilk	37.5714286	No	LessThan35	CSection	0
No	breastMilk	40.7142857	No	LessThan35	VaginalBirths	0
No	breastMilk	40	No	LessThan35	VaginalBirths	0
No	breastMilk	41.1428571	No	LessThan35	VaginalBirths	2
No	nonBreastMi	39.5714286	No	LessThan35	CSection	1
No	breastMilk	38.4285714	No	LessThan35	VaginalBirths	0
No	nonBreastMi	40.8571429	No	LessThan35	VaginalBirths	1
No	breastMilk	41.5714286	No	MoreThan35	VaginalBirths	0
No	breastMilk	41	No	LessThan35	VaginalBirths	0
GestationalD	nonBreastMi	38.8571429	No	MoreThan35	VaginalBirths	3
No	breastMilk	37.7142857	No	LessThan35	VaginalBirths	0
No	nonBreastMi	38.1428571	No	LessThan35	CSection	MoreThan3
No	breastMilk	39.7142857	No	LessThan35	VaginalBirths	0
No	breastMilk	39.5714286	No	MoreThan35	VaginalBirths	3
No	breastMilk	41.8571429	No	LessThan35	CSection	0
No	breastMilk	40.5714286	No	LessThan35	VaginalBirths	0
No	nonBreastMi	39	No	LessThan35	CSection	1
No	breastMilk	41.4285714	No	LessThan35	CSection	0
No	breastMilk	38.7142857	No	LessThan35	VaginalBirths	0
No	breastMilk	38.8571429	No	LessThan35	CSection	2
No	breastMilk	40.5714286	No	LessThan35	VaginalBirths	2
No	breastMilk	40.4285714	No	LessThan35	CSection	0
No	breastMilk	37	No	LessThan35	VaginalBirths	2
No	breastMilk	40.8571429	No	LessThan35	VaginalBirths	2
No	nonBreastMi	40.8571429	No	MoreThan35	CSection	MoreThan3
No	breastMilk	38.7142857	No	LessThan35	VaginalBirths	0
No	breastMilk	41.4285714	No	MoreThan35	CSection	1
No	breastMilk	40.2857143	No	LessThan35	VaginalBirths	0
No	nonBreastMi	40.4285714	No	MoreThan35	CSection	1
No	breastMilk	38.5714286	No	LessThan35	VaginalBirths	1
No	breastMilk	38.8571429	No	LessThan35	VaginalBirths	1
No	breastMilk	40	No	MoreThan35	VaginalBirths	0
No	breastMilk	40.2857143	No	LessThan35	VaginalBirths	0
No	breastMilk	39	No	LessThan35	VaginalBirths	2
No	breastMilk	40.5714286	No	LessThan35	VaginalBirths	2
No	breastMilk	40.7142857	No	LessThan35	VaginalBirths	1
No	breastMilk	40	No	LessThan35	CSection	2
No	breastMilk	38.8571429	No	LessThan35	CSection	1
No	breastMilk	39.8571429	No	LessThan35	CSection	0
No	breastMilk	39.7142857	No	LessThan35	CSection	2
No	breastMilk	36.5714286	No	LessThan35	VaginalBirths	1
No	nonBreastMi	39.5714286	No	LessThan20	VaginalBirths	0

PreExisting	breastMilk	38.2857143	No	LessThan35	CSection	1
No	nonBreastMi	40.7142857	No	LessThan35	VaginalBirths	0
No	breastMilk	40.7142857	No	LessThan35	VaginalBirths	0
No	breastMilk	33.2857143	No	LessThan35	CSection	1
No	nonBreastMi	38.2857143	No	LessThan35	VaginalBirths	1
No	breastMilk	41.7142857	No	LessThan35	VaginalBirths	0
No	breastMilk	39.4285714	No	LessThan35	VaginalBirths	1
No	breastMilk	40.4285714	No	MoreThan35	VaginalBirths	3
No	breastMilk	38.1428571	No	LessThan35	VaginalBirths	1
No	breastMilk	33.1428571	No	LessThan35	CSection	1
No	breastMilk	40.8571429	No	LessThan35	VaginalBirths	1
No	breastMilk	40	No	MoreThan35	VaginalBirths	MoreThan3
No	nonBreastMi	40	No	LessThan35	VaginalBirths	3
No	breastMilk	41	No	MoreThan35	VaginalBirths	1
No	breastMilk	39	No	LessThan35	CSection	0
No	breastMilk	39	Yes	LessThan35	CSection	0
No	breastMilk	40.5714286	No	LessThan35	VaginalBirths	3
No	breastMilk	39.7142857	No	LessThan20	VaginalBirths	0
No	breastMilk	37	No	LessThan35	CSection	0
GestationalD	nonBreastMi	37.4285714	No	LessThan35	CSection	0
No	breastMilk	41.5714286	No	LessThan35	CSection	0
No	nonBreastMi	40.2857143	No	LessThan35	VaginalBirths	1
No	nonBreastMi	40.7142857	No	LessThan35	CSection	0
No	breastMilk	39.4285714	No	LessThan20	VaginalBirths	0
No	breastMilk	38.1428571	No	LessThan35	CSection	1
No	nonBreastMi	40.7142857	No	MoreThan35	VaginalBirths	2
No	breastMilk	38.5714286	No	MoreThan35	CSection	0
No	nonBreastMi	37	No	LessThan35	CSection	1
No	breastMilk	41.2857143	No	LessThan35	VaginalBirths	0
No	nonBreastMi	41.2857143	No	MoreThan35	VaginalBirths	0
No	breastMilk	37.7142857	No	LessThan35	CSection	0
No	nonBreastMi	36.4285714	No	MoreThan35	CSection	2
No	breastMilk	41.2857143	No	LessThan35	VaginalBirths	0
No	breastMilk	39	No	LessThan35	CSection	0
No	nonBreastMi	41.4285714	No	LessThan35	CSection	0
No	breastMilk	39.7142857	No	LessThan35	CSection	2
No	breastMilk	39.2857143	No	LessThan35	CSection	1
No	nonBreastMi	40	No	LessThan35	VaginalBirths	1
No	breastMilk	38	No	LessThan35	CSection	1
No	nonBreastMi	39.7142857	No	LessThan35	CSection	1
No	breastMilk	40.5714286	No	LessThan35	VaginalBirths	1
No	breastMilk	39.8571429	No	LessThan35	VaginalBirths	MoreThan3
No	nonBreastMi	40.8571429	No	LessThan20	VaginalBirths	0
No	breastMilk	39	No	LessThan35	CSection	1
No	breastMilk	37.7142857	No	MoreThan35	VaginalBirths	1
No	breastMilk	39.5714286	No	LessThan35	CSection	1
No	breastMilk	41.1428571	No	LessThan35	VaginalBirths	0
No	breastMilk	39.4285714	No	MoreThan35	VaginalBirths	0

GestationalD	breastMilk	35	No		MoreThan35	VaginalBirths		2
No	breastMilk	40	No		MoreThan35	VaginalBirths	MoreThan3	
No	nonBreastMi	40	No		LessThan35	VaginalBirths		1
No	breastMilk	36.7142857	No		MoreThan35	VaginalBirths		2
No	breastMilk	41.2857143	No		LessThan35	VaginalBirths		0
No	nonBreastMi	41.4285714	No		MoreThan35	VaginalBirths		2
No	breastMilk	39.4285714	No		LessThan35	VaginalBirths		1
No	breastMilk	35.7142857	No		LessThan35	CSection		0
No	breastMilk	41.1428571	No		LessThan35	CSection		1
No	nonBreastMi	37.8571429	No		LessThan35	CSection		0
No	breastMilk	38.7142857	No		LessThan35	VaginalBirths		1
No	nonBreastMi	39.7142857	No		LessThan35	CSection	MoreThan3	
No	nonBreastMi	40.4285714	No		LessThan35	VaginalBirths		1
GestationalD	breastMilk	39.4285714	No		LessThan35	VaginalBirths		1
No	breastMilk	38.2857143	No		LessThan35	CSection		2
No	nonBreastMi	36.1428571	No		MoreThan35	VaginalBirths		2
No	nonBreastMi	37.8571429	No		LessThan35	VaginalBirths		1
No	nonBreastMi	39.7142857	No		LessThan35	VaginalBirths		1
No	breastMilk	37.2857143	No		MoreThan35	VaginalBirths		3
No	breastMilk	37.7142857	No		LessThan35	CSection		0
No	breastMilk	38	No		LessThan35	VaginalBirths		1
No	breastMilk	38	No		LessThan35	VaginalBirths		1
No	breastMilk	41.2857143	No		LessThan35	VaginalBirths		0
No	breastMilk	40.2857143	No		LessThan35	VaginalBirths		1
No	breastMilk	41.1428571	No		LessThan35	VaginalBirths		0
No	breastMilk	39	No		LessThan20	VaginalBirths		0
No	nonBreastMi	39.1428571	No		LessThan35	VaginalBirths		2
No	breastMilk	39	No		LessThan35	CSection		1
No	nonBreastMi	35.4285714	No		LessThan35	VaginalBirths		0
No	breastMilk	37.5714286	No		LessThan35	VaginalBirths		0
No	breastMilk	41	No		LessThan35	VaginalBirths		0
No	breastMilk	39	No		LessThan35	VaginalBirths		1
No	breastMilk	40.7142857	No		LessThan35	VaginalBirths		2
No	breastMilk	39.1428571	No		LessThan35	VaginalBirths		0
No	breastMilk	39.1428571	No		LessThan35	CSection		1
No	breastMilk	38.8571429	No		LessThan35	CSection		1
No	breastMilk	41.2857143	No		LessThan35	VaginalBirths		0
GestationalD	breastMilk	38.8571429	No		MoreThan35	VaginalBirths		1
No	breastMilk	41.4285714	No		LessThan35	VaginalBirths		0
No	breastMilk	41.4285714	No		LessThan35	CSection		0
No	breastMilk	38.8571429	No		LessThan35	CSection		0
No	breastMilk	40.4285714	No		LessThan35	VaginalBirths		2
No	breastMilk	40.4285714	No		LessThan35	VaginalBirths		1
No	breastMilk	41.5714286	No		MoreThan35	VaginalBirths		2
No	nonBreastMi	38	No		LessThan35	CSection		2
No	breastMilk	40.8571429	No		MoreThan35	VaginalBirths		2
No	breastMilk	40.8571429	No		LessThan35	VaginalBirths		0
GestationalD	breastMilk	39.1428571	No		MoreThan35	CSection		2

No	breastMilk	39.7142857	No	LessThan35	VaginalBirth:	MoreThan3
No	breastMilk	38	No	LessThan35	CSection	1
No	breastMilk	41.1428571	No	MoreThan35	VaginalBirth:	1
No	breastMilk	41.2857143	No	LessThan35	VaginalBirth:	1
No	nonBreastMi	40.4285714	No	LessThan35	CSection	0
No	nonBreastMi	37.2857143	No	MoreThan35	CSection	3
No	nonBreastMi	40.7142857	Yes	LessThan35	VaginalBirth:	3
No	breastMilk	38.1428571	No	LessThan35	CSection	2
No	nonBreastMi	38.7142857	No	LessThan35	VaginalBirth:	1
No	breastMilk	41.2857143	No	LessThan35	VaginalBirth:	3
No	breastMilk	40.1428571	No	LessThan35	VaginalBirth:	1
No	nonBreastMi	39.1428571	No	LessThan35	CSection	1
No	breastMilk	41.1428571	No	LessThan35	VaginalBirth:	0
No	breastMilk	38.7142857	No	LessThan20	VaginalBirth:	0
No	breastMilk	39.4285714	No	LessThan35	VaginalBirth:	0
No	nonBreastMi	40.4285714	No	LessThan35	CSection	0
No	breastMilk	39	No	MoreThan35	CSection	2
No	breastMilk	40	No	LessThan35	VaginalBirth:	3
No	breastMilk	40.1428571	No	LessThan35	VaginalBirth:	3
No	nonBreastMi	38.1428571	No	LessThan35	VaginalBirth:	0
No	breastMilk	40.7142857	No	LessThan35	VaginalBirth:	0
No	breastMilk	34.1428571	No	LessThan35	VaginalBirth:	0
No	breastMilk	40.8571429	No	LessThan35	VaginalBirth:	1
No	breastMilk	39	No	LessThan35	VaginalBirth:	1
No	breastMilk	39.7142857	No	LessThan35	VaginalBirth:	0
No	nonBreastMi	41.1428571	No	LessThan20	CSection	0
No	breastMilk	41	No	LessThan35	CSection	0
No	breastMilk	41	No	MoreThan35	VaginalBirth:	1
No	breastMilk	41.5714286	No	LessThan35	CSection	0
No	breastMilk	40.5714286	No	LessThan35	VaginalBirth:	0
No	breastMilk	39.5714286	No	LessThan35	VaginalBirth:	0
No	nonBreastMi	39	No	LessThan35	VaginalBirth:	1
No	nonBreastMi	40	No	LessThan20	VaginalBirth:	0
No	nonBreastMi	41.2857143	No	LessThan35	CSection	0
No	breastMilk	39.5714286	No	LessThan20	CSection	0
No	breastMilk	41.1428571	No	LessThan35	VaginalBirth:	0
No	breastMilk	39	No	LessThan35	CSection	1
No	nonBreastMi	37.7142857	No	LessThan35	VaginalBirth:	3
No	nonBreastMi	40	No	LessThan35	CSection	1
No	breastMilk	39.4285714	No	LessThan35	VaginalBirth:	0
No	breastMilk	37.7142857	No	LessThan35	VaginalBirth:	1
No	nonBreastMi	39.2857143	No	LessThan35	CSection	2
No	breastMilk	40.2857143	No	LessThan35	VaginalBirth:	0
No	breastMilk	38.5714286	No	LessThan35	CSection	1
No	breastMilk	39.7142857	No	LessThan35	VaginalBirth:	1
No	breastMilk	40.4285714	No	LessThan35	VaginalBirth:	2
No	nonBreastMi	41.4285714	No	LessThan35	VaginalBirth:	1
No	nonBreastMi	39.5714286	No	LessThan35	VaginalBirth:	1

No	breastMilk	38.1428571	No	LessThan35	CSection	1
No	nonBreastMi	39.7142857	No	LessThan35	VaginalBirths	1
No	breastMilk	39.5714286	No	LessThan35	VaginalBirths	1
No	breastMilk	40	No	MoreThan35	CSection	0
No	nonBreastMi	39.2857143	No	LessThan35	VaginalBirths	2
No	breastMilk	40.2857143	No	MoreThan35	VaginalBirths	1
No	breastMilk	36.4285714	No	MoreThan35	VaginalBirths	1
No	nonBreastMi	39.7142857	No	LessThan35	VaginalBirths	1
No	breastMilk	39.7142857	No	LessThan35	VaginalBirths	0
No	nonBreastMi	39.7142857	No	LessThan35	VaginalBirths	0
No	breastMilk	38.8571429	No	LessThan35	CSection	1
No	breastMilk	40.4285714	No	LessThan20	CSection	0
No	breastMilk	39.1428571	No	LessThan35	VaginalBirths	0
No	nonBreastMi	37.8571429	No	MoreThan35	CSection	1
No	breastMilk	39.1428571	No	LessThan35	VaginalBirths	0
No	nonBreastMi	40	No	LessThan35	VaginalBirths	1
No	nonBreastMi	38.5714286	No	MoreThan35	VaginalBirths	1
No	breastMilk	41.1428571	No	LessThan35	VaginalBirths	0
No	breastMilk	38	No	LessThan35	VaginalBirths	0
No	breastMilk	39.2857143	No	LessThan35	VaginalBirths	0
No	breastMilk	38	No	LessThan35	CSection	0
No	nonBreastMi	40.5714286	No	LessThan35	VaginalBirths	3
No	breastMilk	40.5714286	No	LessThan35	VaginalBirths	1
No	nonBreastMi	37.1428571	No	LessThan20	CSection	0
No	breastMilk	41.5714286	No	LessThan35	CSection	0
No	nonBreastMi	40.7142857	No	LessThan35	VaginalBirths	MoreThan3
No	breastMilk	40.2857143	No	LessThan35	VaginalBirths	0
No	nonBreastMi	39	No	LessThan35	CSection	1
No	breastMilk	35.5714286	No	LessThan35	VaginalBirths	MoreThan3
No	breastMilk	40.5714286	No	LessThan35	VaginalBirths	1
No	breastMilk	41.4285714	No	LessThan35	VaginalBirths	0
No	breastMilk	39.4285714	No	LessThan35	VaginalBirths	0
No	nonBreastMi	39.7142857	No	LessThan35	VaginalBirths	1
No	breastMilk	41.1428571	No	LessThan35	VaginalBirths	1
No	breastMilk	35.1428571	No	LessThan35	CSection	0
GestationalD	breastMilk	36.4285714	No	LessThan35	VaginalBirths	2
GestationalD	nonBreastMi	38.5714286	No	LessThan35	CSection	1
No	nonBreastMi	39.4285714	No	LessThan35	CSection	0
No	breastMilk	41.5714286	No	MoreThan35	VaginalBirths	2
No	breastMilk	38.1428571	No	LessThan35	VaginalBirths	0
No	breastMilk	41	No	LessThan35	VaginalBirths	0
GestationalD	breastMilk	38.4285714	No	LessThan35	CSection	2
No	breastMilk	38.2857143	No	LessThan35	CSection	3
No	breastMilk	41.2857143	No	LessThan35	VaginalBirths	0
No	breastMilk	37.1428571	No	MoreThan35	CSection	3
No	breastMilk	42.2857143	No	LessThan35	CSection	0
No	breastMilk	38.8571429	No	LessThan35	CSection	MoreThan3
No	breastMilk	37.2857143	No	LessThan35	VaginalBirths	1

GestationalD	breastMilk	38.5714286	No	LessThan35	CSection	1
No	breastMilk	39.8571429	No	LessThan35	CSection	1
No	breastMilk	37.7142857	No	MoreThan35	VaginalBirths	1
GestationalD	breastMilk	38.7142857	No	LessThan35	CSection	2
No	breastMilk	32.7142857	No	LessThan35	CSection	0
No	breastMilk	41.4285714	No	LessThan35	VaginalBirths	0
No	breastMilk	41.4285714	No	LessThan35	VaginalBirths	0
No	breastMilk	39.1428571	No	LessThan35	VaginalBirths	1
No	nonBreastMi	41.4285714	No	LessThan35	VaginalBirths	1
No	nonBreastMi	38	No	LessThan35	VaginalBirths	3
No	nonBreastMi	39.8571429	No	LessThan35	VaginalBirths	1
No	breastMilk	40.5714286	No	LessThan35	VaginalBirths	3
No	nonBreastMi	39.2857143	No	LessThan35	VaginalBirths	0
No	breastMilk	39.7142857	Yes	LessThan35	CSection	1
No	breastMilk	39.7142857	No	LessThan35	VaginalBirths	1
No	breastMilk	39.7142857	No	LessThan35	VaginalBirths	1
No	breastMilk	42.2857143	No	LessThan35	CSection	0
No	breastMilk	39.4285714	No	LessThan35	VaginalBirths	0
No	breastMilk	37.5714286	No	LessThan35	VaginalBirths	1
No	nonBreastMi	41.1428571	No	LessThan35	VaginalBirths	3
No	breastMilk	41.4285714	No	LessThan35	VaginalBirths	0
No	nonBreastMi	41.1428571	No	LessThan35	CSection	2
No	nonBreastMi	39	No	LessThan35	CSection	1
No	breastMilk	39.1428571	No	LessThan35	VaginalBirths	0
No	breastMilk	40.1428571	No	MoreThan35	VaginalBirths	3
No	breastMilk	37.8571429	No	LessThan35	VaginalBirths	2
No	nonBreastMi	41	No	LessThan35	VaginalBirths	MoreThan3
No	breastMilk	38.5714286	No	LessThan35	CSection	1
No	breastMilk	40.5714286	No	LessThan35	VaginalBirths	0
No	breastMilk	39.5714286	No	LessThan20	VaginalBirths	0
No	breastMilk	41.5714286	No	LessThan35	VaginalBirths	0
GestationalD	nonBreastMi	39.4285714	No	LessThan35	CSection	2
No	breastMilk	36	No	MoreThan35	CSection	3
No	nonBreastMi	41.1428571	No	LessThan35	CSection	MoreThan3
No	breastMilk	38.2857143	No	LessThan35	VaginalBirths	1
No	nonBreastMi	34.8571429	No	LessThan35	CSection	1
No	breastMilk	38.7142857	No	LessThan35	VaginalBirths	2
No	breastMilk	40.4285714	No	LessThan35	CSection	0
No	nonBreastMi	40.5714286	No	LessThan35	VaginalBirths	2
No	breastMilk	38	No	LessThan35	CSection	1
No	breastMilk	39.5714286	No	LessThan35	VaginalBirths	0
No	breastMilk	40.7142857	No	LessThan35	VaginalBirths	1
No	breastMilk	41.2857143	No	LessThan35	VaginalBirths	1
No	breastMilk	41.1428571	No	LessThan35	VaginalBirths	0
No	breastMilk	38.7142857	No	LessThan35	VaginalBirths	1
No	breastMilk	39.4285714	No	LessThan35	VaginalBirths	1
No	breastMilk	40.5714286	No	LessThan35	VaginalBirths	0
No	breastMilk	40.5714286	No	LessThan35	CSection	1

No	breastMilk	38.4285714	No	LessThan35	CSection	1
No	breastMilk	38.8571429	No	LessThan35	VaginalBirths	0
No	breastMilk	39	No	LessThan20	CSection	0
No	nonBreastMi	41.5714286	No	LessThan35	VaginalBirths	3
No	breastMilk	39.8571429	No	LessThan35	VaginalBirths	1
No	breastMilk	38.2857143	No	LessThan35	VaginalBirths	1
No	breastMilk	41.4285714	No	LessThan35	VaginalBirths	1
No	breastMilk	41.4285714	No	LessThan35	CSection	0
No	breastMilk	38.5714286	No	LessThan35	VaginalBirths	1
No	breastMilk	40.4285714	No	LessThan35	VaginalBirths	0
No	breastMilk	41.1428571	No	LessThan35	VaginalBirths	0
GestationalD	breastMilk	39.1428571	No	LessThan35	CSection	0
No	nonBreastMi	38	No	LessThan35	VaginalBirths	1
No	nonBreastMi	40.1428571	No	MoreThan35	VaginalBirths	MoreThan3
No	breastMilk	39.1428571	No	LessThan35	CSection	1
No	breastMilk	36.8571429	No	LessThan35	VaginalBirths	1
No	breastMilk	38.5714286	No	LessThan35	VaginalBirths	2
No	nonBreastMi	34.4285714	No	LessThan35	VaginalBirths	0
No	breastMilk	38.1428571	No	LessThan35	CSection	2
No	nonBreastMi	36.8571429	Yes	LessThan20	VaginalBirths	0
No	breastMilk	41	No	LessThan35	VaginalBirths	1
No	breastMilk	38.2857143	No	MoreThan35	VaginalBirths	3
No	breastMilk	41.4285714	No	MoreThan35	CSection	MoreThan3
No	nonBreastMi	39.7142857	No	LessThan35	VaginalBirths	1
No	nonBreastMi	39.2857143	No	LessThan35	VaginalBirths	1
No	nonBreastMi	39.4285714	No	LessThan20	CSection	0
No	breastMilk	39.1428571	No	LessThan35	CSection	0
No	nonBreastMi	39.2857143	No	LessThan35	CSection	0
No	nonBreastMi	38.7142857	No	LessThan35	CSection	2
No	breastMilk	37.7142857	No	LessThan35	VaginalBirths	1
No	nonBreastMi	38.2857143	No	MoreThan35	CSection	1
No	breastMilk	41.2857143	No	LessThan35	CSection	2
No	nonBreastMi	32.1428571	No	LessThan35	VaginalBirths	3
No	breastMilk	41.4285714	No	LessThan35	VaginalBirths	1
No	breastMilk	38.7142857	No	LessThan35	CSection	3
No	breastMilk	41.2857143	No	MoreThan35	VaginalBirths	3
No	nonBreastMi	38	Yes	LessThan35	VaginalBirths	0
No	breastMilk	41	No	LessThan35	VaginalBirths	2
No	breastMilk	41.7142857	No	LessThan35	VaginalBirths	1
No	breastMilk	40	No	LessThan35	VaginalBirths	0
No	breastMilk	40.4285714	No	LessThan35	VaginalBirths	0
No	breastMilk	40.2857143	No	LessThan35	VaginalBirths	0
No	nonBreastMi	39	No	LessThan35	CSection	2
No	breastMilk	38.1428571	No	LessThan35	VaginalBirths	1
No	nonBreastMi	39.2857143	No	LessThan35	CSection	1
No	breastMilk	40.1428571	No	LessThan35	VaginalBirths	0
No	breastMilk	40.5714286	No	LessThan35	VaginalBirths	3
No	breastMilk	38.4285714	No	LessThan35	CSection	1

No	breastMilk	41.1428571	No	LessThan35	CSection	MoreThan3
No	breastMilk	39.1428571	No	LessThan35	VaginalBirths	1
No	breastMilk	40	No	LessThan35	CSection	0
No	nonBreastMi	40.2857143	No	LessThan35	CSection	1
No	breastMilk	38.5714286	No	LessThan35	VaginalBirths	1
No	nonBreastMi	41.2857143	No	LessThan35	CSection	0
No	breastMilk	40.4285714	No	LessThan35	VaginalBirths	1
No	breastMilk	38.5714286	No	LessThan35	CSection	1
No	breastMilk	40.1428571	No	LessThan35	VaginalBirths	2
No	breastMilk	33.5714286	No	LessThan35	CSection	1
No	breastMilk	38.5714286	No	LessThan35	CSection	0
No	nonBreastMi	37.7142857	No	LessThan35	VaginalBirths	0
No	breastMilk	37.8571429	No	MoreThan35	VaginalBirths	1
No	breastMilk	39.4285714	No	LessThan35	CSection	1
No	breastMilk	40	No	LessThan35	VaginalBirths	2
No	breastMilk	37.2857143	No	MoreThan35	CSection	2
GestationalD	breastMilk	39.5714286	No	LessThan35	CSection	3
No	breastMilk	35.1428571	Yes	LessThan35	CSection	0
No	breastMilk	40.4285714	No	LessThan20	VaginalBirths	0
No	nonBreastMi	40.7142857	No	LessThan35	VaginalBirths	1
No	nonBreastMi	36.8571429	No	LessThan20	VaginalBirths	0
No	breastMilk	38.7142857	No	LessThan35	VaginalBirths	1
No	breastMilk	35.4285714	No	LessThan35	VaginalBirths	2
GestationalD	breastMilk	40.5714286	No	LessThan35	CSection	1
No	breastMilk	39.4285714	No	LessThan35	CSection	0
No	breastMilk	40.4285714	No	LessThan35	CSection	0
No	breastMilk	39.4285714	No	LessThan35	VaginalBirths	0
No	breastMilk	41.1428571	No	LessThan35	VaginalBirths	0
No	breastMilk	41.4285714	No	MoreThan35	VaginalBirths	2
No	nonBreastMi	41.4285714	No	MoreThan35	VaginalBirths	3
No	nonBreastMi	39.5714286	No	LessThan35	VaginalBirths	1
No	nonBreastMi	40.2857143	No	MoreThan35	VaginalBirths	3
No	breastMilk	37.5714286	No	LessThan35	VaginalBirths	0
No	nonBreastMi	40.7142857	No	LessThan35	CSection	0
No	breastMilk	35.5714286	No	MoreThan35	CSection	1
No	breastMilk	39.1428571	No	LessThan35	CSection	3
No	breastMilk	39.8571429	No	LessThan35	VaginalBirths	0
No	breastMilk	40.4285714	No	MoreThan35	VaginalBirths	MoreThan3
GestationalD	breastMilk	33.5714286	No	MoreThan35	VaginalBirths	3
No	breastMilk	40.1428571	No	LessThan35	CSection	0
No	breastMilk	40.7142857	No	LessThan20	VaginalBirths	0
GestationalD	breastMilk	40	No	LessThan35	CSection	0
No	breastMilk	37	No	LessThan35	VaginalBirths	0
No	breastMilk	39.7142857	No	LessThan35	VaginalBirths	2
GestationalD	breastMilk	39.4285714	No	LessThan35	VaginalBirths	0
No	nonBreastMi	41.4285714	No	LessThan35	VaginalBirths	0
No	nonBreastMi	39.1428571	No	LessThan35	VaginalBirths	1
No	breastMilk	34.2857143	No	LessThan35	CSection	1

GestationalD	breastMilk	36.2857143	No	LessThan35	CSection	0
No	breastMilk	39.4285714	No	LessThan35	VaginalBirths	1
No	breastMilk	41.4285714	No	MoreThan35	VaginalBirths	MoreThan3
No	breastMilk	41	No	LessThan35	VaginalBirths	0
No	breastMilk	40.1428571	No	LessThan35	CSection	0
No	nonBreastMi	39.1428571	No	LessThan35	CSection	2
No	breastMilk	39.2857143	No	LessThan35	VaginalBirths	2
No	breastMilk	40.5714286	No	LessThan35	VaginalBirths	1
GestationalD	breastMilk	38.4285714	No	LessThan35	CSection	0
No	breastMilk	41.1428571	No	LessThan20	VaginalBirths	0
No	breastMilk	39.1428571	No	LessThan35	CSection	1
No	breastMilk	39.2857143	No	LessThan35	CSection	2
No	breastMilk	39	No	MoreThan35	VaginalBirths	1
No	breastMilk	41.1428571	No	LessThan35	VaginalBirths	2
No	nonBreastMi	34.5714286	No	LessThan35	CSection	0
No	breastMilk	37.8571429	No	LessThan35	CSection	0
No	breastMilk	40.4285714	No	LessThan35	VaginalBirths	0
No	breastMilk	39.7142857	No	LessThan35	VaginalBirths	2
No	nonBreastMi	38.8571429	No	MoreThan35	CSection	2
GestationalD	nonBreastMi	40.2857143	No	LessThan35	VaginalBirths	0
No	breastMilk	40.1428571	No	LessThan35	VaginalBirths	0
No	breastMilk	38.7142857	No	MoreThan35	CSection	1
No	breastMilk	40.1428571	No	MoreThan35	CSection	2
No	breastMilk	38.8571429	No	LessThan35	VaginalBirths	0
No	breastMilk	37.4285714	No	LessThan35	VaginalBirths	1
No	breastMilk	38.2857143	No	LessThan35	VaginalBirths	1
No	breastMilk	39	No	LessThan35	VaginalBirths	1
No	breastMilk	38.7142857	No	LessThan35	VaginalBirths	3
No	breastMilk	40	No	LessThan35	VaginalBirths	0
No	breastMilk	39.5714286	No	LessThan35	VaginalBirths	0
No	breastMilk	39.4285714	No	LessThan35	VaginalBirths	0
No	breastMilk	40	No	LessThan35	VaginalBirths	1
GestationalD	breastMilk	39	No	MoreThan35	VaginalBirths	1
No	breastMilk	39	No	LessThan35	VaginalBirths	1
No	breastMilk	41.2857143	No	LessThan35	VaginalBirths	0
No	breastMilk	34.8571429	No	MoreThan35	CSection	1
No	breastMilk	40.8571429	No	LessThan35	CSection	1
No	breastMilk	40.8571429	No	LessThan35	VaginalBirths	3
GestationalD	breastMilk	38.2857143	No	MoreThan35	VaginalBirths	3
No	nonBreastMi	40.5714286	No	LessThan20	VaginalBirths	0
No	nonBreastMi	41.1428571	No	LessThan35	CSection	1
No	breastMilk	37.7142857	No	MoreThan35	VaginalBirths	3
No	breastMilk	40.5714286	No	MoreThan35	VaginalBirths	MoreThan3
No	breastMilk	38	No	LessThan35	VaginalBirths	1
No	breastMilk	40.5714286	No	MoreThan35	VaginalBirths	MoreThan3
No	breastMilk	39.4285714	No	LessThan35	CSection	2
No	breastMilk	38.4285714	No	LessThan35	CSection	2
No	breastMilk	39.1428571	No	LessThan35	VaginalBirths	0

No	breastMilk	40.1428571	No	LessThan20	VaginalBirths	0
No	breastMilk	40.2857143	No	LessThan35	VaginalBirths	3
No	nonBreastMi	38	No	LessThan35	VaginalBirths	3
No	nonBreastMi	41	No	LessThan35	CSection	0
GestationalD	nonBreastMi	38	No	LessThan35	CSection	0
No	nonBreastMi	38.8571429	No	LessThan35	VaginalBirths	0
No	breastMilk	41.2857143	No	LessThan35	CSection	0
No	breastMilk	40.8571429	No	LessThan35	CSection	0
No	nonBreastMi	40.2857143	No	LessThan35	CSection	1
No	breastMilk	39.7142857	No	MoreThan35	CSection	0
No	breastMilk	39.5714286	No	LessThan20	VaginalBirths	0
No	nonBreastMi	38.7142857	No	LessThan35	CSection	2
GestationalD	nonBreastMi	40	No	LessThan35	VaginalBirths	0
No	breastMilk	40.7142857	No	LessThan35	CSection	0
No	breastMilk	38.7142857	No	LessThan20	VaginalBirths	0
No	breastMilk	41.2857143	No	MoreThan35	CSection	1
No	breastMilk	40.8571429	No	LessThan35	VaginalBirths	0
No	nonBreastMi	40.5714286	No	LessThan35	VaginalBirths	0
No	breastMilk	40.1428571	No	LessThan35	VaginalBirths	1
No	breastMilk	39.5714286	No	LessThan35	CSection	2
No	nonBreastMi	40.1428571	No	LessThan35	CSection	1
No	nonBreastMi	39.2857143	No	LessThan35	CSection	2
No	breastMilk	41	No	LessThan35	VaginalBirths	1
No	breastMilk	40.7142857	No	LessThan35	CSection	0
No	breastMilk	41.1428571	No	LessThan35	VaginalBirths	0
No	breastMilk	38.7142857	No	LessThan35	CSection	1
No	breastMilk	41.4285714	No	LessThan35	VaginalBirths	3
No	breastMilk	37.2857143	No	MoreThan35	CSection	0
No	breastMilk	39	No	LessThan35	CSection	0
No	nonBreastMi	40.7142857	No	MoreThan35	CSection	2
No	breastMilk	38.2857143	No	LessThan35	CSection	1
No	breastMilk	40.4285714	No	LessThan35	VaginalBirths	0
No	nonBreastMi	40.7142857	No	LessThan35	VaginalBirths	3
GestationalD	breastMilk	35.2857143	No	LessThan35	CSection	0
No	breastMilk	39.2857143	No	LessThan35	CSection	1
No	breastMilk	40	No	LessThan35	CSection	2
No	breastMilk	39	No	LessThan35	CSection	1
No	breastMilk	38.1428571	No	LessThan35	VaginalBirths	1
No	breastMilk	37.1428571	No	LessThan35	VaginalBirths	0
No	breastMilk	41.2857143	No	LessThan35	VaginalBirths	0
No	breastMilk	40.4285714	No	MoreThan35	VaginalBirths	2
No	breastMilk	39	No	LessThan35	CSection	0
No	nonBreastMi	39.5714286	No	LessThan35	VaginalBirths	0
No	nonBreastMi	40.2857143	No	LessThan35	VaginalBirths	1
No	nonBreastMi	39.5714286	No	MoreThan35	CSection	0
No	breastMilk	39.4285714	No	MoreThan35	CSection	3
No	breastMilk	37.4285714	No	LessThan35	VaginalBirths	1
No	breastMilk	37.5714286	No	LessThan35	CSection	0

No	breastMilk	41.1428571	No	LessThan35	VaginalBirths	1
No	breastMilk	41.2857143	No	LessThan35	VaginalBirths	1
No	breastMilk	39	No	LessThan35	CSection	1
No	nonBreastMi	41.5714286	No	LessThan35	CSection	0
No	breastMilk	39	No	LessThan35	CSection	1
No	breastMilk	40.7142857	No	LessThan35	VaginalBirths	0
No	breastMilk	41	No	LessThan35	CSection	0
No	nonBreastMi	38.5714286	No	LessThan20	VaginalBirths	0
No	breastMilk	39.8571429	No	LessThan35	CSection	0
No	nonBreastMi	41.4285714	No	LessThan35	VaginalBirths	2
No	nonBreastMi	33.8571429	No	LessThan35	VaginalBirths	0
GestationalD	breastMilk	41.1428571	No	LessThan35	CSection	0
No	breastMilk	39.5714286	No	LessThan35	VaginalBirths	1
GestationalD	breastMilk	37.7142857	No	LessThan35	VaginalBirths	0
No	breastMilk	40.4285714	No	LessThan35	VaginalBirths	0
No	breastMilk	41.4285714	No	LessThan35	VaginalBirths	1
No	breastMilk	38.5714286	No	LessThan35	CSection	0
No	nonBreastMi	39.8571429	No	MoreThan35	VaginalBirths	2
GestationalD	breastMilk	41.1428571	No	LessThan35	CSection	0
PreExisting	breastMilk	33.4285714	No	LessThan35	CSection	0
No	breastMilk	41.5714286	No	MoreThan35	VaginalBirths	1
No	breastMilk	39.5714286	No	MoreThan35	VaginalBirths	1
No	nonBreastMi	40.2857143	No	LessThan35	VaginalBirths	0
No	breastMilk	38.5714286	No	LessThan35	VaginalBirths	2
No	breastMilk	41.2857143	No	LessThan35	CSection	1
No	nonBreastMi	38.1428571	No	MoreThan35	CSection	1
No	breastMilk	38.1428571	Yes	MoreThan35	VaginalBirths	MoreThan3
GestationalD	breastMilk	37	No	LessThan35	CSection	0
No	breastMilk	39.8571429	No	LessThan35	VaginalBirths	2
No	nonBreastMi	41.2857143	No	LessThan35	VaginalBirths	2
No	breastMilk	40	No	LessThan35	VaginalBirths	1
No	breastMilk	40.8571429	No	LessThan35	VaginalBirths	1
No	breastMilk	39	No	MoreThan35	VaginalBirths	MoreThan3
No	breastMilk	38.5714286	No	LessThan35	CSection	1
No	nonBreastMi	38.7142857	No	MoreThan35	CSection	1
No	breastMilk	36.8571429	No	LessThan35	VaginalBirths	0
No	breastMilk	39.1428571	No	LessThan35	CSection	1
No	nonBreastMi	40.7142857	No	MoreThan35	VaginalBirths	1
No	breastMilk	40.5714286	No	LessThan35	CSection	0
No	nonBreastMi	40.5714286	No	LessThan35	VaginalBirths	0
No	breastMilk	40.4285714	Yes	LessThan20	VaginalBirths	0
No	breastMilk	41.2857143	No	MoreThan35	CSection	0
No	nonBreastMi	37.5714286	No	LessThan35	CSection	2
No	nonBreastMi	40.7142857	No	LessThan35	VaginalBirths	0
No	breastMilk	39.7142857	No	LessThan35	VaginalBirths	1
No	breastMilk	37.7142857	No	LessThan35	CSection	0
No	breastMilk	34.2857143	No	LessThan35	CSection	0
No	breastMilk	37.8571429	No	LessThan35	VaginalBirths	0

No	nonBreastMi	38.8571429	No	LessThan20	VaginalBirths	0
No	breastMilk	41.2857143	No	LessThan35	CSection	0
No	nonBreastMi	41	No	LessThan35	CSection	0
No	breastMilk	40.4285714	No	LessThan35	VaginalBirths	1
No	breastMilk	41.4285714	No	LessThan35	VaginalBirths	1
No	breastMilk	40.8571429	No	LessThan35	VaginalBirths	0
No	breastMilk	40.5714286	No	MoreThan35	VaginalBirths	MoreThan3
No	nonBreastMi	39	No	LessThan35	VaginalBirths	3
No	breastMilk	37.8571429	No	LessThan35	VaginalBirths	0
No	nonBreastMi	39.1428571	No	MoreThan35	VaginalBirths	MoreThan3
No	breastMilk	41.2857143	No	LessThan35	VaginalBirths	0
No	breastMilk	41.2857143	No	LessThan35	VaginalBirths	0
No	breastMilk	38.8571429	No	MoreThan35	VaginalBirths	2
No	nonBreastMi	38.5714286	No	LessThan35	CSection	0
PreExisting	breastMilk	38	No	LessThan35	VaginalBirths	MoreThan3
No	breastMilk	39.8571429	No	LessThan35	VaginalBirths	0
No	breastMilk	40.1428571	No	LessThan35	VaginalBirths	1
No	breastMilk	40.1428571	No	LessThan35	CSection	0
No	breastMilk	40.2857143	No	LessThan35	VaginalBirths	1
GestationalD	breastMilk	35.2857143	No	MoreThan35	VaginalBirths	3
No	breastMilk	38.7142857	No	LessThan35	VaginalBirths	0
No	breastMilk	37.1428571	No	LessThan35	VaginalBirths	MoreThan3
No	breastMilk	39.4285714	No	LessThan35	VaginalBirths	0
GestationalD	breastMilk	40.1428571	No	LessThan35	VaginalBirths	3
No	nonBreastMi	38.8571429	No	LessThan35	VaginalBirths	0
No	breastMilk	40.5714286	No	LessThan35	VaginalBirths	0
No	breastMilk	40.1428571	No	LessThan35	VaginalBirths	1
No	nonBreastMi	39.7142857	No	LessThan35	CSection	0
No	breastMilk	41.5714286	No	LessThan35	VaginalBirths	0
No	breastMilk	40.1428571	Yes	LessThan35	VaginalBirths	2
No	breastMilk	41	No	LessThan35	VaginalBirths	0
No	breastMilk	38.5714286	No	LessThan35	CSection	2
No	nonBreastMi	40.4285714	No	LessThan35	VaginalBirths	0
No	breastMilk	34.4285714	No	LessThan35	VaginalBirths	0
No	breastMilk	40.8571429	No	LessThan35	VaginalBirths	0
GestationalD	breastMilk	39	No	MoreThan35	CSection	0
PreExisting	breastMilk	35.4285714	No	LessThan35	VaginalBirths	0
No	breastMilk	37	No	LessThan35	VaginalBirths	1
PreExisting	breastMilk	38.1428571	No	LessThan35	CSection	1
No	nonBreastMi	39.1428571	No	MoreThan35	CSection	2
No	breastMilk	38.5714286	No	LessThan35	CSection	2
No	nonBreastMi	40.4285714	No	LessThan35	VaginalBirths	3
No	breastMilk	39	No	LessThan20	VaginalBirths	0
No	breastMilk	38.8571429	No	LessThan35	CSection	1
No	breastMilk	41.5714286	No	LessThan35	VaginalBirths	2
No	breastMilk	39.7142857	No	LessThan35	CSection	0
No	breastMilk	39	No	LessThan35	CSection	1
No	breastMilk	39.4285714	No	LessThan35	VaginalBirths	0

No	breastMilk	41.5714286	No	LessThan35	VaginalBirths	2
No	breastMilk	38.1428571	No	LessThan35	CSection	1
No	nonBreastMi	41.1428571	No	LessThan20	CSection	0
No	breastMilk	38.1428571	No	MoreThan35	CSection	0
No	nonBreastMi	40	No	LessThan35	CSection	2
No	breastMilk	40.1428571	No	LessThan35	VaginalBirths	0
No	nonBreastMi	33.5714286	Yes	LessThan20	VaginalBirths	0
No	breastMilk	41.4285714	No	LessThan35	VaginalBirths	0
No	nonBreastMi	38.8571429	No	MoreThan35	CSection	0
No	breastMilk	41	No	LessThan35	CSection	0
No	breastMilk	37.5714286	No	LessThan35	CSection	2
No	breastMilk	41.4285714	No	LessThan35	VaginalBirths	0
No	breastMilk	40.5714286	No	LessThan35	VaginalBirths	1
No	breastMilk	37.5714286	No	LessThan35	CSection	0
No	nonBreastMi	38.8571429	No	LessThan35	CSection	2
No	breastMilk	40.2857143	No	LessThan20	CSection	0
No	breastMilk	40.1428571	No	LessThan35	VaginalBirths	1
No	nonBreastMi	41.1428571	No	MoreThan35	VaginalBirths	MoreThan3
No	breastMilk	38	No	LessThan20	CSection	1
No	breastMilk	35.4285714	No	LessThan35	CSection	0
No	breastMilk	39.5714286	No	LessThan35	CSection	0
No	breastMilk	39.7142857	No	LessThan35	VaginalBirths	1
No	breastMilk	39.4285714	No	LessThan35	VaginalBirths	1
No	breastMilk	38	No	LessThan20	VaginalBirths	0
No	breastMilk	40	No	LessThan35	VaginalBirths	1
No	breastMilk	40	No	LessThan35	VaginalBirths	0
No	breastMilk	38.4285714	No	LessThan35	CSection	2
No	breastMilk	40.1428571	No	LessThan35	VaginalBirths	1
No	breastMilk	39	No	LessThan35	CSection	1
No	breastMilk	37.4285714	No	LessThan35	VaginalBirths	3
No	nonBreastMi	39.4285714	No	LessThan35	VaginalBirths	1
No	breastMilk	39.2857143	No	LessThan35	VaginalBirths	1
No	nonBreastMi	38.1428571	No	LessThan35	VaginalBirths	0
No	breastMilk	39.1428571	No	LessThan35	VaginalBirths	0
No	breastMilk	39.5714286	No	MoreThan35	VaginalBirths	0
No	nonBreastMi	40.7142857	No	LessThan35	VaginalBirths	3
No	breastMilk	41.1428571	No	LessThan35	VaginalBirths	0
No	nonBreastMi	40	No	LessThan35	VaginalBirths	2
No	nonBreastMi	37.1428571	No	LessThan35	VaginalBirths	1
No	breastMilk	40.8571429	No	LessThan35	VaginalBirths	0
No	breastMilk	39.8571429	No	LessThan35	VaginalBirths	0
No	breastMilk	37.5714286	No	LessThan20	VaginalBirths	0
No	breastMilk	40.8571429	No	LessThan35	VaginalBirths	0
No	breastMilk	40.2857143	No	LessThan35	VaginalBirths	0
GestationalD	breastMilk	40.7142857	No	MoreThan35	CSection	2
No	nonBreastMi	41.1428571	No	LessThan35	VaginalBirths	3
No	breastMilk	38.7142857	No	LessThan35	VaginalBirths	2
No	nonBreastMi	40.5714286	No	LessThan35	VaginalBirths	0

No	nonBreastMi	41.2857143	No	LessThan35	VaginalBirths		0
No	breastMilk	40.5714286	Yes	LessThan35	VaginalBirths		0
No	breastMilk	39.5714286	No	LessThan35	CSection		0
No	breastMilk	38.7142857	No	LessThan35	CSection		1
No	breastMilk	36.4285714	No	LessThan35	VaginalBirths		0
No	nonBreastMi	38.7142857	No	LessThan35	CSection		0
No	breastMilk	38.5714286	No	LessThan35	CSection		1
No	breastMilk	36.7142857	No	LessThan35	VaginalBirths	MoreThan3	
No	breastMilk	37.4285714	No	LessThan35	CSection		0
No	breastMilk	40.1428571	No	LessThan35	VaginalBirths		0
No	breastMilk	38.7142857	No	LessThan20	VaginalBirths		0
No	breastMilk	40	No	LessThan35	CSection		0
No	breastMilk	41.2857143	No	LessThan35	VaginalBirths		2
No	breastMilk	39.1428571	No	MoreThan35	VaginalBirths		0
No	nonBreastMi	39	No	LessThan35	CSection		3
No	breastMilk	41.2857143	No	MoreThan35	VaginalBirths		0
No	breastMilk	41.1428571	No	LessThan35	VaginalBirths		0
No	breastMilk	41.4285714	No	LessThan35	VaginalBirths		2
No	breastMilk	36.4285714	No	LessThan35	CSection		1
No	breastMilk	39.5714286	No	LessThan35	CSection		1
No	breastMilk	34	No	LessThan35	CSection		0
No	breastMilk	33.4285714	No	LessThan35	CSection		0
No	nonBreastMi	33.1428571	No	LessThan35	CSection	MoreThan3	
No	breastMilk	39.8571429	No	LessThan20	CSection		0
No	breastMilk	39.7142857	No	LessThan35	VaginalBirths		2
No	breastMilk	40	No	MoreThan35	CSection		2
No	breastMilk	40	No	MoreThan35	CSection	MoreThan3	
No	nonBreastMi	40.4285714	No	LessThan20	VaginalBirths		0
No	breastMilk	40.4285714	No	LessThan35	VaginalBirths		3
No	breastMilk	40	No	LessThan35	VaginalBirths		0
No	breastMilk	41.5714286	No	LessThan35	CSection		0
No	breastMilk	40.8571429	No	LessThan35	VaginalBirths		1
No	breastMilk	41.4285714	No	LessThan35	VaginalBirths		0
No	nonBreastMi	38.8571429	No	LessThan35	CSection		3
No	breastMilk	38.4285714	No	LessThan20	VaginalBirths		0
No	breastMilk	38	No	LessThan35	VaginalBirths		2
No	breastMilk	41.2857143	No	LessThan35	VaginalBirths		2
No	breastMilk	41	No	LessThan35	CSection		0
No	breastMilk	40.1428571	No	MoreThan35	VaginalBirths		1
GestationalD	breastMilk	35.5714286	No	LessThan35	VaginalBirths		3
No	breastMilk	40	No	LessThan35	VaginalBirths		3
No	breastMilk	40.7142857	No	LessThan35	CSection		0
No	nonBreastMi	39.1428571	No	MoreThan35	CSection	MoreThan3	
No	breastMilk	37.8571429	No	LessThan35	VaginalBirths		0
No	breastMilk	41.4285714	No	LessThan35	CSection		0
No	breastMilk	40.2857143	No	LessThan35	VaginalBirths		3
No	breastMilk	38.8571429	No	LessThan35	VaginalBirths		0
GestationalD	breastMilk	38.5714286	No	LessThan35	VaginalBirths		0

No	breastMilk	40.1428571	No	LessThan35	VaginalBirths	1
No	nonBreastMi	38.1428571	No	MoreThan35	CSection	2
No	breastMilk	40.5714286	No	LessThan35	CSection	0
No	nonBreastMi	35.7142857	No	LessThan35	VaginalBirths	3
No	breastMilk	40.7142857	No	LessThan35	VaginalBirths	1
No	breastMilk	41.4285714	No	LessThan35	VaginalBirths	1
No	breastMilk	35.8571429	No	MoreThan35	CSection	1
No	breastMilk	39.8571429	No	LessThan35	VaginalBirths	0
No	nonBreastMi	41.1428571	No	LessThan35	VaginalBirths	0
No	breastMilk	40.1428571	No	LessThan35	VaginalBirths	2
No	breastMilk	41.4285714	No	LessThan35	CSection	0
No	breastMilk	41	No	LessThan35	CSection	1
No	breastMilk	40.2857143	No	LessThan35	VaginalBirths	3
No	breastMilk	34.4285714	No	LessThan35	CSection	1
No	breastMilk	40.1428571	No	LessThan35	VaginalBirths	1

prematurePr	seasonOfBirt	sexObstData	smokeBookir	threatenedPr	typeConcepti	zScoreBW
No	Autumn	Female	Yes	No	Spontaneous	0.38406788
No	Spring	Female	No	No	Spontaneous	0.42703247
No	Spring	Male	No	No	Spontaneous	-0.5251316
No	Spring	Female	Yes	No	Spontaneous	-0.9658054
No	Spring	Female	No	No	Spontaneous	-1.7696291
No	Summer	Male	No	No	Spontaneous	0.16802664
No	Spring	Male	No	No	Spontaneous	0.37240701
No	Winter	Male	No	No	Spontaneous	1.62221715
No	Spring	Male	No	No	Spontaneous	0.17374897
No	Autumn	Male	No	No	Spontaneous	-0.4430571
No	Spring	Male	No	No	Spontaneous	-0.7172751
No	Autumn	Male	No	No	Spontaneous	1.11782502
No	Autumn	Female	Yes	No	Spontaneous	-1.8388443
No	Spring	Female	No	No	Spontaneous	1.06389636
No	Autumn	Male	No	No	Assisted	-0.1712861
No	Summer	Male	No	No	Assisted	0.12103617
No	Summer	Male	Yes	No	Spontaneous	0.20135463
No	Winter	Female	No	No	Spontaneous	2.32221166
No	Summer	Male	No	No	Spontaneous	-0.7234272
No	Summer	Male	No	No	Spontaneous	0.84917029
No	Autumn	Male	No	No	Spontaneous	1.60453228
No	Summer	Female	No	No	Spontaneous	-0.54468
No	Winter	Female	Yes	No	Spontaneous	0.20702366
No	Summer	Female	No	No	Spontaneous	0.96564551
No	Spring	Female	No	No	Spontaneous	-0.574577
No	Winter	Female	No	No	Spontaneous	-0.6720674
No	Winter	Male	Yes	No	Spontaneous	-0.2754582
No	Winter	Male	No	No	Spontaneous	0.98060831
No	Summer	Female	No	No	Spontaneous	-0.8172094
No	Summer	Male	No	No	Spontaneous	-0.3949178
No	Autumn	Male	No	No	Spontaneous	-0.7725398
No	Summer	Female	No	No	Spontaneous	-0.2653621
Yes	Winter	Male	No	No	Spontaneous	-0.4348975
No	Winter	Female	No	No	Spontaneous	0.77379408
No	Summer	Female	No	TplAdmitted	Spontaneous	0.32435111
No	Winter	Male	No	No	Spontaneous	-0.1250086
No	Winter	Male	No	No	Spontaneous	-0.7660725
No	Autumn	Male	No	No	Spontaneous	0.43276089
No	Summer	Male	No	No	Spontaneous	-0.2750581
No	Winter	Male	No	No	Spontaneous	-0.8422777
No	Winter	Male	No	No	Spontaneous	-0.1197958
No	Summer	Female	No	No	Spontaneous	-0.9226028
No	Summer	Female	No	No	Spontaneous	0.50924452
No	Spring	Male	No	No	Assisted	0.99449175
No	Summer	Female	No	No	Spontaneous	0.40352277
No	Winter	Female	No	No	Spontaneous	-1.0221199
No	Winter	Male	Yes	No	Spontaneous	0.27949742

No	Autumn	Male	No	No	Spontaneous	0.18418119
No	Winter	Male	No	No	Spontaneous	-0.258344
No	Winter	Female	Yes	No	Spontaneous	-0.7412896
No	Winter	Male	Yes	No	Spontaneous	-2.5330209
No	Spring	Male	Yes	No	Spontaneous	-0.9717196
No	Spring	Male	No	No	Spontaneous	-0.4108903
No	Spring	Female	No	No	Spontaneous	0.13202718
No	Spring	Female	No	No	Assisted	-0.2315368
No	Spring	Female	No	No	Spontaneous	-0.5654003
No	Autumn	Male	No	No	Spontaneous	-0.8219833
No	Autumn	Male	No	No	Spontaneous	-0.2700897
No	Spring	Male	No	TplAdmitted	Spontaneous	0.34020374
No	Winter	Male	No	No	Assisted	-1.6399343
No	Spring	Male	No	No	Spontaneous	-0.0164076
No	Summer	Male	No	No	Spontaneous	0.24803806
No	Winter	Female	No	No	Spontaneous	0.86622114
No	Spring	Female	No	No	Spontaneous	-0.7189476
No	Winter	Female	Yes	No	Spontaneous	0.67878177
No	Winter	Male	No	No	Spontaneous	2.2775382
No	Winter	Female	No	No	Assisted	-0.1085181
No	Autumn	Female	Yes	No	Spontaneous	-0.8880297
No	Spring	Male	No	No	Spontaneous	0.4733667
No	Winter	Female	No	No	Spontaneous	1.2378776
No	Autumn	Female	No	No	Spontaneous	0.14018964
No	Autumn	Male	No	No	Spontaneous	-0.3205237
No	Winter	Female	No	No	Spontaneous	-0.9589696
No	Winter	Male	No	No	Spontaneous	-0.590869
No	Spring	Female	No	No	Spontaneous	-2.0808309
No	Winter	Male	Yes	No	Spontaneous	-0.7823253
No	Autumn	Female	No	TplAdmitted	Spontaneous	-0.0755417
No	Summer	Male	No	No	Spontaneous	0.01487722
Yes	Winter	Male	No	TplAdmitted	Spontaneous	0.27602206
No	Summer	Female	No	No	Spontaneous	-0.2723608
No	Spring	Female	No	No	Spontaneous	-0.2512051
No	Summer	Male	No	TplAdmitted	Spontaneous	-0.2597461
No	Summer	Male	No	No	Spontaneous	1.73757603
No	Summer	Male	No	No	Spontaneous	0.39201433
No	Autumn	Female	No	No	Spontaneous	0.74818372
No	Summer	Male	No	No	Spontaneous	-0.2526242
No	Summer	Female	No	No	Spontaneous	0.3624711
No	Winter	Female	Yes	No	Spontaneous	0.98146764
No	Autumn	Male	No	No	Spontaneous	-0.5389224
No	Summer	Male	No	No	Spontaneous	-0.8553564
Yes	Autumn	Male	No	No	Spontaneous	2.21426893
No	Autumn	Male	No	No	Spontaneous	0.15617159
No	Winter	Male	No	No	Spontaneous	-0.2597461
Yes	Summer	Female	No	No	Spontaneous	1.00117726
No	Autumn	Female	No	No	Spontaneous	0.83185429

No	Winter	Female	No	No	Spontaneous	2.09508838
No	Spring	Male	No	No	Spontaneous	-1.0429114
No	Summer	Female	No	No	Spontaneous	1.78232953
No	Winter	Male	No	No	Spontaneous	-0.1256998
No	Winter	Male	No	No	Spontaneous	-0.1336041
No	Winter	Female	No	No	Spontaneous	-0.7902517
No	Winter	Female	No	No	Spontaneous	0.74176653
No	Summer	Male	No	No	Spontaneous	-0.4165279
No	Autumn	Male	Yes	No	Spontaneous	0.52188638
No	Winter	Male	No	No	Spontaneous	0.2400321
No	Spring	Male	No	No	Spontaneous	-1.2377527
No	Summer	Male	No	No	Spontaneous	0.11079154
No	Autumn	Male	No	No	Spontaneous	2.26112921
No	Summer	Female	Yes	No	Spontaneous	-1.0551269
No	Spring	Female	No	No	Spontaneous	-0.3661156
No	Autumn	Male	No	No	Spontaneous	-0.9348619
No	Autumn	Female	No	No	Spontaneous	-1.2106737
No	Spring	Female	No	No	Spontaneous	0.40730619
No	Autumn	Male	No	No	Spontaneous	1.57852302
No	Autumn	Female	Yes	No	Spontaneous	2.44115872
No	Summer	Female	No	No	Spontaneous	2.10699331
No	Spring	Female	No	No	Spontaneous	0.80578664
No	Autumn	Male	No	No	Spontaneous	0.04918756
No	Winter	Male	No	No	Spontaneous	-0.6994785
No	Summer	Male	No	No	Spontaneous	0.16508549
No	Spring	Female	No	No	Spontaneous	-0.5073378
No	Winter	Male	No	No	Spontaneous	-0.4811869
No	Spring	Female	No	No	Spontaneous	-0.8788741
No	Autumn	Male	No	No	Spontaneous	1.03693759
No	Winter	Male	No	No	Spontaneous	-0.7913234
No	Spring	Male	Yes	No	Spontaneous	-2.0401976
No	Winter	Female	No	No	Spontaneous	-1.3743953
No	Summer	Female	No	No	Spontaneous	-1.0748854
No	Autumn	Male	No	No	Spontaneous	0.68232232
No	Winter	Male	No	No	Spontaneous	-0.4885372
No	Autumn	Female	No	No	Spontaneous	-1.3107617
No	Winter	Male	Yes	No	Spontaneous	1.84552269
No	Summer	Female	Yes	No	Spontaneous	-0.5677004
No	Autumn	Male	No	No	Spontaneous	0.51094557
No	Summer	Male	No	No	Spontaneous	-1.5096171
No	Winter	Male	No	No	Spontaneous	-0.5211453
No	Winter	Male	No	No	Spontaneous	-1.8656494
No	Autumn	Female	No	No	Spontaneous	0.85261718
No	Winter	Male	No	No	Spontaneous	-0.804996
No	Winter	Male	No	No	Spontaneous	0.17943484
No	Autumn	Male	No	No	Spontaneous	-0.2089977
No	Spring	Female	No	No	Spontaneous	-0.2217092
No	Summer	Female	No	No	Spontaneous	0.33388253

No	Winter	Female	No	No	Spontaneous	0.74020815
No	Autumn	Male	Yes	No	Spontaneous	-0.2910022
No	Summer	Female	No	No	Spontaneous	-0.7990922
No	Winter	Male	No	No	Spontaneous	-0.1772713
No	Winter	Male	No	No	Spontaneous	1.15012501
No	Autumn	Male	No	No	Spontaneous	0.40089739
No	Autumn	Female	No	No	Spontaneous	0.01519772
No	Autumn	Female	No	No	Spontaneous	0.49912358
Yes	Summer	Male	Yes	No	Spontaneous	-0.8500673
No	Autumn	Male	Yes	No	Spontaneous	-0.4135878
No	Winter	Male	Yes	No	Spontaneous	0.05314013
No	Spring	Male	No	No	Spontaneous	1.05087787
No	Winter	Female	No	No	Spontaneous	2.33111988
No	Summer	Male	No	No	Spontaneous	0.36528957
No	Winter	Female	No	No	Spontaneous	-0.506491
No	Winter	Female	Yes	No	Spontaneous	-0.3708374
Yes	Summer	Male	No	TplAdmitted	Spontaneous	0.08336961
No	Autumn	Male	No	No	Assisted	0.52641909
No	Autumn	Male	No	No	Spontaneous	-0.5076621
No	Summer	Male	No	No	Spontaneous	2.27036673
No	Summer	Female	No	No	Spontaneous	1.77035896
No	Summer	Female	No	No	Spontaneous	0.26809589
No	Summer	Male	No	No	Assisted	-1.3007232
No	Spring	Male	No	No	Spontaneous	1.39020698
No	Summer	Male	Yes	No	Spontaneous	-1.6069656
No	Autumn	Male	No	No	Spontaneous	-1.3310287
No	Winter	Male	No	No	Spontaneous	0.19131039
No	Summer	Male	Yes	No	Spontaneous	-0.3321583
No	Winter	Female	No	No	Assisted	0.65240651
No	Spring	Female	No	No	Spontaneous	0.39944118
No	Spring	Male	Yes	No	Spontaneous	-0.8171708
No	Spring	Female	No	No	Spontaneous	0.63838945
No	Autumn	Female	No	No	Assisted	1.12008203
No	Winter	Male	No	No	Spontaneous	-0.5116305
No	Summer	Female	Yes	No	Spontaneous	-0.8076221
No	Winter	Male	No	No	Spontaneous	0.94932664
No	Spring	Male	No	No	Spontaneous	1.69389295
No	Autumn	Male	No	No	Spontaneous	-1.4245214
No	Summer	Male	No	No	Spontaneous	-1.2645735
No	Summer	Female	No	No	Spontaneous	0.16030143
No	Summer	Female	No	No	Spontaneous	0.27396327
No	Summer	Male	Yes	No	Spontaneous	1.06482499
No	Spring	Female	No	TplAdmitted	Spontaneous	1.54225321
No	Winter	Male	No	No	Spontaneous	0.01554249
No	Spring	Male	No	No	Spontaneous	0.52660193
No	Winter	Female	No	No	Spontaneous	1.2101181
No	Summer	Male	Yes	No	Spontaneous	-1.2175022
No	Autumn	Male	No	No	Spontaneous	-0.6990669

No	Autumn	Male	No	No	Spontaneous	-1.1360719
No	Spring	Female	No	No	Spontaneous	0.27440471
No	Summer	Female	Yes	No	Spontaneous	-0.1104004
No	Winter	Female	Yes	No	Spontaneous	-1.4073821
No	Autumn	Female	No	No	Spontaneous	-1.1340505
No	Summer	Male	No	No	Spontaneous	0.7719311
No	Winter	Male	No	No	Spontaneous	1.50170363
No	Winter	Female	No	No	Spontaneous	-0.0689152
No	Autumn	Female	No	No	Spontaneous	-0.8750895
No	Winter	Male	Yes	No	Spontaneous	0.18867393
No	Winter	Male	No	No	Spontaneous	0.93237024
No	Summer	Female	No	No	Spontaneous	1.58719926
No	Winter	Male	Yes	No	Spontaneous	0.15643168
No	Winter	Male	No	No	Spontaneous	-1.9467653
Yes	Winter	Male	Yes	No	Spontaneous	-0.4846832
No	Winter	Male	No	No	Spontaneous	0.67285067
No	Spring	Female	No	No	Spontaneous	0.34936432
No	Summer	Male	Yes	No	Spontaneous	-1.414148
No	Winter	Male	No	No	Spontaneous	-0.8555214
No	Winter	Female	Yes	No	Spontaneous	-2.095081
No	Winter	Male	Yes	No	Spontaneous	-0.3874676
No	Spring	Male	No	No	Spontaneous	-0.014215
No	Winter	Female	No	No	Spontaneous	0.9710629
No	Spring	Male	No	No	Spontaneous	1.32712918
No	Winter	Male	No	No	Spontaneous	-0.2688187
No	Spring	Male	No	No	Spontaneous	-1.4688382
Yes	Winter	Female	No	No	Spontaneous	-1.602773
No	Winter	Female	No	No	Spontaneous	-0.2531814
No	Summer	Male	No	No	Spontaneous	-0.1635139
Yes	Winter	Female	Yes	No	Spontaneous	-0.5064
No	Spring	Male	No	No	Spontaneous	-0.3315742
No	Winter	Female	No	No	Spontaneous	0.59039149
No	Autumn	Male	Yes	No	Spontaneous	0.23934391
No	Winter	Male	Yes	No	Spontaneous	0.8475693
No	Summer	Female	No	No	Spontaneous	-0.2158315
No	Autumn	Male	Yes	No	Spontaneous	-0.2783947
No	Summer	Male	No	TplAdmitted	Spontaneous	0.81894786
No	Spring	Male	No	No	Spontaneous	0.17568733
No	Spring	Male	No	No	Spontaneous	-0.6436341
No	Summer	Female	No	No	Spontaneous	0.43321887
No	Spring	Female	No	No	Spontaneous	0.82843921
No	Winter	Male	No	TplAdmitted	Spontaneous	0.41351762
No	Spring	Male	No	No	Spontaneous	-0.7763752
No	Autumn	Female	No	No	Spontaneous	-0.496994
No	Autumn	Female	No	No	Spontaneous	-0.1221851
No	Autumn	Male	No	No	Spontaneous	-1.0417573
No	Summer	Male	Yes	No	Spontaneous	-0.9685895
Yes	Autumn	Female	Yes	No	Spontaneous	-0.0476292

No	Autumn	Female	No	No	Spontaneous	-0.5495804
No	Autumn	Female	Yes	No	Spontaneous	-1.6674258
No	Spring	Male	No	No	Spontaneous	-1.1033199
No	Summer	Male	No	No	Spontaneous	-0.8992197
No	Spring	Male	Yes	No	Spontaneous	1.55096464
No	Autumn	Male	No	No	Spontaneous	-1.7469266
No	Winter	Male	Yes	No	Spontaneous	0.3332343
No	Summer	Male	No	No	Spontaneous	-0.0018111
No	Summer	Female	No	No	Spontaneous	1.02292602
No	Winter	Male	No	No	Spontaneous	0.22975702
No	Autumn	Male	No	No	Spontaneous	1.10742912
No	Summer	Female	No	No	Spontaneous	-0.2953245
No	Autumn	Female	No	No	Spontaneous	0.81000448
No	Summer	Male	No	No	Spontaneous	0.72290655
No	Autumn	Male	No	No	Spontaneous	0.37773732
No	Winter	Male	Yes	No	Spontaneous	0.68903993
No	Autumn	Male	Yes	No	Spontaneous	-1.2186119
No	Winter	Male	Yes	No	Spontaneous	-0.2883476
No	Spring	Female	Yes	No	Spontaneous	0.12170344
No	Autumn	Male	No	No	Spontaneous	1.26076129
No	Autumn	Female	Yes	No	Spontaneous	-1.3109357
No	Spring	Female	No	No	Spontaneous	0.29712992
No	Summer	Male	No	No	Spontaneous	-0.4605425
No	Summer	Male	No	No	Spontaneous	0.90971779
No	Summer	Male	No	No	Spontaneous	-0.9356217
No	Autumn	Female	No	No	Spontaneous	-0.3641815
No	Summer	Male	No	No	Spontaneous	0.57870728
No	Winter	Male	No	No	Spontaneous	-0.919424
No	Autumn	Male	No	No	Spontaneous	0.55090797
No	Summer	Female	No	No	Spontaneous	0.67028386
No	Autumn	Male	No	No	Spontaneous	-0.1254004
No	Summer	Male	Yes	No	Spontaneous	-0.1841578
No	Summer	Male	No	No	Spontaneous	0.23648971
No	Summer	Female	No	No	Spontaneous	0.54452081
No	Summer	Male	No	TplAdmitted	Spontaneous	-0.9950954
No	Winter	Male	Yes	No	Spontaneous	-0.7101677
No	Winter	Male	No	No	Spontaneous	-0.0735884
No	Spring	Male	No	TplAdmitted	Spontaneous	-1.2140394
No	Spring	Female	No	No	Spontaneous	1.44362653
No	Winter	Male	No	No	Spontaneous	0.28260682
No	Summer	Female	No	No	Spontaneous	0.13873704
No	Autumn	Female	No	No	Spontaneous	0.27984173
No	Summer	Male	No	No	Spontaneous	0.68086029
No	Autumn	Female	No	No	Spontaneous	0.03842345
No	Spring	Male	No	No	Spontaneous	0.87996905
No	Winter	Male	No	No	Spontaneous	-0.8584385
No	Autumn	Male	Yes	No	Spontaneous	-0.8423343
No	Summer	Female	Yes	No	Spontaneous	-0.4072945

No	Winter	Male	No	No	Spontaneous	-0.2270888
No	Winter	Female	Yes	No	Spontaneous	0.96385718
No	Winter	Male	No	No	Spontaneous	0.8320635
No	Summer	Male	No	No	Spontaneous	-0.8935933
No	Autumn	Female	No	No	Spontaneous	2.17593898
No	Summer	Male	No	No	Spontaneous	1.45494454
No	Summer	Female	Yes	No	Spontaneous	1.05838813
No	Spring	Female	Yes	No	Spontaneous	2.01697414
No	Winter	Female	No	No	Spontaneous	-0.1799118
No	Spring	Female	No	No	Spontaneous	-0.1007428
No	Spring	Female	No	No	Spontaneous	-0.4806529
No	Spring	Male	No	No	Spontaneous	0.55611394
No	Autumn	Female	No	No	Spontaneous	-0.5206601
No	Winter	Male	No	No	Spontaneous	-0.3794475
No	Summer	Female	Yes	No	Spontaneous	-1.1990556
No	Autumn	Female	No	No	Spontaneous	0.25889859
No	Autumn	Female	No	No	Spontaneous	1.18043745
No	Winter	Female	No	No	Spontaneous	-0.568003
No	Spring	Male	Yes	No	Spontaneous	0.70653479
No	Summer	Female	No	No	Spontaneous	-0.2827484
No	Winter	Female	No	No	Spontaneous	1.06122918
No	Autumn	Male	No	No	Spontaneous	0.76071759
No	Spring	Male	No	No	Spontaneous	0.53749135
No	Spring	Male	Yes	No	Spontaneous	-0.9043468
No	Winter	Male	No	No	Spontaneous	1.20527055
No	Spring	Female	Yes	No	Spontaneous	0.79421468
No	Autumn	Male	Yes	No	Spontaneous	1.66858258
No	Autumn	Male	No	No	Spontaneous	0.13110344
No	Winter	Male	No	No	Spontaneous	0.03241679
Yes	Spring	Male	No	TplAdmitted	Spontaneous	0.16435773
No	Spring	Female	No	No	Spontaneous	-0.4825511
No	Summer	Female	No	No	Spontaneous	1.3299532
No	Winter	Female	No	No	Spontaneous	0.69905323
No	Summer	Male	No	No	Spontaneous	1.12120671
No	Spring	Male	No	No	Spontaneous	-1.1553059
Yes	Spring	Female	Yes	No	Spontaneous	-0.6571114
No	Spring	Female	No	No	Spontaneous	0.11159076
No	Summer	Male	No	No	Spontaneous	-1.9947569
No	Autumn	Female	No	No	Spontaneous	0.98123057
No	Summer	Female	No	No	Spontaneous	-1.3415435
No	Spring	Female	No	No	Spontaneous	-0.9314703
No	Spring	Female	No	No	Spontaneous	-0.3404384
No	Winter	Male	No	No	Spontaneous	-0.5474357
No	Summer	Female	Yes	No	Spontaneous	0.80878682
No	Winter	Male	No	No	Spontaneous	-0.0718173
No	Spring	Male	No	No	Spontaneous	-0.1967202
No	Autumn	Female	No	No	Spontaneous	1.16659116
No	Autumn	Male	No	No	Spontaneous	0.12680673

No	Spring	Female	Yes	No	Spontaneous	-2.4698212
No	Autumn	Male	No	No	Spontaneous	0.68479656
No	Autumn	Male	No	No	Spontaneous	-0.2625985
No	Summer	Male	No	No	Spontaneous	-0.2858683
No	Winter	Female	Yes	No	Spontaneous	-0.5160171
No	Spring	Male	No	No	Spontaneous	0.65091509
No	Summer	Female	No	TplAdmitted	Spontaneous	-0.9205567
No	Winter	Male	No	No	Spontaneous	-1.4367753
No	Winter	Male	No	No	Assisted	-1.1972966
No	Winter	Male	No	No	Spontaneous	-0.3126557
No	Spring	Female	No	No	Spontaneous	0.32499714
No	Autumn	Female	No	No	Spontaneous	0.25771035
No	Autumn	Male	No	No	Spontaneous	0.84285898
No	Spring	Male	No	No	Spontaneous	-0.7040595
No	Summer	Female	Yes	No	Spontaneous	-0.8992284
No	Summer	Female	No	No	Spontaneous	0.35917091
No	Autumn	Female	Yes	No	Spontaneous	0.53087621
No	Summer	Male	No	No	Spontaneous	0.76258519
No	Summer	Female	No	No	Spontaneous	-0.1153058
No	Autumn	Male	No	No	Spontaneous	1.81418407
No	Summer	Male	No	No	Assisted	0.55951964
No	Summer	Female	No	No	Spontaneous	0.24748798
No	Winter	Male	Yes	No	Spontaneous	0.14664072
No	Spring	Female	No	No	Spontaneous	0.25794504
No	Spring	Male	Yes	No	Spontaneous	-0.7771116
No	Winter	Male	No	No	Spontaneous	-1.642309
No	Summer	Female	No	No	Spontaneous	0.15193998
No	Autumn	Female	No	No	Spontaneous	0.54341047
No	Summer	Female	Yes	No	Assisted	-0.1443592
No	Winter	Female	No	No	Spontaneous	0.73436683
No	Summer	Female	No	No	Spontaneous	0.78300321
No	Autumn	Male	No	No	Spontaneous	-0.9080261
No	Winter	Male	Yes	No	Spontaneous	-1.6811301
No	Summer	Male	No	No	Spontaneous	-0.0149698
No	Spring	Male	No	No	Spontaneous	1.05223747
No	Autumn	Female	No	No	Spontaneous	1.11751557
No	Winter	Male	No	No	Spontaneous	-0.2819962
No	Summer	Female	No	No	Spontaneous	-0.9588089
Yes	Winter	Female	No	No	Spontaneous	-1.2325857
No	Autumn	Male	No	No	Spontaneous	1.49095388
No	Summer	Female	No	No	Spontaneous	0.2543426
No	Summer	Female	No	No	Spontaneous	0.5479886
No	Spring	Male	No	No	Spontaneous	1.5259303
No	Spring	Female	No	No	Spontaneous	-0.5064
No	Spring	Male	Yes	No	Spontaneous	-0.3253593
No	Autumn	Male	Yes	No	Spontaneous	0.92473393
Yes	Winter	Female	No	No	Spontaneous	0.38138773
No	Spring	Female	No	No	Spontaneous	-0.5178157

No	Winter	Male	No	No	Spontaneous	-0.8031992
No	Summer	Female	No	No	Spontaneous	1.68338098
No	Summer	Male	No	No	Spontaneous	0.18002059
No	Winter	Male	Yes	No	Spontaneous	0.86984016
No	Autumn	Male	No	No	Spontaneous	0.42215962
No	Autumn	Female	No	No	Spontaneous	-0.9597765
No	Summer	Male	No	No	Spontaneous	0.32458043
No	Winter	Male	No	No	Spontaneous	0.69375712
No	Winter	Male	No	No	Spontaneous	-0.7928393
No	Winter	Male	Yes	No	Spontaneous	-0.6872702
No	Spring	Male	No	TplAdmitted	Spontaneous	-1.0420213
No	Spring	Male	No	No	Spontaneous	-0.7929182
No	Summer	Female	No	No	Spontaneous	0.91316983
No	Spring	Male	No	No	Spontaneous	0.92883984
No	Spring	Female	No	No	Spontaneous	-0.4819601
No	Summer	Female	No	No	Spontaneous	0.63074664
Yes	Summer	Male	Yes	TplAdmitted	Spontaneous	0.72688014
No	Autumn	Female	No	No	Spontaneous	-1.2978318
No	Autumn	Male	No	No	Spontaneous	0.65628281
No	Summer	Male	Yes	No	Spontaneous	-0.1978241
No	Summer	Male	No	No	Spontaneous	0.04192064
No	Summer	Male	No	No	Spontaneous	-0.1172167
Yes	Spring	Male	Yes	No	Spontaneous	-1.4191269
No	Spring	Female	No	TplAdmitted	Spontaneous	1.74177826
No	Spring	Female	No	No	Spontaneous	0.08964694
No	Autumn	Male	No	No	Spontaneous	-0.732928
No	Autumn	Male	Yes	No	Spontaneous	0.35860302
No	Autumn	Female	No	No	Spontaneous	0.67839791
No	Winter	Female	No	No	Spontaneous	0.01499937
No	Winter	Male	No	No	Spontaneous	-0.4988426
No	Summer	Female	No	No	Spontaneous	-0.8959149
No	Autumn	Male	No	No	Spontaneous	0.14859514
No	Summer	Male	No	No	Spontaneous	0.16796372
No	Summer	Female	No	No	Spontaneous	-0.8788419
No	Spring	Female	Yes	No	Spontaneous	0.04001401
No	Summer	Male	No	No	Spontaneous	-0.0588014
No	Spring	Female	No	No	Spontaneous	-1.3781692
No	Spring	Male	No	No	Spontaneous	0.92104636
No	Spring	Female	Yes	No	Spontaneous	-0.8924559
No	Spring	Male	Yes	No	Spontaneous	1.50091541
No	Autumn	Female	No	No	Spontaneous	0.37821433
Yes	Autumn	Male	No	No	Spontaneous	1.48482615
No	Summer	Female	No	No	Spontaneous	0.38113049
No	Summer	Male	No	No	Spontaneous	-0.5520139
No	Winter	Male	Yes	No	Spontaneous	-0.6748641
No	Summer	Female	No	No	Assisted	0.08832802
No	Autumn	Female	No	No	Spontaneous	0.26655978
No	Winter	Male	No	No	Spontaneous	-0.2577839

No	Winter	Male	Yes	No	Spontaneous	0.34893609
No	Spring	Female	No	No	Spontaneous	-1.769524
No	Summer	Female	No	No	Spontaneous	-0.312894
No	Spring	Female	No	No	Spontaneous	0.27994343
No	Spring	Male	No	No	Spontaneous	-1.3446082
No	Spring	Female	Yes	No	Spontaneous	0.93949226
No	Spring	Male	No	No	Spontaneous	0.47055869
No	Summer	Male	No	No	Spontaneous	-1.2232175
No	Summer	Female	No	No	Spontaneous	0.4265958
No	Spring	Male	Yes	No	Spontaneous	0.05941665
No	Autumn	Female	No	No	Spontaneous	0.3474759
No	Spring	Male	No	No	Spontaneous	-0.5163074
No	Winter	Male	No	No	Spontaneous	0.68622139
No	Summer	Female	No	No	Spontaneous	0.44566814
No	Spring	Male	No	No	Spontaneous	1.36249052
No	Winter	Female	No	No	Spontaneous	-0.8274555
No	Spring	Male	No	No	Spontaneous	2.15865899
No	Winter	Male	No	No	Spontaneous	1.45011524
No	Autumn	Male	No	No	Spontaneous	1.17458422
Yes	Summer	Male	No	No	Spontaneous	-0.0925657
No	Spring	Male	No	No	Spontaneous	0.93139551
No	Spring	Male	No	No	Assisted	-0.182184
No	Spring	Male	No	No	Spontaneous	1.99887674
No	Summer	Female	Yes	No	Spontaneous	0.09405384
No	Spring	Male	No	No	Spontaneous	-0.24506
No	Autumn	Female	No	No	Spontaneous	-0.7607366
No	Winter	Male	No	No	Spontaneous	0.05043667
No	Spring	Male	No	No	Spontaneous	-0.089789
No	Spring	Male	No	No	Spontaneous	2.03358832
No	Spring	Male	Yes	No	Spontaneous	-1.4970431
No	Autumn	Male	No	No	Spontaneous	1.52708555
No	Winter	Male	No	No	Spontaneous	0.21636298
No	Winter	Female	No	No	Spontaneous	1.24827861
No	Autumn	Female	No	No	Spontaneous	0.33812968
No	Spring	Male	No	No	Assisted	-1.5602091
Yes	Autumn	Female	No	TplAdmitted	Spontaneous	1.85601091
No	Autumn	Female	No	No	Spontaneous	-0.53171
No	Spring	Female	Yes	No	Spontaneous	-0.1666567
No	Autumn	Male	No	No	Spontaneous	0.0567381
No	Winter	Female	Yes	TplAdmitted	Spontaneous	0.82494366
No	Winter	Female	Yes	TplAdmitted	Spontaneous	-0.3814223
No	Winter	Male	No	No	Spontaneous	-0.9868315
No	Winter	Male	No	No	Spontaneous	-0.6249014
No	Winter	Female	No	No	Spontaneous	-0.0532489
No	Winter	Male	Yes	No	Spontaneous	-0.9187406
No	Autumn	Male	No	No	Spontaneous	-0.1417814
No	Summer	Male	No	No	Spontaneous	-0.3925878
No	Autumn	Female	Yes	No	Spontaneous	1.38351181

No	Summer	Female	Yes	No	Spontaneous	0.1959967
No	Summer	Female	No	No	Spontaneous	0.57381318
No	Autumn	Female	Yes	No	Spontaneous	0.63053658
No	Spring	Male	Yes	No	Spontaneous	-1.0087986
No	Winter	Male	No	No	Spontaneous	-2.4342686
No	Summer	Male	No	No	Spontaneous	1.94418494
No	Winter	Male	No	No	Spontaneous	0.70002113
No	Spring	Female	No	No	Spontaneous	-0.5817755
No	Winter	Female	No	No	Spontaneous	0.84744308
No	Summer	Male	No	No	Spontaneous	-0.4150267
No	Autumn	Male	No	No	Spontaneous	-1.2087249
No	Winter	Male	No	No	Assisted	-0.6417275
No	Spring	Female	No	No	Spontaneous	-0.1127413
No	Winter	Female	Yes	No	Spontaneous	0.90771851
No	Autumn	Male	No	No	Spontaneous	-0.3067693
No	Spring	Male	No	No	Spontaneous	0.65091509
No	Spring	Female	No	No	Assisted	-1.9581544
No	Summer	Female	No	No	Assisted	0.74176653
No	Autumn	Male	No	No	Spontaneous	0.02934111
No	Winter	Female	No	No	Spontaneous	0.27619564
No	Spring	Female	Yes	No	Spontaneous	-0.9816337
No	Winter	Female	No	No	Spontaneous	-0.2708567
No	Summer	Female	Yes	No	Spontaneous	0.64152907
No	Summer	Female	Yes	No	Spontaneous	-1.1263802
No	Summer	Female	No	No	Spontaneous	-0.0857934
No	Summer	Female	No	No	Spontaneous	0.01372178
No	Winter	Female	Yes	TplAdmitted	Spontaneous	-1.4289838
No	Winter	Female	Yes	No	Spontaneous	0.72352397
No	Winter	Male	Yes	No	Spontaneous	1.49956309
No	Autumn	Female	No	No	Spontaneous	-0.4384286
No	Autumn	Female	No	No	Spontaneous	-0.8994492
No	Spring	Male	No	No	Spontaneous	0.34696561
No	Autumn	Female	Yes	No	Spontaneous	-1.6687425
No	Autumn	Male	Yes	No	Spontaneous	-0.4119321
No	Autumn	Male	No	No	Spontaneous	0.46354707
No	Winter	Female	Yes	No	Spontaneous	-1.0913942
No	Winter	Female	No	No	Spontaneous	0.11327405
No	Summer	Male	No	No	Spontaneous	0.2100051
No	Winter	Male	No	No	Assisted	-0.2147588
Yes	Autumn	Male	No	TplAdmitted	Spontaneous	-0.5363079
No	Spring	Male	No	No	Spontaneous	0.74999073
No	Summer	Male	No	No	Spontaneous	0.23483785
No	Winter	Female	No	No	Spontaneous	-1.3845662
No	Summer	Male	Yes	No	Spontaneous	1.13883321
No	Winter	Female	No	No	Spontaneous	-0.130739
No	Autumn	Male	No	No	Spontaneous	0.961959
No	Autumn	Female	No	No	Spontaneous	-0.7385608
No	Spring	Male	No	No	Spontaneous	1.6040391

No	Summer	Male	No	No	Spontaneous	-0.8443527
No	Spring	Male	No	No	Spontaneous	-0.1441078
No	Summer	Female	No	No	Spontaneous	0.50853314
No	Spring	Female	Yes	No	Spontaneous	0.09856987
No	Spring	Female	No	No	Spontaneous	0.20957648
No	Spring	Male	No	No	Spontaneous	0.87522055
No	Winter	Female	No	No	Spontaneous	0.52013136
No	Spring	Male	Yes	No	Assisted	0.31073431
No	Autumn	Female	No	No	Spontaneous	1.9359343
No	Winter	Male	No	No	Spontaneous	-1.1653751
No	Summer	Female	Yes	No	Spontaneous	0.95997683
No	Spring	Male	No	No	Spontaneous	-1.6105556
No	Summer	Male	No	No	Spontaneous	-1.0334434
No	Winter	Male	No	No	Spontaneous	1.10977352
No	Winter	Male	No	No	Assisted	-0.5434606
No	Winter	Female	No	No	Spontaneous	-0.476836
No	Winter	Female	No	No	Spontaneous	-0.0813579
No	Autumn	Female	Yes	No	Assisted	-0.6619079
No	Autumn	Female	No	No	Spontaneous	-0.7262446
No	Winter	Female	No	No	Spontaneous	1.55895254
No	Summer	Male	Yes	No	Spontaneous	-1.0826875
No	Winter	Female	No	No	Spontaneous	-1.1521008
No	Summer	Male	No	No	Spontaneous	-1.4400499
No	Spring	Male	No	No	Spontaneous	0.99692849
No	Summer	Male	Yes	TplAdmitted	Spontaneous	0.01301759
No	Spring	Female	Yes	No	Spontaneous	0.2811019
No	Autumn	Female	No	No	Spontaneous	-1.0669451
No	Summer	Male	No	No	Spontaneous	-0.3947401
No	Spring	Male	No	No	Spontaneous	0.2855552
No	Summer	Female	No	No	Spontaneous	-1.2331274
No	Spring	Male	No	No	Spontaneous	-0.0931955
No	Autumn	Female	No	No	Spontaneous	-0.7672046
No	Autumn	Male	No	No	Spontaneous	0.42295989
No	Winter	Male	No	No	Spontaneous	0.04819972
No	Spring	Male	Yes	No	Spontaneous	-0.652069
No	Summer	Male	No	No	Spontaneous	-2.2321151
No	Spring	Male	Yes	No	Spontaneous	0.73942138
No	Winter	Female	No	No	Spontaneous	0.56345142
No	Summer	Female	No	No	Spontaneous	0.4255274
No	Summer	Male	Yes	No	Spontaneous	0.99099377
No	Winter	Female	No	No	Spontaneous	-1.0549547
No	Summer	Male	No	No	Spontaneous	-0.0981241
No	Summer	Female	No	No	Spontaneous	-0.508276
No	Autumn	Female	Yes	No	Spontaneous	0.45424265
No	Winter	Female	No	No	Spontaneous	1.14348459
No	Summer	Female	No	No	Spontaneous	-0.647041
No	Winter	Male	No	No	Spontaneous	-0.7657635
No	Winter	Male	No	No	Spontaneous	0.17513471

No	Autumn	Male	No	No	Spontaneous	-1.2520205
No	Spring	Male	No	No	Spontaneous	1.25420343
No	Autumn	Female	No	No	Spontaneous	-0.3076086
No	Winter	Male	No	No	Spontaneous	0.06567364
No	Winter	Male	No	No	Spontaneous	-0.1438876
Yes	Autumn	Male	No	No	Spontaneous	1.42819198
No	Winter	Male	No	No	Spontaneous	-0.5701072
No	Spring	Male	No	No	Spontaneous	-0.2700897
No	Autumn	Female	Yes	No	Spontaneous	-0.9065736
No	Winter	Male	Yes	No	Spontaneous	-1.1365351
No	Autumn	Male	No	No	Spontaneous	0.28592211
No	Winter	Male	Yes	TplAdmitted	Spontaneous	2.24094212
No	Summer	Male	No	No	Spontaneous	-1.1471233
No	Summer	Male	Yes	No	Assisted	-1.1712976
No	Autumn	Female	No	No	Spontaneous	-0.3305537
No	Winter	Male	No	No	Spontaneous	0.54346787
No	Winter	Male	No	No	Assisted	-1.0242128
Yes	Autumn	Male	No	No	Assisted	0.22609571
No	Autumn	Male	No	No	Spontaneous	-0.2754582
No	Autumn	Male	No	No	Assisted	-0.0645776
No	Spring	Male	No	No	Spontaneous	-0.3994184
No	Spring	Male	Yes	No	Spontaneous	-0.7766856
No	Spring	Male	No	No	Spontaneous	1.35036415
No	Summer	Female	No	No	Spontaneous	-0.3904134
No	Summer	Female	No	No	Spontaneous	0.37226666
No	Autumn	Male	No	No	Spontaneous	-0.1705694
No	Summer	Female	No	No	Spontaneous	-0.7672046
No	Summer	Male	No	No	Spontaneous	0.07072131
No	Winter	Female	Yes	No	Spontaneous	1.17783885
No	Autumn	Male	No	No	Assisted	0.00835356
No	Spring	Female	No	No	Spontaneous	1.51000857
No	Spring	Male	No	No	Spontaneous	-0.0705069
No	Spring	Female	No	No	Spontaneous	1.31998017
No	Summer	Male	No	No	Spontaneous	-0.6448833
No	Spring	Female	No	No	Spontaneous	-0.5160171
Yes	Summer	Male	No	No	Spontaneous	0.48801146
No	Summer	Male	No	No	Spontaneous	-0.7307036
No	Autumn	Male	No	No	Spontaneous	1.29942368
No	Summer	Female	No	No	Spontaneous	-1.3335023
No	Spring	Female	No	No	Spontaneous	0.9276312
No	Autumn	Female	Yes	No	Spontaneous	0.08988599
No	Autumn	Male	No	No	Spontaneous	0.13728871
No	Spring	Female	No	No	Spontaneous	0.47672082
No	Winter	Male	Yes	No	Spontaneous	0.87615954
No	Autumn	Male	No	No	Spontaneous	-0.5748769
Yes	Winter	Male	No	TplAdmitted	Spontaneous	0.35829626
No	Autumn	Female	No	No	Spontaneous	0.88122262
No	Autumn	Male	No	No	Spontaneous	-1.5514809

No	Winter	Male	Yes	No	Spontaneous	-0.5372051
No	Winter	Female	No	No	Spontaneous	0.01123457
No	Autumn	Female	No	No	Spontaneous	1.38142909
No	Winter	Male	No	No	Spontaneous	1.26388828
No	Autumn	Male	No	No	Spontaneous	-0.85686
No	Autumn	Male	No	No	Spontaneous	1.68077037
No	Autumn	Male	No	No	Spontaneous	-1.3363853
No	Winter	Female	No	No	Spontaneous	0.41647366
No	Autumn	Male	No	No	Spontaneous	0.30133854
No	Spring	Female	Yes	No	Spontaneous	0.66097835
No	Spring	Female	No	No	Assisted	1.31746338
No	Autumn	Female	Yes	No	Spontaneous	0.52699581
No	Summer	Male	Yes	No	Spontaneous	-0.6650769
No	Winter	Female	No	No	Spontaneous	0.80171025
No	Spring	Male	No	No	Spontaneous	-0.8854922
No	Autumn	Female	No	No	Spontaneous	-0.1898638
No	Autumn	Male	No	No	Spontaneous	-1.4367753
No	Winter	Female	No	No	Spontaneous	0.58601974
No	Summer	Female	No	No	Spontaneous	1.38989322
No	Winter	Male	No	No	Spontaneous	0.55868268
No	Summer	Female	No	No	Spontaneous	1.3680052
No	Summer	Male	No	No	Spontaneous	0.09140839
No	Winter	Female	No	No	Spontaneous	-0.6332113
No	Autumn	Male	No	No	Spontaneous	0.51560254
No	Spring	Male	No	No	Spontaneous	-0.6235061
No	Autumn	Male	Yes	No	Spontaneous	0.83233269
No	Spring	Female	Yes	TplAdmitted	Spontaneous	0.33311508
No	Spring	Male	No	No	Spontaneous	-0.2963204
No	Autumn	Female	No	No	Spontaneous	0.92603127
No	Winter	Male	No	No	Spontaneous	-0.367398
No	Autumn	Female	No	No	Spontaneous	-0.1417922
No	Summer	Female	No	No	Spontaneous	0.30297295
No	Spring	Female	Yes	No	Spontaneous	0.06942161
No	Autumn	Female	No	No	Spontaneous	0.9432535
No	Summer	Male	No	No	Spontaneous	-1.2808872
No	Autumn	Female	No	No	Spontaneous	-1.6344603
No	Spring	Female	No	No	Spontaneous	-0.6216895
No	Spring	Female	Yes	No	Spontaneous	0.56409624
No	Summer	Male	No	No	Spontaneous	1.11073623
No	Autumn	Male	No	No	Spontaneous	1.41261876
No	Summer	Female	No	No	Spontaneous	0.09038925
No	Winter	Male	Yes	No	Spontaneous	-0.7504666
No	Summer	Female	No	No	Spontaneous	-0.6567552
No	Summer	Female	No	No	Spontaneous	0.3474759
No	Autumn	Female	No	No	Assisted	0.05416407
No	Winter	Male	No	No	Spontaneous	0.28418431
No	Winter	Male	No	No	Spontaneous	-0.4099004
No	Winter	Male	No	No	Spontaneous	-0.1881948

No	Summer	Female	No	No	Spontaneous	1.02196168
No	Autumn	Male	No	No	Spontaneous	-1.976602
No	Spring	Female	No	No	Spontaneous	-0.0532489
No	Winter	Male	No	No	Spontaneous	-0.4708906
No	Winter	Female	No	No	Spontaneous	0.38991239
No	Summer	Male	No	No	Spontaneous	0.35482483
No	Summer	Female	No	No	Spontaneous	-0.2317312
No	Spring	Female	No	No	Spontaneous	-0.2654341
No	Summer	Male	No	No	Spontaneous	0.25245093
No	Autumn	Male	No	No	Spontaneous	-1.090457
No	Autumn	Female	No	No	Spontaneous	-0.8610959
No	Winter	Male	Yes	No	Spontaneous	1.10775031
No	Summer	Female	No	No	Spontaneous	-0.5799354
No	Summer	Female	No	No	Spontaneous	0.49983496
Yes	Autumn	Female	No	No	Spontaneous	-0.4645391
No	Autumn	Male	No	No	Spontaneous	1.11869451
No	Summer	Female	No	No	Spontaneous	-0.2634908
No	Winter	Female	No	No	Spontaneous	-1.5822087
No	Spring	Male	No	No	Spontaneous	-0.2433212
No	Autumn	Male	No	No	Spontaneous	0.27916036
No	Spring	Male	No	No	Spontaneous	0.75591486
No	Autumn	Female	No	No	Spontaneous	0.63580841
No	Winter	Male	No	No	Spontaneous	1.56927321
Yes	Winter	Female	No	No	Spontaneous	-0.0072118
No	Autumn	Male	No	No	Spontaneous	0.11079154
No	Autumn	Male	No	No	Spontaneous	0.63442457
No	Spring	Male	No	No	Spontaneous	0.49784004
No	Spring	Female	Yes	No	Spontaneous	-0.2436052
No	Summer	Female	No	No	Spontaneous	1.36222775
No	Autumn	Male	No	No	Spontaneous	0.05149426
No	Summer	Male	No	No	Spontaneous	0.10995629
Yes	Autumn	Male	Yes	TplAdmitted	Spontaneous	1.36909045
No	Summer	Male	No	No	Spontaneous	-0.4659123
No	Autumn	Female	No	No	Spontaneous	-0.1202199
No	Winter	Male	No	No	Spontaneous	-0.4889613
No	Autumn	Male	No	TplAdmitted	Spontaneous	-1.1473103
No	Winter	Male	No	No	Spontaneous	-0.052343
No	Winter	Male	No	No	Spontaneous	0.5600532
No	Autumn	Female	No	No	Spontaneous	0.92948577
No	Spring	Male	No	No	Spontaneous	0.87958081
No	Spring	Male	No	No	Spontaneous	0.54653389
No	Winter	Female	No	No	Spontaneous	0.95247381
No	Spring	Male	No	No	Spontaneous	1.10717014
No	Autumn	Male	No	No	Spontaneous	-0.3897589
No	Summer	Female	Yes	No	Spontaneous	-0.3616098
No	Winter	Male	No	No	Spontaneous	1.44766141
No	Summer	Female	No	No	Spontaneous	-0.5364925
No	Autumn	Male	No	No	Spontaneous	-0.0609823

No	Summer	Female	No	No	Spontaneous	1.2280636
No	Winter	Female	Yes	No	Spontaneous	-1.8124661
No	Summer	Female	No	No	Spontaneous	-0.6023605
No	Summer	Female	No	No	Spontaneous	-0.0340326
No	Summer	Male	Yes	No	Spontaneous	0.7662098
No	Summer	Male	No	No	Spontaneous	-0.7669365
No	Autumn	Male	No	No	Spontaneous	-2.2075451
Yes	Spring	Female	No	TplAdmitted	Spontaneous	0.13581302
No	Winter	Female	No	TplAdmitted	Spontaneous	-1.655146
No	Spring	Male	No	No	Spontaneous	-0.6974981
No	Autumn	Female	No	No	Spontaneous	0.49912358
No	Spring	Male	No	No	Spontaneous	0.15182817
No	Winter	Female	No	No	Spontaneous	-0.7747919
No	Autumn	Male	No	No	Spontaneous	-0.3502554
No	Spring	Male	No	No	Spontaneous	0.71398851
No	Autumn	Male	Yes	No	Spontaneous	-0.41562
No	Autumn	Male	Yes	No	Spontaneous	-0.8865304
No	Autumn	Male	No	No	Spontaneous	-2.2810075
No	Winter	Female	Yes	No	Spontaneous	-0.9720481
No	Winter	Female	No	No	Spontaneous	0.02681239
No	Summer	Female	No	No	Spontaneous	-0.6936074
No	Autumn	Male	No	No	Assisted	1.03292083
No	Summer	Female	No	No	Spontaneous	-0.2959002
No	Spring	Male	No	No	Spontaneous	-0.4923919
No	Winter	Female	No	No	Spontaneous	1.55333431
No	Spring	Male	Yes	No	Spontaneous	-0.5768383
No	Summer	Male	No	No	Spontaneous	-0.0769136
No	Summer	Male	Yes	No	Spontaneous	0.58612704
No	Winter	Female	No	No	Spontaneous	-1.2570236
No	Winter	Male	No	No	Spontaneous	0.08580384
No	Winter	Male	No	No	Spontaneous	1.99640343
No	Autumn	Male	No	No	Spontaneous	-1.2186119
No	Spring	Female	No	No	Spontaneous	0.32510474
No	Summer	Female	No	No	Spontaneous	-1.1860439
Yes	Summer	Female	No	No	Spontaneous	1.45457954
No	Autumn	Male	No	No	Spontaneous	2.03348328
No	Spring	Male	No	No	Spontaneous	0.95175338
No	Summer	Male	No	No	Spontaneous	-0.3351435
No	Winter	Male	No	No	Spontaneous	-0.0238384
No	Autumn	Female	No	No	Spontaneous	-0.6121653
No	Summer	Male	No	No	Spontaneous	1.43219934
No	Winter	Male	No	No	Spontaneous	0.78245349
No	Spring	Male	No	No	Spontaneous	0.26968654
No	Winter	Female	No	No	Spontaneous	-0.392891
No	Autumn	Male	No	No	Spontaneous	0.23064469
No	Spring	Female	Yes	No	Spontaneous	-1.9079512
No	Winter	Male	No	No	Spontaneous	-1.0117715
No	Autumn	Male	Yes	No	Spontaneous	-0.3715981

No	Summer	Female	No	No	Spontaneous	0.00167702
No	Autumn	Male	No	No	Spontaneous	-0.7336704
No	Autumn	Male	No	No	Spontaneous	0.97678828
No	Autumn	Male	Yes	No	Spontaneous	1.30308313
No	Summer	Female	No	No	Spontaneous	-0.8219503
No	Autumn	Female	No	No	Spontaneous	0.8525495
No	Autumn	Male	No	No	Spontaneous	0.24803806
No	Winter	Female	Yes	No	Spontaneous	1.56760593
No	Summer	Male	No	No	Spontaneous	0.64761399
No	Autumn	Female	No	No	Spontaneous	1.80847496
No	Summer	Female	No	No	Assisted	0.54556771
No	Winter	Male	No	No	Spontaneous	-0.8196504
No	Winter	Female	No	No	Spontaneous	-0.9183646
No	Spring	Female	No	No	Spontaneous	-1.8497471
No	Summer	Male	No	No	Spontaneous	-1.7870935
No	Autumn	Male	No	No	Spontaneous	-0.6120102
No	Winter	Male	No	No	Spontaneous	0.82918082
No	Autumn	Female	No	No	Spontaneous	0.99087051
No	Summer	Male	No	No	Assisted	-0.1419123
Yes	Autumn	Male	Yes	No	Spontaneous	0.12387646
No	Autumn	Male	Yes	No	Spontaneous	-0.550236
No	Spring	Female	Yes	No	Spontaneous	-2.4719376
No	Summer	Female	No	No	Spontaneous	-0.6033951
Yes	Spring	Male	No	No	Spontaneous	1.42724571
No	Summer	Male	No	No	Spontaneous	-0.3336288
No	Winter	Female	No	No	Spontaneous	1.2907218
No	Autumn	Female	No	No	Spontaneous	0.2754044
No	Winter	Female	No	No	Spontaneous	-0.1459443
No	Winter	Male	No	No	Spontaneous	-0.8728742
No	Autumn	Male	No	No	Spontaneous	0.84157512
No	Autumn	Male	No	No	Spontaneous	-0.0981241
No	Autumn	Male	No	No	Spontaneous	0.8200508
No	Winter	Female	No	No	Spontaneous	0.1542959
No	Spring	Male	Yes	No	Spontaneous	-0.0800633
No	Autumn	Male	No	No	Spontaneous	0.02608119
No	Winter	Male	No	No	Spontaneous	-1.3107832
No	Summer	Female	No	No	Spontaneous	0.59359236
No	Winter	Male	No	No	Spontaneous	0.1842964
No	Autumn	Female	No	No	Spontaneous	0.63674998
No	Autumn	Female	Yes	No	Spontaneous	-0.9908523
No	Spring	Female	Yes	No	Spontaneous	1.78447506
No	Autumn	Male	No	No	Spontaneous	1.01820303
No	Spring	Female	No	No	Spontaneous	0.5676196
No	Summer	Female	No	No	Spontaneous	-0.9786251
No	Winter	Female	No	No	Spontaneous	-1.404234
No	Autumn	Female	No	TplAdmitted	Spontaneous	-0.8816586
No	Winter	Female	No	No	Spontaneous	-0.2211142
No	Summer	Male	No	No	Spontaneous	0.84350896

No	Autumn	Male	No	No	Spontaneous	0.34788384
No	Winter	Female	No	No	Spontaneous	0.11135247
No	Summer	Male	No	No	Spontaneous	0.71608678
No	Winter	Female	No	No	Spontaneous	-1.3715459
No	Summer	Male	No	No	Spontaneous	0.19965386
No	Autumn	Male	No	No	Spontaneous	2.01295729
No	Autumn	Male	No	No	Spontaneous	1.73077603
No	Winter	Female	No	TplAdmitted	Spontaneous	-0.0979605
No	Summer	Female	No	No	Spontaneous	-0.9502774
No	Summer	Female	No	No	Spontaneous	0.51080367
No	Winter	Female	No	No	Spontaneous	2.36436773
No	Autumn	Female	No	No	Spontaneous	-0.0080416
No	Spring	Female	No	No	Spontaneous	2.039991
No	Autumn	Male	No	No	Assisted	-0.9903237
No	Autumn	Female	Yes	No	Spontaneous	-1.2906263
No	Spring	Male	No	No	Spontaneous	1.23470454
No	Summer	Male	No	No	Spontaneous	1.13002425
No	Winter	Female	No	No	Spontaneous	0.2467721
No	Summer	Male	No	No	Spontaneous	0.86894287
No	Summer	Male	No	No	Spontaneous	-0.9680874
No	Spring	Female	No	No	Spontaneous	1.05502734
No	Autumn	Male	No	No	Spontaneous	1.82904536
No	Summer	Male	No	No	Spontaneous	1.48918398
No	Summer	Male	Yes	No	Spontaneous	0.32149328
No	Spring	Female	No	No	Spontaneous	-1.0640362
No	Autumn	Male	No	No	Spontaneous	0.07518354
No	Summer	Male	No	No	Spontaneous	-0.7640118
No	Autumn	Male	No	No	Spontaneous	0.05758039
Yes	Summer	Female	No	No	Spontaneous	-1.1614294
No	Winter	Female	Yes	No	Spontaneous	-1.1661966
No	Autumn	Female	No	No	Spontaneous	-0.0599076
No	Summer	Female	No	No	Spontaneous	1.13519745
No	Winter	Male	Yes	No	Spontaneous	1.98280343
No	Winter	Male	No	No	Spontaneous	-0.6762637
No	Spring	Male	No	No	Spontaneous	-0.638584
No	Autumn	Male	No	No	Spontaneous	1.20150146
No	Summer	Male	Yes	No	Spontaneous	-0.585543
No	Winter	Female	No	No	Spontaneous	-0.5631836
Yes	Winter	Male	No	TplAdmitted	Spontaneous	0.57389228
No	Summer	Male	No	No	Spontaneous	0.02866172
No	Autumn	Male	No	No	Spontaneous	-1.0026941
No	Spring	Female	No	No	Spontaneous	2.10923585
Yes	Winter	Female	No	No	Spontaneous	-0.9916133
No	Spring	Male	No	No	Spontaneous	1.06410759
No	Autumn	Male	No	No	Spontaneous	-0.4320011
No	Winter	Male	Yes	No	Spontaneous	0.98355606
No	Spring	Female	No	No	Spontaneous	-0.1364816
No	Summer	Female	No	No	Spontaneous	-0.3658501

No	Summer	Female	No	No	Spontaneous	0.40727549
No	Spring	Female	No	No	Assisted	-0.9779128
No	Winter	Male	No	No	Spontaneous	0.60421456
No	Spring	Female	No	No	Spontaneous	-0.429526
No	Autumn	Male	No	No	Spontaneous	-0.6017098
No	Winter	Female	No	No	Spontaneous	0.25780255
No	Spring	Male	Yes	No	Spontaneous	-2.2043744
No	Summer	Female	No	No	Spontaneous	0.87111778
No	Winter	Female	No	No	Spontaneous	0.53175464
No	Summer	Female	No	No	Spontaneous	0.06958658
No	Winter	Male	Yes	No	Spontaneous	0.43276089
No	Spring	Male	Yes	No	Spontaneous	-0.4350266
No	Winter	Female	No	No	Spontaneous	1.35894841
No	Autumn	Male	Yes	No	Spontaneous	-0.2037677
No	Summer	Female	No	No	Spontaneous	0.58459756
No	Spring	Male	Yes	No	Spontaneous	0.31156203
No	Winter	Male	No	No	Spontaneous	1.40138893
No	Winter	Male	No	No	Spontaneous	0.12891958
No	Summer	Male	No	No	Spontaneous	-0.8173323
No	Winter	Male	No	No	Spontaneous	-0.5917582
No	Winter	Female	No	No	Spontaneous	0.87428351
No	Winter	Male	No	No	Spontaneous	-0.6055962
No	Summer	Male	No	No	Spontaneous	-0.7956346
No	Winter	Male	No	No	Spontaneous	-1.3778731
No	Autumn	Female	Yes	No	Spontaneous	-0.700345
No	Summer	Male	No	No	Spontaneous	-1.3019808
No	Summer	Male	No	No	Spontaneous	0.99894994
No	Autumn	Female	No	No	Spontaneous	-2.4483623
No	Spring	Male	No	No	Spontaneous	2.11152537
No	Spring	Male	No	No	Spontaneous	-0.464738
No	Spring	Male	No	No	Spontaneous	0.83569922
No	Autumn	Female	No	No	Spontaneous	-1.8942547
No	Autumn	Male	No	No	Spontaneous	-0.5665225
No	Autumn	Female	No	No	Assisted	-0.4596861
No	Spring	Male	No	No	Spontaneous	-3.0803368
No	Autumn	Female	No	No	Spontaneous	0.01912385
No	Summer	Female	No	No	Spontaneous	0.61932806
No	Autumn	Male	No	No	Spontaneous	0.35381687
No	Autumn	Female	No	No	Spontaneous	1.11530107
No	Summer	Male	Yes	TplAdmitted	Spontaneous	1.67276142
No	Spring	Female	Yes	No	Spontaneous	0.76900552
No	Winter	Female	No	No	Spontaneous	-0.199365
No	Autumn	Male	No	No	Spontaneous	-0.364398
No	Winter	Male	No	No	Spontaneous	0.27227046
No	Autumn	Female	No	No	Spontaneous	-0.0445138
No	Autumn	Male	No	No	Spontaneous	-0.6427421
No	Spring	Male	No	No	Spontaneous	1.14341084
No	Spring	Female	No	No	Spontaneous	1.39808703

No	Autumn	Male	No	No	Spontaneous	0.35567394
No	Winter	Female	Yes	No	Spontaneous	0.73538649
No	Winter	Male	No	No	Assisted	1.00529132
No	Summer	Female	Yes	No	Spontaneous	0.07557741
No	Winter	Female	No	No	Spontaneous	0.68471588
No	Winter	Female	No	TplAdmitted	Spontaneous	0.18821639
No	Spring	Male	No	No	Spontaneous	0.93925304
No	Autumn	Male	No	No	Spontaneous	0.63714752
No	Spring	Male	No	No	Spontaneous	0.10449105
No	Autumn	Male	No	No	Spontaneous	0.72290655
No	Summer	Male	No	No	Spontaneous	0.8595129
No	Winter	Female	Yes	No	Spontaneous	0.40982236
No	Spring	Female	No	No	Spontaneous	1.99672486
No	Spring	Female	Yes	No	Spontaneous	-1.4753816
No	Winter	Male	No	No	Spontaneous	0.041943
No	Summer	Female	No	No	Spontaneous	0.52145957
No	Spring	Female	No	No	Spontaneous	1.31009928
No	Spring	Female	Yes	No	Spontaneous	0.59932974
No	Winter	Female	No	No	Spontaneous	-1.2001252
No	Spring	Female	No	No	Spontaneous	0.23560088
No	Autumn	Male	No	No	Spontaneous	1.22533158
No	Winter	Male	No	No	Spontaneous	0.33904504
No	Autumn	Female	No	No	Spontaneous	-1.1761083
No	Winter	Female	Yes	No	Spontaneous	-1.8682126
No	Winter	Male	No	No	Spontaneous	0.23780291
No	Winter	Male	No	No	Spontaneous	-0.8482165
No	Spring	Male	No	No	Spontaneous	1.6498164
No	Winter	Male	No	No	Spontaneous	0.24017851
No	Winter	Male	No	No	Spontaneous	0.07276293
No	Spring	Female	No	No	Spontaneous	-0.6469769
No	Autumn	Male	No	No	Spontaneous	0.80511354
No	Winter	Male	No	No	Spontaneous	0.51390921
No	Autumn	Female	No	No	Spontaneous	2.13225151
No	Winter	Male	No	No	Spontaneous	-0.2883476
No	Autumn	Male	No	No	Spontaneous	-1.5675841
No	Spring	Male	No	No	Spontaneous	0.74972473
No	Spring	Male	Yes	No	Spontaneous	0.05149426
No	Spring	Male	No	No	Spontaneous	1.15943492
No	Spring	Male	No	TplAdmitted	Spontaneous	1.06666665
No	Spring	Male	Yes	No	Spontaneous	-0.1419123
No	Summer	Female	No	No	Spontaneous	1.53975953
No	Summer	Male	No	No	Spontaneous	-0.7426587
No	Summer	Male	No	No	Spontaneous	-1.5252747
No	Winter	Male	No	No	Spontaneous	0.47247783
No	Winter	Male	No	No	Spontaneous	0.93918513
No	Summer	Male	No	No	Spontaneous	0.49840382
No	Spring	Male	No	No	Spontaneous	-0.4158385
No	Winter	Female	Yes	No	Spontaneous	0.3145831

No	Autumn	Female	No	No	Spontaneous	-1.1681094
No	Autumn	Male	No	No	Spontaneous	0.68539553
No	Winter	Male	No	No	Spontaneous	-1.6912438
No	Winter	Female	No	No	Spontaneous	-0.70418
No	Autumn	Female	No	No	Spontaneous	1.05437763
No	Winter	Male	Yes	No	Spontaneous	0.29727226
No	Summer	Female	No	No	Spontaneous	-1.5674754
No	Winter	Male	No	No	Spontaneous	0.48899129
No	Summer	Female	No	No	Spontaneous	0.2991167
No	Autumn	Male	No	No	Spontaneous	-2.2603219
No	Winter	Female	No	No	Spontaneous	0.86032522
No	Winter	Female	No	No	Spontaneous	1.42132646
No	Winter	Male	No	No	Spontaneous	-1.2067324
No	Autumn	Female	No	No	Spontaneous	0.34117135
No	Spring	Male	No	No	Spontaneous	-0.2040408
No	Spring	Female	No	No	Spontaneous	0.29211726
No	Summer	Male	No	No	Spontaneous	-0.8712895
No	Winter	Male	Yes	No	Spontaneous	0.73392327
No	Summer	Female	No	No	Spontaneous	-0.6694863
No	Spring	Female	No	TplAdmitted	Spontaneous	-0.1360367
No	Winter	Female	No	No	Spontaneous	1.03444909
No	Summer	Male	Yes	No	Spontaneous	0.7291981
No	Autumn	Male	No	No	Spontaneous	1.34888433
No	Summer	Male	No	No	Spontaneous	1.46286618
No	Spring	Male	No	No	Spontaneous	-1.045618
No	Summer	Female	No	No	Assisted	1.02292602
No	Autumn	Male	No	No	Spontaneous	-0.3678316
No	Spring	Male	Yes	No	Spontaneous	-0.7956142
No	Autumn	Male	No	No	Spontaneous	0.6595762
No	Winter	Female	Yes	No	Spontaneous	-0.1279479
No	Winter	Female	No	No	Spontaneous	-2.5534193
No	Autumn	Female	No	No	Spontaneous	-0.4848054
No	Autumn	Male	No	No	Spontaneous	0.26069941
Yes	Spring	Female	No	No	Spontaneous	0.98442331
No	Summer	Female	No	No	Spontaneous	-0.3243406
No	Summer	Male	No	TplAdmitted	Spontaneous	-0.9366658
No	Autumn	Female	No	No	Spontaneous	0.96064487
No	Summer	Male	No	No	Spontaneous	1.36240994
No	Winter	Female	No	No	Spontaneous	0.5056675
No	Summer	Male	No	No	Spontaneous	1.76871932
No	Autumn	Female	No	No	Spontaneous	1.31618189
No	Autumn	Male	No	No	Spontaneous	1.73079461
No	Spring	Female	No	No	Spontaneous	1.54817392
No	Spring	Male	No	No	Spontaneous	-0.7016053
No	Spring	Female	No	No	Spontaneous	-0.17919
No	Summer	Female	No	No	Spontaneous	0.36866266
No	Winter	Female	No	No	Spontaneous	0.28271896
No	Winter	Female	No	No	Spontaneous	1.38639145

No	Summer	Female	No	No	Spontaneous	0.6733722
No	Summer	Female	No	No	Spontaneous	1.765856
No	Summer	Male	Yes	No	Spontaneous	-0.3651781
No	Summer	Male	No	No	Spontaneous	0.30133854
No	Autumn	Male	No	No	Spontaneous	1.36165347
No	Summer	Female	No	No	Spontaneous	0.32152287
No	Autumn	Female	No	No	Spontaneous	0.83911208
No	Spring	Male	No	No	Spontaneous	0.63934356
No	Autumn	Male	No	No	Spontaneous	-0.9632574
No	Spring	Male	Yes	No	Spontaneous	0.78452403
No	Summer	Male	No	No	Spontaneous	0.45995117
No	Autumn	Female	No	No	Spontaneous	1.23110109
No	Summer	Male	Yes	No	Spontaneous	0.37657002
No	Spring	Female	No	No	Spontaneous	0.26419108
No	Winter	Male	No	No	Spontaneous	-1.8730337
No	Winter	Male	No	No	Spontaneous	1.84603021
No	Spring	Female	No	No	Spontaneous	-0.856156
No	Summer	Male	No	No	Spontaneous	0.54098723
No	Autumn	Male	No	No	Spontaneous	-0.434284
No	Winter	Male	No	No	Spontaneous	-0.3095632
No	Summer	Female	No	No	Spontaneous	0.7089284
No	Summer	Female	No	No	Spontaneous	0.18598615
No	Summer	Male	No	No	Spontaneous	0.25863613
No	Spring	Male	No	No	Spontaneous	0.82758796
No	Summer	Male	No	No	Spontaneous	0.1332919
No	Spring	Male	No	No	Spontaneous	-0.8476838
No	Spring	Male	No	No	Spontaneous	-0.1385332
No	Summer	Male	Yes	No	Spontaneous	0.10986685
No	Summer	Male	No	No	Spontaneous	1.14122519
No	Winter	Female	No	No	Spontaneous	0.17982556
No	Spring	Female	No	No	Spontaneous	1.87761755
No	Summer	Male	No	No	Assisted	-0.8825692
No	Autumn	Female	No	No	Spontaneous	-0.4621839
No	Spring	Female	No	No	Spontaneous	0.35071423
No	Spring	Male	No	No	Spontaneous	1.23891587
No	Summer	Female	No	TplAdmitted	Spontaneous	-0.2117943
No	Spring	Male	No	No	Spontaneous	2.14035124
No	Summer	Female	No	No	Spontaneous	-0.5410818
No	Autumn	Male	No	No	Spontaneous	1.19219373
No	Autumn	Female	No	No	Spontaneous	0.88300629
No	Autumn	Male	No	No	Spontaneous	-0.0478172
No	Winter	Female	No	No	Spontaneous	-1.8089566
No	Summer	Male	No	No	Spontaneous	0.05969063
No	Winter	Male	Yes	No	Spontaneous	-0.5180886
No	Spring	Female	Yes	No	Spontaneous	-0.5197352
No	Summer	Male	Yes	No	Spontaneous	0.93173896
No	Autumn	Female	No	No	Assisted	-0.5759209
No	Spring	Male	No	No	Spontaneous	0.55732232

No	Winter	Female	No	No	Spontaneous	2.9023119
No	Spring	Male	Yes	No	Spontaneous	0.37045877
No	Summer	Male	Yes	No	Spontaneous	-0.1058699
No	Summer	Female	No	No	Spontaneous	-1.0861262
No	Summer	Male	No	No	Spontaneous	0.38566153
No	Summer	Male	No	No	Spontaneous	0.46900073
No	Winter	Female	No	No	Spontaneous	-1.1340505
No	Winter	Male	No	No	Spontaneous	0.52634557
No	Winter	Female	No	No	Spontaneous	0.42428667
No	Spring	Female	Yes	No	Spontaneous	-0.2690899
No	Winter	Female	No	No	Spontaneous	-0.2961461
No	Winter	Female	Yes	No	Spontaneous	0.664305
No	Summer	Female	No	No	Spontaneous	-0.2841897
No	Winter	Female	No	No	Spontaneous	0.80950059
No	Winter	Male	No	No	Spontaneous	-0.039124
No	Winter	Male	Yes	No	Spontaneous	-1.6616126
No	Summer	Male	No	No	Spontaneous	-1.4145313
No	Winter	Male	No	No	Spontaneous	1.75118483
No	Autumn	Female	No	No	Spontaneous	-0.632184
No	Winter	Female	No	No	Assisted	0.87025354
No	Winter	Male	Yes	No	Spontaneous	-0.0230177
No	Summer	Female	No	No	Spontaneous	-0.1665252
No	Summer	Male	No	No	Assisted	-0.7548297
No	Spring	Male	Yes	No	Spontaneous	-1.5367742
No	Spring	Female	Yes	No	Spontaneous	0.00540248
No	Summer	Male	No	No	Spontaneous	1.26396335
No	Spring	Male	Yes	No	Spontaneous	1.23594159
No	Summer	Female	No	No	Assisted	-1.1605003
No	Autumn	Female	No	No	Spontaneous	0.69905323
No	Winter	Female	Yes	No	Spontaneous	-1.2500522
No	Autumn	Male	No	No	Spontaneous	-1.4070888
No	Winter	Male	No	No	Spontaneous	1.39921587
No	Spring	Female	No	No	Spontaneous	0.93436744
No	Spring	Male	No	No	Spontaneous	-0.817488
No	Spring	Male	Yes	No	Spontaneous	0.12031774
No	Spring	Male	No	No	Spontaneous	-0.0925009
No	Autumn	Female	Yes	No	Assisted	-0.3779334
No	Summer	Female	Yes	No	Spontaneous	-1.481228
No	Spring	Male	Yes	No	Spontaneous	-0.4161199
No	Spring	Male	No	No	Spontaneous	-0.7421874
No	Autumn	Male	No	No	Spontaneous	0.21309545
No	Spring	Male	Yes	No	Spontaneous	0.6637153
No	Autumn	Female	No	No	Spontaneous	0.49692334
No	Summer	Female	No	No	Spontaneous	1.5638984
No	Autumn	Female	No	No	Spontaneous	-0.6143366
No	Summer	Male	No	No	Spontaneous	1.69111911
No	Winter	Female	No	No	Spontaneous	-0.0325969
No	Winter	Male	No	No	Spontaneous	-0.1712861

No	Spring	Male	No	No	Spontaneous	2.25379894
No	Summer	Female	Yes	No	Spontaneous	0.82002442
No	Summer	Female	No	No	Spontaneous	0.09038925
Yes	Spring	Female	No	No	Spontaneous	-0.1782699
No	Summer	Female	No	No	Spontaneous	0.69905323
No	Spring	Male	No	No	Spontaneous	1.7716109
No	Autumn	Female	Yes	No	Spontaneous	-2.4160554
No	Summer	Male	No	No	Assisted	-1.0412052
No	Winter	Male	No	No	Spontaneous	-0.246372
No	Autumn	Male	Yes	No	Spontaneous	-1.0847725
No	Autumn	Female	Yes	No	Spontaneous	0.44455757
No	Winter	Male	No	No	Spontaneous	0.60082751
No	Winter	Male	Yes	No	Spontaneous	0.99898885
No	Winter	Female	No	No	Spontaneous	-0.2852322
No	Summer	Male	Yes	No	Spontaneous	-0.6047957
No	Summer	Male	No	No	Spontaneous	1.51155665
No	Summer	Male	Yes	No	Spontaneous	-0.4399826
No	Summer	Male	No	No	Spontaneous	0.44839863
No	Autumn	Male	Yes	No	Spontaneous	-0.0330709
No	Autumn	Male	No	No	Spontaneous	-2.1136965
No	Spring	Male	No	No	Spontaneous	0.88559529
No	Spring	Male	No	No	Spontaneous	-1.1140439
No	Winter	Male	No	No	Spontaneous	-0.8633284
No	Autumn	Male	No	No	Spontaneous	0.60084601
No	Summer	Male	No	No	Spontaneous	0.01301759
No	Winter	Female	Yes	No	Spontaneous	-0.7996907
No	Winter	Female	Yes	No	Spontaneous	-0.7929401
No	Summer	Male	Yes	No	Spontaneous	-1.2186119
Yes	Summer	Female	Yes	No	Spontaneous	0.65158811
No	Summer	Male	No	No	Spontaneous	0.95707798
No	Summer	Male	Yes	No	Spontaneous	1.72743261
No	Autumn	Male	No	No	Spontaneous	-0.2625985
No	Winter	Female	No	No	Spontaneous	-0.7033714
No	Spring	Female	No	No	Spontaneous	0.72391881
No	Summer	Male	No	No	Spontaneous	1.49524796
No	Spring	Female	No	No	Spontaneous	0.57794016
No	Spring	Male	No	No	Spontaneous	0.58078147
No	Autumn	Female	No	No	Spontaneous	0.42209201
No	Summer	Female	No	No	Spontaneous	0.20173518
No	Summer	Female	No	No	Spontaneous	1.29871401
No	Spring	Female	No	No	Spontaneous	0.46386215
No	Autumn	Female	No	No	Spontaneous	0.63925971
No	Winter	Female	No	No	Spontaneous	0.59359236
No	Winter	Male	No	No	Spontaneous	0.81465441
No	Summer	Female	Yes	No	Spontaneous	-1.0037486
No	Autumn	Female	No	No	Spontaneous	-0.5093505
No	Autumn	Female	No	No	Assisted	0.16797534
No	Summer	Female	No	No	Spontaneous	0.22096323

No	Winter	Male	Yes	No	Spontaneous	0.00718597
No	Winter	Female	No	No	Spontaneous	-1.839861
No	Autumn	Male	No	No	Spontaneous	0.86079181
No	Winter	Male	No	No	Spontaneous	0.55863936
No	Summer	Female	Yes	No	Spontaneous	-1.4464038
No	Summer	Female	No	TplAdmitted	Spontaneous	0.59585188
No	Summer	Male	Yes	No	Spontaneous	0.42455049
No	Spring	Female	No	No	Assisted	0.54132783
No	Spring	Male	No	No	Spontaneous	0.36268384
No	Spring	Female	No	No	Spontaneous	0.97831523
No	Autumn	Female	No	No	Spontaneous	-0.3658501
No	Winter	Male	No	No	Spontaneous	-0.3502554
No	Winter	Female	No	No	Spontaneous	0.75118922
No	Winter	Male	No	No	Spontaneous	-0.6898302
No	Spring	Female	No	No	Spontaneous	-0.6580109
No	Winter	Male	No	No	Spontaneous	0.01926756
No	Summer	Female	No	No	Spontaneous	-0.2217092
No	Winter	Female	Yes	No	Spontaneous	0.56282804
No	Winter	Male	No	No	Spontaneous	0.7616803
No	Autumn	Male	No	No	Spontaneous	0.79603456
No	Summer	Male	No	No	Spontaneous	-0.1285612
No	Summer	Female	No	TplAdmitted	Spontaneous	1.75835815
No	Spring	Male	No	No	Spontaneous	-0.911084
No	Winter	Male	No	No	Spontaneous	0.46954676
No	Spring	Female	No	No	Spontaneous	-1.7178977
No	Autumn	Female	No	No	Spontaneous	1.34613593
No	Winter	Female	No	No	Spontaneous	-1.3655243
No	Autumn	Male	No	No	Spontaneous	-1.2950393
No	Autumn	Male	No	No	Spontaneous	0.06606589
No	Spring	Female	No	No	Spontaneous	0.86529318
No	Autumn	Male	No	No	Spontaneous	-0.4382919
No	Winter	Male	No	No	Spontaneous	0.71891971
No	Spring	Female	No	No	Spontaneous	-0.3544196
No	Spring	Female	No	No	Spontaneous	1.2978227
No	Summer	Female	No	No	Spontaneous	-0.9917907
No	Winter	Female	No	No	Spontaneous	0.62577877
No	Spring	Female	No	No	Spontaneous	0.26655978
No	Spring	Male	No	No	Spontaneous	-0.9978306
No	Winter	Female	Yes	No	Spontaneous	-0.4478912
No	Spring	Female	No	No	Spontaneous	0.2673333
No	Winter	Male	Yes	No	Spontaneous	0.55188695
No	Summer	Female	No	No	Spontaneous	-0.2547039
No	Spring	Male	No	No	Spontaneous	-0.4322231
No	Spring	Female	No	No	Spontaneous	-1.5134945
No	Winter	Male	Yes	No	Spontaneous	-1.5240004
No	Summer	Male	No	No	Spontaneous	-0.2408715
No	Winter	Male	No	No	Spontaneous	1.49190012
No	Winter	Female	No	No	Spontaneous	-1.1131321

No	Winter	Male	Yes	No	Spontaneous	-0.5517363
No	Spring	Female	No	No	Spontaneous	-0.5473465
No	Summer	Female	No	No	Spontaneous	-1.9746127
No	Winter	Female	No	No	Spontaneous	-0.7492482
No	Winter	Male	No	TplAdmitted	Spontaneous	-0.6055962
No	Spring	Female	No	No	Spontaneous	-0.5901771
No	Autumn	Male	No	TplAdmitted	Spontaneous	0.15926223
No	Winter	Female	Yes	No	Spontaneous	-1.9375834
No	Summer	Female	No	No	Spontaneous	1.17723905
No	Autumn	Female	No	No	Spontaneous	1.11504251
No	Winter	Male	No	No	Spontaneous	1.0970282
No	Winter	Female	No	No	Spontaneous	1.02878928
No	Winter	Male	No	No	Spontaneous	-0.1897685
No	Winter	Male	No	No	Spontaneous	1.68790116
No	Winter	Male	No	No	Spontaneous	-2.7649836
No	Autumn	Male	Yes	No	Spontaneous	-0.1754676
No	Autumn	Male	Yes	No	Spontaneous	-0.2162148
No	Spring	Male	No	No	Spontaneous	-0.5477
No	Spring	Female	No	No	Spontaneous	-1.0240612
No	Autumn	Female	No	No	Spontaneous	0.63838945
No	Spring	Male	No	No	Spontaneous	0.59635343
No	Autumn	Male	Yes	No	Spontaneous	0.29229772
No	Spring	Female	Yes	No	Spontaneous	-0.9872879
No	Spring	Male	No	No	Spontaneous	-1.147587
No	Autumn	Female	No	No	Spontaneous	1.45136825
No	Summer	Female	No	No	Spontaneous	0.7752972
No	Winter	Male	No	No	Spontaneous	-0.9142296
No	Spring	Female	No	No	Spontaneous	0.43719669
Yes	Summer	Male	No	No	Spontaneous	1.71988474
No	Winter	Male	Yes	No	Spontaneous	-1.0331735
No	Autumn	Male	No	No	Spontaneous	0.16508549
No	Summer	Male	No	No	Spontaneous	-0.8051176
No	Spring	Male	No	No	Spontaneous	0.09578922
No	Summer	Female	No	No	Spontaneous	0.08646127
No	Autumn	Male	No	No	Spontaneous	-1.1230617
No	Winter	Female	No	No	Spontaneous	-0.5967458
No	Spring	Female	No	No	Spontaneous	1.35619292
No	Winter	Male	No	No	Spontaneous	-0.7735598
No	Summer	Female	No	No	Spontaneous	-0.5978541
No	Winter	Male	No	No	Spontaneous	-0.2071772
No	Winter	Female	Yes	No	Spontaneous	-3.1964663
No	Winter	Female	No	No	Spontaneous	0.08978573
No	Spring	Male	No	No	Spontaneous	1.258804
No	Winter	Female	No	No	Spontaneous	0.58457783
No	Summer	Male	No	No	Spontaneous	1.22846975
No	Winter	Male	No	No	Spontaneous	-1.1908305
No	Autumn	Female	No	No	Spontaneous	0.29595108
No	Autumn	Female	Yes	No	Spontaneous	0.52377937

No	Winter	Male	No	No	Spontaneous	0.28260682
No	Summer	Male	No	No	Spontaneous	-0.1104839
No	Autumn	Female	No	No	Spontaneous	0.0820766
No	Winter	Female	No	No	Spontaneous	-1.1197874
No	Winter	Male	No	No	Assisted	0.50562919
No	Autumn	Female	Yes	No	Spontaneous	-0.426862
No	Winter	Female	No	No	Spontaneous	0.49473176
No	Spring	Female	No	No	Spontaneous	-0.1417922
No	Summer	Female	Yes	No	Spontaneous	0.14257132
No	Spring	Male	No	No	Spontaneous	0.51780561
No	Winter	Male	No	No	Spontaneous	-0.3547933
No	Summer	Male	No	No	Spontaneous	-1.1696666
No	Summer	Male	Yes	No	Spontaneous	1.01279583
No	Winter	Male	No	No	Spontaneous	2.3332655
No	Autumn	Male	No	No	Spontaneous	-0.1699661
No	Summer	Male	No	No	Spontaneous	0.44839863
No	Autumn	Male	Yes	No	Spontaneous	-0.6728513
No	Winter	Male	No	No	Spontaneous	-0.2583919
No	Autumn	Male	No	No	Spontaneous	1.73426909
No	Autumn	Female	No	No	Spontaneous	2.06904298
No	Spring	Female	No	No	Spontaneous	0.37821433
No	Spring	Female	No	No	Spontaneous	0.71716482
No	Spring	Male	Yes	No	Spontaneous	0.93251776
No	Summer	Female	No	No	Spontaneous	-0.130739
No	Autumn	Male	No	No	Spontaneous	0.7181056
No	Summer	Male	No	No	Spontaneous	-1.4737263
No	Summer	Female	No	No	Spontaneous	0.94317447
No	Autumn	Female	No	No	Spontaneous	0.4625057
No	Spring	Female	Yes	No	Spontaneous	-0.2646872
No	Spring	Male	No	No	Spontaneous	-1.1358683
No	Winter	Male	No	No	Spontaneous	-0.4546061
No	Winter	Male	No	No	Spontaneous	0.79975319
No	Winter	Male	No	No	Spontaneous	-0.6004696
No	Winter	Female	Yes	No	Spontaneous	-0.7297139
No	Spring	Female	Yes	No	Spontaneous	-1.1552865
No	Summer	Male	No	No	Spontaneous	1.62178184
No	Winter	Female	No	No	Spontaneous	-0.335194
No	Autumn	Female	No	No	Spontaneous	0.1867149
No	Spring	Male	No	No	Spontaneous	0.47247783
No	Autumn	Male	No	No	Spontaneous	-0.4060637
No	Summer	Female	Yes	No	Spontaneous	-1.4258948
No	Summer	Male	No	No	Spontaneous	0.33658894
No	Spring	Male	No	TplAdmitted	Spontaneous	-2.5100125
No	Autumn	Female	No	No	Spontaneous	0.78507259
No	Autumn	Female	No	No	Spontaneous	-1.3473003
No	Summer	Female	No	No	Spontaneous	-0.6467754
No	Spring	Female	No	No	Spontaneous	1.29579965
No	Autumn	Male	Yes	No	Spontaneous	-0.5211453

No	Spring	Male	Yes	No	Spontaneous	0.67624723
No	Winter	Male	No	No	Spontaneous	1.69077712
No	Summer	Male	No	No	Spontaneous	-0.3791857
No	Spring	Female	No	TplAdmitted	Spontaneous	0.27595548
No	Winter	Female	No	No	Spontaneous	0.48653267
No	Winter	Female	No	No	Spontaneous	-1.967179
No	Spring	Male	No	No	Spontaneous	-1.6354709
No	Winter	Male	No	No	Spontaneous	0.07553432
No	Winter	Male	No	No	Spontaneous	0.36512061
No	Summer	Male	No	No	Spontaneous	-0.9258146
No	Summer	Male	No	No	Spontaneous	0.57502053
No	Autumn	Male	No	No	Spontaneous	0.02492888
No	Summer	Male	No	No	Spontaneous	0.67435009
No	Autumn	Male	No	No	Spontaneous	1.46992019
No	Autumn	Male	No	No	Spontaneous	-0.8443527
No	Winter	Male	Yes	No	Spontaneous	-1.6619413
No	Winter	Female	No	No	Spontaneous	0.81125412
Yes	Autumn	Male	No	TplAdmitted	Spontaneous	1.75633263
No	Summer	Female	No	No	Spontaneous	-0.546473
No	Winter	Male	Yes	TplAdmitted	Spontaneous	-0.392955
No	Winter	Female	No	No	Spontaneous	0.13586531
No	Spring	Male	No	No	Spontaneous	1.18505435
No	Winter	Male	No	No	Spontaneous	-0.0588014
No	Spring	Male	No	No	Spontaneous	-0.6626415
No	Autumn	Female	No	No	Spontaneous	1.3629539
No	Autumn	Female	No	No	Spontaneous	1.52846103
No	Winter	Female	No	No	Spontaneous	0.45902494
No	Winter	Male	Yes	No	Spontaneous	-0.4243101
No	Summer	Female	No	No	Spontaneous	-0.175218
Yes	Summer	Female	No	No	Spontaneous	-0.8359983
No	Summer	Male	No	No	Spontaneous	-1.0412044
No	Spring	Male	No	No	Spontaneous	-0.712401
Yes	Spring	Male	Yes	TplAdmitted	Spontaneous	1.46225009
No	Autumn	Male	No	No	Spontaneous	0.90292989
No	Autumn	Male	No	No	Spontaneous	-1.8403122
No	Autumn	Male	No	No	Spontaneous	-0.3711469
No	Winter	Male	Yes	No	Spontaneous	0.19921136
No	Autumn	Male	No	No	Spontaneous	-0.7018832
No	Autumn	Female	No	No	Spontaneous	0.0818967
No	Summer	Male	No	No	Spontaneous	-1.1925123
No	Summer	Female	No	TplAdmitted	Assisted	0.4183217
No	Spring	Male	No	No	Spontaneous	-0.7630762
No	Autumn	Male	No	No	Spontaneous	1.4368823
No	Autumn	Male	Yes	No	Spontaneous	-0.7517296
No	Spring	Female	No	No	Spontaneous	0.18044614
No	Summer	Female	No	No	Spontaneous	0.39561763
No	Spring	Female	No	No	Spontaneous	0.62833958
No	Autumn	Male	No	No	Spontaneous	0.56593896

No	Summer	Male	Yes	No	Spontaneous	0.418772
No	Spring	Male	No	No	Spontaneous	1.59662005
No	Winter	Female	No	No	Spontaneous	0.4146105
No	Summer	Female	No	No	Spontaneous	0.77350986
No	Spring	Male	No	No	Spontaneous	0.05438173
No	Winter	Male	No	No	Spontaneous	-0.1317469
No	Autumn	Male	No	No	Spontaneous	-1.2512292
No	Winter	Male	No	TplAdmitted	Spontaneous	-0.402892
No	Summer	Female	Yes	No	Spontaneous	-0.7389007
No	Autumn	Female	No	No	Spontaneous	0.71524558
No	Autumn	Female	No	No	Assisted	0.09214653
No	Winter	Male	No	No	Spontaneous	-0.1951141
No	Spring	Male	No	No	Spontaneous	-1.0371624
No	Autumn	Female	No	No	Spontaneous	-0.9146089
No	Spring	Male	No	No	Spontaneous	0.12688294
No	Winter	Male	No	No	Spontaneous	0.70041251
No	Spring	Male	No	No	Spontaneous	-0.7929182
No	Autumn	Male	No	No	Spontaneous	-2.4065773
No	Spring	Female	Yes	No	Spontaneous	0.60502842
No	Spring	Female	No	No	Spontaneous	2.17612904
Yes	Spring	Male	No	No	Spontaneous	-0.8401753
No	Spring	Female	No	No	Spontaneous	0.07933654
No	Spring	Female	No	No	Spontaneous	1.72679338
No	Winter	Male	No	No	Spontaneous	0.45003691
No	Summer	Male	No	No	Spontaneous	0.29737411
No	Spring	Female	No	No	Spontaneous	0.24548174
No	Autumn	Female	No	No	Spontaneous	0.13294216
No	Autumn	Male	No	No	Spontaneous	0.75167539
No	Spring	Female	No	No	Spontaneous	1.26770233
No	Autumn	Male	No	No	Spontaneous	0.37718718
No	Summer	Male	Yes	No	Spontaneous	-0.0109194
No	Winter	Female	No	No	Spontaneous	0.4549927
No	Spring	Male	No	No	Spontaneous	2.25796115
No	Summer	Male	Yes	No	Spontaneous	1.44380888
No	Winter	Female	No	No	Spontaneous	-2.3955822
No	Summer	Male	No	No	Spontaneous	-1.4210159
No	Spring	Male	No	No	Spontaneous	-0.5528347
No	Autumn	Female	No	No	Spontaneous	-0.9278195
No	Spring	Male	No	TplAdmitted	Assisted	0.20373588
No	Autumn	Female	Yes	No	Spontaneous	-0.0834432
No	Winter	Male	Yes	No	Spontaneous	0.0076881
No	Autumn	Male	No	No	Assisted	-0.7058959
No	Winter	Female	No	No	Spontaneous	1.44956247
No	Autumn	Female	No	No	Spontaneous	-0.4092224
No	Spring	Female	No	No	Spontaneous	-0.4663784
No	Spring	Male	No	No	Spontaneous	0.31034187
No	Autumn	Female	No	No	Spontaneous	0.45902494
No	Autumn	Male	No	No	Spontaneous	-0.0281072

No	Spring	Female	No	No	Spontaneous	0.4134227
No	Summer	Female	No	No	Spontaneous	0.59164113
No	Spring	Female	No	No	Spontaneous	1.39055617
No	Autumn	Male	Yes	No	Spontaneous	-0.0754751
No	Summer	Female	No	No	Spontaneous	-0.4362531
No	Winter	Male	No	No	Spontaneous	0.74553375
No	Autumn	Female	No	No	Spontaneous	-1.4994189
No	Winter	Female	No	No	Spontaneous	0.19778434
No	Winter	Male	No	No	Spontaneous	-0.5326301
No	Winter	Female	No	No	Spontaneous	1.56647164
No	Spring	Female	No	No	Spontaneous	0.4697464
No	Spring	Female	Yes	No	Spontaneous	-0.723739
No	Winter	Male	No	No	Spontaneous	1.17978802
No	Winter	Female	Yes	No	Spontaneous	0.45213934
No	Autumn	Female	No	No	Spontaneous	-0.9654346
No	Summer	Male	No	No	Spontaneous	1.1287361
No	Summer	Male	No	No	Spontaneous	-0.5322347
No	Summer	Male	No	No	Spontaneous	0.64408179
No	Winter	Female	No	No	Spontaneous	0.75172257
No	Winter	Male	No	No	Spontaneous	0.31077516
No	Summer	Female	Yes	No	Spontaneous	-1.0465026
No	Summer	Female	No	No	Spontaneous	0.48566883
No	Winter	Male	No	No	Spontaneous	2.2693584
No	Summer	Female	No	No	Spontaneous	-0.2931785
No	Spring	Male	No	No	Spontaneous	0.33413527
No	Summer	Male	Yes	No	Assisted	0.1003952
No	Autumn	Female	No	No	Spontaneous	0.55317762
No	Spring	Male	No	No	Spontaneous	-0.1183006
No	Spring	Female	No	No	Spontaneous	-0.2021796
No	Autumn	Male	Yes	No	Spontaneous	-0.9192041
No	Summer	Male	No	No	Spontaneous	-1.0538476
No	Winter	Female	No	TplAdmitted	Spontaneous	0.11371332
No	Spring	Female	No	No	Spontaneous	-1.4865642
No	Spring	Male	No	No	Spontaneous	-0.6936922
No	Autumn	Male	No	No	Spontaneous	-0.5922955
No	Winter	Male	No	No	Spontaneous	-0.7331091
No	Winter	Male	No	No	Spontaneous	0.05936475
No	Spring	Male	No	No	Spontaneous	1.49365668
No	Autumn	Male	No	No	Spontaneous	-0.554235
No	Summer	Female	Yes	No	Spontaneous	-1.3630813
No	Winter	Male	No	No	Spontaneous	-1.8597428
No	Autumn	Female	No	No	Spontaneous	-0.6042718
No	Winter	Male	No	No	Spontaneous	1.6151754
No	Spring	Male	No	No	Spontaneous	-1.3095791
No	Autumn	Male	No	No	Spontaneous	-0.0931904
No	Summer	Male	No	No	Spontaneous	-1.114888
No	Winter	Female	No	No	Spontaneous	-0.5031703
No	Winter	Male	No	No	Spontaneous	0.13224043

No	Winter	Male	Yes	No	Spontaneous	-0.2420589
No	Autumn	Female	Yes	No	Spontaneous	0.35071423
No	Autumn	Female	No	No	Spontaneous	0.88019246
No	Autumn	Male	Yes	No	Spontaneous	-0.4583764
No	Summer	Female	No	TplAdmitted	Spontaneous	-1.660544
No	Summer	Male	No	No	Spontaneous	0.64810131
No	Autumn	Male	Yes	No	Spontaneous	0.70197122
No	Autumn	Male	No	No	Spontaneous	0.87631668
No	Autumn	Male	No	No	Spontaneous	0.09670291
No	Winter	Male	Yes	No	Spontaneous	1.21021979
No	Autumn	Female	No	No	Spontaneous	-0.3586719
No	Winter	Female	No	No	Spontaneous	0.61820472
No	Summer	Male	No	No	Spontaneous	0.26103138
No	Summer	Male	No	No	Spontaneous	-1.4263624
No	Spring	Male	Yes	No	Spontaneous	-0.597449
No	Autumn	Male	No	No	Spontaneous	1.67516859
No	Winter	Male	Yes	No	Spontaneous	-1.3798164
No	Summer	Male	No	No	Spontaneous	0.70621657
No	Summer	Male	Yes	No	Spontaneous	0.47574982
No	Spring	Male	No	No	Spontaneous	0.1759514
No	Summer	Male	No	No	Spontaneous	-0.6417355
No	Autumn	Male	Yes	No	Spontaneous	-2.2628434
No	Summer	Female	No	No	Spontaneous	-1.6564289
No	Spring	Female	No	No	Spontaneous	2.58133261
No	Autumn	Male	No	No	Spontaneous	0.99019376
No	Winter	Female	No	No	Spontaneous	-1.0046586
No	Spring	Female	Yes	No	Spontaneous	-1.0693108
No	Summer	Male	No	No	Spontaneous	0.34805933
No	Winter	Female	Yes	No	Spontaneous	-0.2545012
No	Summer	Female	No	No	Spontaneous	-0.7845361
No	Spring	Male	No	No	Spontaneous	0.31468392
No	Winter	Male	No	No	Spontaneous	0.05324681
No	Autumn	Male	No	No	Spontaneous	1.37697245
No	Summer	Male	No	No	Spontaneous	-0.3233434
No	Spring	Female	No	No	Spontaneous	0.49841333
No	Autumn	Male	No	No	Spontaneous	0.49427452
No	Spring	Male	No	No	Spontaneous	1.39949425
No	Summer	Female	Yes	No	Spontaneous	0.78996418
No	Summer	Female	No	No	Spontaneous	1.8926459
No	Summer	Male	No	No	Spontaneous	0.61393553
No	Spring	Male	No	No	Spontaneous	-0.6429432
No	Winter	Male	No	No	Spontaneous	-1.5609861
No	Spring	Female	No	No	Spontaneous	0.44819616
No	Summer	Female	No	No	Spontaneous	1.48413087
No	Spring	Male	No	No	Spontaneous	-0.0439188
No	Spring	Male	No	No	Spontaneous	1.37584798
No	Autumn	Male	No	No	Spontaneous	-0.5671584
No	Winter	Female	No	TplAdmitted	Spontaneous	0.19021631

No	Winter	Female	No	No	Spontaneous	-1.7342337
No	Summer	Female	No	No	Spontaneous	-0.8496884
No	Autumn	Female	No	No	Spontaneous	-0.8429291
No	Autumn	Male	No	No	Spontaneous	-1.5876036
No	Spring	Male	No	No	Spontaneous	1.05223747
No	Summer	Male	No	No	Spontaneous	0.5725296
No	Summer	Female	Yes	No	Spontaneous	-0.5884572
No	Autumn	Female	No	No	Spontaneous	-0.9386708
No	Winter	Male	No	No	Spontaneous	0.75019552
No	Winter	Male	No	No	Spontaneous	-0.5031736
Yes	Autumn	Male	No	TplAdmitted	Spontaneous	0.30380523
No	Autumn	Male	No	No	Spontaneous	-0.5366357
No	Autumn	Female	No	No	Spontaneous	0.85291426
No	Autumn	Female	No	No	Spontaneous	1.34014123
No	Winter	Male	No	No	Spontaneous	0.07589752
No	Spring	Female	No	No	Spontaneous	0.23716399
No	Spring	Male	No	No	Spontaneous	1.83825031
No	Winter	Male	No	No	Spontaneous	0.41193171
No	Summer	Male	No	No	Spontaneous	-1.2418124
No	Spring	Female	No	No	Spontaneous	0.29415555
No	Winter	Male	No	No	Spontaneous	0.03265562
No	Summer	Female	No	No	Assisted	-0.8355737
No	Summer	Female	No	No	Assisted	0.40874218
No	Autumn	Male	No	No	Spontaneous	0.17905792
No	Spring	Male	No	No	Spontaneous	1.80790522
No	Summer	Male	No	No	Spontaneous	1.9736635
No	Winter	Female	No	No	Spontaneous	-1.2217503
No	Spring	Male	No	TplAdmitted	Assisted	1.3513485
No	Spring	Female	No	No	Spontaneous	1.20471438
No	Spring	Female	No	No	Spontaneous	-0.9518164
No	Spring	Female	No	No	Spontaneous	0.70907349
No	Summer	Male	Yes	No	Spontaneous	0.51147824
No	Summer	Male	No	No	Spontaneous	-0.2838304
No	Spring	Male	No	No	Assisted	-1.2627072
No	Autumn	Male	No	No	Spontaneous	1.92293404
Yes	Spring	Male	No	No	Spontaneous	0.48247069
No	Winter	Female	No	No	Spontaneous	1.26848797
No	Winter	Male	No	No	Spontaneous	-0.9390341
No	Spring	Male	No	No	Spontaneous	-0.0024251
No	Autumn	Male	No	No	Spontaneous	0.22442018
No	Autumn	Male	Yes	No	Spontaneous	-0.9898948
No	Autumn	Female	No	No	Spontaneous	0.44548946
No	Summer	Female	No	No	Spontaneous	-0.8964523
No	Spring	Female	Yes	No	Spontaneous	-1.0017462
No	Summer	Female	No	No	Spontaneous	1.11504251
No	Autumn	Female	Yes	No	Spontaneous	2.023334
No	Summer	Male	No	No	Assisted	0.38129695
No	Autumn	Female	No	No	Spontaneous	-1.2717477

No	Autumn	Female	Yes	No	Spontaneous	0.50548281
No	Winter	Male	Yes	No	Spontaneous	-0.8526264
No	Spring	Female	No	No	Spontaneous	2.10699331
No	Winter	Male	No	No	Spontaneous	0.51518956
No	Summer	Female	No	No	Spontaneous	-1.7940086
No	Spring	Female	No	No	Spontaneous	0.66976889
No	Autumn	Male	Yes	No	Spontaneous	-0.0705069
No	Winter	Female	Yes	No	Spontaneous	0.2987114
No	Spring	Female	Yes	No	Spontaneous	0.03812
No	Autumn	Male	Yes	No	Spontaneous	0.55771852
No	Autumn	Female	No	No	Spontaneous	0.73311118
No	Summer	Female	No	No	Spontaneous	-1.217573
No	Spring	Male	Yes	No	Spontaneous	0.49118123
No	Winter	Female	No	No	Spontaneous	-0.4017086
No	Spring	Female	No	No	Spontaneous	-1.8784845
No	Winter	Male	No	No	Assisted	0.53286973
No	Autumn	Male	No	No	Spontaneous	-1.050093
No	Autumn	Male	No	No	Spontaneous	-0.8803125
No	Spring	Female	No	No	Spontaneous	-0.4495867
Yes	Autumn	Male	No	No	Spontaneous	-0.0816935
No	Autumn	Male	No	No	Spontaneous	-0.5148448
No	Summer	Female	No	No	Spontaneous	0.54678922
No	Winter	Female	No	No	Assisted	-0.2625685
No	Autumn	Male	No	No	Spontaneous	-0.3985148
No	Autumn	Female	Yes	No	Spontaneous	-0.0667622
No	Autumn	Male	No	No	Spontaneous	-1.2933023
No	Spring	Female	No	No	Spontaneous	-0.2717961
No	Winter	Female	Yes	No	Spontaneous	0.1559634
No	Summer	Male	No	No	Spontaneous	-0.2230354
No	Autumn	Female	Yes	No	Spontaneous	-0.8079582
No	Autumn	Male	No	No	Spontaneous	-0.5917582
No	Summer	Female	No	No	Spontaneous	1.53314923
No	Spring	Female	Yes	No	Spontaneous	-0.4164872
Yes	Spring	Male	No	TplAdmitted	Spontaneous	0.48016634
No	Winter	Female	No	No	Spontaneous	-1.3803738
No	Spring	Male	No	No	Spontaneous	1.18950232
Yes	Winter	Female	No	TplAdmitted	Spontaneous	0.43087244
No	Autumn	Male	No	No	Spontaneous	-0.149527
No	Spring	Male	No	No	Spontaneous	1.39076704
No	Spring	Female	No	No	Spontaneous	0.93044348
No	Summer	Female	Yes	No	Spontaneous	-1.146545
No	Summer	Female	Yes	No	Spontaneous	-0.6277683
No	Summer	Male	No	No	Spontaneous	-0.5791033
No	Autumn	Male	Yes	No	Spontaneous	0.66655376
No	Spring	Female	No	No	Spontaneous	0.93838213
No	Autumn	Female	No	No	Spontaneous	-1.1889003
No	Summer	Male	No	No	Spontaneous	-0.5057094
No	Winter	Female	No	No	Spontaneous	1.66843156

No	Winter	Male	No	No	Spontaneous	-0.3225929
No	Autumn	Male	No	No	Assisted	0.51117772
No	Spring	Male	Yes	No	Spontaneous	-0.3249401
No	Winter	Female	No	No	Spontaneous	0.46344903
No	Winter	Female	No	No	Spontaneous	1.18544322
No	Summer	Male	No	No	Spontaneous	-0.1073252
No	Autumn	Female	Yes	TplAdmitted	Spontaneous	-1.5056572
No	Autumn	Male	No	No	Spontaneous	0.47711982
No	Autumn	Male	No	No	Assisted	1.06772101
No	Autumn	Male	No	No	Spontaneous	0.07185897
No	Spring	Female	No	No	Spontaneous	0.09396428
No	Winter	Male	No	No	Spontaneous	-0.237477
No	Autumn	Male	No	No	Spontaneous	-0.1838343
No	Summer	Male	No	No	Spontaneous	1.26639275
No	Winter	Male	No	No	Spontaneous	0.57400528
No	Autumn	Female	Yes	No	Spontaneous	-0.8334253
No	Winter	Male	No	No	Spontaneous	0.60846848
No	Spring	Female	No	No	Spontaneous	-1.1263802
No	Summer	Male	Yes	No	Spontaneous	-1.0663135
No	Autumn	Male	No	No	Spontaneous	-1.1219971
No	Autumn	Female	No	No	Spontaneous	-1.1350471
No	Autumn	Male	No	No	Spontaneous	0.45933461
No	Spring	Male	No	No	Spontaneous	0.96589563
No	Autumn	Female	Yes	No	Spontaneous	0.58019999
No	Autumn	Male	No	No	Spontaneous	-0.0299381
No	Spring	Male	No	No	Spontaneous	-1.0264654
No	Autumn	Male	No	No	Spontaneous	0.06489892
No	Winter	Female	No	No	Spontaneous	1.1296761
No	Winter	Male	No	No	Spontaneous	0.25863613
No	Autumn	Male	No	No	Spontaneous	-0.6347532
No	Spring	Male	No	No	Spontaneous	-0.442638
No	Autumn	Male	No	No	Spontaneous	0.06156631
No	Spring	Male	No	TplAdmitted	Spontaneous	-1.3762681
No	Winter	Male	No	No	Spontaneous	-0.2754582
No	Spring	Female	No	No	Spontaneous	-0.9139125
No	Summer	Male	Yes	No	Spontaneous	0.31398512
No	Autumn	Female	No	No	Spontaneous	0.57975031
No	Autumn	Male	Yes	No	Spontaneous	0.48322897
No	Summer	Male	No	No	Spontaneous	-0.2632503
No	Winter	Female	No	No	Spontaneous	-0.2247543
No	Autumn	Female	No	No	Spontaneous	-0.5081567
Yes	Autumn	Male	No	No	Spontaneous	-0.7867461
No	Winter	Male	No	No	Spontaneous	-0.2694034
No	Winter	Male	No	No	Spontaneous	-1.361271
No	Autumn	Male	Yes	No	Spontaneous	-1.305346
No	Winter	Female	Yes	No	Spontaneous	-0.0564318
No	Spring	Female	Yes	No	Spontaneous	-0.761679
No	Winter	Female	No	No	Spontaneous	0.30367017

No	Spring	Female	Yes	No	Spontaneous	-0.6304209
No	Autumn	Female	Yes	No	Spontaneous	-0.7813352
No	Autumn	Female	No	No	Assisted	-0.0496485
No	Winter	Male	No	No	Spontaneous	0.73163406
No	Winter	Male	No	No	Spontaneous	0.25665016
No	Spring	Male	No	No	Spontaneous	1.136011
No	Spring	Female	No	TplAdmitted	Assisted	-0.1696818
Yes	Spring	Male	No	TplAdmitted	Spontaneous	-0.8162243
No	Summer	Female	No	No	Spontaneous	-1.1086081
No	Summer	Female	No	No	Spontaneous	-0.9449207
No	Spring	Male	Yes	No	Spontaneous	-1.1790714
No	Spring	Male	No	No	Spontaneous	1.22096401
No	Autumn	Male	No	No	Spontaneous	0.3805241
No	Spring	Male	No	No	Spontaneous	-0.7199382
No	Summer	Male	Yes	No	Spontaneous	-0.039124
No	Spring	Male	Yes	No	Spontaneous	-1.7726341
No	Summer	Male	No	No	Assisted	-0.4478214
No	Winter	Male	No	No	Spontaneous	-0.7856591
No	Summer	Male	No	No	Spontaneous	-0.3650525
No	Autumn	Male	No	No	Spontaneous	0.0440779
Yes	Summer	Female	No	No	Spontaneous	-0.1749647
Yes	Autumn	Male	No	No	Spontaneous	0.45423108
No	Summer	Female	Yes	TplAdmitted	Spontaneous	0.92023061
No	Winter	Male	Yes	No	Spontaneous	0.16804394
No	Summer	Male	No	No	Spontaneous	-0.0371249
No	Summer	Male	No	No	Spontaneous	0.52736679
No	Winter	Male	No	No	Spontaneous	0.46111662
No	Spring	Female	No	No	Spontaneous	-1.4570292
No	Autumn	Female	No	No	Spontaneous	-1.6859626
No	Winter	Female	No	No	Spontaneous	0.36866266
No	Summer	Male	No	No	Spontaneous	-0.2562649
No	Spring	Male	No	No	Spontaneous	-0.6384519
No	Summer	Female	No	No	Spontaneous	-0.0711748
No	Spring	Female	No	No	Spontaneous	1.50014236
No	Spring	Male	No	No	Spontaneous	0.844454
No	Autumn	Female	Yes	No	Spontaneous	-0.41251
No	Spring	Male	No	No	Spontaneous	0.18989877
No	Winter	Female	No	No	Spontaneous	0.17146907
No	Autumn	Female	No	No	Spontaneous	-0.553204
No	Spring	Male	No	No	Spontaneous	0.36899289
No	Summer	Male	No	No	Spontaneous	1.0657011
No	Autumn	Female	Yes	No	Spontaneous	-1.40848
No	Spring	Male	No	No	Spontaneous	-0.2326366
No	Summer	Male	No	No	Spontaneous	-0.0797123
No	Autumn	Male	No	No	Spontaneous	0.77344487
No	Spring	Male	No	No	Spontaneous	0.06283035
No	Autumn	Female	No	No	Spontaneous	0.06197753
No	Summer	Female	No	No	Spontaneous	0.54333998

No	Spring	Female	No	No	Spontaneous	1.85104715
No	Summer	Male	Yes	No	Spontaneous	-1.1985889
No	Summer	Male	No	No	Spontaneous	0.7722448
Yes	Autumn	Female	Yes	No	Spontaneous	-0.8694928
No	Summer	Male	No	TplAdmitted	Spontaneous	-1.4661419
No	Autumn	Male	No	No	Spontaneous	-0.1705694
No	Winter	Male	Yes	TplAdmitted	Spontaneous	-1.9596534
No	Summer	Female	No	No	Spontaneous	0.8428145
No	Autumn	Male	No	No	Spontaneous	1.52708555
No	Autumn	Male	No	No	Spontaneous	0.36436942
No	Winter	Male	No	No	Spontaneous	0.89220089
No	Autumn	Male	No	No	Spontaneous	0.78885171
No	Winter	Female	No	No	Spontaneous	0.11667372
No	Summer	Female	No	No	Spontaneous	0.94539509
No	Winter	Male	No	No	Spontaneous	1.54844496

zScoreHC	zScoreLength
0.038788	0.95492425
1.37389214	-1.1424779
1.32191169	-0.8180647
-1.0424122	-1.5518396
-0.9663603	-1.182269
0.01693919	0.60088094
0.89030553	-0.0594662
2.29379386	1.4033977
0.5725637	1.24169284
-0.2534566	0.70443637
0.8487697	-0.7298095
0.52831734	0.15711126
-0.9663603	-2.1211908
1.87550905	-0.2643728
-0.505987	0.41982113
-1.1809221	1.96046294
-0.6786141	-0.2667789
0.83925158	1.56990027
-0.0723853	-0.7476692
0.5655585	0.01218233
1.9024738	1.41418833
-1.9643377	0.36567777
-0.5521115	0.32518325
0.15172909	-0.0894362
-1.1081481	-0.9360939
-0.1319501	-1.0610051
0.20928723	0.02892968
0.20928723	-0.382667
0.62379783	-0.7689199
-0.1434823	-0.922539
-0.5685	-1.2919939
-0.068631	0.23418766
-0.246458	-0.1272737
-0.5978249	2.02125283
0.64957521	-0.2911539
-0.1259538	-0.7834769
-1.1048331	0.05319939
-0.1796384	0.01218233
-0.1809283	-0.0389863
-0.3965653	0.12063913
0.52191605	1.32364414
-0.290613	-0.4836124
1.13781685	1.00908506
0.18410399	2.1417182
-0.2900763	-1.0748043
-1.7485856	-0.3114576
-1.3577886	0.39326568

0.96800713	-0.7003143
-0.1737567	-0.2344106
-0.9669612	-0.1584219
-1.1809221	-1.5631606
-0.505987	-1.8895915
0.5725637	-1.1151912
0.83925158	-0.8499488
-0.7722165	-1.1091106
-1.2414262	-0.7698277
-0.9511727	0.80230474
-0.7674116	-0.7298095
-0.1670504	-0.4286167
0.89686214	-1.1860111
0.23448637	0.80774628
0.5725637	2.69647837
-0.9948031	1.60788433
0.81179927	-0.6889345
1.8585796	-0.4728665
1.49166496	2.11172456
0.69142682	0.13847156
-1.2097715	-1.7230723
0.90344377	-0.773303
0.44628173	0.22534009
0.01049711	1.80602107
0.89030553	-1.2644925
-1.8159991	-1.6159283
0.88541224	-1.0223938
-1.0410116	-1.7525072
-0.8747998	1.22594638
-0.1094397	0.79667044
-0.5506074	0.15711126
0.86818085	1.42498675
-1.2476068	-0.291069
-0.6339	-0.3135505
-1.5944332	-0.8180647
0.89026426	-0.0263606
1.21843474	1.10761951
-0.3369778	0.30616383
0.18841783	-0.8253817
-0.2494369	0.47001607
1.37969927	0.76393801
0.25111415	0.48977803
-0.8778808	-0.859717
1.35345109	0.61929826
0.62001531	0.26531803
-0.9011276	-0.409705
1.15843691	0.09122931
0.70222011	-0.4302468

0.64965594	1.31002642
0.48131447	-1.7237429
0.44565998	0.12880463
0.43901504	0.10629191
0.39452736	0.59757126
-0.3999938	0.47001607
0.5840431	-0.1146244
-1.5009114	-1.2024379
0.9161714	0.05319939
1.02507639	0.98445706
0.41997157	-1.3034072
0.5572961	0.44204027
2.62995309	0.82962549
-1.5589465	-0.1328186
0.43494466	-1.1436946
-0.9532793	-0.5775612
-0.4348674	-1.8900963
-1.337725	-0.6537669
1.02507639	0.60088094
2.55152225	1.61563092
-0.1754157	0.98135475
0.62768078	0.36567777
-0.3886968	-0.0821906
-0.9011276	0.4340106
-0.5440282	0.01218233
0.8697978	0.14946437
-1.4903514	-0.7476692
-1.4100417	0.3908827
0.96800713	0.87979763
-0.9970883	-2.0083055
-2.5328766	-2.5597502
0.43494466	-1.1436946
-1.165386	-0.3801578
0.54878258	0.0118658
-0.4793499	1.96783442
-0.9396209	0.22534009
1.24805831	1.96783442
-1.421437	0.85258115
0.89689791	-0.0377219
-1.1617036	-2.0060044
0.45867667	0.67135039
-0.7859525	-1.4688044
1.48811043	1.05591992
-0.5685	-0.9095103
0.77063498	0.68734011
0.16518134	-0.4821408
0.09485513	0.63429952
-1.0410116	0.43553719

0.09485513	1.05591992
-0.1608135	-0.8110236
0.05085189	1.07001946
-0.5355354	1.19640497
-0.1387933	0.84052696
-0.5355354	0.51517265
0.88740643	-0.7075379
0.7885523	2.27046126
-1.2550856	-0.1273969
0.54878258	0.0118658
-0.9011276	1.24083467
1.7290776	0.44204027
0.70222011	1.52845544
0.76760953	1.78509729
-0.3369778	0.09122931
0.60802349	-0.1401029
1.21124128	-1.0273927
0.06836209	0.5848518
0.25111415	1.81951128
2.03124264	-0.1482902
0.08008114	0.56476566
-0.3369778	0.09122931
-1.5982026	-1.3452131
-0.0608825	0.91211883
-1.7168994	-1.4688044
0.5732886	-2.3917122
-0.4950528	0.0118658
-1.3591125	-0.4316229
-0.4335438	-1.4449619
2.13561749	0.98850748
0.25111415	-0.7111214
2.18802495	1.44195813
1.27372994	0.04815896
0.05915981	0.42653372
-0.4713359	-0.0339807
0.93533667	0.08405981
2.30028358	2.00716128
0.08858034	0.2504103
-0.5487076	-0.4727652
-0.3130828	-0.7302558
0.56776173	0.00203397
1.05001148	2.08858447
1.62941363	1.61779863
-0.0507339	0.84336282
0.10725523	0.2689193
0.43261617	0.39893654
-0.8601857	-0.8381369
-1.1308602	-0.773303

-1.2746898	-0.7408774
-0.5101652	0.36567777
0.48225762	1.97618894
0.15713245	-1.0165687
-0.6459379	-0.6537669
1.63397207	0.12063913
2.28104363	1.9813475
0.09485513	0.02533717
-1.0424122	1.18981448
-0.505987	-0.8110236
0.13918668	0.39376686
1.15843691	-0.9655296
-0.6366476	0.4630135
-1.7383075	-2.0060044
-1.1408262	-0.409569
0.90344377	0.44204027
-1.0701245	-0.3356293
-0.9011276	-0.8180647
-0.3167643	-0.9444389
-1.1921672	-0.8499488
-0.0507339	1.02534707
0.54878258	-0.197643
2.20736527	0.15827449
1.65315523	-0.0263606
0.0605903	-0.1417721
-1.5423218	-1.3739089
-1.7527107	-2.5436785
0.85572797	2.40497143
0.15045599	-0.0857186
-0.8840112	0.06726977
-1.2746898	0.73451682
0.7885523	0.52178099
-1.9637163	-0.4196301
0.81044548	0.72960926
-0.3954458	0.43553719
-0.5945228	-0.4448985
-0.6231517	1.09405138
-1.6052471	0.5280105
0.18841783	-0.4286167
0.78436166	0.17265613
-0.1930146	0.70980325
-0.1208471	1.36759433
-0.7859525	0.46269763
-1.5077887	-1.7908899
-1.5341996	-0.8557615
-0.5487076	-0.8381369
-0.6111145	-1.1644513
0.34842748	-0.6546384

-0.1531583	-0.0440311
-2.1282007	0.19335206
-1.1617036	-0.4727652
-0.7859525	-0.9324737
1.70423466	1.24083467
-0.8292437	-1.9574428
1.59895673	2.27151596
-0.712078	0.13142109
1.14837634	0.99924723
0.35474061	0.20717412
0.89030553	0.78359104
0.41533062	-0.3356293
0.44007912	0.56796124
0.35026717	0.55770894
-0.6846108	-0.0975072
1.59895673	0.31712126
-0.7859525	-1.1151912
0.53741295	1.5792972
1.50287569	-0.2504605
-1.0735147	0.08405981
0.56776173	-1.1091106
-0.276374	-0.6006613
0.76760953	2.16135025
1.93505038	0.84052696
-0.5887542	-0.5378222
0.5840431	0.76328026
0.13283868	-0.1156069
-0.8278458	-0.9833518
0.43901504	-0.0901263
0.38414514	2.07688102
0.20928723	1.22594638
-0.1809283	0.34861758
0.31895787	-0.1401312
0.43261617	-0.6006613
-1.3909114	-1.3034072
-0.8412465	-0.6163749
-0.2131317	-0.5084063
-1.2110993	-1.0521584
1.72774346	0.99599505
0.65413925	-0.6264753
-0.4348674	0.00230302
0.78436166	0.17265613
0.65413925	0.16206778
-0.2971853	-0.7075379
1.59116962	0.82962549
-1.1048331	-1.1151912
-0.8907537	0.00756715
0.10489827	-1.8785549

-0.0100249	-0.2189276
0.42844505	0.17265613
-1.0374314	0.12063913
-1.2996258	-0.1482902
0.43261617	2.36014471
0.35474061	0.60088094
1.12599823	0.08178215
1.1312545	2.08354829
-0.021896	-0.6087847
1.18026279	0.303064
0.13607993	-0.8932855
0.19215338	1.59114168
0.35132443	-0.2282261
-0.1737567	-0.4420973
-0.9948031	-0.6537669
-1.0089501	-0.0692257
1.07620876	2.18361021
-0.6391949	0.58597487
1.27016084	2.52666576
-0.2226933	-1.0165687
-0.021896	0.47001607
2.42919788	0.80230474
1.13776231	-0.0389863
0.84990722	-0.1482902
0.8984733	0.81920561
1.47504977	0.59158127
1.99231217	1.73792144
0.0605903	0.64391046
-0.4912225	-0.7834769
-1.2961857	-0.4927179
1.69817112	-0.5487843
0.44620623	0.99097883
0.10489827	0.67767417
-0.1187872	-0.1537058
0.19215338	-0.8110236
-0.6391949	-0.2320198
-0.276374	-0.2060553
-1.8131246	-0.8253817
-0.276374	-0.2060553
-0.1531583	0.75266956
-0.243168	-0.2503609
0.95871921	1.19887624
-0.3886968	-0.7111976
0.64965594	1.31002642
-0.6669228	0.6356025
0.13283868	1.13578809
1.83920159	1.44195813
1.02507639	0.60088094

-3.3512867	-0.7209288
0.51327876	-0.4448985
-0.1187872	-0.5533592
-0.3965653	1.29137582
-0.0929535	2.14696968
1.57112751	0.03386294
0.44565998	-1.321358
-1.8321742	-1.58236
-0.811544	-1.3598024
0.35026717	2.12617767
0.44007912	1.86875737
-0.6465241	-1.0600865
2.05176624	1.02438077
1.25824394	1.05137504
-1.0254496	-0.9154749
-0.2277352	-0.1328186
1.48927014	0.58597487
0.94242859	0.01218233
0.0705913	-1.0520094
0.16518134	2.30658962
1.32555658	0.16206778
0.07763102	-0.2770455
-0.4668689	-0.0372376
1.20783258	0.75266956
-0.6786141	-0.2667789
-0.5355354	-1.240193
-0.7377751	0.71495284
-0.5059903	0.85292665
0.35802637	0.03553497
-0.276374	-0.404039
0.74334595	0.59158127
-1.5516836	-1.8809973
-1.8131246	-3.0421646
-0.1737567	0.81274124
2.96466255	0.85832333
0.53013696	0.34649275
-0.5440282	-0.8180647
0.0705913	-0.4527412
-1.6808132	-0.6504182
1.59598956	-0.4163746
-0.5521115	-0.5487843
-0.8071511	0.43838944
1.02507639	1.17295273
2.44000484	-0.6034509
-0.7564827	-0.7111214
1.9436645	0.67135039
-0.0497205	0.9727914
0.06070744	1.40797245

-1.5570646	-0.4821408
0.79962128	0.19335206
-0.1208471	-0.9746556
0.15045599	-0.0857186
-0.7473984	-0.7111976
-0.2277352	-1.6428261
-0.635864	0.14744432
0.48117058	1.83606209
-1.7168994	0.6622042
0.45867667	-0.8059708
-0.3965653	-1.5618464
0.18841783	0.81920561
0.85477234	0.29294206
0.18841783	1.21064496
-0.2876162	-0.4488923
0.79173808	0.99924723
0.08498416	0.42796619
-1.001532	-0.6720844
0.73026772	1.02438077
0.89030553	-0.0594662
0.70249864	-0.1482902
0.05915981	1.24041202
-2.3727728	-1.3402155
0.35978784	1.03977118
0.07774251	0.19489341
0.05915981	-0.6385243
1.17370791	-0.7298095
0.60802349	1.26558511
-0.9295819	-0.6501084
-0.6366476	-0.183187
-1.5589465	-1.2750983
-0.0723853	-0.5432669
0.02947441	0.55001384
-0.6963067	-1.514779
-0.8205868	0.34649275
0.19122282	0.4340106
-0.4901333	-0.0440311
0.89689791	0.80104075
-0.6459379	-2.213915
1.72234476	0.25435374
2.25750965	-0.8937341
2.34156959	2.10867242
0.44007912	0.14852485
0.20928723	-0.382667
-1.5516554	-0.5775612
-1.1423179	-0.5487843
-0.9396209	0.22534009
0.99058951	-0.0402278

1.60324924	0.20338551
-1.7226032	-0.7075379
-1.6893755	0.14852485
-0.649019	0.99452811
-2.4943791	0.37037346
1.22569792	1.45616603
1.59116962	0.22188836
-1.2300955	-0.8828678
-0.0459575	-0.7561043
0.39636217	0.64391046
-0.2900763	0.35735903
-0.3586532	-0.2404029
0.01693919	0.00689131
1.373159	0.59154621
0.6215712	0.91211883
0.32453359	0.43838944
0.67391379	0.51667761
0.88003021	0.13147781
-0.4296591	0.48977803
-1.1061743	0.12508095
0.96033984	0.91211883
0.19215338	9.54E-05
1.06218213	1.02438077
0.44628173	0.63429952
-0.3167643	-0.1923799
-1.0424122	-0.7302558
0.25111415	0.08405981
0.42813688	0.60416136
1.89444265	1.78509729
-1.2514348	-1.2919939
-0.7967468	-0.3347809
0.89030553	1.374653
1.87550905	0.99452811
-0.2277352	0.26204686
-0.8278458	-1.1676057
1.92247392	1.10566829
1.13567944	-0.9965677
-0.290613	-0.6889345
0.5725637	-0.3561993
0.31836881	0.41784479
-0.7087782	-0.270336
-0.2131317	0.76115322
0.72414223	-0.9123921
0.42844505	-0.4321041
-0.0218542	-1.1098099
0.01693919	0.60088094
-0.4296591	-0.7111214
0.01049711	0.06536491

-0.4713359	-0.0339807
-0.2966855	0.46411979
0.48225762	-0.2035881
-1.0735147	-2.0983937
-2.248909	-0.6010295
1.59598956	9.54E-05
1.23267315	0.31712126
0.44620623	-0.0692257
0.84251625	0.68145791
0.94242859	0.84326187
0.24558057	0.68162289
-1.1048331	-0.7453292
1.78130226	-0.4674776
-0.6809289	-0.7302558
-0.3965653	-0.6712958
-0.2782413	0.03386294
-1.0215808	-1.1180398
0.95871921	-0.1146244
1.25500288	-0.6440838
-0.9866879	-0.0932142
-0.3667014	-1.4108983
-0.5978249	-0.3766351
-0.290613	0.13990795
0.15713245	-1.4317117
-0.6529911	0.13990795
1.13781685	0.58597487
-0.5324816	0.303064
-0.649019	-0.058633
1.95499219	1.21064496
0.05572629	-0.2498007
-0.3369778	-0.3356293
0.99661549	0.0522141
-1.4869041	-2.2297857
-0.2722539	-0.8921085
-1.4903514	2.46047801
0.79962128	-0.8937341
-0.4651752	1.46798092
0.19285366	0.01063941
-0.4912225	1.08483807
-0.2354529	-0.741956
0.54428837	-0.0097915
0.24558057	-0.372826
-1.1733831	-1.2742958
1.59598956	-0.4163746
-0.6245999	0.19489341
-0.1522862	1.21708103
0.07408975	-1.1180398
0.49992568	0.93697316

-1.1308602	-0.382667
-0.1522862	0.0118658
1.37389214	0.58462738
0.81179927	-1.0958111
1.07620876	-0.4302468
0.52831734	1.1809304
0.24901842	-0.0730457
0.6215712	0.52244291
2.55812574	1.21475555
-0.8412465	0.41982113
0.53214514	0.04646859
1.29760307	0.32305437
-1.1948495	-0.0213207
1.78896398	1.83606209
-1.1948495	-1.219652
-0.2277352	0.6696685
-0.0744285	-0.2504605
-1.3590106	-0.6889345
-0.6809289	-0.3114576
1.04140574	1.71149428
-0.8278458	-1.1676057
-0.6465241	-1.8785549
-1.1140142	-1.4677421
0.39636217	-0.5258678
1.80851463	0.51667761
-0.6625825	1.63877135
-1.7485856	0.11255571
-0.5146739	-0.6296872
0.18841783	-0.8253817
-1.421437	-1.9175014
0.20928723	0.02892968
0.44628173	-0.1723356
0.17654456	0.39005349
-0.1809283	-0.7837571
-0.2265149	-0.8381369
-1.4903514	-3.3239327
0.17654456	-0.2514898
0.53013696	-0.6118496
0.62379783	-0.270336
-0.0774171	1.39572265
-0.4348674	-0.4302468
-1.0980098	0.76388353
-0.3667014	0.28145159
-0.4713359	0.40350948
1.90191171	0.54075228
-0.6167111	-1.2943644
0.43031476	-0.2198048
-0.3965653	0.12063913

0.67391379	-0.3347809
0.86649444	0.10188752
-0.6809289	-0.7302558
0.82906509	1.46822202
-1.2110993	-0.2514898
1.46425329	0.10047938
-0.6542332	-0.9122976
-0.4572895	-1.4114002
-0.9055271	-0.1328186
-1.5173764	-0.0213207
0.3855544	0.40733788
1.82029906	1.14027294
-0.1259538	-0.9834056
-1.5461325	-1.2994397
0.79173808	-0.2504605
0.19122282	0.84326187
-0.8778808	-0.0377219
0.04925799	0.08982171
-0.8098652	0.02892968
-0.5487076	-0.0901263
-2.3919156	9.54E-05
-1.3591125	0.6356025
0.54253891	-0.0213207
-1.2350384	0.26204686
1.18026279	0.303064
-0.9011276	-0.8180647
-0.9396209	-0.1723356
0.02268031	0.17434787
0.24901842	-0.9327045
0.19122282	-0.409705
0.95871921	0.76328026
0.82906509	0.2504103
0.48955547	1.913416
-1.3591125	-0.6385243
0.66682362	0.62202779
0.63764139	0.6752495
-0.9970883	0.16206778
0.88003021	1.16046722
-0.3369778	-0.3356293
0.53013696	2.07224079
0.83925158	1.79457943
0.19818366	-0.9833518
-0.6391949	-0.4321041
-0.1259538	1.28219565
0.52831734	-0.4821408
-0.215206	0.6526441
0.41533062	1.3906595
0.31572285	-1.515572

0.9161714	-0.1537058
1.51505214	0.38085652
0.56776173	1.00632221
1.42810951	1.70992378
-0.5506074	-1.2644925
0.51327876	1.58762745
-2.1181491	-1.5631606
0.797869	0.37565387
-0.0400793	-1.6046225
-0.276374	1.02778607
2.2535277	0.95492425
1.05516113	0.10870804
-0.5146739	-0.8253817
1.1426752	-0.5629424
-0.1387933	0.84052696
-0.7722165	-0.3751877
-0.384913	-1.219652
0.81179927	0.99097883
1.89317901	1.42529779
0.28085833	0.91211883
0.44565998	0.55435334
-0.3965653	0.12063913
-1.7375838	-0.4851088
0.90344377	-0.1792637
-1.9312625	0.22453183
1.97291223	0.78359104
0.48955547	-0.0969208
0.28085833	0.91211883
1.01879109	0.52307267
-0.5616111	0.28851095
0.78518601	-0.6006613
0.038788	0.09122931
0.78436166	-1.2193353
1.13781685	1.22431363
-1.2110993	-0.6624155
-2.0437595	0.28233215
-1.2097715	-0.2516417
1.53387608	0.63403674
0.62001531	1.66975067
0.17654456	1.95231377
-1.0424122	0.97256281
-0.5146739	1.21064496
-1.2097715	-0.8932855
0.44007912	1.21047487
1.85573066	2.32293997
1.06218213	-0.1417721
0.13283868	-1.6904288
0.9161714	1.24169284

1.95633972	0.89599721
-1.9726773	-2.2891837
0.42844505	1.00908506
-0.1670504	-0.6296872
-0.6963067	-0.2488129
0.65413925	-0.2404029
-0.8435226	-0.4674776
0.79554294	-0.5629424
1.24371108	-0.2998042
-1.2216387	-0.915146
-0.2733651	-0.2035881
1.10403746	1.65127738
-0.1323822	-0.5070643
0.43248206	-1.0520094
-0.7027801	-0.0406011
0.19215338	0.82366063
0.43261617	-0.6006613
-0.9055271	-1.6428261
-0.1636492	-0.377777
1.18364895	-1.240193
0.02268031	1.78509729
0.74334595	0.59158127
0.89030553	0.37037346
0.04391124	0.19492789
0.90344377	0.84052696
-0.7473984	-0.0821906
0.41997157	0.72389506
-0.1967841	-0.3183735
1.08050309	-0.0294367
0.18410399	0.81274124
0.9161714	-1.1151912
1.06627163	1.92961367
-0.6786141	-0.6245138
1.12599823	1.913416
-0.1737567	-0.4420973
0.25111415	-0.7111214
-0.894859	-0.0594662
0.56864472	-0.0647997
1.17373239	0.11255571
-0.4225656	1.0289297
-0.5146739	-0.2218621
-0.7719092	-0.180771
0.29960747	0.92607838
0.9161714	-0.7453292
-0.0152612	0.88489616
0.99058951	-0.6264753
-0.8071511	0.00230302
-0.4793499	-0.773303

0.07475883	0.35735903
-1.3305843	-1.4317117
-0.8205868	0.75266956
0.10808694	-0.1829504
1.93505038	0.02892968
-0.7967468	-0.7476692
-2.0114766	-1.4688044
-0.3487069	-0.4851088
0.44007912	-0.6720844
0.72414223	0.55770894
0.41533062	0.09122931
0.88541224	0.43577915
-0.7722165	-1.1091106
0.90344377	-0.5806112
0.10725523	-0.4266101
1.37552494	-0.7834769
-0.7473984	-0.7111976
-2.5237603	-2.7293145
-0.2559851	0.22534009
1.54234896	-0.7075379
0.2054632	0.32518325
1.42810951	1.90018099
-0.6809289	-0.3114576
-1.0215776	-0.6766327
1.50438906	1.15003415
0.6215712	-0.2843507
-0.3072798	-0.0097915
0.67391379	0.30663684
-1.3547128	-0.6934105
0.34809913	0.03315215
-0.7219809	2.027074
-0.7859525	-2.4543859
0.2054632	0.32518325
-0.021896	-0.3956022
0.38414514	1.04333141
2.76515754	2.19711315
0.19818366	0.82962549
-0.7473984	-0.2961761
-0.2265149	-0.0901263
-0.2971853	-1.321358
1.25126401	-0.0097915
0.17654456	2.31741288
0.82906509	0.2504103
-0.021896	0.47001607
0.5610621	0.05839023
-2.5014023	-0.9005462
-0.1259538	-1.7537319
-1.9738182	-0.7111214

0.36180633	0.19489341
0.73373291	-0.0823363
0.51567221	1.92307514
0.53741295	0.17795148
0.79962128	-0.4666435
0.36269541	1.79914784
0.5725637	0.46269763
0.21074315	0.68145791
0.23448637	0.43577915
1.17447958	1.42529779
-0.656578	-0.0690913
-0.8907537	-0.1726111
-0.6010479	-0.5629424
-1.8777263	-0.9005462
-0.894859	-1.9831424
0.43901504	-1.6873303
-0.4296591	2.00137801
0.56776173	-0.3751877
0.16518134	0.37037346
1.13589156	1.06096989
-0.505987	9.54E-05
-2.5113261	-2.0218784
1.0024855	-1.760727
0.95306573	-0.3980196
-0.9511727	1.3946894
0.94153864	0.43553719
1.13545803	1.46103101
0.70222011	1.31039399
-0.5025453	-0.4196301
0.5725637	0.05319939
-0.0218542	0.13403404
0.95306573	0.87659022
0.81410049	1.2015002
0.24558057	0.47520315
0.16518134	-0.0594662
-0.9360877	-0.9389847
0.35802637	0.47001607
-0.1737567	-0.0213207
0.44628173	0.63429952
-1.7763597	-0.0440311
1.13545803	2.36014471
0.11258922	1.10761951
0.81404061	1.97704166
-1.0089501	0.13990795
-1.5991089	-2.0644174
1.2146732	-1.6346834
-0.1094397	0.3908827
-0.7473984	1.36270467

0.05915981	0.21331762
0.12585425	-0.1402319
0.01693919	1.35958306
-0.6963067	0.19335206
0.51327876	-0.4448985
0.88003021	1.54688739
0.08858034	-0.6033754
-0.8470735	-0.2320198
-0.7214073	-0.6934105
1.14837634	0.15827449
0.44007912	0.78035223
0.44565998	-0.5003595
1.07620876	1.09240927
-0.4574295	-0.5533592
-1.1081481	-0.2927725
0.95306573	1.23566847
-0.5355354	1.5792972
0.07006751	-0.630892
0.20928723	-0.382667
-0.2265149	-1.0148045
0.41533062	1.82912688
0.93533667	2.00137801
1.57279893	1.55197529
-1.5944332	1.24083467
-0.9844121	0.17265613
0.45133529	-0.2404029
0.08858034	-1.0040254
-0.2265149	-0.0901263
-0.7940497	-0.3505393
-1.0215808	-0.2919015
0.7885523	0.95492425
-0.5101652	1.23878724
0.5655585	1.04337826
0.08858034	-0.6033754
-1.6417323	0.52244291
1.92342911	0.6356025
-0.2309168	-0.8509563
-0.2277352	-0.9005462
0.61524158	0.34698154
-0.2722539	-0.1417721
0.67391379	-0.3347809
2.26256826	0.55435334
-1.1531328	-0.4950336
1.57112751	1.46822202
-0.4574295	-0.3561993
1.37552494	0.88474317
-0.9295819	0.56468011
-0.3369778	0.09122931

-0.5521115	-0.7624401
0.07774251	-1.182269
0.11258922	0.2851563
-0.6245999	-0.6006613
-0.1796384	1.62876278
-0.9844121	0.58597487
-1.4431533	-2.1701223
0.81179927	0.13990795
0.44565998	-0.2919015
-1.8159991	0.93228883
-0.9011276	1.24083467
-1.1512121	-0.6010295
0.36269541	0.72275019
-0.3167643	-0.1923799
0.09485513	1.05591992
1.25824394	0.05319939
0.20928723	1.60097796
0.15045599	0.34579098
-0.5887542	-0.1156069
0.35026717	0.13403404
1.51979766	0.14852485
-1.7728359	0.82962549
-1.0672779	0.39326568
-0.8638924	-2.2691049
-1.001532	-0.8740769
-0.9532793	-0.1923799
0.5655585	1.62876278
-1.7354807	-1.6382092
0.80053085	0.68961296
0.52831734	-0.0594662
0.08858034	1.07633145
-0.669224	-0.999602
-0.0608825	-1.3912073
-0.3369778	1.82912688
-3.2656761	-2.6793379
-1.1447816	-0.0440311
0.06070744	0.54075228
1.25824394	0.46269763
-0.2906357	2.32293997
0.89689791	1.19640497
0.03281802	1.35292168
-0.6167111	-0.7698277
0.82906509	-1.0040254
0.99058951	-0.2404029
0.53526885	0.72118425
-1.6052471	-1.3221267
1.2448234	0.22188836
0.07408975	2.30338977

0.49992568	-0.1156069
0.38894882	0.06536491
0.53741295	1.00047135
0.07763102	0.99097883
0.35802637	-0.3956022
0.07774251	-0.2060553
-0.5146739	1.21064496
0.52831734	1.1809304
0.52831734	1.1809304
0.72414223	0.96655496
-1.3591125	1.04218262
0.4492204	-0.6702245
-0.0029101	0.14982398
-0.2920648	-1.260223
0.19122282	-0.409705
1.06602681	0.99184288
2.19580162	1.24435223
-0.3453793	-0.999602
-0.6809289	-0.7302558
0.21074315	-0.4026838
0.8984733	0.81920561
-1.2300955	0.78359104
-1.2097715	-0.4674776
-1.4632151	-1.5244415
0.6215712	-0.2843507
0.22706281	-0.3561993
1.1002717	0.30201136
-0.8601857	0.68734011
0.23448637	0.05839023
0.2054632	-1.3904143
1.19974175	0.67135039
0.18410399	0.61050364
-0.1754157	0.76328026
0.17654456	-0.859717
-0.7035122	-1.4587858
-0.4574295	1.42958771
-0.5237183	0.40405277
0.8994119	-0.2344106
0.86688128	1.53416501
-0.1967636	-0.685122
1.23943969	0.3908827
-0.1796384	-1.5985241
-1.0170942	-2.5067305
0.08858034	1.07633145
-0.5506074	-0.4821408
0.99058951	0.75449289
-1.1352108	0.22917504
0.41533062	1.82912688

-0.3254157	-1.1032035
-1.853149	1.19640497
-0.2782413	-1.3881465
0.44565998	-1.1180398
0.78520938	1.27253034
0.15045599	0.34579098
-1.421437	-1.514779
0.17654456	-0.859717
-0.8205868	-0.0440311
-2.1030516	-1.4994675
0.81179927	0.99097883
1.15831387	1.43225961
-0.5146739	-1.5631606
0.32453359	1.31039399
0.5655585	-0.6156071
0.84251625	0.68145791
-0.7982412	-0.9389847
0.72414223	0.13403404
-0.2559851	-1.3266674
0.74047748	0.55434376
-0.6963067	1.07001946
0.5725637	-0.3561993
2.12267398	0.56060223
1.47640912	2.12617767
-0.8601857	-0.2835999
0.79173808	0.36536826
1.24019914	1.74518682
-0.6402031	-0.9444389
-0.6111145	0.70443637
0.53526885	-1.0165687
-1.1733831	-2.4131291
-0.0152612	0.0938184
1.24805831	-0.382667
1.58570687	1.07197483
-0.2900763	0.78035223
-0.8098652	-0.382667
0.43248206	0.99924723
0.22931666	1.92753158
0.22949629	1.2205165
2.31773452	1.46822202
2.84181286	-0.5916353
0.89030553	0.78359104
0.7885523	0.52178099
-0.7967468	0.09307518
-0.6835278	0.40465377
0.06070744	0.54075228
-1.0254496	0.30394875
1.47758608	-0.3956022

0.35802637	0.03553497
0.64965594	2.22046981
0.6466253	0.31712126
0.89689791	-0.4597904
0.72414223	1.74859731
-0.1323822	0.80309635
0.48955547	1.46798092
0.80221884	-0.2198048
-0.5506074	-0.8828678
2.19151076	0.17888289
0.8984733	0.81920561
-0.1094397	0.59154621
0.39452736	0.59757126
-0.3768572	0.14982398
-1.0980098	-1.1098099
1.95202846	0.8824571
-0.5141319	-0.4949935
1.42851511	0.30201136
0.18410399	-0.4420973
-0.5146739	0.41288882
0.2693758	0.95259429
1.07620876	0.65645042
1.94015599	-0.7453292
0.5655585	1.62876278
-0.0608825	1.29137582
-0.4296591	0.48977803
-0.6366476	-0.6033754
-0.2534566	0.70443637
0.73373291	1.66019158
-0.5101652	-1.3503454
3.19826835	1.89038566
-0.7219809	-0.6712958
-0.6963067	-1.1032035
-1.1026097	1.80602107
1.21843474	1.10761951
-0.8205868	-0.6118496
2.2905103	1.2637708
-0.3130828	0.11255571
1.59895673	0.31712126
0.79554294	0.63429952
-0.6366476	-0.6033754
-0.8435226	-0.8932855
-0.1967636	0.78359104
0.25111415	0.8824571
0.44565998	0.98461405
1.60989289	1.20614028
-0.1725016	-0.1475429
0.7569964	-0.8253817

2.59660215	1.70936166
0.05915981	0.21331762
-0.3080782	-0.2198048
-0.7397295	-0.8848309
-0.9072323	-0.1417721
-0.2786676	2.1149868
-1.6752756	-1.8323464
-0.9596509	-0.5696545
-0.0152612	0.47981949
0.28811855	0.52148801
0.62768078	0.80309635
-0.6809289	-1.7539506
-0.6809289	-0.3114576
0.2054632	0.76328026
-0.2554363	-0.3561993
-1.4148955	-1.4688044
-1.9867396	0.67135039
1.60989289	-1.219652
-1.7329456	-0.270336
0.21074315	1.7215089
0.51327876	1.3946894
-0.3667014	-1.2044565
-1.0170942	-1.0386542
-1.4845096	-1.8895915
-0.669224	-0.6419848
-1.0170942	1.81886009
1.32555658	0.94649803
0.01207562	-0.933614
0.85572797	1.1133979
-1.3757438	-1.0600865
-1.3112426	-1.9875317
2.05853165	1.75627922
1.22551391	-0.2035881
1.25824394	1.05137504
0.5655585	0.4340106
0.8994119	-0.8407003
-0.2875304	-1.4127088
-0.3130828	-1.5518396
0.48117058	1.10357332
-1.1948495	-1.219652
0.82906509	-0.395846
-0.1834889	-0.4597904
0.24901842	0.36567777
1.83229799	1.93691238
-1.1302408	-1.1871294
1.60324924	1.40175052
-1.3305843	-1.4317117
-0.8412465	-1.0007556

1.75114271	2.33993893
1.17373239	0.97256281
0.06070744	-0.7302558
0.38414514	0.14321022
1.22551391	1.54592321
1.70423466	2.0087867
-2.0079534	-2.213915
-0.3808967	-0.7423
-0.4385176	-0.1222227
-0.4572895	-0.7298095
-0.1930146	0.303064
-0.5237183	0.40405277
0.84990722	-0.3616215
-1.337725	-0.0468728
-0.2722539	-0.1417721
0.05354183	1.16341515
-0.136327	-0.367377
0.8994119	0.19320496
-0.8907537	-0.3499416
-1.3112426	-2.1394791
0.48117058	-0.0389863
-0.1809283	-0.4196301
-0.8488786	-1.5650355
-0.232118	1.33135731
-0.4385176	0.92607838
-0.0459575	-0.9478167
-1.6323981	-1.3732313
-0.1187872	-0.7453292
-0.1116668	0.49077001
0.5984627	1.74518682
1.47640912	0.34775957
-0.4574295	0.05319939
0.13607993	-0.2516417
1.13545803	0.6057364
1.59173754	1.03469566
0.11999626	0.87876049
0.62001531	-0.372826
0.46803822	-0.1328186
0.79962128	0.19335206
1.89677205	0.63403674
1.84199924	0.6696685
0.73462404	1.3412614
-0.3999938	1.12315033
-0.5945228	-0.0263606
-0.6167111	0.13847156
-0.1323822	-0.5070643
0.62768078	-0.0730457
1.13545803	0.6057364

-0.5237183	0.61050364
-0.9320678	-2.6031047
1.05001148	1.70992378
1.83364058	0.68162289
-2.5511063	-0.3956022
0.2489131	0.52785018
0.05915981	0.6356025
-0.0152612	-0.277854
0.28085833	-0.2843507
0.10489827	0.23796022
-0.706706	0.09122931
-0.4793499	-1.1445253
0.90948713	0.50218674
-0.0608825	-0.6712958
0.0705913	-0.6537669
1.21843474	-0.9706532
-0.2559851	-0.1723356
-0.3130828	0.11255571
-0.5685	1.16046722
0.10536038	0.30201136
0.80221884	1.81886009
1.47077889	1.87434071
-1.0540559	-0.6746298
-0.4574295	0.46269763
-0.290613	-0.6889345
0.90948713	0.28233215
-0.5521115	-0.9738587
-1.4409892	-0.2961761
-0.2309168	1.19963824
1.07620876	-0.4302468
-0.8524423	-0.0097915
-0.4574295	-0.5533592
-0.3130828	-0.3114576
0.85572797	0.67767417
-0.649019	0.14852485
-0.2226933	0.28233215
-0.2559851	-0.5629424
-0.7224803	-1.3551952
1.17373239	1.40797245
-1.337725	-1.0520094
0.88541224	0.43577915
1.48927014	-0.2320198
0.13283868	-0.1156069
0.8697978	0.34649275
0.18410399	-1.219652
0.11258922	1.49771417
2.08962416	1.62876278
-1.001532	-1.6691454

0.43901504	-0.4727652
0.07763102	-0.2770455
-1.001532	-1.2742416
0.43494466	-1.5518396
-0.1522862	0.42839427
0.76404894	2.02569609
-0.0337019	-0.5509747
-2.3793637	-1.4975746
1.17447958	0.13990795
2.25016209	1.86536964
0.59418561	0.48977803
0.35802637	0.03553497
0.20928723	-0.773303
1.82029906	0.76979716
-2.3346454	-1.8406885
-0.1967636	-0.0594662
-0.6829502	-0.0402278
-1.1472187	-0.3347809
-0.9320678	0.32841161
0.42844505	0.58597487
0.48117058	1.65444472
0.08858034	-0.6033754
-0.9543362	-0.8557615
-1.1352108	-1.1363129
-0.3768572	0.81041177
1.04140574	0.40350948
-0.5887542	-0.1156069
0.44628173	0.22534009
1.90774758	1.15433529
-0.6366476	-1.1979554
-0.1796384	0.01218233
-0.505987	-0.8110236
-0.5237183	1.20614028
1.28010819	-0.5923925
-0.9896323	-0.073395
-0.4197893	1.90051982
0.18867112	0.34649275
0.19215338	9.54E-05
-1.4659877	-0.2927725
-0.8601857	-0.0901263
-2.7024063	-2.2012628
0.22949629	-0.745498
0.73026772	2.66587712
-0.2733651	-1.0600865
1.01440251	0.93789475
0.31876314	-0.9856082
1.15808454	1.09140604
0.2489131	-1.0332233

-0.3586532	0.75449289
-0.1834889	0.80104075
-0.5141319	0.60493741
-1.5132304	-0.8499488
0.85401349	0.15122142
-1.7768433	-0.6815959
0.05085189	1.93139925
-0.276374	0.6057364
-0.6963067	0.19335206
1.78896398	0.54005946
-0.1834889	-0.0377219
-1.6606231	-1.0040254
-0.1522862	-0.6010295
1.96463844	1.95892592
0.18410399	1.01114995
0.18410399	-1.0324053
-0.7837194	-1.1814314
-0.1608135	-1.1860111
1.24019914	1.20638867
2.37607291	1.15678108
-0.6963067	1.28648686
-0.2226933	0.06179549
1.25824394	0.05319939
-0.6245999	1.02778607
-0.5685	-0.0857186
-0.5826233	-0.367377
0.95871921	2.2813864
1.20783258	0.75266956
-0.4348674	0.65645042
-1.1809221	-1.2024379
0.13918668	0.39376686
-0.505987	-0.8110236
-0.4797464	-0.0765186
-0.9669612	0.72118425
-0.7214073	-1.4072199
1.25599395	2.0778539
0.83925158	-0.0894362
1.47758608	-0.3956022
0.08858034	0.67135039
-0.5025453	0.72960926
-1.6893755	-0.058633
1.92342911	-0.2198048
-1.1991206	-3.3690246
-0.5978249	-0.1584219
-1.9540592	-1.2205186
0.43248206	-1.8323464
1.81525859	-0.4302468
-0.6366476	-0.183187

0.69090048	0.20717412
-0.0926365	0.8824571
-0.4574295	-0.7453292
1.11083911	0.59158127
0.07408975	0.55435334
-0.7214073	-0.6934105
-1.5944332	-0.409705
0.94242859	0.4340106
-0.3586532	0.94649803
-0.6111145	-0.1482902
-0.0723853	0.92607838
-0.4793499	1.60097796
0.14974839	1.10357332
1.60723944	1.16046722
-0.1387933	-0.773303
-1.5968657	-1.4927248
-0.0167114	-0.7978388
1.37760007	1.75765994
-0.3453793	-0.277854
-0.4668689	-0.2087013
0.2054632	0.32518325
1.06218213	1.02438077
0.19122282	0.64021925
-0.8638924	0.40405277
0.78436166	1.00908506
1.14837634	1.43233953
-0.276374	1.02778607
-0.8278458	-1.1676057
-0.1930146	0.303064
-1.1302408	0.22354985
-0.9072323	0.25435374
0.62001531	-0.9834056
1.72112272	1.56526867
0.5655585	0.01218233
-2.2152547	-0.6712958
-0.1259538	-0.7834769
0.48117058	-0.7837571
-0.7473984	-0.7111976
0.6890216	0.55162593
-0.9626032	-0.8828678
0.20636009	-0.6087847
0.86649444	0.31712126
1.59938284	0.85832333
0.10536038	-1.5238692
0.42844505	1.44195813
-0.3369778	0.73803553
1.07620876	0.22028114
0.69090048	0.20717412

0.67391379	0.30663684
1.24805831	1.03469566
0.43494466	-0.7302558
1.50438906	0.71495284
-0.3586532	-0.0402278
0.62001531	-1.1801199
0.11258922	-0.9706532
1.32555658	-0.2404029
0.41533062	-0.7569871
1.21302381	0.8815318
-0.4901333	0.75266956
0.5610621	-0.4983843
-0.7674116	-1.7338811
0.0705913	-0.0468728
-1.2300955	-0.4821408
0.98422975	-0.1726111
-0.8524423	-0.4286167
-2.1065509	-2.4463753
1.10763049	-0.3956022
0.66682362	1.49381481
-0.7612904	0.13572775
-0.5324816	-0.0894362
0.42239882	1.49999316
0.82906509	0.67135039
0.19215338	-0.8110236
-1.1376722	1.3412614
0.43248206	-0.3719805
0.29960747	0.09307518
0.42713802	1.07001946
0.5655585	1.04337826
0.18841783	-0.4286167
0.38894882	0.06536491
0.9202217	1.38662462
0.05915981	0.6356025
-2.4629422	-2.6056545
-1.7474897	0.02892968
-0.8778808	-0.4597904
-1.1376722	0.47001607
-0.2887527	-0.310855
0.7885523	0.95492425
-1.0170942	0.21331762
0.16518134	-0.4821408
-0.2920648	1.81068461
0.07763102	-0.4836124
1.85573066	-0.2504605
-0.1796384	1.24083467
1.13545803	1.24302234
-0.5997177	-0.3210824

1.05464011	0.94251202
-1.337725	0.15827449
1.53387608	-0.0283259
0.35026717	-0.2961761
0.41533062	1.82912688
0.20928723	-0.382667
-0.9844121	-1.4127088
-1.1733831	1.09240927
-0.1844142	-1.6323498
2.01289789	0.72118425
-0.9663603	0.19489341
-0.9844121	0.17265613
-0.1187872	2.69647837
0.15713245	-0.3766351
-1.0965086	-0.9221568
1.49734909	0.01063941
0.84990722	-0.5696545
1.60989289	1.20614028
1.15808454	0.26204686
-0.3759017	0.31712126
-1.0701245	-1.5828455
0.49645062	0.70980325
0.88003021	1.54688739
0.46803822	0.6696685
0.63399072	0.14744432
0.73026772	0.25435374
-0.2559851	0.63429952
-0.0608825	0.12063913
0.43494466	-0.3114576
-0.5146739	-1.5631606
-0.505987	-0.8110236
1.53887445	-1.7539506
-0.9396209	-1.3266674
-0.1187872	-0.9324737
0.62001531	-1.1801199
-0.3238491	0.03465465
0.61826294	-0.0423909
0.39452736	1.78509729
0.39636217	-0.1417721
-1.5341996	0.00230302
0.29960747	-1.9045671
-0.1967841	-0.3183735
1.57112751	1.8496641
-1.1140142	-0.9599237
0.82906509	1.07633145
-0.8412465	-2.222816
-0.7722165	0.79667044
-0.8098652	-1.4994675

0.88003021	0.55566517
0.38894882	-0.5763027
0.69142682	1.13763684
0.35026717	-0.7111976
0.03281802	-1.2943644
0.45718792	0.08405981
0.99661549	0.88474317
0.76760953	-0.6746298
0.13283868	1.9019371
0.8994119	1.58735312
-1.3481224	-0.8740769
0.15172909	0.70980325
0.89030553	0.15711126
-1.0170942	-0.6385243
-0.3965653	-0.6712958
1.37552494	0.47520315
-0.3445693	-0.2576432
0.82906509	-0.183187
0.51567221	-0.0857186
1.95499219	0.41288882
0.88003021	-0.5084063
-2.0724336	-2.5192862
-1.647566	-1.3904143
1.48661634	2.36486774
-0.7967468	0.09307518
-1.1921672	-0.4728665
-0.3254157	-0.8937341
0.66792821	0.55001384
-0.2559851	-0.5629424
0.66682362	-0.4674776
0.39636217	0.25435374
-0.6111145	-0.1482902
1.17478233	1.04218262
-0.2252317	0.4776778
0.42844505	1.88437647
1.25137076	2.30658962
0.9161714	1.42958771
-0.0152612	1.31002642
0.90171465	1.56594437
0.62001531	0.0522141
-0.6111145	0.2851563
-0.7859525	0.46269763
-0.2900763	-1.0748043
1.50438906	0.15173288
-0.5146739	-0.4286167
0.19215338	9.54E-05
-2.5464688	0.6957899
1.18942374	-0.2734061

-2.7413987	-1.0165687
-0.6465241	-1.0600865
0.09485513	-0.3683625
-1.6445221	-1.8195817
2.28104363	0.6622042
0.80221884	-0.2198048
0.95871921	-0.5487843
-0.4901333	-0.0440311
0.89689791	0.39005349
0.19122282	0.64021925
0.32403232	0.14632711
0.29960747	0.51667761
1.16232624	0.99452811
1.73207712	0.60493741
0.11258922	-0.5696545
0.79962128	0.19335206
1.32555658	1.32369728
-0.5355354	-1.240193
-1.4903514	-0.3347809
-0.0894633	-0.8091599
-1.6445221	1.19963824
-1.001532	0.35735903
2.23525528	-0.3638722
-0.6829502	-0.6264753
0.62001531	0.88474317
1.1002717	2.16326363
-1.2981934	-0.999602
1.96976321	1.31438719
0.07408975	0.55435334
-0.2733651	-0.2035881
0.80593743	0.54075228
0.02268031	0.59757126
-0.3231751	1.05137504
-0.9970883	-2.0083055
0.96033984	2.38672938
-0.4668689	1.68265212
-0.276374	0.6057364
-1.0170942	-0.2198048
0.45867667	-0.183187
-0.2782413	1.07633145
-0.9596509	-1.7237429
-1.0138078	2.40497143
-1.0768218	-0.4483916
-0.4713359	-0.8932855
0.81179927	1.86536964
-0.5141319	-1.5233696
0.41502027	0.82786285
-0.5648132	-0.5426746

0.46803822	0.26204686
-0.1259538	-0.372826
1.3298945	-0.1146244
0.48131447	1.68871248
-1.421437	-1.7171822
0.24901842	-0.0730457
0.82906509	-1.0040254
0.44628173	-0.5629424
-2.075055	-0.8946113
0.5572961	0.02892968
0.48225762	1.1133979
-0.2733651	0.67767417
1.27496345	0.08405981
0.18867112	-0.6118496
0.03281802	-1.966624
0.17654456	-0.0377219
-0.9144088	-0.5084063
0.15045599	-0.9095103
-1.1026097	-0.3638722
0.32074193	0.6287148
-0.5930518	-0.6712958
0.90171465	0.75382418
0.0705913	0.15827449
0.15045599	-0.5084063
-0.9055271	-0.5197024
-0.9861411	-2.1177119
0.41533062	-0.3356293
1.17447958	1.42529779
-0.2309168	-0.8509563
-1.0701245	-0.7569871
0.35026717	-0.7111976
1.20783258	1.61563092
-1.1376722	0.90526409
-0.1427416	0.77647543
-0.1323822	-0.7209288
2.14464785	0.6622042
0.42239882	0.18643012
1.0442671	0.44436633
1.75598622	1.61882369
0.78518601	-0.2060553
-0.1531583	-0.7978388
-0.021896	0.47001607
-0.1187872	-1.4688044
0.59418561	0.48977803
-0.0029101	0.14982398
-1.0089501	-0.2770455
-2.0114766	-2.1367269
0.0705913	0.57454433

-0.9511727	-1.2454523
-0.8601857	-0.0901263
-1.1472187	-0.3347809
0.64965594	0.88489616
0.06070744	0.75621021
0.15045599	-0.0857186
-1.3820308	-1.3533025
-0.9011276	2.0087867
0.25111415	1.2637708
-0.7473984	-1.1098099
0.48955547	0.26424
0.19122282	1.62876278
0.08858034	0.2504103
-0.0507339	1.56618445
1.27496345	0.48977803
-0.7377751	-0.5763027
0.51567221	-0.0857186
-0.9669612	-1.4317117
-0.8133456	-0.4196301
-1.2164143	-0.4820005
-0.649019	-0.2643728
0.32764306	1.20614028
0.19215338	0.41982113
0.42855585	0.72275019
0.16518134	-0.8828678
-1.2300955	-0.0594662
0.01693919	0.20717412
0.41533062	0.30616383
-0.4574295	-0.5533592
-0.328067	-1.2444165
-0.1608135	-0.8110236
0.19818366	0.42839427
-0.5487076	-1.8479908
-0.4793499	0.44204027
-0.649019	-0.2643728
-1.3591125	-0.6385243
0.90948713	0.28233215
0.16518134	-0.0594662
-1.1352108	0.22917504
0.24901842	-0.291069
-0.2971853	-0.7075379
-0.6786141	-0.6245138
-0.7035122	-0.2576432
0.86649444	-0.1156069
-0.6669228	-1.4231195
0.15713245	0.72118425
0.83925158	-0.4728665
-0.4348674	0.87444551

0.10489827	-0.637084
-0.0563787	-1.4811021
-0.2900763	0.56796124
0.28085833	0.52244291
-0.0337019	0.10047938
1.63397207	1.29137582
0.18867112	-1.1647696
-0.4225656	-0.9746556
-0.1059205	-0.4026838
0.41533062	0.09122931
-0.3965653	-0.6712958
0.52831734	-0.0594662
-0.1259538	0.47520315
-0.4793499	-0.9611361
1.59938284	0.05319939
-1.8788306	-1.1801199
-0.7967468	0.51667761
-1.5944332	0.01218233
-0.3317131	-1.1727047
-1.1809221	1.40175052
-0.5600744	-0.2371752
0.09519178	0.17350222
1.95745966	1.62774314
0.17654456	-0.0377219
0.18410399	-0.4420973
-0.1967636	-0.0594662
1.25137076	-0.2735526
-0.3999938	-0.3956022
-1.4979064	0.03553497
-0.3130828	0.54075228
0.51327876	0.80230474
-0.3445693	-0.2576432
0.05085189	1.50212177
0.81404061	0.46411979
0.69090048	0.20717412
-0.6167111	0.92758497
-0.1259538	0.0522141
-0.5521115	0.32518325
-1.0701245	0.09122931
-1.1611051	0.69140001
1.25137076	0.37037346
-0.4713359	0.40350948
0.20928723	0.44204027
-0.4572895	0.76979716
0.19122282	1.24083467
0.13283868	1.9019371
1.15808454	0.46411979
0.53013696	0.75266956

1.15843691	2.27046126
-0.8601857	-0.8381369
0.08858034	1.46822202
-0.4695892	-0.2039627
-0.3080782	-1.2326595
-0.5440282	0.84326187
-1.699907	-1.832695
1.17948541	0.98461405
2.57733601	0.92607838
0.51567221	-0.0857186
1.32191169	1.24083467
-0.3886968	0.96655496
0.01049711	0.93228883
1.85667775	2.12281069
0.15045599	1.92307514
