# Supplementary material for: Modelling maternal and perinatal risk factors to predict poorly controlled childhood asthma
Source: PLoS One. 2021 May 27;16(5):e0252215. doi: 10.1371/journal.pone.0252215 (PMC8158992; doi:10.1371/journal.pone.0252215)
Supplement: S1 File — This file includes supplementary method descriptions, supplementary figure descriptions as well as supplementary tables. (DOCX) [file pone.0252215.s012.docx]

**Modelling maternal and perinatal risk factors to predict poorly controlled childhood asthma**

Samuel Schäfer^1,2^, Kevin Wang^3^, Felicia Sundling^1^, Jean Yang^3,4^, Anthony Liu^1,5^, Ralph Nanan^1,5,*^

^1^) Sydney Medical School – Nepean, Discipline of Paediatrics and Child Health, The University of Sydney, Sydney, NSW, Australia

^2^) Centre for Personalized Medicine, Linköping University, Linköping, Sweden

^3^) School of Mathematics and Statistics, The University of Sydney, Sydney, Australia

^4^) The Judith and David Coffey Life Lab, Charles Perkins Centre, University of Sydney, Sydney, Australia

^5^) Charles Perkins Centre – Nepean, The University of Sydney, Sydney, Australia

*) Corresponding author. E-mail: [ralph.nanan@sydney.edu.au](mailto:ralph.nanan@sydney.edu.au) (RN)

# Supplementary Methods

Perinatal growth curves were created based on all 50352 children in eMaternity passing inclusion and exclusion criteria. We converted birth weight, length and head circumference (HC) into gestational age and sex adjusted z-scores using the *lms()* function in the *gamlss [36]* package in R. This process made anthropometric measurements comparable between children of different gestational age and sex. In order to exclude outliers from our material, while still allowing for differences between populations, we considered only birth weights, lengths or HCs within |z| < 4 of the INTERGROWTH-21 [37] growth charts.

As there is a great need for reference curves for small gestational ages, we created growth curves starting from 22 weeks gestation **(S1-S3 Fig)**. In total, male growth curves for birth weight, length and HC were based on the anthropometric parameters of 25683, 24835 and 25150 male children. The corresponding numbers for female growth curve creations are 24039, 23563 and 22984. More detailed information can be found in **S2 Table.**

# Supplementary tables

| **Supplementary table 1. Full list of recorded variables, derived from both databases, and according definitions.** | | |
| --- | --- | --- |
| **Recorded variable** | **Database** | **Definition** |
| Maternal medical record number (MRN) | Obstetric database | Unique, patient-specific number. |
| Maternal date of birth | Obstetric database |  |
| Pre-pregnancy weight | Obstetric database | In kilogram. |
| Height | Obstetric database | In centimetre. |
| Gravidity | Obstetric database | number of prior pregnancies |
| Parity | Obstetric database | number of prior viable pregnancies (>20 weeks gestation) |
| Threatened premature labour | Obstetric database | spontaneous onset of labour prior to 37 weeks gestation |
| Antepartum haemorrhage | Obstetric database | haemorrhage after 20 weeks gestation |
| Diabetes | Obstetric database | Defined using standard plasma glucose cut-offs, as recommended by the International association of diabetes and pregnancy study groups consensus panel, after a 75 g oral glucose tolerance test (OGTT). All mothers were offered and strongly encouraged to undergo an OGTT from 24 - 29 weeks gestation, additionally early screening was offered to mothers with risk factors. Coded as yes or no. |
| Alcohol consumption | Obstetric database | Coded as yes or no. |
| Smoking status | Obstetric database | Coded as yes or no. |
| Illegal drug intake | Obstetric database | Coded as yes or no. |
| Infant medical record number (MRN) | Obstetric database | Unique, patient-specific number. |
| Date of birth | Obstetric database |  |
| Infant sex | Obstetric database | Male or female. |
| Gestational age | Obstetric database | In weeks. |
| Birth weight | Obstetric database | In gram. |
| Birth length | Obstetric database | In centimetre. |
| Birth HC | Obstetric database |  |
| ACB-pH | Obstetric database |  |
| ACB-lactate | Obstetric database | mg/dL |
| ACB-BE | Obstetric database | mEq/L |
| Neonatal outcome | Obstetric database | Livebirth or stillbirth. |
| Feeding discharge | Obstetric database | Breastmilk or infant formula. Feeding combinations including breast milk were counted coded breastmilk. |
| Infant medical record number (MRN) | Paediatric database | Unique, patient-specific number. |
| Date of birth | Paediatric database |  |
| Infant sex | Paediatric database | Male or female. |
| Allergies | Paediatric database | Coded yes or no. Either reported by parent or detected by skin prick test. |
| Admission cause | Paediatric database | Coded as asthma or non-asthma. |
| Abbreviations: HC, head circumference; ACB, arterial cord blood; BE, base excess. | | |

| **Supplementary Table 2. Listing n children that were included in the creation of all growth curves according to gestational age.** | | | | | | |
| --- | --- | --- | --- | --- | --- | --- |
|  | **Male** | | | **Female** | | |
| **Gestation age interval** | **Birth weight** | **Birth length** | **Birth HC** | **Birth weight** | **Birth length** | **Birth HC** |
| 21+0 to 21+6 | 1 | 1 | 1 | - | - | - |
| 22+0 to 22+6 | 8 | 7 | 7 | 10 | 9 | 8 |
| 23+0 to 23+6 | 11 | 4 | 7 | 12 | 5 | 9 |
| 24+0 to 24+6 | 21 | 11 | 15 | 26 | 12 | 15 |
| 25+0 to 25+6 | 25 | 14 | 19 | 37 | 21 | 22 |
| 26+0 to 26+6 | 45 | 21 | 32 | 32 | 18 | 27 |
| 27+0 to 27+6 | 46 | 25 | 37 | 37 | 20 | 28 |
| 28+0 to 28+6 | 72 | 42 | 55 | 67 | 42 | 53 |
| 29+0 to 29+6 | 64 | 46 | 56 | 62 | 45 | 53 |
| 30+0 to 30+6 | 109 | 91 | 107 | 82 | 64 | 81 |
| 31+0 to 31+6 | 191 | 150 | 170 | 125 | 98 | 118 |
| 32+0 to 32+6 | 198 | 174 | 188 | 214 | 174 | 196 |
| 33+0 to 33+6 | 328 | 301 | 308 | 265 | 242 | 258 |
| 34+0 to 34+6 | 395 | 357 | 376 | 394 | 358 | 383 |
| 35+0 to 35+6 | 528 | 498 | 504 | 516 | 484 | 494 |
| 36+0 to 36+6 | 1051 | 993 | 1013 | 955 | 901 | 928 |
| 37+0 to 37+6 | 2216 | 2150 | 2166 | 2094 | 1990 | 2054 |
| 38+0 to 38+6 | 5284 | 5153 | 5211 | 4620 | 4437 | 4531 |
| 39+0 to 39+6 | 6638 | 6512 | 6548 | 6304 | 6117 | 6228 |
| 40+0 to 40+6 | 5366 | 5256 | 5286 | 5164 | 4994 | 5092 |
| 41+0 to 41+6 | 3027 | 2972 | 2987 | 2985 | 2917 | 2949 |
| 42+0 to 42+6 | 59 | 57 | 57 | 33 | 31 | 31 |
| 43+0 to 43+6 | - | - | - | 5 | 5 | 5 |
| Total n children | 25683 | 24835 | 25150 | 24039 | 22984 | 23563 |
